# From Monocyclization to Pentacyclization: A Versatile Plant Cyclase Produces Diverse Sesterterpenes with Anti‐Liver Fibrosis Potential

**DOI:** 10.1002/advs.202415370

**Published:** 2025-01-10

**Authors:** Kai Guo, Xue Tang, Yan‐Chun Liu, Hui‐Zhen Cheng, Huan Liu, Yu‐Zhou Fan, Xiao‐Yu Qi, Rui Xu, Juan‐Juan Kang, De‐Sen Li, Guo‐Dong Wang, Jonathan Gershenzon, Yan Liu, Sheng‐Hong Li

**Affiliations:** ^1^ State Key Laboratory of Southwestern Chinese Medicine Resources, and Innovative Institute of Chinese Medicine and Pharmacy Chengdu University of Traditional Chinese Medicine Chengdu 611137 P. R. China; ^2^ State Key Laboratory of Phytochemistry and Natural Medicines, and Yunnan Key Laboratory of Natural Medicinal Chemistry Kunming Institute of Botany Chinese Academy of Sciences Kunming 650201 P. R. China; ^3^ State Key Laboratory of Plant Genomics and National Center for Plant Gene Research Institute of Genetics and Developmental Biology The Innovative Academy of Seed Design Chinese Academy of Sciences Beijing 100101 P. R. China; ^4^ Max Planck Institute for Chemical Ecology 07745 Jena Germany

**Keywords:** anti‐liver fibrosis, *Capsella bursa‑pastoris*, cyclizations, sesterterpene synthase, sesterterpenes

## Abstract

A prolific multi‐product sesterterpene synthase CbTPS1 is characterized from the medicinal Brassicaceae plant *Capsella bursa‐pastoris*. Twenty different sesterterpenes including 16 undescribed compounds, possessing 10 different mono‐/di‐/tri‐/tetra‐/penta‐carbocyclic skeletons, including the unique 15‐membered macrocyclic and 24(15→14)‐*abeo*‐capbuane scaffolds, are isolated and structurally elucidated from engineered *Escherichia coli* strains expressing CbTPS1. Site‐directed mutagenesis assisted by molecular dynamics simulations resulted in the variant L354m with up to 13.2‐fold increased sesterterpene production. These structurally diverse products suggest a comprehensive cyclization mechanism for plant sesterterpenes and provide compelling evidence for the initial cyclization of geranylfarnesyl diphosphate via a crucial 15‐membered monocyclic carbocation. The activities of these sesterterpenes against liver fibrosis is inferred from the inhibition of the transforming growth factor‐β/Smad signaling pathway and collagen synthesis. These findings greatly expand the chemical space and biological functions of sesterterpenes and provide new insights into the catalytic mechanism of terpene synthases.

## Graphical abstract

A versatile sesterterpene synthase, CbTPS1, is characterized from the medicinal plant *Capsella bursa‐pastoris*, producing 20 sesterterpenes including 16 new ones with 10 varied mono‐/di‐/tri‐/tetra‐/penta‐cyclic skeletons, including the novel 15‐membered macrocyclic and 24(15→14)‐*abeo*‐capbuane scaffolds. A key enzyme variant L354m with 2.0‐ to 13.2‐fold increased production is described. The anti‐liver fibrosis activity of these sesterterpene products via transforming growth factor‐β/Smad signaling pathway is observed.

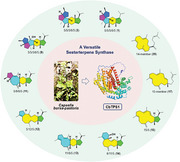

## 1 Introduction

Terpenoids constitute the most diverse class of natural products with over 100 000 documented compounds. In comparison to the large subgroups of sesquiterpenoids (C_15_), diterpenoids (C_20_), and triterpenoids (C_30_), to date, only ≈1500 sesterterpenoids (C_25_) have been discovered from a range of sources including marine invertebrates, plants, fungi, bacteria, and insects. Despite this limited number of members, sesterterpenoids exhibit impressive structural diversity and complexity with chemical scaffolds ranging from acyclic, mono‐carbocyclic to hexa‐carbocyclic systems. They also possess a broad spectrum of biological properties, including immunosuppressive, anti‐adipogenic, antineoplastic, antimicrobial, and antifeedant activities.^[^ ^1^, ^2^ ^]^

**Figure 1:**
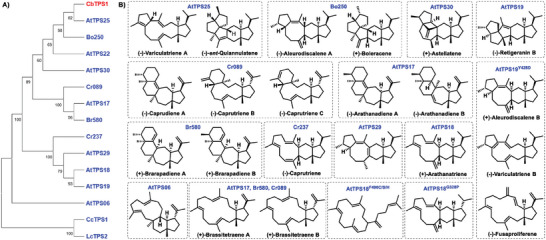
Plant sesterterpene synthases (StTSs) and chemical structures of their products. A) Phylogenetic tree of all characterized plant StTSs (see the Supporting Information for details). The candidate StTS investigated in this work is marked in red while the previously reported ones are in blue. B) Chemical structures of sesterterpenes generated by the reported Brassicaceae StTSs and their enzyme variants.^[^ ^5^, ^6^, ^7^, ^8^ ^]^

Biosynthetically, the chemical skeletons of sesterterpenoids are constructed by sesterterpene synthases (StTSs) utilizing the acyclic C_25_ precursor geranylfarnesyl diphosphate (GFDP) as a common substrate. The majority of StTSs have been identified from fungi, whereas plant‐derived and bacterial StTSs are comparatively underexplored.^[^ ^3^ ^]^ Unlike fungal StTSs, which contain both a prenyltransferase (PT) domain and a terpene cyclization (TC) domain, plant StTS genes are often clustered with GFDP synthase (GFDPS) genes in genomes, suggesting that their expression is regulated in common.^[^ ^3^ ^]^ Apart from CcTPS1 and LcTPS1, the two Lamiaceae StTSs that produce eight mono‐carbocyclic sesterterpenes,^[^ ^4^ ^]^ the other 12 plant StTSs have all been reported from the Brassicaceae family and are responsible for the production of at least 24 sesterterpenes (**Figure** **1**).^[^ ^5^, ^6^, ^7^, ^8^ ^]^ However, more research is still needed to better understand the cyclization process of plant StTSs from the Brassicaceae and document the diversity of products.

The genus *Capsella*, closely related to *Arabidopsis*, contains only three species: the tetraploid *C. bursa‐pastoris* and two diploids *C. rubella* and *C. grandiflora*. Among them, *C. bursa‐pastoris* is notable for its wide geographical distribution and adaptation to various environmental conditions.^[^ ^9^ ^]^ Besides, as a popular wild vegetable, *C. bursa‐pastoris* is valued for its dual medicinal and dietary properties, and used for calming the liver, improving eyesight, and reducing fever in traditional Chinese medicine.^[^ ^10^ ^]^ Recently, a chromosome‐scale assembly and a genetic map of *C. bursa‐pastoris* genome have been reported.^[^ ^11^ ^]^ In this study, we focus on the functional characterization of a StTS (CbTPS1) from *C. bursa‐pastoris* that produces a panel of 20 mono‐/di‐/tri‐/tetra‐/penta‐carbocyclic sesterterpenes, including 16 new structures and two novel skeletons, a 15‐membered macrocycle and a 24(15→14)‐*abeo*‐capbuane skeleton. A vital amino acid residue for the enzyme activity was determined by molecular dynamics (MD) simulations and mutagenesis experiments, resulting in the construction of an enzyme variant with remarkably increased sesterterpene production. The in vitro anti‐liver fibrosis activity of these sesterterpene products was also demonstrated.

## 2 Results and Discussion

### 2.1 Identification of CbTPS1 From *C. Bursa‐Pastoris* and Three Major Sesterterpene Products

The genome (GenBank: GCA_036452645.1) and assembled transcriptome of *C. bursa‐pastoris* were used to search for candidate StTSs.^[^ ^11^ ^]^ A potential candidate sharing 59% and 56% amino acid sequence identity with Cr089 and Cr237 (reported from *C. rubella* ^[^ ^5^, ^7^ ^]^) respectively, drew our attention. Accordingly, the full‐length coding sequence (CDS) of this candidate gene, designated as *CbTPS1* (GenBank: PQ213475), was amplified by reverse transcription polymerase chain reaction (RT‐PCR) from the mRNA of *C. bursa‐pastoris*. Maximum‐likelihood phylogenetic analysis (Figure 1A; Figure S1, and Table S1, Supporting Information) showed that CbTPS1 fell within the clade of Type A StTSs belonging to the TPS‐a subfamily. Multiple sequence alignment with the known Brassicaceae StTSs (Figure S2, Supporting Information) indicated that CbTPS1 contained the typical “DDXXD” and “NSE/DTE” conserved motifs.

**Figure 2:**
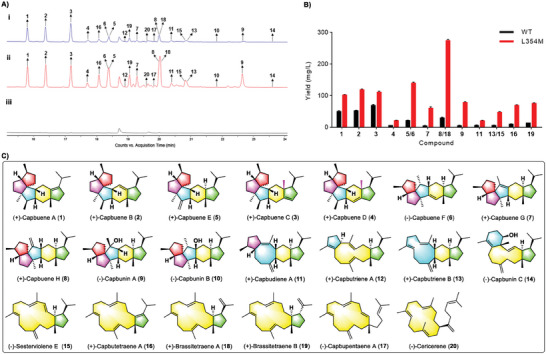
Functional characterization of CbTPS1 and its enzyme variant CbTPS1^L354M^. A) Total ion chromatograms (TICs) of GC‐MS analysis of the sesterterpenes produced by engineered *E. coli* harboring CbTPS1 i), CbTPS1^L354M^ ii), and empty vector iii), respectively. B) Sesterterpene yields of the wild type CbTPS1 (WT) and the enzyme variant CbTPS1^L354M^ (L354m) determined using an *E. coli* expression system. C) Chemical structures of compounds **1**–**20**, corresponding to peaks 1–20 in (A).

For functional characterization, CbTPS1 was heterologously expressed in *E. coli*. The GFPP‐producing engineered *E. coli* strain harboring two plasmids was constructed, in which a complete gene set of the mevalonate (MVA) pathway and a farnesyl diphosphate (FDP) synthase gene were integrated into one plasmid (pBbA5c‐MevT‐MBIS),^[^ ^12^ ^]^ while an isopentenyl diphosphate (IDP) isomerase (idi), a PT domain of AaTPS1 (AaTPS1‐PT),^[^ ^13^ ^]^ and two rate‐limiting enzymes of the *E. coli* methylerythritol 4‐phosphate (MEP) pathway, deoxyxylulose 5‐phosphate reductoisomerase (DXR)/deoxyxylulose 5‐phosphate sythase (DXS), were integrated into a second plasmid (RSPT). The full‐length cDNA of CbTPS1 was cloned into the vector pMAL‐c2x, and transformed into the GFPP‐producing *E. coli*, generating the engineered *E. coli* strain EC‐CbTPS1. Gas chromatography–mass spectrometry (GC–MS) analysis of the fermentation product indicated that EC‐CbTPS1 produced a suite of sesterterpenes with characteristic molecular ion peaks at *m*/*z* 340, three of which (peaks 1–3) were the major components (**Figure** **2**A; Figure S3, Supporting Information).

**Figure 3:**
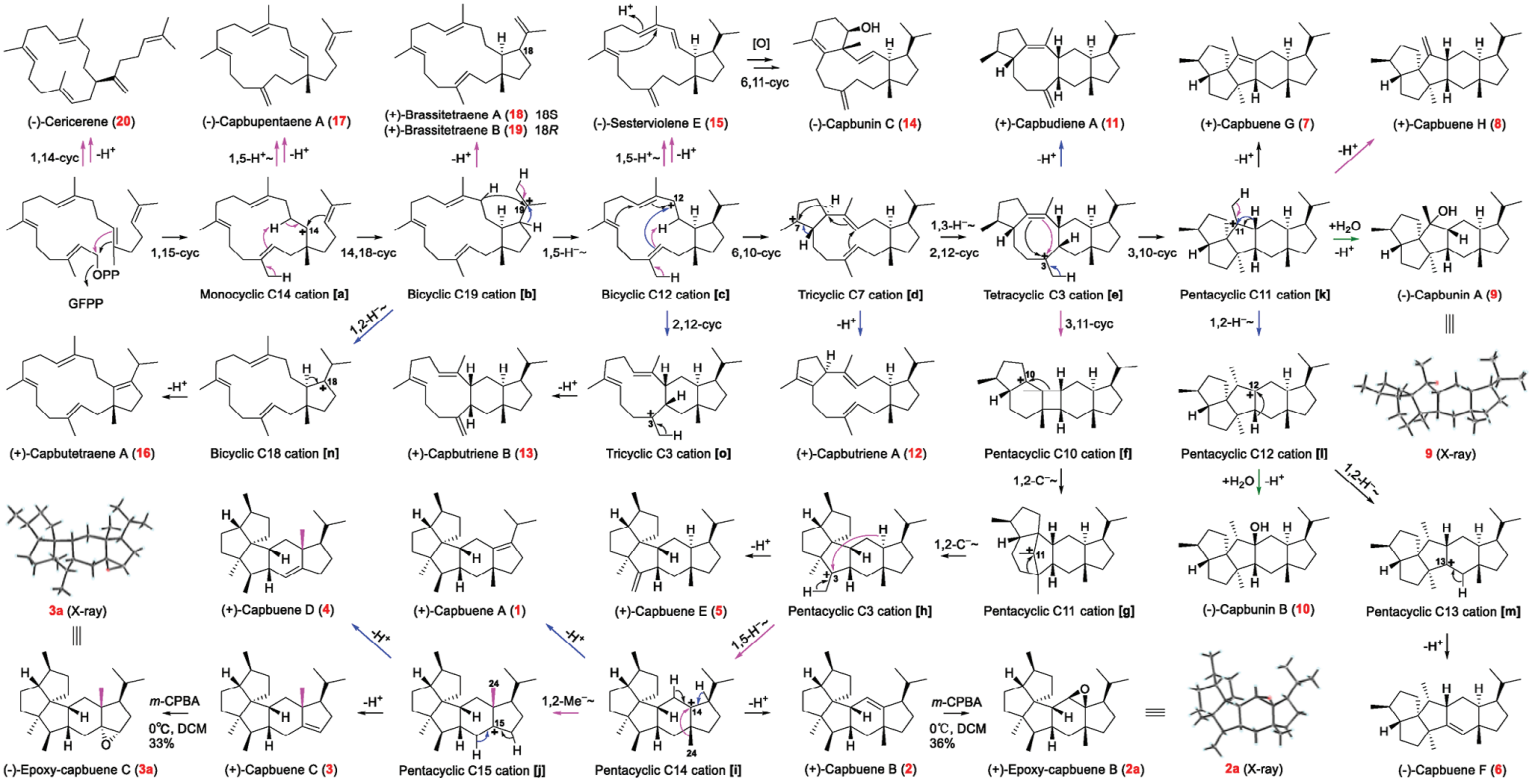
Proposed cyclization paths toward the formation of sesterterpenes **1**–**20**. Different line colors indicate different cyclization paths. Epoxides **2a** and **3a** were synthesized by epoxidation with *meta*‐chloroperoxybenzoic acid (*m*‐CPBA) for X‐ray diffraction analysis.

To obtain sufficient amounts of compounds **1**–**3** for structural elucidation, EC‐CbTPS1 were cultured in a large scale, and the resultant metabolites were extracted with EtOAc and purified by chromatographic methods. Through comprehensive nuclear magnetic resonance (NMR) analysis (Tables S3, S4, and S6, Supporting Information), the planar structures and relative configurations of **1**–**3** were established. In addition, the epoxides **2a** and **3a** of compounds **2** and **3** were chemically synthesized, and suitable single crystals were successfully obtained for the X‐ray diffraction analysis, which thus unambiguously defined their structures including absolute configurations (Figures 2C and **3**). Compounds **1** and **2**, sharing a common 5/5/5/6/5 fused penta‐carbocyclic scaffold (named capbuane herein), were trivially named capbuenes A and B respectively. So far only one known compound, *ent*‐quiannulatene (produced by AtTPS25) possessing the same scafold (Figure 1), was reported.^[^ ^5^ ^]^ Compound **3**, named capbuene C, has an intriguing methyl migration, furnishing a novel 5/5/5/6/5 penta‐carbocyclic skeleton [24(15→14)‐*abeo*‐capbuane]. However, due to very low abundance, attempts to isolate and identify the other sesterterpene products (peaks after *t*_R_ 17.5 min) failed.

### 2.2 Construction of the Enzyme Variant CbTPS1^L354M^ Increases Sesterterpene Production

**Figure 4:**
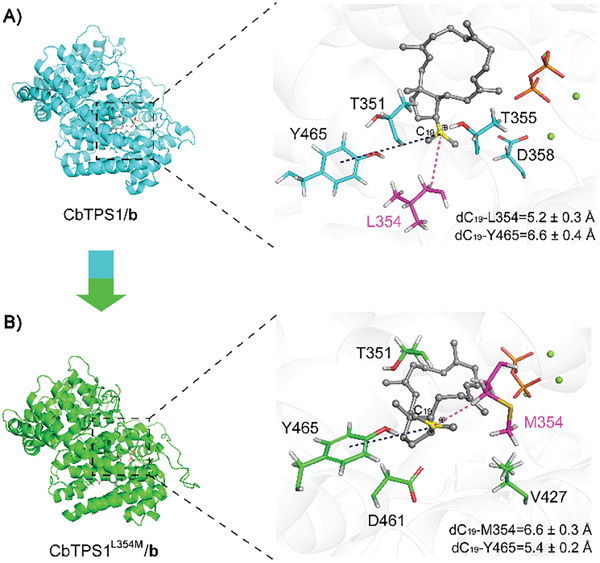
Representative MD snapshots of A) CbTPS1/**b** (in blue) and B) CbTPS1^L354M^/**b** (in green) and key residues surrounding **b**. Intermediate **b**, residues L354/M354, PPi, and Mg^2+^ are colored in gray, magenta, orange, and green, respectively.

To improve the sesterterpene production (especially those with low abundance), site‐directed mutagenesis of CbTPS1 assisted by molecular dynamics (MD) simulations was performed. In the cyclization paths catalyzed by Type A StTSs, the bicyclic C19 cation (**b**) is generally regarded as a common intermediate (Figure 3).^[^ ^5^ ^]^ Hence, MD simulations of the CbTPS/**b** complex using an AlphaFold‐generated model were performed. Analysis of the MD trajectories (**Figure** **4**A) revealed that five residues (Y465, T351, T355, D358, and L354) were located in the D‐helix region within the active pocket of CbTPS1. Alanine scanning mutagenesis experiments of these residues was carried out. The results revealed that alanine substitution of L354 decreased the production of the three major sesterterpenes **1**–**3**, but significantly increased some minor products such as the peaks with retention times ≈20 min (Figure S3, Supporting Information), indicating a crucial role of L354. Then, saturation mutagenesis experiments on L354 were carried out. Notably, the variant L354m resulted in a remarkable increase in the production of nearly all sesterterpene products (2.0‐ to 13.2‐fold higher than the wild‐type), and an overall increase in the total sesterterpene production (approximately four times higher than the wild‐type) (Figure 2A,B; Table S32, Supporting Information). It has been well established that aromatic residues play an important role in stabilizing carbocation intermediates during terpene cyclization,^[^ ^14^ ^]^ and MD trajectory analysis of CbTPS1/**b** and CbTPS1^L354M^/**b** (Figure 4) indicated that Y465 was a key aromatic residue in stabilizing **b** by cation‐π interaction. The variant L354m might enhance the cation‐π interaction and facilitate further cyclization of **b** via bridging the distance between Y465 and the positive charged C19 of **b** (from 6.6 to 5.4 Å), thus ultimately improving the sesterterpene production.

Accordingly, a large‐scale cultivation of the engineered *E. coli* strain harboring the variant L354m (EC‐CbTPS1^L354M^) was carried out, enabling the isolation of pure sesterterpenes **4**–**20** for structural elucidation by extensive NMR analysis. Among them, capbuene D (**4**) shares the same 24(15→14)‐*abeo*‐capbuane penta‐carbocyclic skeleton as **3** (Table S8, Supporting Information). Capbuene E (**5**), differing from boleracene (produced by Bo250) only in the C‐7 configuration (Figure 1),^[^ ^5^ ^]^ has the same 5/5/5/6/5 fused penta‐carbocyclic scaffold as **1** and **2**. Compounds **6**–**10** represent another new 5/5/5/6/5 fused penta‐carbocyclic scaffold, distinct from that of retigeranin B (produced by AtTPS19)^[^ ^5^ ^]^ and the retigeranes (isolated from lichens of the Lobariaceae)^[^ ^15^ ^]^ in the configurations of the 5/5/5 ring system (at C3, C6, C7, and C10 positions). The absolute configuration of the hydrated product, capbunin A (**9**), was confirmed by X‐ray diffraction analysis (Figure 3; Table S13, Supporting Information). It was noteworthy that production of capbunin A (**9**) by CbTPS1^L354M^ showed the highest increase, approximately 13.2‐fold higher than the yield of wild‐type enzyme (Table S32, Supporting Information). Capbudiene A (**11**), with a 5/8/6/5 fused ring, is the sole tetra‐carbocyclic sesterterpene produced by both CbTPS1 and CbTPS1^L354M^. The configuration of **11** was deduced from electronic circular dichroism (ECD) calculations (Table S15, Supporting Information), and was different from the known tetra‐carbocyclic sesterterpenes (produced by AtTPS29 and AtTPS19^Y428D^)^[^ ^5^, ^6^ ^]^ at C6, C7, and C12. It was interesting to find that **11** is a geometric isomer of lentzeadiene, the main product of a bacterial StTS (LaLDS).^[^ ^3^ ^]^ Three tri‐carbocyclic sesterterpenes, capbutrienes A–B and capbunin C (**12**–**14**) having a 5/12/5, 11/6/5, and 6/11/5 fused ring, respectively, were minor products identified for the first time, and their absolute configurations were assigned by ECD calculations (in the case of **14** assisted by DP4+ analysis of NMR calculations) (Tables S16–S19, Supporting Information). The four 15/5 di‐carbocyclic sesterterpenes comprise a new compound capbutetraene A (**16**), as well as three known ones, sesterviolene E (**15**), and brassitetraenes A (**18**) and B (**19**), which were previously reported as the products of fungal SvSS (for **15**)^[^ ^3^ ^]^ or three plant StTSs (for **18** and **19**).^[^ ^7^ ^]^ Among the two mono‐carbocyclic sesterterpene products, capbupentaene A (**17**) features a rare 15‐membered ring scaffold, while the known compound cericerene (**20**) with a 14‐membered ring is secreted by a scale insect *Ceroplastes ceriferus* ^[^ ^16^ ^]^ and also produced by the AtTPS18 enzyme variant.^[^ ^6^ ^]^ Intriguingly, the enantiomer of **20** was previously identified as one of the prodcuts of LcTPS2,^[^ ^4^ ^]^ as well as enzyme variants of SvES (bacterial sesquiterpene synthase)^[^ ^17^ ^]^ and VenA (bacterial diterpene synthase).^[^ ^18^ ^]^

### 2.3 Cyclization Pathways to the Formation of Mono‐, bi‐, tri‐, tetra‐, and Penta‐Carbocyclic Scaffolds of Sesterterpenes

Given that enzyme products could provide insights into the carbocation intermediates involved in their formation,^[^ ^19^ ^]^ we delved into the cyclization mechanism of CbTPS1 (Figure 3) assisted by the 20 identified sesterterpene products. Although the bicyclic intermediate cation **b** is commonly regarded as the initially‐formed cation,^[^ ^5^, ^20^ ^]^ this study demonstrated that a monocyclic C14 cation **a** likely participates in the formation of **b**, resulting in a concerted but asynchronous bicyclization process from the acyclic GFDP to the bicyclic carbocation. This is supported by the isolation and characterization of compound **17** derived from the intermediate cation (**a**). Apart from the 1,15‐cyclization of GFDP to form **a**, compound **20** was generated via a 1,14‐cyclization of GFDP. Following the 14,18‐cyclization process from **a** to **b**, subsequent deprotonation of **b** resulted in a pair of bicyclic 18‐epimers (**18** and **19**). Alternatively, the bicyclic C19 cation **b** can undergo a 1,5‐hydride migration to form **c**, or a 1,2‐hydride migration and deprotonation to give **16**. Despite their low yields, it is noteworthy that the production of **15** and **14** from **c** via deprotonation‐reprotonation and 6,11‐cyclization processes closely resembles the catalytic processes of previously reported StTSs belonging to clade IV (Cr089, AtTPS17, and Br580) (Figure 1B).^[^ ^7^ ^]^ The other two minor tricyclic products, **12** and **13**, were derived from 6,10‐ and 2,12‐cyclizations of **c**, respectively. The tricyclic C7 cation **d** can undergo a 1,3‐hydride migration and a 2,12‐cyclization furnishing the key tetracyclic cation **e**, as evident from the direct deprotonation products **12** and **11**. For the major penta‐carbocyclic sesterterpenes products of CbTPS1, their biosynthesis appears to diverge into two paths, namely, 3,10‐cyclization (to **k**) or 3,11‐cyclization (to **f**) from the common tetracyclic C3 cation **e**. The existence of the pentacyclic C11 cation **k** is evident from its deprotonation products (**7** and **8**) and hydrated product (**9**). This species undergoes successive 1,2‐hydride migrations leading to the pentacyclic C13 cation **m** as evident from the deprotonation product **6**, while another hydrated product **10** suggests the existence of a pentacyclic C12 cation **l**. The second path from **e** appears to proceed by several steps to the pentacyclic C3 cation **h** (evidenced by its deprotonation product **5**) by a mechanism similar to that of *ent*‐quiannulatene (produced by AtTPS25)^[^ ^5^ ^]^ and quiannulatene (produced by EvQS)^[^ ^20^ ^]^ via a cyclobutane intermediate **f** and a 5/6/5/6/5 pentacyclic C11 cation **g**. Subsequently, another 1,5‐hydride migration transforms **h** into the pentacyclic C14 cation **i**, as evidenced by its deprotonation products **1** and **2**. This unexpected 1,5‐hydride migration from C14 to C3 has not been observed in other plant or microbial StTSs. The isolation of products **3** and **4** suggests the formation of the 24(15→14)‐*abeo*‐capbuane pentacyclic C15 cation **j** from **i** through a 1,2‐methyl migration.

**Figure 5:**
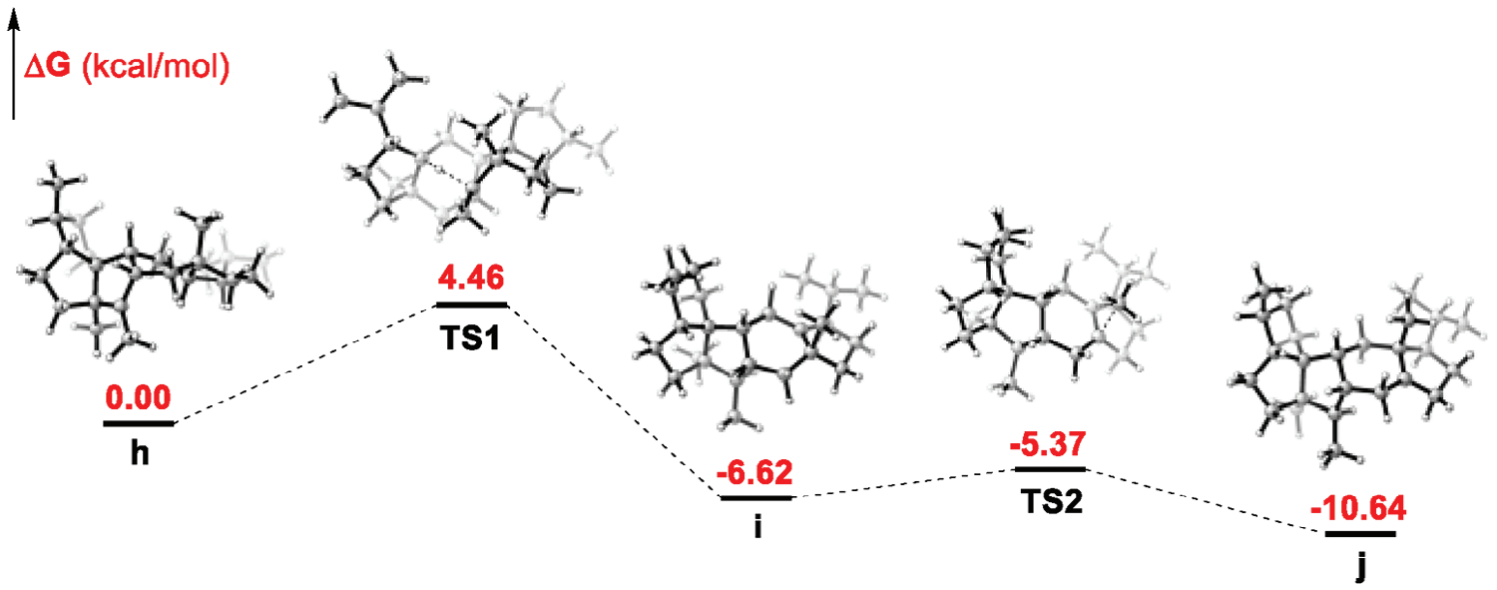
DFT calculation for carbocation intermediates and transition‐state structures along the path **h**–**i**–**j**.

**Figure 6:**
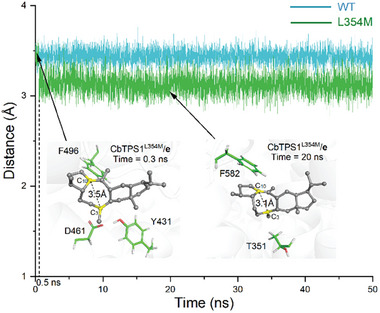
Comparison of the distances between C3 and C10 in CbTPS1/**e** and CbTPS1^L354M^/**e** by MD simulations.

Considering that compounds **1**–**3** are the major products of CbTPS1, this unusual 1,5‐hydride migration from C14 to C3 followed by 1,2‐methyl migration from C15 to C14 highlight the unique catalytic properties of this plant StTS. To probe the validity of the proposed paths **h**–**i**–**j**, density functional theory (DFT) calculations were performed. The results (**Figure** **5**) demonstrated the feasibility of a 1,5‐hydride migration (from **h** to **i**) with a free‐energy barrier of 4.46 kcal mol^−1^, as well as the high‐efficiency of the subsequent 1,2‐methyl migration (from **i** to **j**) with a free‐energy barrier of 1.25 kcal mol^−1^. With the aid of the proposed monocyclic C14 cation **a**, the MD trajectory analysis of CbTPS1/**a** and CbTPS1^L354M^/**a** were performed. The results (Figure S5, Supporting Information) indicated that the variant L354m could enhance the cation‐π interaction via bridging the distances between the positive charged C14 of **a** and the surrounding aromatic residues (F582, Y576, F496, and Y431), thus facilitating further cyclization of **a** and finally improving the overall sesterterpenes production (including the minor products). Moreover, although CbTPS1^L354M^ has generally enhanced production of all sesterterpenes, the increases of **9** (13.2‐fold), **7** (10.8‐fold), and **8** (≈9.0‐fold) were much higher than those of **1** (2.0‐fold), **2** (2.3‐fold), and **3** (1.6‐fold) (Figure 2B; Table S32, Supporting Information). This implied that the mutation of L354 with M led to a favorable influence on the 3,10‐cyclization process of **e**. To investigate the mechanism of this enhancement, MD simulations on CbTPS1/**e** and CbTPS1^L354M^/**e** were performed (**Figure** **6**). During 50 ns simulations, the distance between C3 and C10 in CbTPS1/**e** remained stable at approximately 3.4 Å, whereas this distance decreased at 0.5 ns (from 3.4 to 3.1 Å) in CbTPS1^L354M^/**e**. These results indicated that the steric hindrance within the active sites in the variant L354m might have been modified, thus promoting the 3,10‐cyclization of **e**. Such alterations in steric hindrance are often associated with product profile shifts in terpene synthases.^[^ ^18^ ^]^

### 2.4 Anti‐Liver Fibrosis Effect of Sesterterpenes Through Inhibition of the Transforming Growth Factor (TGF)‐β/Smad Signaling Pathway

**Figure 7:**
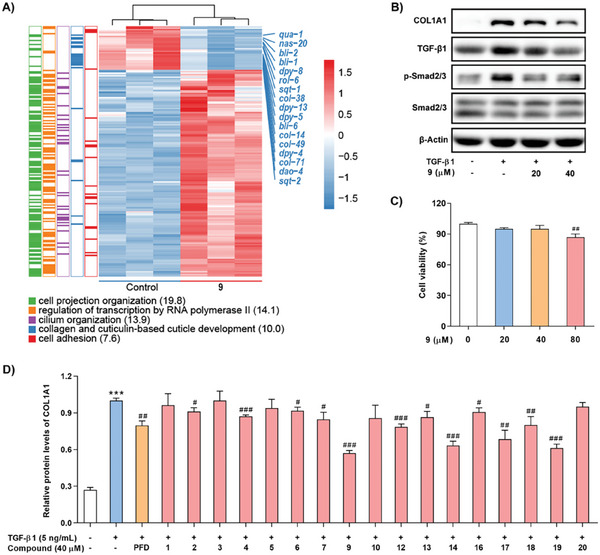
Anti‐liver fibrosis assay of the sesterterpenes. A) The top five enriched GO biological processes and DEGs in *C. elegans* treated with compound **9** at 200 µm. Numbers in brackets are –log_10_(*p*‐value). B) Western blot analysis for the expression of COL1A1, TGF‐β1, p‐Smad2/3, and Smad2/3 in TGF‐β1‐stimulated LX‐2 cells treated with indicated concentrations of compound **9**. C) Cytotoxicity assay of compound **9** on LX‐2 cells. D) ELISA for COL1A1 expression in TGF‐β1‐stimulated LX‐2 cells treated with compounds (40 µm). Data are represented as mean ± SD from three biological replicates using one‐way analysis of variance (ANOVA) (^#^ *P* < 0.05, ^##^ *P* < 0.01, and ^###^ *P* < 0.001 vs TGF‐β1‐treated group; ^***^ *P* < 0.001 vs control group).

The free‐living nematode *Caenorhabditis elegans* is a well‐established model for investigating the bioactivity and molecular mechanism of natural products.^[^ ^21^ ^]^ To explore the potential biological functions of the sesterterpenes produced by CbTPS1, RNA sequencing was performed on *C. elegans* treated with one of the major products, compound **9**, at 200 µm. The top five enriched Gene Ontology (GO) biological processes (BPs) and differentially expressed genes (DEGs) are shown in **Figure** **7**A. Most DEGs involved in the BP of collagen and cuticulin‐based cuticle development were found to be down‐regulated, and gene set enrichment analysis (GSEA) demonstrated that this BP was significantly inhibited (Figure S5, Supporting Information). Analysis of these down‐regulated DEGs indicated that the majority (*col‐14*/*38*/*49*/*71*, *dpy‐5*/*8*/*13*, *bli‐1*/*2*/*6*, *sqt‐1*/*2*, and so on) play critical roles in the encoding and formation of nematode collagen.^[^ ^22^ ^]^ As the main component of human extracellular matrix (ECM) proteins, collagen is also a representative marker of liver fibrosis,^[^ ^23^ ^]^ and western blot assay revealed that compound **9** could markedly inhibit the production of collagen type I alpha‐1 (COL1A1) in TGF‐β1 stimulated LX‐2 cells at 40 µm without cytotoxicity (Figure 7B,C). Given that the TGF‐β/Smad signaling pathway plays a central role in regulating collagen expression and the pathogenesis of liver fibrosis,^[^ ^24^ ^]^ the influence of compound **9** on the TGF‐β/Smad signaling pathway was explored, which indicated that compound **9** could significantly suppress the expression of TGF‐β1 and p‐Smad2/3 in TGF‐β1 stimulated LX‐2 cells (Figure 7B). In addition, all isolated sesterterpenes (except for **8**, **11**, and **15**) were evaluated for their COL1A1 inhibitory activity using an enzyme‐linked immunosorbent assay (ELISA), with pirfenidone (PFD) as a positive control. The results (Figure 7D) showed that most of the sesterterpenes exhibited inhibitory effects on the production of COL1A1 with different potencies, with **9**, **14**, **17**, and **19** being the most active. Comparing these structures, it seems that C‐11 hydroxylation (**9** vs **7**) and an 18*R*‐configuration (**19** vs **18**) might contribute to the anti‐liver fibrosis activity of this class of sesterterpenes.

## 3 Conclusion

In this study, we discovered and functionally characterized a multi‐product StTS (CbTPS1) from the Brassicaceae plant *C. bursa‐pastoris*, which catalyzed the formation of 16 new and four known sesterterpenes, including two mono‐carbocyclic, four di‐carbocyclic, three tri‐carbocyclic, one tetra‐carbocyclic, and 10 penta‐carbocyclic ones. These sesterterpenes encompass ten varied scaffolds, including the novel 15‐membered macrocyclic and 24(15→14)‐*abeo*‐capbuane skeletons. Site‐directed mutagenesis combined with MD simulations identified an important variant L354m that had increased production of nearly all sesterterpenes by 2.0‐ to 13.2‐fold compared to the wild‐type enzyme. On basis of these structures, we suggest a comprehensive overview of the cyclization paths from GFDP to the penta‐carbocyclic sesterterpenes, and propose a concerted but asynchronous bicyclization process from acyclic GFDP to the bicyclic cation via a monocyclic C14 cation. Although yet to be experimentally validated, this proposed asynchronous bicyclization might also be applicable to other StTSs and diterpene synthases (DTSs) of Type A. Previous studies have pointed out the potential parallels in the catalytic processes of sesterterpene synthesis between plant StTSs and the only distantly related fungal bifunctional StTSs.^[^ ^5^, ^6^ ^]^ From the present work, it is evident that this divergence likely begins at the initial 1,15‐cyclization of GFDP. In comparing CbTPS1 to other reported plant StTSs, it is intriguing to find out why their sesterterpene products all retain the same configuration at C14 and C15, but vary at some other positions (C3, C6, C7, or C12). This discrepancy is presumably due to the functional differences in the 6,10‐ or 2,12‐cyclization process. Further elaboration of the sequence and function of StTSs from Brassicaceae plants should help elucidate the mechanisms that control product configuration. Most of the sesterterpenes characterized exhibited anti‐liver fibrosis activity in vitro by inhibiting the production of collagen COL1A1, potentially via the TGF‐β/Smad signaling pathway, since the expression of TGF‐β1 and p‐Smad2/3 were both inhibited by compound **9**. These findings greatly expand our knowledge of the chemodiversity, biosynthesis, and biological activities of sesterterpenes, and will help support in‐depth studies of StTSs aimed at understanding the mechanism for generating sesterterpene diversity and exploring their biological functions.

## Conflict of Interest

The authors declare no conflict of interest.

## Supporting information



Supporting Information

## Data Availability

The data that support the findings of this study are available from the corresponding author upon reasonable request.
