# Supplementary material for: From Monocyclization to Pentacyclization: A Versatile Plant Cyclase Produces Diverse Sesterterpenes with Anti‐Liver Fibrosis Potential
Source: Adv Sci (Weinh). 2025 Jan 10;12(9):2415370. doi: 10.1002/advs.202415370 (PMC11884544; doi:10.1002/advs.202415370)
Supplement: Supplementary file 1 — Supporting Information [file ADVS-12-2415370-s001.pdf]

ADVANCED SCIENCE
Open Access

Supporting Information

for *Adv. Sci.*, DOI 10.1002/advs.202415370

From Monocyclization to Pentacyclization: A Versatile Plant Cyclase Produces Diverse Sesterterpenes with Anti-Liver Fibrosis Potential

Kai Guo, Xue Tang, Yan-Chun Liu, Hui-Zhen Cheng, Huan Liu, Yu-Zhou Fan, Xiao-Yu Qi, Rui Xu, Juan-Juan Kang, De-Sen Li, Guo-Dong Wang, Jonathan Gershenzon, Yan Liu and Sheng-Hong Li**

Supporting Information for

From Monocyclization to Pentacyclization: A Versatile Plant Cyclase Produces Diverse Sesterterpenes with Anti-Liver Fibrosis Potential

Kai Guo+[a], Xue Tang+[a], Yan-Chun Liu+[b], Hui-Zhen Cheng[a], Huan Liu[a], Yu-Zhou Fan[a], Xiao-Yu Qi[a], Rui Xu[a], Juan-Juan Kang[a], De-Sen Li[b], Guo-Dong Wang[c], Jonathan Gershenzon[d], Yan Liu*[a], and Sheng-Hong Li*[a,b]

Experimental Procedure 6
1. General procedures 6
2. Plant materials, plasmids, strains and culture conditions 6
3. Gene cloning and phylogenetic analysis 7
4. Enzyme activity assay *in vivo* using engineered *E. coli* 7
5. Molecular dynamics simulation and site-directed mutagenesis 8
6. Isolation of sesterterpenes by large-scale fermentation of engineered *E. coli* 8
7. Chemical synthesis of sesterterpene derivatives for X-ray diffraction analysis 10
8. Quantum chemical calculations and configuration determination of sesterterpenes 11
9. Quantitative analysis of metabolites in engineered *E. coli* EC-CbTPS1 and its variants 12
10. Nematodes cultivation and RNA-seq analysis 12
11. *In vitro* anti-liver fibrosis activity and western blot assay 13
12. Statistical analysis 13
References 14
Supplementary Tables 15
Table S1. Plant TPSs used for the phylogenetic analysis 15
Table S2. Primers used in this study 17
Table S3. ^{1}H (700 MHz) and ^{13}C (150 MHz) NMR data of (+)-capbuene A (**1**) in $CDCl_3$ 18
Table S4. ^{1}H (700 MHz) and ^{13}C (150 MHz) NMR data of (+)-capbuene B (**2**) in $CDCl_3$ 19
Table S5. ^{1}H (700 MHz) and ^{13}C (150 MHz) NMR data of (+)-epoxy-capbuene B (**2a**) in $CDCl_3$ 20
Table S6. ^{1}H (700 MHz) and ^{13}C (150 MHz) NMR data of (+)-capbuene C (**3**) in $CDCl_3$ 21
Table S7. ^{1}H (700 MHz) and ^{13}C (150 MHz) NMR data of (–)-epoxy-capbuene C (**3a**) in $CDCl_3$ 22
Table S8. ^{1}H (700 MHz) and ^{13}C (150 MHz) NMR data of (+)-capbuene D (**4**) in $CDCl_3$ 23
Table S9. ^{1}H (700 MHz) and ^{13}C (150 MHz) NMR data of (+)-capbuene E (**5**) in $CDCl_3$ 24
Table S10. ^{1}H (700 MHz) and ^{13}C (150 MHz) NMR data of (–)-capbuene F (**6**) in $CDCl_3$ 25
Table S11. ^{1}H (700 MHz) and ^{13}C (150 MHz) NMR data of (+)-capbuene G (**7**) in $CDCl_3$ 26
Table S12. ^{1}H (700 MHz) and ^{13}C (150 MHz) NMR data of (+)-capbuene H (**8**) in $CDCl_3$ 27
Table S13. ^{1}H (700 MHz) and ^{13}C (150 MHz) NMR data of (–)-capbunin A (**9**) in $CDCl_3$ 28
Table S14. ^{1}H (700 MHz) and ^{13}C (150 MHz) NMR data of (–)-capbunin B (**10**) in $CDCl_3$ 29
Table S15. ^{1}H (700 MHz) and ^{13}C (150 MHz) NMR data of (+)-capbudiene A (**11**) in $CDCl_3$ 30
Table S16. ^{1}H (700 MHz) and ^{13}C (150 MHz) NMR data of (+)-capbutriene A (**12**) in $CDCl_3$ 31
Table S17. ^{1}H (700 MHz) and ^{13}C (150 MHz) NMR data of (+)-capbutriene B (**13**) in $CDCl_3$ 32
Table S18. ^{1}H (700 MHz) and ^{13}C (150 MHz) NMR data of (–)-capbunin C (**14**) in $CDCl_3$ 33
Table S19. DP4+ evaluation of theoretical and experimental NMR data of (–)-capbunin C (**14**) [Isomer 1: **14** (10*R*,11*R*); Isomer 2: 10-*epi*-**14** (10*S*,11*R*)] 34
Table S20. ^{1}H (700 MHz) and ^{13}C (150 MHz) NMR data of (–)-sesterviolene E (**15**) in $CDCl_3$ 35
Table S21. ^{1}H (700 MHz) and ^{13}C (150 MHz) NMR data of (+)-capbutetraene A (**16**) in $CDCl_3$ 36
Table S22. ^{1}H (700 MHz) and ^{13}C (150 MHz) NMR data of (–)-capbupentaene A (**17**) in $CDCl_3$ 37
Table S23. ^{1}H (700 MHz) and ^{13}C (150 MHz) NMR data of (+)-brassitetraene A (**18**) and (+)-brassitetraene B (**19**) in $CDCl_3$ 38
Table S24. ^{1}H (700 MHz) and ^{13}C (150 MHz) NMR data of (–)-cericerne (**20**) in $CDCl_3$ 39
Table S25. Optimized lowest energy 3D conformers and energy analysis for **10** 40
Table S26. Optimized lowest energy 3D conformers and energy analysis for **11** 41
Table S27. Optimized lowest energy 3D conformers and energy analysis for **12** 41
Table S28. Optimized lowest energy 3D conformers and energy analysis for **13** 41
Table S29. Optimized lowest energy 3D conformers and energy analysis for **14** 42
Table S30. Optimized lowest energy 3D conformers and energy analysis for **17** 43

Table S31. Optimized lowest energy 3D conformers and energy analysis for **20** 44
Table S32. The yield of sesterterpenes produced by CbTPS1 and its variants 45
Supplementary Figures 46
Figure S1. Phylogenetic tree of the plant TPSs listed in Table S1 using the maximum-likelihood method 46
Figure S2. Multiple sequence alignment of CbTPS1 with the known plant StTSs from the Brassicaceae 47
Figure S3. Total ion chromatograms (TICs) of GC-MS analysis of the metabolites produced in engineered *E. coli* heterologously expressing CbTPS1 and its variants 48
Figures S4A–J. EI-MS spectra of compounds **1**–**8**, **2a** and **3a** 49
Figures S4K–T. EI-MS spectra of compounds **9**–**18** 50
Figures S4U–V. EI-MS spectra of compounds **19** and **20** 51
Figures S5. Representative MD snapshots of A) CbTPS1/**a** (in blue) and B) CbTPS1^{L354M}/**a** (in green) and key aromatic residues surrounding **a** 51
Figures S6. GSEA result of collagen and cuticulin-based cuticle development 51
Figure S7. ^{1}H NMR spectrum of compound **1** in $CDCl_3$ (700 MHz) 52
Figure S8. ^{13}C NMR and DEPT spectra of compound **1** in $CDCl_3$ (150 MHz) 52
Figure S9. ^{1}H-^{1}H COSY spectrum of compound **1** in $CDCl_3$ 53
Figure S10. HSQC spectrum of compound **1** in $CDCl_3$ 53
Figure S11. HMBC spectrum of compound **1** in $CDCl_3$ 54
Figure S12. NOESY spectrum of compound **1** in $CDCl_3$ 54
Figure S13. ^{1}H NMR spectrum of compound **2** in $CDCl_3$ (700 MHz) 55
Figure S14. ^{13}C NMR and DEPT spectra of compound **2** in $CDCl_3$ (150 MHz) 55
Figure S15. ^{1}H-^{1}H COSY spectrum of compound **2** in $CDCl_3$ 56
Figure S16. HSQC spectrum of compound **2** in $CDCl_3$ 56
Figure S17. HMBC spectrum of compound **2** in $CDCl_3$ 57
Figure S18. NOESY spectrum of compound **2** in $CDCl_3$ 57
Figure S19. ^{1}H NMR spectrum of compound **2a** in $CDCl_3$ (700 MHz) 58
Figure S20. ^{13}C NMR and DEPT spectra of compound **2a** in $CDCl_3$ (150 MHz) 58
Figure S21. ^{1}H-^{1}H COSY spectrum of compound **2a** in $CDCl_3$ 59
Figure S22. HSQC spectrum of compound **2a** in $CDCl_3$ 59
Figure S23. HMBC spectrum of compound **2a** in $CDCl_3$ 60
Figure S24. NOESY spectrum of compound **2a** in $CDCl_3$ 60
Figure S25. ^{1}H NMR spectrum of compound **3** in $CDCl_3$ (700 MHz) 61
Figure S26. ^{13}C NMR and DEPT spectra of compound **3** in $CDCl_3$ (150 MHz) 61
Figure S27. ^{1}H-^{1}H COSY spectrum of compound **3** in $CDCl_3$ 62
Figure S28. HSQC spectrum of compound **3** in $CDCl_3$ 62
Figure S29. HMBC spectrum of compound **3** in $CDCl_3$ 63
Figure S30. NOESY spectrum of compound **3** in $CDCl_3$ 63
Figure S31. ^{1}H NMR spectrum of compound **3a** in $CDCl_3$ (700 MHz) 64
Figure S32. ^{13}C NMR and DEPT spectra of compound **3a** in $CDCl_3$ (150 MHz) 64
Figure S33. ^{1}H-^{1}H COSY spectrum of compound **3a** in $CDCl_3$ 65
Figure S34. HSQC spectrum of compound **3a** in $CDCl_3$ 65
Figure S35. HMBC spectrum of compound **3a** in $CDCl_3$ 66
Figure S36. NOESY spectrum of compound **3a** in $CDCl_3$ 66
Figure S37. ^{1}H NMR spectrum of compound **4** in $CDCl_3$ (700 MHz) 67
Figure S38. ^{13}C NMR and DEPT spectra of compound **4** in $CDCl_3$ (150 MHz) 67
Figure S39. ^{1}H-^{1}H COSY spectrum of compound **4** in $CDCl_3$ 68
Figure S40. HSQC spectrum of compound **4** in $CDCl_3$ 68
Figure S41. HMBC spectrum of compound **4** in $CDCl_3$ 69
Figure S42. NOESY spectrum of compound **4** in $CDCl_3$ 69

Figure S43. ^{1}H NMR spectrum of compound **5** in $CDCl_3$ (700 MHz) 70
Figure S44. ^{13}C NMR and DEPT spectra of compound **5** in $CDCl_3$ (150 MHz) 70
Figure S45. ^{1}H-^{1}H COSY spectrum of compound **5** in $CDCl_3$ 71
Figure S46. HSQC spectrum of compound **5** in $CDCl_3$ 71
Figure S47. HMBC spectrum of compound **5** in $CDCl_3$ 72
Figure S48. NOESY spectrum of compound **5** in $CDCl_3$ 72
Figure S49. ^{1}H NMR spectrum of compound **6** in $CDCl_3$ (700 MHz) 73
Figure S50. ^{13}C NMR and DEPT spectra of compound **6** in $CDCl_3$ (150 MHz) 73
Figure S51. ^{1}H-^{1}H COSY spectrum of compound **6** in $CDCl_3$ 74
Figure S52. HSQC spectrum of compound **6** in $CDCl_3$ 74
Figure S53. HMBC spectrum of compound **6** in $CDCl_3$ 75
Figure S54. NOESY spectrum of compound **6** in $CDCl_3$ 75
Figure S55. ^{1}H NMR spectrum of compound **7** in $CDCl_3$ (700 MHz) 76
Figure S56. ^{13}C NMR and DEPT spectra of compound **7** in $CDCl_3$ (150 MHz) 76
Figure S57. ^{1}H-^{1}H COSY spectrum of compound **7** in $CDCl_3$ 77
Figure S58. HSQC spectrum of compound **7** in $CDCl_3$ 77
Figure S59. HMBC spectrum of compound **7** in $CDCl_3$ 78
Figure S60. NOESY spectrum of compound **7** in $CDCl_3$ 78
Figure S61. ^{1}H NMR spectrum of compound **8** in $CDCl_3$ (700 MHz) 79
Figure S62. ^{13}C NMR and DEPT spectra of compound **8** in $CDCl_3$ (150 MHz) 79
Figure S63. ^{1}H-^{1}H COSY spectrum of compound **8** in $CDCl_3$ 80
Figure S64. HSQC spectrum of compound **8** in $CDCl_3$ 80
Figure S65. HMBC spectrum of compound **8** in $CDCl_3$ 81
Figure S66. NOESY spectrum of compound **8** in $CDCl_3$ 81
Figure S67. ^{1}H NMR spectrum of compound **9** in $CDCl_3$ (700 MHz) 82
Figure S68. ^{13}C NMR and DEPT spectra of compound **9** in $CDCl_3$ (150 MHz) 82
Figure S69. ^{1}H-^{1}H COSY spectrum of compound **9** in $CDCl_3$ 83
Figure S70. HSQC spectrum of compound **9** in $CDCl_3$ 83
Figure S71. HMBC spectrum of compound **9** in $CDCl_3$ 84
Figure S72. NOESY spectrum of compound **9** in $CDCl_3$ 84
Figure S73. ^{1}H NMR spectrum of compound **10** in $CDCl_3$ (700 MHz) 85
Figure S74. ^{13}C NMR and DEPT spectra of compound **10** in $CDCl_3$ (150 MHz) 85
Figure S75. ^{1}H-^{1}H COSY spectrum of compound **10** in $CDCl_3$ 86
Figure S76. HSQC spectrum of compound **10** in $CDCl_3$ 86
Figure S77. HMBC spectrum of compound **10** in $CDCl_3$ 87
Figure S78. NOESY spectrum of compound **10** in $CDCl_3$ 87
Figure S79. ^{1}H NMR spectrum of compound **11** in $CDCl_3$ (700 MHz) 88
Figure S80. ^{13}C NMR and DEPT spectra of compound **11** in $CDCl_3$ (150 MHz) 88
Figure S81. ^{1}H-^{1}H COSY spectrum of compound **11** in $CDCl_3$ 89
Figure S82. HSQC spectrum of compound **11** in $CDCl_3$ 89
Figure S83. HMBC spectrum of compound **11** in $CDCl_3$ 90
Figure S84. NOESY spectrum of compound **11** in $CDCl_3$ 90
Figure S85. ^{1}H NMR spectrum of compound **12** in $CDCl_3$ (700 MHz) 91
Figure S86. ^{13}C NMR and DEPT spectra of compound **12** in $CDCl_3$ (150 MHz) 91
Figure S87. ^{1}H-^{1}H COSY spectrum of compound **12** in $CDCl_3$ 92
Figure S88. HSQC spectrum of compound **12** in $CDCl_3$ 92
Figure S89. HMBC spectrum of compound **12** in $CDCl_3$ 93
Figure S90. NOESY spectrum of compound **12** in $CDCl_3$ 93
Figure S91. ^{1}H NMR spectrum of compound **13** in $CDCl_3$ (700 MHz) 94

Figure S92. ^{13}C NMR and DEPT spectra of compound **13** in $CDCl_3$ (150 MHz) 94
Figure S93. ^{1}H-^{1}H COSY spectrum of compound **13** in $CDCl_3$ 95
Figure S94. HSQC spectrum of compound **13** in $CDCl_3$ 95
Figure S95. HMBC spectrum of compound **13** in $CDCl_3$ 96
Figure S96. NOESY spectrum of compound **13** in $CDCl_3$ 96
Figure S97. ^{1}H NMR spectrum of compound **14** in $CDCl_3$ (700 MHz) 97
Figure S98. ^{13}C NMR and DEPT spectra of compound **14** in $CDCl_3$ (150 MHz) 97
Figure S99. ^{1}H-^{1}H COSY spectrum of compound **14** in $CDCl_3$ 98
Figure S100. HSQC spectrum of compound **14** in $CDCl_3$ 98
Figure S101. HMBC spectrum of compound **14** in $CDCl_3$ 99
Figure S102. NOESY spectrum of compound **14** in $CDCl_3$ 99
Figure S103. ^{1}H NMR spectrum of compound **15** in $CDCl_3$ (700 MHz) 100
Figure S104. ^{13}C NMR and DEPT spectra of compound **15** in $CDCl_3$ (150 MHz) 100
Figure S105. ^{1}H-^{1}H COSY spectrum of compound **15** in $CDCl_3$ 101
Figure S106. HSQC spectrum of compound **15** in $CDCl_3$ 101
Figure S107. HMBC spectrum of compound **15** in $CDCl_3$ 102
Figure S108. NOESY spectrum of compound **15** in $CDCl_3$ 102
Figure S109. ^{1}H NMR spectrum of compound **16** in $CDCl_3$ (700 MHz) 103
Figure S110. ^{13}C NMR and DEPT spectra of compound **16** in $CDCl_3$ (150 MHz) 103
Figure S111. ^{1}H-^{1}H COSY spectrum of compound **16** in $CDCl_3$ 104
Figure S112. HSQC spectrum of compound **16** in $CDCl_3$ 104
Figure S113. HMBC spectrum of compound **16** in $CDCl_3$ 105
Figure S114. NOESY spectrum of compound **16** in $CDCl_3$ 105
Figure S115. ^{1}H NMR spectrum of compound **17** in $CDCl_3$ (700 MHz) 106
Figure S116. ^{13}C NMR and DEPT spectra of compound **17** in $CDCl_3$ (150 MHz) 106
Figure S117. ^{1}H-^{1}H COSY spectrum of compound **17** in $CDCl_3$ 107
Figure S118. HSQC spectrum of compound **17** in $CDCl_3$ 107
Figure S119. HMBC spectrum of compound **17** in $CDCl_3$ 108
Figure S120. NOESY spectrum of compound **17** in $CDCl_3$ 108
Figure S121. ^{1}H NMR spectrum of compound **18** in $CDCl_3$ (700 MHz) 109
Figure S122. ^{13}C NMR and DEPT spectra of compound **18** in $CDCl_3$ (150 MHz) 109
Figure S123. ^{1}H-^{1}H COSY spectrum of compound **18** in $CDCl_3$ 110
Figure S124. HSQC spectrum of compound **18** in $CDCl_3$ 110
Figure S125. HMBC spectrum of compound **18** in $CDCl_3$ 111
Figure S126. NOESY spectrum of compound **18** in $CDCl_3$ 111
Figure S127. ^{1}H NMR spectrum of compound **19** in $CDCl_3$ (700 MHz) 112
Figure S128. ^{13}C NMR and DEPT spectra of compound **19** in $CDCl_3$ (150 MHz) 112
Figure S129. ^{1}H-^{1}H COSY spectrum of compound **19** in $CDCl_3$ 113
Figure S130. HSQC spectrum of compound **19** in $CDCl_3$ 113
Figure S131. HMBC spectrum of compound **19** in $CDCl_3$ 114
Figure S132. NOESY spectrum of compound **19** in $CDCl_3$ 114
Figure S133. ^{1}H NMR spectrum of compound **20** in $CDCl_3$ (700 MHz) 115
Figure S134. ^{13}C NMR and DEPT spectra of compound **20** in $CDCl_3$ (150 MHz) 115

Experimental Procedure

1. General procedures

GC-MS analyses were performed using an Agilent GC (8890)-MSD (7000D) instrument with a HP-5MS quartz capillary column (30 m × 250 μm i.d., 0.25 μm film thickness). High purity helium was used as carrier gas. For mass spectral detector, ion source, transfer-line and quadruple temperatures were set at 230 °C, 250 °C and 150 °C, respectively, with electronic ionization (EI) mode at 70 eV and a scan range of *m*/*z* 40-450. Column chromatography (CC) was carried out on the silica gel or silver nitrate impregnated silica gel (200–300 mesh, Qingdao Marine Chemical Factory). Semi-preparative HPLC separations were performed on an Agilent 1260 series instrument with a ChromCore Phenyl column (5 μm, 10 × 250 mm, 3 mL/min). Analytical thin-layer chromatography (TLC) was performed on silica gel plates (GF_{254}, 10–40 μm, Qingdao Marine Chemical Factory). Spots on TLC were visualized by heating after spraying with 5% H_2SO_4 in EtOH (v/v). NMR experiments were carried out on a Bruker AV-600 or AV-700 spectrometer with TMS as the internal standard. ECD spectra were conducted with a Chirascan qCD spectrometer. Optical rotations were obtained on a Jasco P-1020 spectropolarimeter. X-ray crystallographic analysis was performed with a Bruker D8 Quest instrument (Bruker, Karlsruher, Germany) using Cu K*α* radiation. *n*-Hexane, cyclohexane, petroleum ether (PE) and EtOAc used for extraction and isolation were analytical grade and distilled before use. Chemicals and solvents used for chemical synthesis were purchased from Sigma-Aldrich.

2. Plant materials, plasmids, strains and culture conditions

Seeds of *Capsella bursa-pastoris* (L.) Medik. were requested from National Wild Plant Germplasm Resource Center. After grown in a constant greenhouse at 22 °C with a 16-h-light/8-h-dark cycle for 3–4 weeks, the whole plants of *C. bursa-pastoris* were used for cloning sesterterpene synthases (StTSs). Plasmid pBbA5c-MevT-MBIS containing the complete gene set of MVA pathway and a FDPS gene was kindly provided by Prof. Tao Liu at Tianjin Institute of Industrial Biotechnology, Chinese Academy of Sciences. Plasmid RSPT was constructed by successively integrating idi (IDP isomerase), AaTPS1-PT (PT domain of AaTPS1), and DXR/DXS (rate-limiting enzymes of *Escherichia coli* MEP pathway) under the inducible expression of T7 promoter in pCDF-duet1 vector. *E. coli* DH5α and C41(DE3) (Transgen Biotech) were used for plasmid propagation and gene heterologous expression, respectively. Vector pMAL-c2x was purchased from Takara Bio companies. The culture media for *E. coli* strains were Luria-Bertani (LB) medium (10 g/L tryptone, 5 g/L yeast extract, 10 g/L NaCl) or Terrific Broth (TB) medium (12 g/L tryptone, 24 g/L yeast extract, 4 mL/L glycerol, 2.31 g/L KH_2PO_4, 12.54 g/L K_2HPO_4).

3. Gene cloning and phylogenetic analysis

Candidate StTSs were searched from the genome and transcriptome of *C. bursa-pastoris* using the local BLAST program with the amino acid sequences of known plant StTSs (Table S1). Total RNA was prepared from *C. bursa-pastoris* using the Total RNA Extractor (Trizol). The complementary DNA (cDNA) was reverse-transcribed from total RNA by the HiScript II 1st Strand cDNA Synthesis Kit (Vazyme). The full-length cDNA of *CbTPS1* was amplified with gene-specific primers (Table S2) by high-fidelity PrimeSTAR Max DNA Polymerase (Takara). The resultant fragment of *CbTPS1* was subcloned into EcoRI and PstI sites of expression vector pMAL-c2x digested with corresponding restriction endonucleases (Takara), which was then transferred into *E. coli* DH5α by the heat shock method. The colonies were checked by colony polymerase chain reaction (PCR), and the plasmids named pMAL-*CbTPS1* were extracted from the positive colonies and confirmed by DNA sequencing. Amino acid sequences of the known TPSs were obtained from the NCBI database (Table S1) and aligned using Clustal W. The maximum-likelihood phylogenetic tree was generated based on this alignment using Mega11 with 1000 bootstrap resampling.

4. Enzyme activity assay *in vivo* using engineered *E. coli*

The recombinant plasmids pMAL-*CbTPS1* was co-transferred into the *E. coli* expression strain C41(DE3) harboring pBbA5c-MevT-MBIS and RSPT vectors as described above to generate engineered *E. coli* EC-CbTPS1. An *E. coli* strain containing empty vector pMAL-c2x, pBbA5c-MevT-MBIS, and RSPT was used as a negative control. The colonies were selected on the LB agarose plates containing 100 μg/mL ampicillin, 34 μg/mL chloramphenicol, and 50 μg/mL streptomycin, and then confirmed by colony-PCR. The confirmed transformants were inoculated in 50 mL TB liquid medium containing the corresponding antibiotics at 37 °C, 180 rpm/min to an OD_{600} of 0.6–0.7, and then induced by 0.5 mM IPTG at 18 °C for 24 h, followed by the supplement of 20 mM sodium pyruvate and a continuing cultivation at 25 °C for 72 h. Subsequently, the culture media were ultrasonically extracted with EtOAc (100 mL × 3, 15 min per time) and centrifuged (3900 rpm, 10 min). The obtained EtOAc extraction was condensed to dryness, and then redissolved in *n*-hexane to 500 μL. 1 μL of each sample was injected in splitless mode for GC-MS analysis. The temperature program of GC-MS analysis was set as follows: initial temperature 60 °C, holding for 2 min; ramp at rate of 35 °C/min to 180 °C, holding for 2 min; ramp at rate of 4 °C/min to 300 °C, holding for 1 min; ramp at rate of 35 °C/min to 310 °C, holding for 3 min. The products of engineered *E. coli* EC-CbTPS1 were searched by comparison of their retention time and mass spectra with those of the negative control.

5. Molecular dynamics simulation and site-directed mutagenesis

The protein structures of CbTPS1 and CbTPS1^{L354M} were modeled using Alphafold2.[1] To reconstruct the Mg^{2+} coordination shell, encompassing three Mg^{2+} ions and the PPi group, we drew upon the well-established coordination mode found in class I terpene synthases (PDB code: 4KUX).[2] The intermediate was then docked into both CbTPS1 and CbTPS1^{L354M} using AutoDock Vina. A grid box of 25 × 25 × 25 Å^3 was defined to encompass the substrate-binding site, and the exhaustiveness was set to 64. Default settings were used for all other parameters. Potential poses were identified based on the docking results. The protein model was generated using the Amber ff99SB force field, with solvent water molecules represented by the TIP3P model.[3] Ligand force field parameters were derived from the general AMBER force field (GAFF), and partial atomic charges were calculated using the restrained electrostatic potential (RESP) method based on HF/6-31G* calculations performed with Gaussian 09. Initial coordinates and topology files were prepared using the tleap module in AMBER20. Classical molecular dynamics (MD) simulations were conducted exclusively with AMBER20, employing periodic boundary conditions with cubic models. The system preparation involved a series of minimization steps to relax the solvent and the protein-ligand complex, beginning with the solute atoms constrained, followed by the protein backbone, and ending with the removal of all constraints. The systems were then gradually heated from 0 to 300 K in the NVT ensemble over 100 ps, followed by 100 ps of MD simulations in the NPT ensemble at 300 K and 1.0 atm. After equilibration, 50 ns of production MD simulations were performed in the NVT ensemble at 300 K, using the GPU-accelerated pmemd program in AMBER20. A time step of 1 fs was used throughout the MD simulations, with the SHAKE algorithm applied to constrain the high-frequency vibrations of bonds involving hydrogen atoms. A 10 Å cutoff was applied to van der Waals interactions, while electrostatic interactions were calculated without a cutoff. Protein-ligand interaction images were generated using PyMOL. Data are presented as mean ± SD, based on three independent experiments.

Site-directed mutagenesis was performed by PCR amplification using the pMAL-*CbTPS1* as template and the mutated complementary sequences as primers (Table S2). PCR amplicons were purified using a DNA quick purification kit and individually sub-cloned into pMAL-c2x vector using ClonExpress II One Step Cloning Kit (Vazyme). Each variant was confirmed by DNA sequencing, and then transformed into an *E. coli* expression train C41(DE3) harboring pBbA5c-MevT-MBIS and RSPT vectors. The products of all the variants were extracted and analyzed as described above.

6. Isolation of sesterterpenes by large-scale fermentation of engineered *E. coli*

For the functional identification of CbTPS1, the positive transformant was grown in 15 L TB medium using the aforementioned culture condition. The culture media was extracted with EtOAc (15 L × 3), and then the

EtOAc extraction was evaporated under reduced pressure. The obtained oil residue was dissolved in PE and subjected to silica gel CC with PE as the elution to afford three fractions (Fr. 1–Fr. 3). Fr. 1 was separated over repeated silica gel (impregnated with silver nitrate) CC eluted by *n*-hexane to yield compounds **1** (106.3 mg) and **2** (122.0 mg). Fr. 2 was purified by repeated silica gel (impregnated with silver nitrate) CC eluted by cyclohexane to yield compound **3** (135.8 mg).

For the functional identification of CbTPS1^{L354M}, the positive transformant was cultured in 90 L TB medium under the same condition. The culture media was extracted with EtOAc (90 L × 3), and then the EtOAc extraction was evaporated under reduced pressure. The obtained oil residue was dissolved in PE and subjected to silica gel CC with a stepwise-gradient elution of PE-EtOAc (100:0, 50:1, 10:1, and 0:1, v/v) to afford seven fractions (Fr. 1–Fr. 7). Fr. 1 was chromatographed on repeated silica gel (impregnated with silver nitrate) CC eluted by *n*-hexane to yield compounds **8** (22.0 mg) and **11** (13.7 mg). Fr. 2 was subjected to repeated silica gel (impregnated with silver nitrate) CC eluted with *n*-hexane-EtOAc (100:0 and 10:1, v/v) to yield compound **14** (1.6 mg). Fr. 3 was separated by repeated silica gel (impregnated with silver nitrate) CC with *n*-hexane as the eluent to afford compound **5** (24.1 mg), and two subfractions (Fr. 3-1 and Fr. 3-2). Fr. 3-1 was applied to semi-preparative HPLC using MeOH-H_2O (95:5, v/v) as the mobile phase to yield compounds **7** (t_R 16.2 min, 18.3 mg) and **4** (t_R 16.4 min, 19.0 mg). Fr. 3-2 was applied to semi-preparative HPLC using MeOH-H_2O (90:10, v/v) as the mobile phase to yield compounds **19** (t_R 18.4 min, 8.2 mg) and **17** (t_R 19.8 min, 5.6 mg). Fr. 4 was separated by repeated silica gel (impregnated with silver nitrate) CC with *n*-hexane as the eluent to afford compound **12** (3.8 mg), and four subfractions (Fr. 4-1–Fr. 4-3). Fr. 4-1 was further applied to semi-preparative HPLC using MeOH-H_2O (90:10, v/v) as the mobile phase to yield compound **20** (t_R 22.6 min, 2.0 mg). Fr. 4-3 was applied to semi-preparative HPLC using MeOH-H_2O (100:0, v/v) as the mobile phase to yield compound **15** (t_R 32.0 min, 1.8 mg). Fr. 5 was subjected to repeated silica gel (impregnated with silver nitrate) CC eluted with *n*-hexane or cyclohexane to yield compounds **6** (1.5 mg), **13** (8.5 mg), **16** (13.3 mg), and **18** (20.2 mg). Fr. 6 and Fr. 7 was separately subjected to silica gel (impregnated with silver nitrate) CC eluted with *n*-hexane-EtOAc (100:0 and 10:1, v/v) to yield compounds **9** (136.0 mg) and **10** (8.0 mg), respectively.

1: colorless oil, $[\alpha]_D^{20}$ +13.6 (*c* 0.04, EtOH); $[M]^+$ = 340 (EI-MS); ^1H and ^{13}C NMR data, see Table S3.

2: colorless oil, $[\alpha]_D^{20}$ +17.1 (*c* 0.03, EtOH); $[M]^+$ = 340 (EI-MS); ^1H and ^{13}C NMR data, see Table S4.

3: colorless oil, $[\alpha]_D^{20}$ +19.0 (*c* 0.04, EtOH); $[M]^+$ = 340 (EI-MS); ^1H and ^{13}C NMR data, see Table S6.

4: colorless oil, $[\alpha]_D^{20}$ +9.1 (*c* 0.03, EtOH); $[M]^+$ = 340 (EI-MS); ^1H and ^{13}C NMR data, see Table S8.

5: colorless oil, $[\alpha]_D^{20}$ +50.0 (*c* 0.02, EtOH); $[M]^+$ = 340 (EI-MS); ^1H and ^{13}C NMR data, see Table S9.

6: colorless oil, $[\alpha]_D^{20}$ –550 (*c* 0.01, EtOH); $[M]^+$ = 340 (EI-MS); ^1H and ^{13}C NMR data, see Table S10.

7: colorless oil, $[\alpha]_D^{20}$ +11.7 (*c* 0.03, EtOH); $[M]^+$ = 340 (EI-MS); ^1H and ^{13}C NMR data, see Table S11.

8: colorless oil, $[\alpha]_D^{20}$ +11.1 (*c* 0.02, EtOH); $[M]^+$ = 340 (EI-MS); ^{1}H and ^{13}C NMR data, see Table S12.

9: colorless crystal, $[\alpha]_D^{20}$ –197 (*c* 0.03, EtOH); ^{1}H and ^{13}C NMR data, see Table S13; Crystallographic data: $C_{25}H_{41}O$, *M-H* = 357.58, *a* = 8.0125(13) Å, *b* = 13.987(2) Å, *c* = 9.9775(15) Å, α = 90°, β = 95.631(9)°, γ = 90°, *V* = 1112.8(3) Å^3, *T* = 293(2) K, space group *P*1211, *Z* = 2, μ(Cu Kα) = 0.463 mm^{-1}, 16683 reflections measured, 4010 independent reflections (R_{int} = 0.0653). The final R_1 values were 0.0586 ($I > 2\sigma(I)$). The final $wR(F^2)$ values were 0.1385 ($I > 2\sigma(I)$). The final R_1 values were 0.0867 (all data). The final $wR(F^2)$ values were 0.1683 (all data). The goodness of fit on F^2 was 1.060. Flack parameter = –0.2(3). The crystallographic data have been deposited at the Cambridge Crystallographic Data Centre (CCDC 2378877).

10: colorless oil, $[\alpha]_D^{20}$ –110 (*c* 0.05, EtOH); $[M]^+$ = 358 (EI-MS); ^{1}H and ^{13}C NMR data, see Table S14.

11: colorless oil, $[\alpha]_D^{20}$ +302 (*c* 0.04, EtOH); $[M]^+$ = 340 (EI-MS); ^{1}H and ^{13}C NMR data, see Table S15.

12: colorless oil, $[\alpha]_D^{20}$ +21.7 (*c* 0.02, EtOH); $[M]^+$ = 340 (EI-MS); ^{1}H and ^{13}C NMR data, see Table S16.

13: colorless oil, $[\alpha]_D^{20}$ +23.8 (*c* 0.02, EtOH); $[M]^+$ = 340 (EI-MS); ^{1}H and ^{13}C NMR data, see Table S17.

14: colorless oil, $[\alpha]_D^{20}$ –285 (*c* 0.02, EtOH); $[M]^+$ = 356 (EI-MS); ^{1}H and ^{13}C NMR data, see Table S18.

15: colorless oil, $[\alpha]_D^{20}$ –38.3 (*c* 0.03, EtOH); $[M]^+$ = 340 (EI-MS); ^{1}H and ^{13}C NMR data, see Table S20.

16: colorless oil, $[\alpha]_D^{20}$ +7.3 (*c* 0.04, EtOH); $[M]^+$ = 340 (EI-MS); ^{1}H and ^{13}C NMR data, see Table S21.

17: colorless oil, $[\alpha]_D^{20}$ –416 (*c* 0.02, EtOH); $[M]^+$ = 340 (EI-MS); ^{1}H and ^{13}C NMR data, see Table S22.

20: colorless oil, $[\alpha]_D^{20}$ –218 (*c* 0.03, EtOH); $[M]^+$ = 340 (EI-MS); ^{1}H and ^{13}C NMR data, see Table S24.

7. Chemical synthesis of sesterterpene derivatives for X-ray diffraction analysis

*Synthesis of (+)-epoxy-capbuene B (**2a**) by epoxidation of (+)-capbuene B (**2**) with m-CPBA.* (+)-Capbuene B (MW = 340, 38 mg, 112 μmol) was dissolved in dichloromethane (2 mL) in a vial which was chilled with ice-water afterwards. To the solution was then added *m*-CPBA (MW = 172.6, 82 mg, 475 μmol) in one lot. The resulting solution was stirred for 30 min before quenching with saturated $NaSO_3$ solution (1 mL). Reaction products were extracted with Et_2O (3 × 2 mL). Combined ether extracts were dried under N_2 and purified by silica gel CC using gradient *n*-hexane/EtOAc (100:0 to 10:1, v/v) as eluents to yield (+)-epoxy-capbuene B (14 mg, 36%, white solid). (+)-Epoxy-capbuene B (**2a**) was crystallized by slow evaporation from *n*-hexane-Et_2O (5:1, v/v) to form suitable crystals for X-ray diffraction analysis.

2a: colorless crystal, $[\alpha]_D^{20}$ +150 (*c* 0.05, EtOH); ^{1}H and ^{13}C NMR data, see Table S5; Crystallographic data: $C_{25}H_{40}O$, *M* = 356.57, *a* = 7.7009(15) Å, *b* = 13.311(3) Å, *c* = 11.029(2) Å, α = 90°, β = 106.339(11)°, γ = 90°, *V* = 1084.9(4) Å^3, *T* = 293(2) K, space group *P*1211, *Z* = 2, μ(Cu Kα) = 0.475 mm^{-1}, 13866 reflections measured, 3931 independent reflections (R_{int} = 0.0661). The final R_1 values were 0.0481 ($I > 2\sigma(I)$). The final $wR(F^2)$ values were 0.1109 ($I > 2\sigma(I)$). The final R_1 values were 0.0666 (all data). The final $wR(F^2)$ values were 0.1271 (all data). The goodness of fit on F^2 was 1.082. Flack parameter = –0.1(2). The

crystallographic data have been deposited at the Cambridge Crystallographic Data Centre (CCDC 2378876).

*Synthesis of (–)-epoxy-capbuene C (**3a**) by epoxidation of (+)-capbuene C (**3**) with m-CPBA.* (+)-Capbuene C (MW = 340, 18 mg, 53 μmol) was dissolved in dichloromethane (2 mL) in a vial which was chilled with ice-water afterwards. To the solution was then added *m*-CPBA (MW = 172.6, 39 mg, 226 μmol) in one lot. The resulting solution was stirred for 30 min before quenching with saturated $NaSO_3$ solution (1 mL). Reaction products were extracted with Et_2O (3 × 2 mL). Combined ether extracts were dried under N_2 and purified by silica gel CC using gradient *n*-hexane/EtOAc (100:0 to 10:1, v/v) as eluents to yield (–)-epoxy-capbuene C (6.2 mg, 33%, white solid). (–)-Epoxy-capbuene C (**3a**) was crystallized by slow evaporation from *n*-hexane-Et_2O (5:1, v/v) to form suitable crystals for X-ray diffraction analysis.

3a: colorless crystal, $[\alpha]_D^{20}$ –148 (*c* 0.02, EtOH); ^{1}H and ^{13}C NMR data, see Table S7; Crystallographic data: $C_{25}H_{40}O$, $M = 356.57$, $a = 9.6681(3)$ Å, $b = 7.1687(2)$ Å, $c = 15.8911(5)$ Å, $\alpha = 90°$, $\beta = 90.9250(10)°$, $\gamma = 90°$, $V = 1084.89(6)$ Å^3, $T = 293(2)$ K, space group $P1211$, $Z = 2$, μ(Cu Kα) = 0.475 mm^{-1}, 21801reflections measured, 3923 independent reflections ($R_{int} = 0.0631$). The final R_1 values were 0.0420 ($I > 2\sigma(I)$). The final $wR(F^2)$ values were 0.1026 ($I > 2\sigma(I)$). The final R_1 values were 0.0510 (all data). The final $wR(F^2)$ values were 0.1106 (all data). The goodness of fit on F^2 was 1.072. Flack parameter = 0.09(19). The crystallographic data have been deposited at the Cambridge Crystallographic Data Centre (CCDC 2378878).

8. Quantum chemical calculations and configuration determination of sesterterpenes

Conformational analyses of the structures were carried out via Monte Carlo searching using molecular mechanism with MMFF force field in the Spartan 18 program. The force field minimum energy conformers thus obtained were subsequently optimized by applying the density functional theory (DFT) with the B3LYP/6-31+G(d,p) level in vacuum in Gaussian 09 software. Harmonic vibrational frequencies were also performed to confirm no imaginary frequencies of the finally optimized conformers. All the conformers were used for calculated electronic circular dichroism (ECD) by DFT at the CAM-B3LYP/DGDZVP level in ethanol with the polarizable continuum model (PCM) in Gaussian 09 software. The overall calculated ECD curves were generated by Boltzmann weighting of their selected low-energy conformers with σ = ~0.3 eV using SpecDis 1.62 software. Gauge independent atomic orbital (GIAO) calculations of NMR chemical shifts were performed by DFT at the MPW1PW91/6-311+G(d,p) level in chloroform with PCM in Gaussian 09 software. NMR chemical shifts of TMS were calculated in the same level and used as the references. The experimental and calculated NMR data of isomeric compounds were analyzed by the DP4+ method.[4]

The cyclization mechanism from **h** to **j** was studied using DFT calculations with Gaussian 09 software. Geometry optimizations and vibrational frequency analyses of all carbocation intermediates and transition states involved in the mechanism were conducted in the gas phase with the B3LYP/6-31G(d) method.

Additionally, intrinsic reaction coordinate (IRC) calculations were performed. Single-point energies were obtained using the M06-2X/6-311+G(d,p) method, and the Gibbs free energies discussed were derived by applying gas-phase Gibbs free energy corrections.[5]

In general, the configurations of the new sesterterpenes (**1**–**17**) were determined based on the NOESY experiments, the X-ray diffraction analysis of compounds **2a**, **3a**, and **9**, and a viewpoint of biosynthetic paths (Figure 3). In particular, the absolute configurations of compounds **1**–**17** were further confirmed by quantum chemical calculations. For (–)-capbunin B (**10**), the calculated ECD curve of 12*R*-**10** instead of that of 12*S*-**10** matched well with the experimental curve (Table S14), indicating the β-configuration of 12-OH. For (+)-capbudiene A (**11**), the calculated ECD curve matched well with the experimental curve of **11** (Table S15), further confirming the configuration determination. For (+)-capbutriene A (**12**), the good consistency between the calculated ECD curve of 10*S*-**12** and the experimental one determined the 10*S* configuration (Table S16). For (+)-capbutriene B (**13**), the calculated ECD curve matched well with the experimental one of **13** (Table S17). For (–)-capbunin C (**14**), the ECD calculations (Table S18) suggested the (10*R*,11*R*) or (10*S*,11*R*) configuration of **14**, while the further DP4+ analysis of ^{1}H and ^{13}C NMR calculations (Table S19) indicated an overwhelming superiority of the (10*R*,11*R*) configuration (with final score of 100.00%) against the (10*S*,11*R*) configuration. For (–)-capbupentaene A (**17**), the results of ECD calculations (Table S22) supported the 15*R*-configuration of **17**. For (–)-cericerne (**20**), the ECD calculations were performed to further confirmed the 14*R*-configuration of **20** (Table S24).

9. Quantitative analysis of metabolites in engineered *E. coli* EC-CbTPS1 and its variants

Five concentration gradients of compound **1** (400, 200, 100, 50, and 25 mg/L) were prepared for GC-MS using the aforementioned method. Each concentration was injected for three replicates, and the linearity of standard curve was made by plotting the peak area versus concentration. The equation and correlation coefficient obtained from the linearity study was $y = 3\times 10^6 x - 329167$ ($R^2 = 0.9991$). The engineered *E. coli* EC-CbTPS1 and its variant EC-CbTPS1^{L354M} were cultured and analyzed by the method as described above. The yields of sesterterpenes produced by CbTPS1 and its variants were calculated by the peak areas and the calibration curve (Table S32). Each experiment was repeated with three independent biological replicates.

10. Nematodes cultivation and RNA-seq analysis

The *Caenorhabditis elegans* strains (N_2 worms) were maintained on standard nematode growth medium (NGM) agar plates seeded with *Escherichia coli* OP50 at 20 °C in an incubator. The synchronized L1 worms were cultured in NGM agar plates supplemented with compound **9** (200 μM) or DMSO for five days. Total

RNA was extracted from around 3000 worms treated with 100 μg/mL PEO or DMSO using RNAeasy™ Animal RNA Isolation Kit (Beyotime, China), and quantified by Qubit3.0 with QubitTM RNA Broad Range Assay kit (Life Technologies, Q10210). A total of 2 μg RNA per sample was used to generate cDNA libraries and sequence. The sequence was performed on a DNBSEQ-T7 sequencer with PE150 model. The de-duplicated consensus sequences were used for standard RNA-seq analysis, and they were aligned to the *C. elegans* reference genome from WormBase (https://parasite.wormbase.org/ftp.html) using STAR software with default parameters. The gene expression levels were quantified by the Reads per Kilobase per Million Reads (RPKM). Genes with |log FC| > 2 and p-value < 0.05 were considered as differentially expressed genes (DEGs). The experiments were performed with three independent biological replicates. Gene ontology (GO) analysis and Kyoto encyclopedia of genes and genomes (KEGG) enrichment analysis for differentially expressed genes were implemented using KOBAS software and Metascape (https://www.metascape.org) with a p-value cutoff of 0.05 to determine statistically significant enrichment.

11. *In vitro* anti-liver fibrosis activity and western blot assay

LX-2 cells were cultured in DMEM supplemented with 10% FBS and 1% penicillin-streptomycin. The cytotoxicity of compounds was evaluated using the CellTiter 96® AQueous One Solution Cell Proliferation Assay (MTS assay). LX-2 cells (1×10^4/well) were plated in a 96-well plate and treated with compounds at indicated concentrations for 48h. Cells were incubated with MTS for 1h and detected at 490 nm by a microplate reader. For western blot assay, the cells were lysed in Western/IP Cell Lysis Buffer with proteinase and phosphatase inhibitors (APE×BIO) and incubated on ice for 15 min and then centrifuged at 4°C, 12000 rpm for 10 min. The supernatant was obtained and the protein concentration was also measured by the BCA method. The protein was added with loading buffer and boiled for 8 min. Proteins (30 μg per sample) were separated by SDS-PAGE and transferred to PVDF membranes. After blocking in 5% non-fat milk for 1 h at room temperature, the membranes were incubated with the corresponding primary antibodies overnight at 4 °C. Then, the membranes were washed and incubated with the secondary antibody for 1 h at room temperature. Finally, the protein bands were detected by an enhanced chemiluminescence reagent (ECL) kit. For ELISA assay of COL1A1, cells (3×10^5/well) were seeded into 6-well plates and treated with compounds and TGF-β1 for 24 h. Then, cells were lysed in NP-40 with proteinase inhibitor (APE×BIO) and then centrifuged (12000 rpm, 10 min, 4°C). The concentration of total protein was determined by a BCA kit. COL1A1 content in lysates was measured using the Human Pro-Collagen Iα1/COL1A1 ELISA Kit.

12. Statistical analysis

Data were presented as the mean ± SD from three biological replicates. Statistical analysis was carried out

using one-way analysis of variance (ANOVA). Prism 9.0.0 (GraphPad Software) was used for data analysis and graph plotting.

References

[1] K. Tunyasuvunakool, J. Adler, Z. Wu, T. Green, M. Zielinski, A. Žídek, A. Bridgland, A. Cowie, C. Meyer, A. Laydon, S. Velankar, G. J. Kleywegt, A. Bateman, R. Evans, A. Pritzel, M. Figurnov, O. Ronneberger, R. Bates, S. A. A. Kohl, A. Potapenko, A. J. Ballard, B. Romera-Paredes, S. Nikolov, R. Jain, E. Clancy, D. Reiman, S. Petersen, A. W. Senior, K. Kavukcuoglu, E. Birney, P. Kohli, J. Jumper, D. Hassabis, *Nature* **2021**, *596*, 590–596.

[2] G. M. Morris, R. Huey, W. Lindstrom, M. F. Sanner, R. K. Belew, D. S. Goodsell, A. J. Olson, *J. Comput. Chem.* **2009**, *30*, 2785–2791.

[3] Y. Duan, C. Wu, S. Chowdhury, M. C. Lee, G. Xiong, W. Zhang, R. Yang, P. Cieplak, R. Luo, T. Lee, J. Caldwell, J. Wang, P. Kollman, *J. Comput. Chem.* **2003**, *24*, 1999–2012.

[4] N. Grimblat, M. M. Zanardi, A. M. Sarotti, *J. Org. Chem.* **2015**, *80*, 12526–12534.

[5] W. Zha, F. Zhang, J. Shao, X. Ma, J. Zhu, P. Sun, R. Wu, J. Zi, *Nat. Commun.* **2022**, *13*, 2508.

Supplementary Tables

Table S1. Plant TPSs used for the phylogenetic analysis

STSs	Accession number	Species	Source
CbTPS1	PQ213475	*Capsella bursa-pastoris*	NCBI
AtTPS25	AT3g29410	*Arabidopsis thaliana*	NCBI
Bo250	LOC106343250	*Brassica oleracea*	NCBI
AtTPS22	AT1g33750	*Arabidopsis thaliana*	NCBI
AtTPS30	AT3g32030	*Arabidopsis thaliana*	NCBI
Cr089	AT3g14490	*Capsella rubella*	NCBI
AtTPS17	CARUB_v10016089mg	*Arabidopsis thaliana*	NCBI
Br580	LOC103859580	*Brassica rapa*	NCBI
Cr237	CARUB_v10016237mg	*Capsella rubella*	NCBI
AtTPS29	AT1g31950	*Arabidopsis thaliana*	NCBI
AtTPS18	AT3g14520	*Arabidopsis thaliana*	NCBI
AtTPS19	AT3g14540	*Arabidopsis thaliana*	NCBI
AtTPS06	AT1g70080	*Arabidopsis thaliana*	NCBI
CcTPS1	MZ686957	*Colquhounia coccinea* var. *mollis*	NCBI
LcTPS2	MZ147599	*Leucosceptrum canum*	NCBI
LcCedS	QBP05430	*Leucosceptrum canum*	NCBI
SmSTPS1	A0A1W6GW32	*Salvia miltiorrhiza*	NCBI
SmSCPS1	AHJ59321	*Salvia miltiorrhiza*	NCBI
SmSCPS2	AHJ59322	*Salvia miltiorrhiza*	NCBI
SmSCPS3	AHJ59323	*Salvia miltiorrhiza*	NCBI
SmSCPS4	AKN91186	*Salvia miltiorrhiza*	NCBI
SmSCPS5	AHJ59324	*Salvia miltiorrhiza*	NCBI
PvHVS	AZB50511	*Prunella vulgaris*	NCBI
PvTPS2	AZB50510	*Prunella vulgaris*	NCBI
PvTPS4	AZB50512	*Prunella vulgaris*	NCBI
PvTPS5	AZB50513	*Prunella vulgaris*	NCBI
AtTPS2	NP_193406	*Arabidopsis thaliana*	NCBI
AtTPS3	NP_567511	*Arabidopsis thaliana*	NCBI
AtTPS8	NP_193754	*Arabidopsis thaliana*	NCBI
AtTPS10	NP_179998	*Arabidopsis thaliana*	NCBI
AtTPS11	NP_199276	*Arabidopsis thaliana*	NCBI
AtTPS13	NP_193066	*Arabidopsis thaliana*	NCBI
AtTPS14	NP_176361	*Arabidopsis thaliana*	NCBI
AtTPS23	NP_189210	*Arabidopsis thaliana*	NCBI
AtTPS24	NP_189209	*Arabidopsis thaliana*	NCBI
AtTPS31	NP_192187	*Arabidopsis thaliana*	NCBI
AmLinS	EF433761	*Antirrhinum majus*	NCBI
AmMyrS	AY195608	*Antirrhinum majus*	NCBI
SlTPS3	JN408284	*Solanum lycopersicum*	NCBI
SlTPS7	JN408287	*Solanum lycopersicum*	NCBI
SlTPS8	JN408288	*Solanum lycopersicum*	NCBI

SlTPS9	JN408289	*Solanum lycopersicum*	NCBI
SlTPS14	JN412091	*Solanum lycopersicum*	NCBI
SlTPS27	JN412084	*Solanum lycopersicum*	NCBI
ObZinS	AAV63788.1	*Ocimum basilicum*	NCBI
ApLinS	ADD81295	*Actinidia polygama*	NCBI
VvTerS	AAS79351.1	*Vitis vinifera*	NCBI
VvLinS	ADR74209.1	*Vitis vinifera*	NCBI
VvPinS	ADR74202.1	*Vitis vinifera*	NCBI
SfCinS1	ABH07677.1	*Salvia fruticosa*	NCBI
MdFarS	AY787633	*Malus domestica*	NCBI
MlOciS	KF857262.1	*Mimulus lewisii*	NCBI
PtTPS4	AEI52904	*Populus trichocarpa*	NCBI
NsCBTS2a	NP_001289541	*Nicotiana sylvestris*	NCBI
TEAS	5EAS_A	*Nicotiana tabacum*	NCBI
EpCaS	AGN70884	*Euphorbia peplus*	NCBI
EpCPS	AGN70883	*Euphorbia peplus*	NCBI
TsCaS	ADB90272	*Triadica sebifera*	NCBI
RcCaS1	XP_002513340	*Ricinus communis*	NCBI
OsLinS	Os02g02930	*Oryza sativa*	NCBI
OsCPS1	Os02g17780	*Oryza sativa*	NCBI
OsKS1	Os04g52230	*Oryza sativa*	NCBI
OsKSL4	Os04g10060	*Oryza sativa*	NCBI
OsKSL5	Os02g36220	*Oryza sativa*	NCBI
OsKSL6	Os02g36264	*Oryza sativa*	NCBI
OsDTC1	Os02g36140	*Oryza sativa*	NCBI
OsKSL10	Os12g30824	*Oryza sativa*	NCBI
AgPheS	AAF61453	*Abies grandis*	NCBI
AgLPS	AAF61455	*Abies grandis*	NCBI
AgTerS	AAF61454	*Abies grandis*	NCBI
AgLimS	AAB70907	*Abies grandis*	NCBI
AgPinS	AAB71085	*Abies grandis*	NCBI
AgCamS	AAB70707	*Abies grandis*	NCBI
AgSelS	AAC05727	*Abies grandis*	NCBI
AgHumS	AAC05728	*Abies grandis*	NCBI
PaLimS	AAS47694	*Picea abies*	NCBI
PaPinS	AAS47692	*Picea abies*	NCBI
PaMyrS	AAS47696	*Picea abies*	NCBI
PaFarS	AAS47697	*Picea abies*	NCBI
PaLonS	AAS47695	*Picea abies*	NCBI

Table S2. Primers used in this study

Gene ID	Forward primer	Reverse primer
CbTPS1	gagggaaggatttcagaattcATGGAAGCATCAAGCATTACT	cagtgccaagcttgcctgcagTTAAAGGAGAAATGGATGCAGG
CbTPS1^{Y465A}	TATCCgctAGCTTGGTTGTAATGGAAG	CAACCAAGCTagcGGATACATAGTCG
CbTPS1^{T351A}	AACTCgctATGATCTTGACGGTTATTG	GTCAAGATCATagcGAGTTTAGCTG
CbTPS1^{T355A}	TCTTGgctGTTATTGATGATACATATG	CATCAATAACagcCAAGATCATAGTG
CbTPS1^{D358A}	TTATTgctGATACATATGATGCCTATG	CATATGTATCagcAATAACCGTCAAG
CbTPS1^{L354A}	CACTATGATCgcgACGGTTATTGATGATACAT	CCGTcgcGATCATAGTGAGTTTAGCTGTTA
CbTPS1^{L354M}	CACTATGATCatgACGGTTATTGATGATACAT	CCGTcatGATCATAGTGAGTTTAGCTGTTATC
CbTPS1^{L354N}	CACTATGATCaatACGGTTATTGATGATACAT	CCGTattGATCATAGTGAGTTTAGCTGTTA
CbTPS1^{L354Y}	CACTATGATCtatACGGTTATTGATGATACAT	CCGTataGATCATAGTGAGTTTAGCTGTTA
CbTPS1^{L354C}	CACTATGATCtgtACGGTTATTGATGATACAT	CCGTacaGATCATAGTGAGTTTAGCTGTTA
CbTPS1^{L354T}	CACTATGATCactACGGTTATTGATGATACAT	CCGTagtGATCATAGTGAGTTTAGCTGTTA
CbTPS1^{L354R}	CACTATGATCcgtACGGTTATTGATGATACAT	CCGTacgGATCATAGTGAGTTTAGCTGTTATC
CbTPS1^{L354K}	CACTATGATCaaaACGGTTATTGATGATACAT	CCGTtttGATCATAGTGAGTTTAGCTGTTA
CbTPS1^{L354D}	CACTATGATCgatACGGTTATTGATGATACAT	CCGTatcGATCATAGTGAGTTTAGCTGTTA
CbTPS1^{L354I}	CACTATGATCattACGGTTATTGATGATACAT	CCGTaatGATCATAGTGAGTTTAGCTGTTA
CbTPS1^{L354V}	CACTATGATCgttACGGTTATTGATGATACA	CCGTaacGATCATAGTGAGTTTAGCTGTTA
CbTPS1^{L354Q}	CACTATGATCcaaACGGTTATTGATGATACAT	CCGTttgGATCATAGTGAGTTTAGCTGTTATC
CbTPS1^{L354W}	CACTATGATCtggACGGTTATTGATGATACAT	CCGTccaGATCATAGTGAGTTTAGCTGTTATC
CbTPS1^{L354S}	CACTATGATCagtACGGTTATTGATGATACAT	CCGTactGATCATAGTGAGTTTAGCTGTTATC
CbTPS1^{L354P}	CACTATGATCccaACGGTTATTGATGATACAT	CCGTtggGATCATAGTGAGTTTAGCTGTTATC
CbTPS1^{L354H}	CACTATGATCcatACGGTTATTGATGATACAT	CCGTatgGATCATAGTGAGTTTAGCTGTTATC
CbTPS1^{L354G}	CACTATGATCgggACGGTTATTGATGATACAT	CCGTcccGATCATAGTGAGTTTAGCTGTTATC
CbTPS1^{L354F}	CACTATGATCtttACGGTTATTGATGATACAT	CCGTaaaGATCATAGTGAGTTTAGCTGTTATC
CbTPS1^{L354E}	CACTATGATCgaaACGGTTATTGATGATACAT	CCGTttcGATCATAGTGAGTTTAGCTGTTATC

Table S3. ^{1}H (700 MHz) and ^{13}C (150 MHz) NMR data of (+)-capbuene A (**1**) in $CDCl_3$

Position	δ_H (ppm), J (Hz)	δ_C (ppm)
1a	1.79 m	44.4 CH_2
1b	1.04 m	
2	1.78 m	42.2 CH
3	1.30 m	51.0 CH
4a	1.78 m	38.1 CH_2
4b	1.24 m	
5a	1.75 m	31.0 CH_2
5b	1.29 m	
6	1.57 q (7.2)	63.2 CH
7	1.51 m	41.9 CH
8a	1.81 m	37.7 CH_2
8b	1.19 m	
9a	1.67 ddd (11.8, 6.2, 3.1)	28.3 CH_2
9b	1.38 m	
10		67.5 C
11		53.7 C
12	2.11 m	45.4 CH
13a	2.20 m	22.0 CH_2
13b	2.07 m	
14		138.1 C
15		46.8 C
16a	1.64 ddd (11.9, 7.6, 1.5)	40.9 CH_2
16b	1.38 m	
17a	2.25 m	27.7 CH_2
17b	2.09 m	
18		137.2 C
19	2.67 hept (6.9)	26.8 CH
20	0.93 d (6.9)	21.3 CH_3
21	0.89 d (7.1)	14.4 CH_3
22	0.97 d (6.6)	20.5 CH_3
23	0.90 s	25.1 CH_3
24	0.98 s	24.5 CH_3
25	0.95 d (6.9)	20.9 CH_3

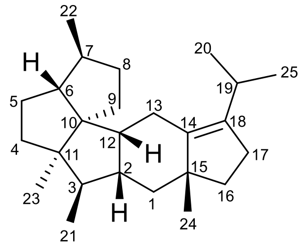

(+)-Capbuene A (**1**)

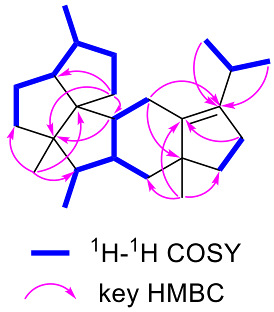

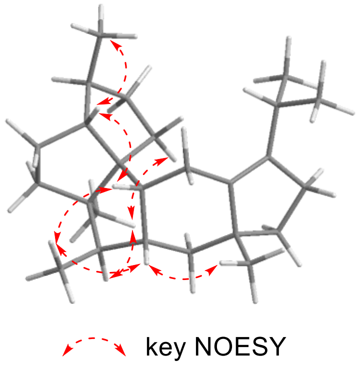

Table S4. ^{1}H (700 MHz) and ^{13}C (150 MHz) NMR data of (+)-capbuene B (**2**) in $CDCl_3$

Position	δ_H (ppm), *J* (Hz)	δ_C (ppm)
1a	1.73 dd (12.0, 5.6)	43.6 CH_2
1b	0.72 t (12.0)	
2	1.90 m	41.6 CH
3	1.20 m	50.3 CH
4a	1.93 m	38.0 CH_2
4b	1.42 m	
5a	1.95 m	28.9 CH_2
5b	1.46 m	
6	1.77 m	61.0 CH
7	1.49 m	43.6 CH
8a	1.82 m	39.3 CH_2
8b	1.20 m	
9	1.35 m	27.0 CH_2
10		67.9 C
11		55.6 C
12	2.50 dt (10.1, 3.2)	49.0 CH
13	5.30 m	118.1 CH
14		151.8 C
15		42.3 C
16a	1.57 dd (11.6, 7.0)	40.8 CH_2
16b	1.17 m	
17a	1.66 dt (12.7, 7.0)	25.1 CH_2
17b	1.50 m	
18	2.38 m	49.9 CH
19	1.77 m	32.1 CH
20	0.95 d (6.5)	21.9 CH_3
21	0.94 d (7.1)	15.1 CH_3
22	0.92 d (6.5)	19.4 CH_3
23	0.91 s	22.8 CH_3
24	0.87 s	24.0 CH_3
25	0.83 d (6.5)	19.1 CH_3

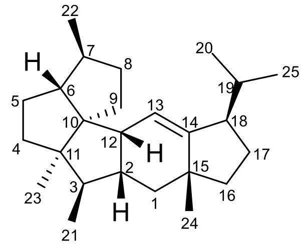

(+)-Capbuene B (**2**)

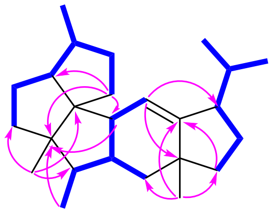

^{1}H-^{1}H COSY

key HMBC

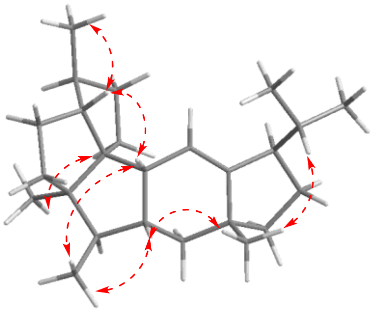

key NOESY

Table S5. ^{1}H (700 MHz) and ^{13}C (150 MHz) NMR data of (+)-epoxy-capbuene B (**2a**) in $CDCl_3$

Position	δ_H (ppm), J (Hz)	δ_C (ppm)
1a	1.42 m	43.6 CH_2
1b	0.95 m	
2	1.51 m	40.6 CH
3	1.21 m	50.1 CH
4a	1.93 m	38.2 CH_2
4b	1.42 m	
5a	1.94 m	29.1 CH_2
5b	1.44 m	
6	1.87 m	60.8 CH
7	1.52 m	42.8 CH
8a	1.97 m	38.8 CH_2
8b	1.26 m	
9a	1.63 ddd (13.7, 10.0, 7.5)	28.6 CH_2
9b	1.39 m	
10		66.5 C
11		55.2 C
12	2.34 br d (8.6)	48.4 CH
13	2.88 br s	59.0 CH
14		70.3 C
15		39.3 C
16a	1.70 dt (12.1, 4.2)	40.9 CH_2
16b	1.32 m	
17	1.59 m	23.6 CH_2
18	1.93 m	48.5 CH
19	1.57 m	26.9 CH
20	0.92 d (7.0)	22.5 CH_3
21	0.92 d (7.0)	15.4 CH_3
22	0.94 d (6.2)	19.5 CH_3
23	0.93 s	23.4 CH_3
24	0.85 s	21.3 CH_3
25	0.89 d (6.7)	19.8 CH_3

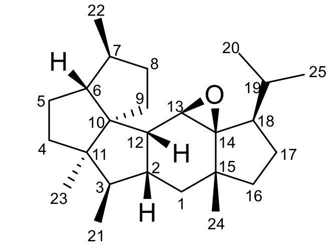

(+)-Epoxy-capbuene B (**2a**)

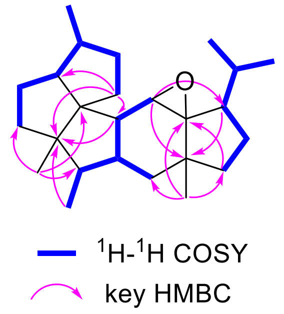

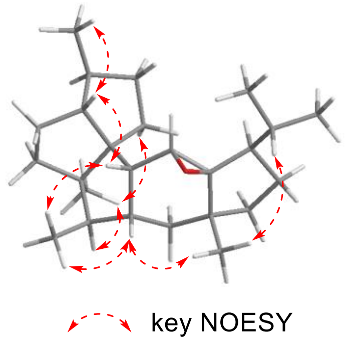

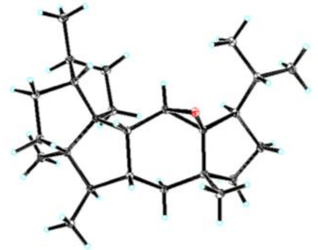

2a (X-ray)

Table S6. ^{1}H (700 MHz) and ^{13}C (150 MHz) NMR data of (+)-capbuene C (**3**) in $CDCl_3$

Position	δ_H (ppm), J (Hz)	δ_C (ppm)
1a	2.27 m	25.7 CH_2
1b	2.23 m	
2	1.60 m	42.5 CH
3	1.45 m	43.0 CH
4a	1.62 m	37.9 CH_2
4b	1.19 m	
5a	1.51 m	31.9 CH_2
5b	1.19 m	
6	1.43 m	64.6 CH
7	1.47 m	42.2 CH
8a	1.74 dtd (11.8, 6.0, 2.8)	36.4 CH_2
8b	1.12 tdd (11.8, 9.7, 6.0)	
9a	1.65 m	29.2 CH_2
9b	1.32 ddd (13.1, 11.8, 6.0)	
10		65.7 C
11		52.2 C
12	1.87 m	45.5 CH
13a	1.79 dd (13.0, 5.6)	41.5 CH_2
13b	1.03 t (13.0)	
14		47.0 C
15		147.8 C
16	5.19 m	120.3 CH
17a	2.25 m	35.9 CH_2
17b	1.87 m	
18	1.36 m	60.7 CH
19	1.69 m	29.6 CH
20	0.97 d (6.5)	23.1 CH_3
21	0.80 d (6.9)	12.2 CH_3
22	1.00 d (6.6)	21.0 CH_3
23	0.92 s	26.5 CH_3
24	0.89 s	16.4 CH_3
25	0.88 d (6.6)	23.1 CH_3

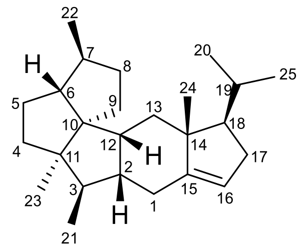

(+)-Capbuene C (**3**)

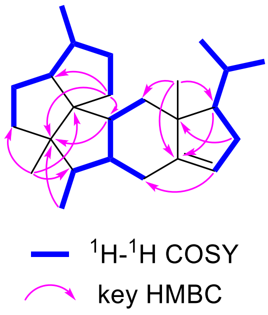

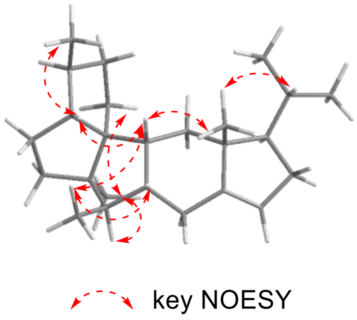

Table S7. ^{1}H (700 MHz) and ^{13}C (150 MHz) NMR data of (–)-epoxy-capbuene C (**3a**) in $CDCl_3$

Position	δ_H (ppm), J (Hz)	δ_C (ppm)
1a	2.28 dd (14.6, 6.0)	24.7 CH_2
1b	1.42 m	
2	1.71 m	41.9 CH
3	1.96 m	44.1 CH
4a	1.62 m	37.9 CH_2
4b	1.19 m	
5a	1.54 m	32.0 CH_2
5b	1.19 m	
6	1.44 m	65.1 CH
7	1.54 m	41.7 CH
8a	1.80 dtd (12.0, 5.8, 2.5)	36.8 CH_2
8b	1.13 tdd (12.0, 9.8, 5.8)	
9a	1.67 ddd (12.5, 5.8, 2.5)	29.4 CH_2
9b	1.37 td (12.5, 5.8)	
10		65.6 C
11		51.8 C
12	1.94 m	45.5 CH
13a	1.73 dd (13.0, 5.5)	34.9 CH_2
13b	1.28 t (13.0)	
14		41.2 C
15		70.4 C
16	3.17 br s	57.5 CH
17a	2.04 dd (13.7, 6.8)	32.4 CH_2
17b	1.24 m	
18	1.05 m	49.9 CH
19	1.46 m	29.3 CH
20	0.91 d (6.5)	23.0 CH_3
21	0.84 d (7.0)	12.4 CH_3
22	1.01 d (6.7)	21.2 CH_3
23	0.95 s	26.4 CH_3
24	0.86 s	15.2 CH_3
25	0.84 d (7.0)	23.0 CH_3

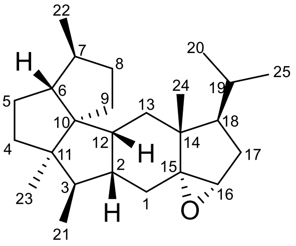

(-)-Epoxy-capbuene C (**3a**)

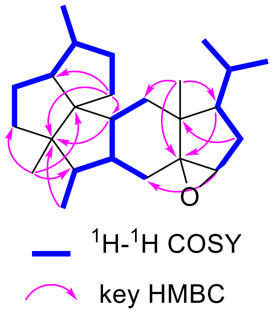

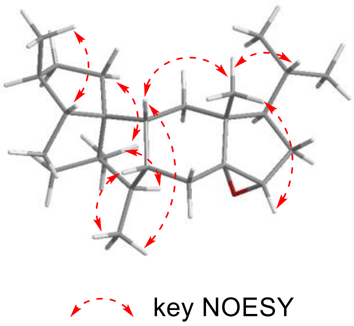

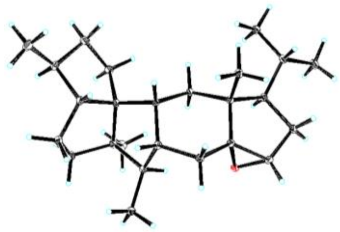

3a (X-ray)

Table S8. ^{1}H (700 MHz) and ^{13}C (150 MHz) NMR data of (+)-capbuene D (**4**) in $CDCl_3$

Position	δ_H (ppm), J (Hz)	δ_C (ppm)
1	5.40 m	119.0 CH
2	1.88 m	43.8 CH
3	1.31 dq (13.7, 6.4)	50.3 CH
4a	1.62 m	38.0 CH_2
4b	1.18 m	
5a	1.62 m	32.1 CH_2
5b	1.18 m	
6	1.51 m	65.7 CH
7	1.58 m	41.2 CH
8a	1.83 m	37.2 CH_2
8b	1.14 m	
9a	1.66 ddd (13.1, 5.8, 2.6)	29.4 CH_2
9b	1.37 m	
10		65.3 C
11		52.9 C
12	1.91 m	45.3 CH
13a	1.80 dd (13.0, 3.9)	37.2 CH_2
13b	0.95 t (13.0)	
14		44.5 C
15		149.3 C
16a	2.35 m	27.5 CH_2
16b	2.09 m	
17a	1.86 m	27.5 CH_2
17b	1.38 m	
18	1.04 dt (11.1, 8.6)	58.9 CH
19	1.58 m	30.4 CH
20	0.99 d (6.6)	23.1 CH_3
21	0.88 d (6.4)	12.5 CH_3
22	1.00 d (6.8)	21.4 CH_3
23	0.87 s	25.7 CH_3
24	0.86 s	18.1 CH_3
25	0.88 d (6.4)	23.1 CH_3

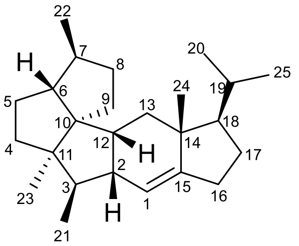

(+)-Capbuene D (**4**)

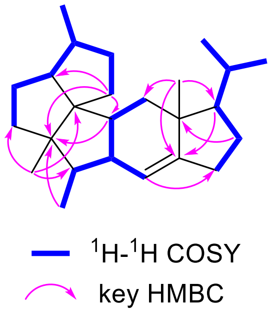

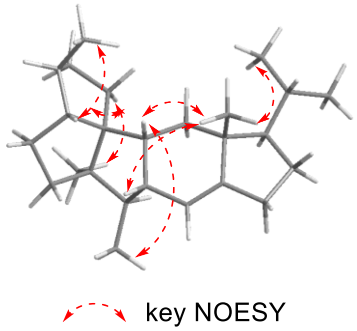

Table S9. ^{1}H (700 MHz) and ^{13}C (150 MHz) NMR data of (+)-capbuene E (**5**) in $CDCl_3$

Position	δ_H (ppm), J (Hz)	δ_C (ppm)
1a	1.67 m	45.5 CH_2
1b	1.15 dd (12.9, 10.1)	
2	2.66 m	42.7 CH
3		168.4 C
4a	1.79 m	44.0 CH_2
4b	1.54 m	
5a	1.66 m	29.8 CH_2
5b	1.33 dddd (12.6, 6.6, 4.9, 3.2)	
6	1.56 m	63.9 CH_2
7	1.51 m	40.5 CH
8a	1.80 m	36.3 CH_2
8b	1.25 m	
9a	1.76 m	29.4 CH_2
9b	1.45 m	
10		55.9 C
11		67.3 C
12		45.1 CH
13a	1.71 m	23.8 CH_2
13b	1.63 m	
14	1.57 m	46.1 CH
15		41.3 C
16a	1.45 m	41.5 CH_2
16b	1.05 td (11.3, 7.7)	
17a	1.74 m	27.8 CH_2
17b	1.50 m	
18	1.67 d	46.5 CH
19	1.58 m	30.4 CH
20	0.93 d (6.3)	24.5 CH_3
21	4.77 m	102.7 CH_2
22	0.97 d (6.7)	21.0 CH_3
23	1.10 s	25.7 CH_3
24	0.81 s	19.2 CH_3
25	0.83 d (6.6)	22.1 CH_3

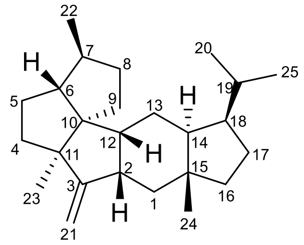

(+)-Capbuene E (**5**)

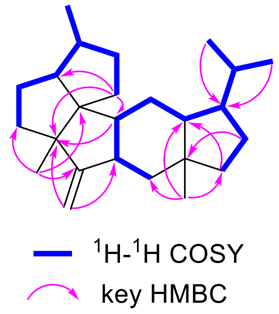

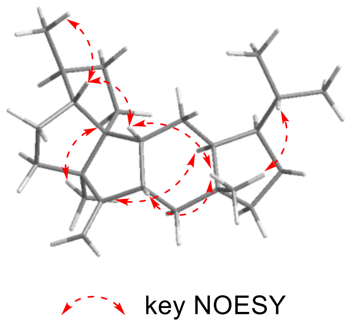

Table S10. ^{1}H (700 MHz) and ^{13}C (150 MHz) NMR data of (–)-capbuene F (**6**) in $CDCl_3$

Position	δ_H (ppm), J (Hz)	δ_C (ppm)
1	5.79 d (3.2)	129.8 CH
2		151.5 C
3		51.6 C
4	1.54 m	44.5 CH_2
5a	1.54 m	31.2 CH_2
5b	1.23 m	
6	1.50 m	63.9 CH
7	1.41 m	43.2 CH
8a	1.67 m	35.7 CH_2
8b	1.12 m	
9a	1.73 m	30.1 CH_2
9b	1.36 m	
10		65.7 C
11	1.77 m	47.3 CH
12	2.77 m	41.1 CH
13a	1.81 m	22.8 CH_2
13b	1.74 m	
14	1.49 m	48.6 CH
15		43.4 C
16a	1.49 m	37.8 CH_2
16b	1.24 m	
17a	1.86 m	29.4 CH_2
17b	1.60 m	
18	1.62 m	45.7 CH
19	1.62 m	30.7 CH
20	0.94 d (5.7)	24.5 CH_3
21	0.99 s	24.6 CH_3
22	0.98 d (6.6)	20.5 CH_3
23	0.72 d (7.1)	12.0 CH_3
24	0.83 s	20.0 CH_3
25	0.84 d (5.9)	22.2 CH_3

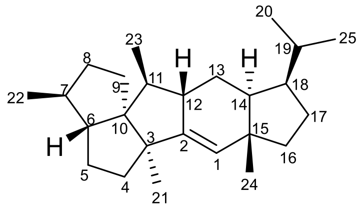

(-)-Capbuene F (**6**)

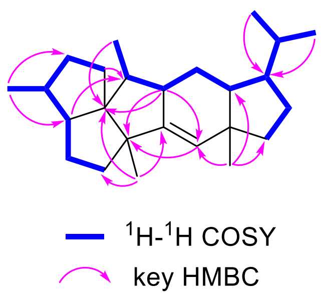

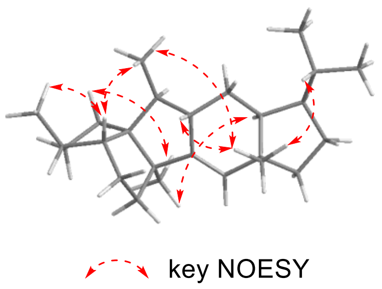

Table S11. ^{1}H (700 MHz) and ^{13}C (150 MHz) NMR data of (+)-capbuene G (**7**) in $CDCl_3$

Position	δ_H (ppm), J (Hz)	δ_C (ppm)
1a	1.51 m	42.8 CH_2
1b	0.86 m	
2	2.43 ddd (12.7, 5.2, 2.6)	49.8 CH
3		52.4 C
4a	1.52 m	40.3 CH_2
4b	1.42 m	
5a	1.72 m	29.6 CH_2
5b	1.36 m	
6	1.61 m	59.0 CH
7	1.41 m	42.2 CH
8a	1.71 m	37.1 CH_2
8b	1.21 dtd (11.9, 10.1, 6.9)	
9	1.50 m	30.9 CH_2
10		72.3 C
11		134.6 C
12		134.1 C
13a	2.50 dd (13.8, 3.9)	25.9 CH_2
13b	1.88 tt (13.8, 1.7)	
14	1.38 m	51.0 CH
15		42.7 C
16a	1.45 m	41.0 CH_2
16b	1.07 td (11.8, 8.2)	
17a	1.78 m	28.1 CH_2
17b	1.58 m	
18	1.68 m	46.6 CH
19	1.63 m	30.9 CH
20	0.95 d (6.3)	24.3 CH_3
21	0.82 s	19.4 CH_3
22	0.96 d (6.6)	19.7 CH_3
23	1.53 m	10.7 CH_3
24	0.85 s	18.3 CH_3
25	0.85 d (6.5)	22.3 CH_3

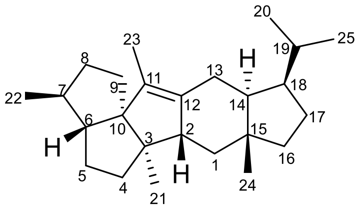

(+)-Capbuene G (**7**)

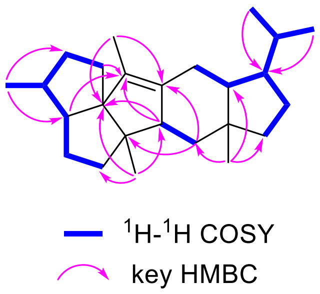

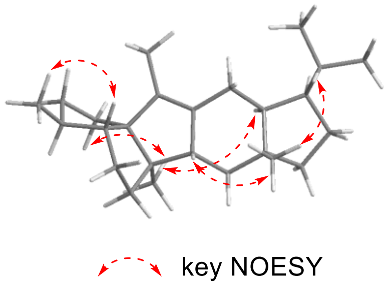

Table S12. ^{1}H (700 MHz) and ^{13}C (150 MHz) NMR data of (+)-capbuene H (**8**) in $CDCl_3$

Position	δ_H (ppm), J (Hz)	δ_C (ppm)
1a	1.47 m	41.1 CH_2
1b	0.58 t (12.7)	
2	1.82 m	44.5 CH
3		53.2 C
4a	1.78 m	43.9 CH_2
4b	1.54 m	
5a	1.79 m	31.4 CH_2
5b	1.26 m	
6	1.87 m	67.5 CH
7	1.67 m	42.0 CH
8a	1.84 m	37.1 CH_2
8b	1.50 m	
9a	1.80 m	36.2 CH_2
9b	1.64 m	
10		65.8 C
11		167.0 C
12	2.91 m	41.4 CH
13a	2.02 ddd (13.5, 3.4, 2.0)	24.2 CH_2
13b	1.72 m	
14	1.45 m	44.6 CH
15		41.9 C
16a	1.37 dd (11.5, 7.6)	41.1 CH_2
16b	0.98 m	
17a	1.72 m	27.7 CH_2
17b	1.49 m	
18	1.61 m	46.9 CH
19	1.60 m	31.1 CH
20	0.96 d (5.9)	24.3 CH_3
21	0.90 s	20.7 CH_3
22	0.99 d (6.8)	20.2 CH_3
23a	4.97 d (2.8)	102.0 CH_2
23b	4.69 d (2.8)	
24	0.76 s	18.1 CH_3
25	0.82 d (6.2)	22.4 CH_3

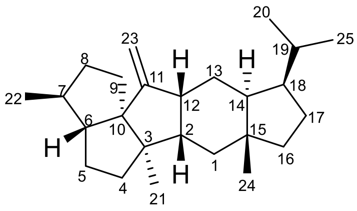

(+)-Capbuene H (**8**)

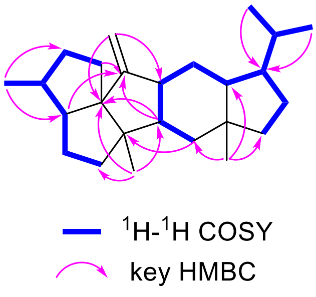

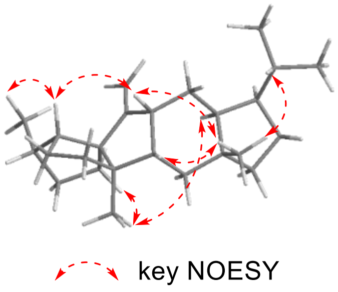

Table S13. ^{1}H (700 MHz) and ^{13}C (150 MHz) NMR data of (–)-capbunin A (**9**) in $CDCl_3$

Position	δ_H (ppm), J (Hz)	δ_C (ppm)
1a	1.58 m	42.6 CH_2
1b	1.25 m	
2	1.71 m	43.8 CH
3		52.5 C
4a	1.52 m	43.7 CH_2
4b	1.45 m	
5a	1.59 m	29.1 CH_2
5b	1.30 m	
6	1.49 m	58.4 CH
7	1.44 m	42.9 CH
8a	1.71 m	36.5 CH_2
8b	1.16 m	
9a	1.75 m	27.8 CH_2
9b	1.47 m	
10		70.9 C
11		88.5 C
12	1.90 m	43.9 CH
13a	1.88 m	22.7 CH_2
13b	1.64 m	
14	1.68 m	46.8 CH
15		41.7 C
16a	1.41 dd (11.8, 7.6)	41.1 CH_2
16b	1.07 td (11.8, 7.6)	
17a	1.73 m	27.6 CH_2
17b	1.52 m	
18	1.71 m	46.8 CH
19	1.60 m	30.3 CH
20	0.94 d (6.3)	24.5 CH_3
21	0.99 s	21.5 CH_3
22	0.96 d (6.4)	19.8 CH_3
23	1.13 s	23.0 CH_3
24	0.77 s	18.0 CH_3
25	0.83 d (6.6)	22.2 CH_3

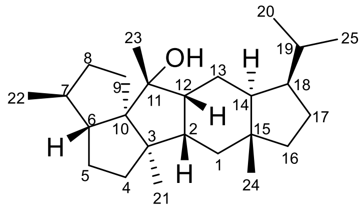

(-)-Capbunin A (**9**)

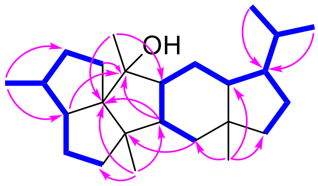

^{1}H-^{1}H COSY
key HMBC

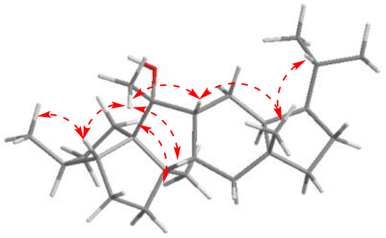
key NOESY

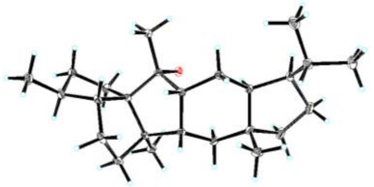
9 (X-ray)

Table S14. ^{1}H (700 MHz) and ^{13}C (150 MHz) NMR data of (–)-capbunin B (**10**) in $CDCl_3$

Position	δ_H (ppm), J (Hz)	δ_C (ppm)
1a	1.46 m	40.7 CH_2
1b	1.20 m	
2	1.53 m	50.2 CH
3		51.3 C
4a	1.48 m	40.0 CH_2
4b	1.24 m	
5a	1.63 m	25.8 CH_2
5b	1.50 m	
6	1.38 m	63.7 CH
7	1.37 m	37.9 CH
8a	1.75 m	37.3 CH_2
8b	1.08 m	
9a	1.66 m	27.2 CH_2
9b	1.53 m	
10		66.1 C
11	1.44 m	53.6 CH
12		82.3 C
13a	1.71 dd (12.8, 3.1)	35.6 CH_2
13b	1.34 m	
14	1.93 ddd (13.0, 9.1, 3.2)	46.5 CH
15		42.8 C
16	1.26 m	36.9 CH_2
17a	1.87 m	28.5 CH_2
17b	1.62 m	
18	1.63 m	46.4 CH
19	1.56 m	31.6 CH
20	0.88 d (6.2)	24.1 CH_3
21	0.89 s	22.7 CH_3
22	0.92 d (6.0)	19.8 CH_3
23	0.90 d (7.3)	8.2 CH_3
24	0.82 s	19.1 CH_3
25	0.82 d (5.1)	22.6 CH_3

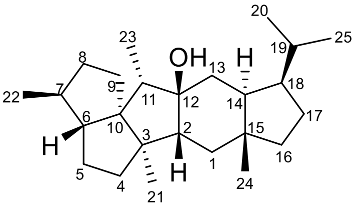

(-)-Capbunin B (**10**)

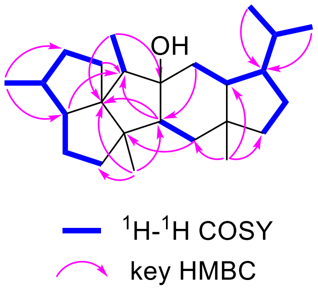

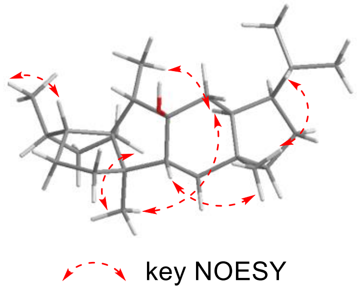

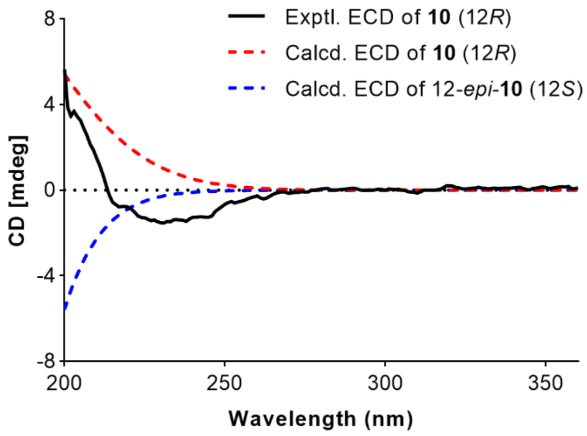

ECD calculations for the determination of 12*R* configuration of (–)-capbunin B (**10**)

Table S15. ^{1}H (700 MHz) and ^{13}C (150 MHz) NMR data of (+)-capbudiene A (**11**) in $CDCl_3$

Position	δ_H (ppm), J (Hz)	δ_C (ppm)
1a	1.60 m	42.8 CH_2
1b	1.28 m	
2	2.43 m	46.3 CH
3		155.2 C
4a	2.50 m	37.7 CH_2
4b	1.84 m	
5a	1.62 m	37.5 CH_2
5b	1.27 m	
6	2.26 m	51.1 CH
7	1.72 m	41.8 CH
8a	1.80 m	31.1 CH_2
8b	1.21 m	
9a	2.29 m	28.5 CH_2
9b	2.10 ddd (15.6, 8.9, 4.3)	
10		140.7 C
11		130.3 C
12	3.30 t (6.4)	37.5 CH
13a	1.88 m	28.2 CH_2
13b	1.85 m	
14	1.98 ddd (13.7, 9.3, 4.0)	47.0 CH
15		41.6 C
16a	1.51 dd (11.5, 7.8)	41.5 CH_2
16b	1.15 m	
17a	1.81 m	27.6 CH_2
17b	1.59 m	
18	1.68 m	46.9 CH
19	1.60 m	30.7 CH
20	0.94 d (6.3)	24.4 CH_3
21a	4.67 br s	107.5 CH_2
21b	4.56 br s	
22	0.86 d (6.9)	20.6 CH_3
23	1.61 br s	19.7 CH_3
24	0.85 s	18.5 CH_3
25	0.84 d (6.4)	22.5 CH_3

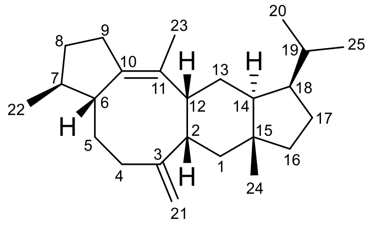

(+)-Capbudiene A (**11**)

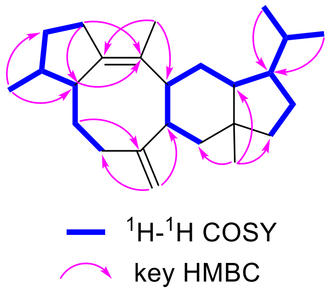

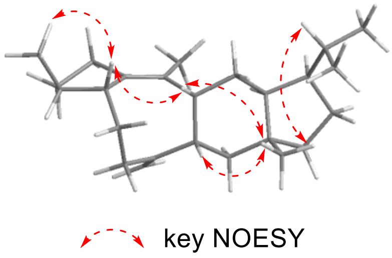

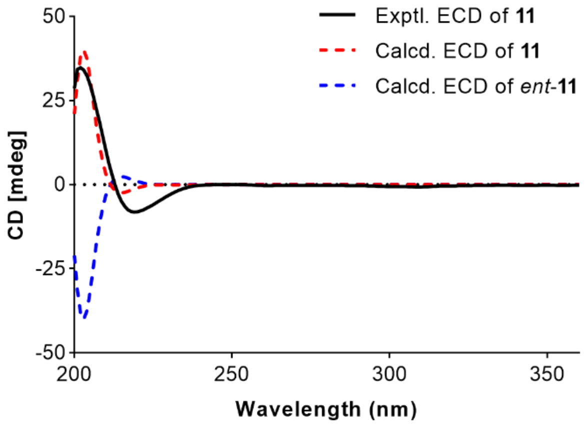

ECD calculations for the configuration determination of (+)-capbudiene A (**11**)

Table S16. ^{1}H (700 MHz) and ^{13}C (150 MHz) NMR data of (+)-capbutriene A (**12**) in $CDCl_3$

Position	δ_H (ppm), J (Hz)	δ_C (ppm)
1a	2.06 m	42.8 CH_2
1b	1.80 m	
2	5.30 m	123.0 CH
3		138.3 C
4a	2.31 m	40.6 CH_2
4b	1.82 m	
5a	2.38 m	24.3 CH_2
5b	1.61 m	
6		138.9 C
7		133.0 C
8a	2.27 m	37.6 CH_2
8b	1.26 m	
9a	1.95 ddt (14.5, 9.4, 4.7)	28.0 CH_2
9b	1.45 m	
10	3.85 m	49.0 CH
11		137.7 C
12	5.34 dd (10.0, 5.1)	127.8 CH
13a	2.13 dd (13.4, 10.0)	24.0 CH_2
13b	1.51 m	
14	1.79 m	49.8 CH
15		45.7 C
16	1.38 m	41.8 CH_2
17a	1.54 m	26.9 CH_2
17b	1.41 m	
18	1.65 m	49.8 CH
19	1.72 m	27.8 CH
20	0.95 d (6.5)	24.3 CH_3
21	1.61 s	17.0 CH_3
22	1.65 s	14.3 CH_3
23	1.42 s	19.1 CH_3
24	0.99 s	24.6 CH_3
25	0.88 d (6.7)	21.5 CH_3

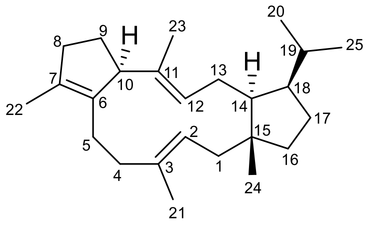

(+)-Capbutriene A (**12**)

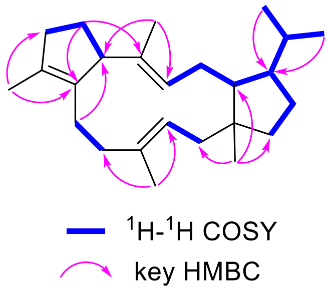

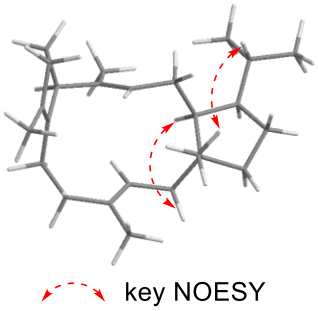

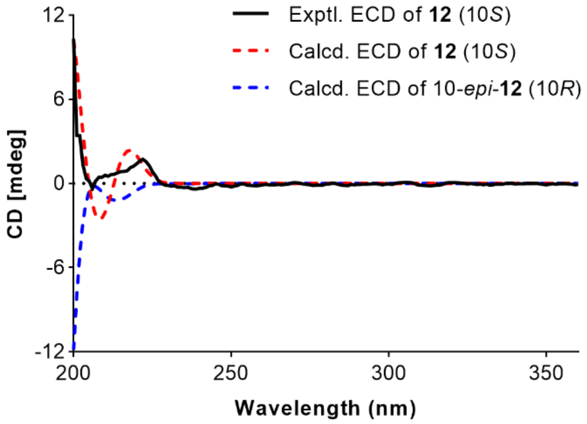

ECD calculations for the determination of 10*S* configuration of (+)-capbutriene A (**12**)

Table S17. ^{1}H (700 MHz) and ^{13}C (150 MHz) NMR data of (+)-capbutriene B (**13**) in $CDCl_3$

Position	δ_H (ppm), *J* (Hz)	δ_C (ppm)
1a	1.44 m	41.0 CH_2
1b	1.08 td (11.5, 8.4)	
2	1.66 m	46.7 CH
3		154.7 C
4a	2.36 m	37.3 CH_2
4b	1.62 m	
5a	2.27 m	24.9 CH_2
5b	1.61 m	
6	5.07 br s	126.7 CH
7		133.0 C
8	2.05 m	40.0 CH_2
9	2.12 m	24.9 CH_2
10	4.83 br s	125.0 CH
11		139.3 C
12	1.61 m	53.6[a] CH
13a	1.82 m	31.8 CH_2
13b	1.49 m	
14	1.50 m	51.4 CH
15		42.0 C
16a	1.74 m	48.8 CH_2
16b	0.90 m	
17a	1.81 m	28.2 CH_2
17b	1.54 m	
18	1.67 m	47.2 CH
19	1.58 m	31.4 CH
20	0.90 d (6.3)	24.2 CH_3
21	4.83 br s	107.2 CH_2
22	1.46 s	15.2 CH_3
23	1.56 s	17.5[a] CH_3
24	0.86 s	19.5 CH_3
25	0.82 d (6.5)	22.6 CH_3

[a] assignments were deduced by analysis of 2D NMR.

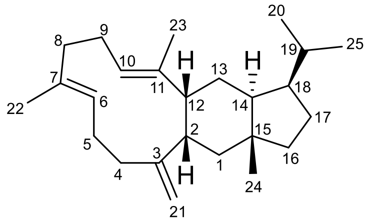

(+)-Capbutriene B (**13**)

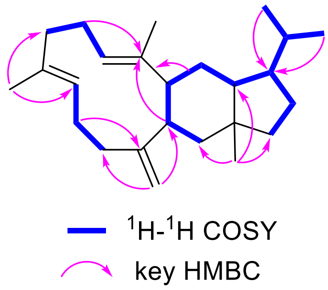

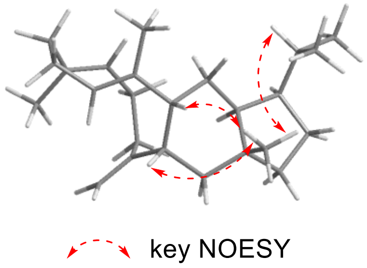

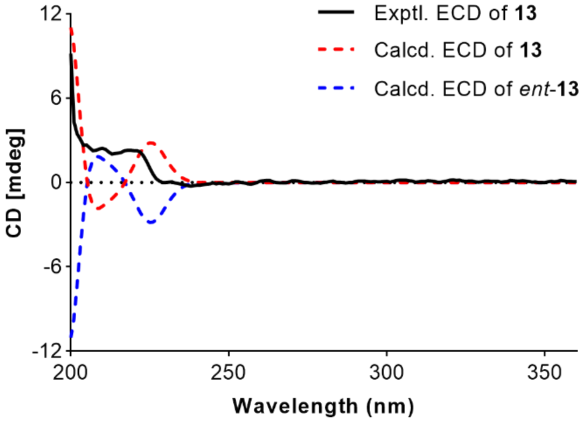

ECD calculations for the configuration determination of (+)-capbutriene B (**13**)

Table S18. ^{1}H (700 MHz) and ^{13}C (150 MHz) NMR data of (–)-capbunin C (**14**) in $CDCl_3$

Position	δ_H (ppm), J (Hz)	δ_C (ppm)
1a	1.70 m	39.6 CH_2
1b	1.28 m	
2a	2.26 m	28.2 CH_2
2b	1.87 m	
3		151.2 C
4a	2.12 m	35.9 CH_2
4b	2.04 m	
5a	2.08 m	27.8 CH_2
5b	1.85 m	
6		135.1 C
7		127.9 C
8a	2.19 m	31.5 CH_2
8b	1.99 m	
9a	1.78 m	25.3 CH_2
9b	1.69 m	
10	3.59 br d (12.1)	75.4 CH
11		48.0 C
12	5.30 d (15.4)	140.2 CH
13	5.68 dd (15.4, 10.8)	131.1 CH
14	2.42 t (10.8)	58.8 CH
15		46.0 C
16a	1.36 m	42.7 CH_2
16b	1.30 m	
17a	1.69 m	27.1 CH_2
17b	1.55 m	
18	1.87 m	50.4 CH
19	1.66 m	30.4 CH
20	0.91 d (6.4)	24.5 CH_3
21	4.63 s	109.4 CH_2
22	1.64 s	19.7 CH_3
23	1.10 s	15.9 CH_3
24	0.93 s	18.6 CH_3
25	0.84 d (6.5)	21.5 CH_3

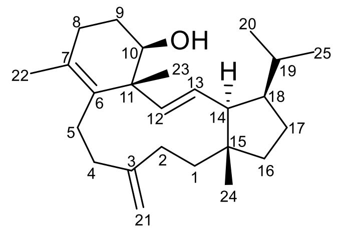

(-)-Capbunin C (**14**)

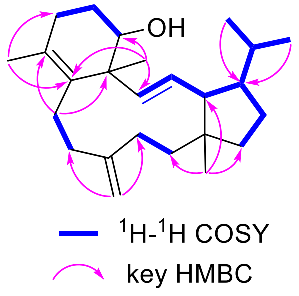

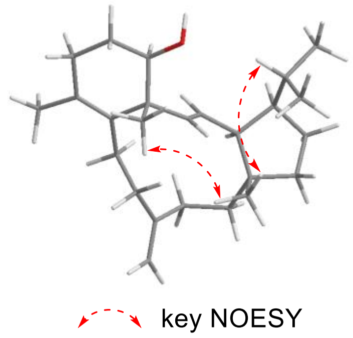

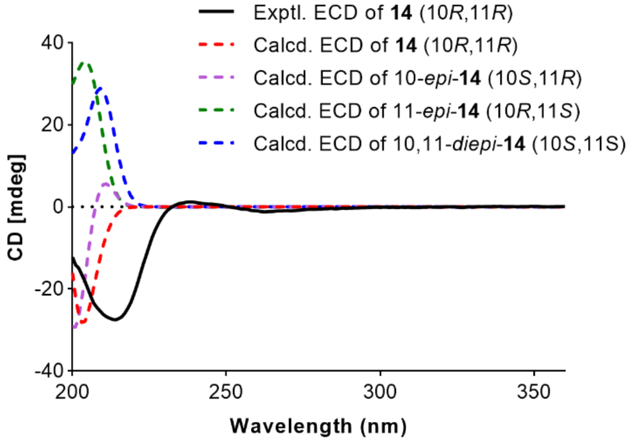

ECD calculations for the configuration determination of (–)-capbunin C (**14**)

Table S19. DP4+ evaluation of theoretical and experimental NMR data of (–)-capbunin C (**14**) [Isomer 1: **14** (10*R*,11*R*); Isomer 2: 10-*epi*-**14** (10*S*,11*R*)]

Functional		Solvent?		Basis Set		Type of Data	
mPW1PW91		PCM		6-311+G(d,p)		Unscaled Shifts	
		DP4+	100.00%	0.00%	-	-	-
Nuclei	sp2?	xperimenta	Isomer 1	Isomer 2	Isomer 3	Isomer 4	Isomer 5
C		39.6	44.5	42.7			
C		28.2	32.3	31.5			
C		151.2	164.1	162.8			
C	x	35.9	41.4	42.1			
C		27.8	31.3	30.8			
C	x	135.1	148.0	145.4			
C	x	127.9	136.8	141.1			
C		31.5	34.7	35.6			
C		25.3	27.8	31.2			
C		75.4	73.8	81.5			
C		48	52.2	54.9			
C	x	140.2	142.44	143.51			
C	x	131.1	140.59	147.99			
C		58.8	59.50	54.11			
C		46	51.58	53.32			
C		42.7	48.46	46.70			
C		27.1	26.03	30.35			
C		50.4	53.18	54.26			
C		30.4	34.71	34.41			
C	x	109.4	114.66	114.31			
C		19.7	21.72	22.51			
C		15.9	23.07	27.68			
C		18.6	20.64	21.98			
C		24.5	24.30	25.58			
C		21.5	18.37	20.11			
H		1.7	1.85	1.68			
H		1.28	1.23	1.62			
H		2.26	2.54	2.29			
H		1.87	2.06	1.93			
H		2.12	2.22	2.56			
H		2.04	2.17	2.17			
H		2.08	2.32	2.43			
H		1.85	2.01	2.18			
H		2.19	2.3	2.13			
H		1.99	2.01	2.1			
H		1.78	1.77	1.54			
H		1.69	1.75	1.51			
H		3.59	4.19	3.24			
H	x	5.3	6.1	5.48			
H	x	5.68	6.11	6.03			
H		2.42	2.53	2.65			
H		1.36	1.46	1.44			
H		1.3	1.31	1.44			
H		1.69	1.56	1.62			
H		1.55	1.54	1.57			
H		1.87	2.4	1.97			
H		1.66	1.58	1.44			
H	x	4.63	4.99	5.18			
H		1.64	1.72	1.8			
H		1.1	1.11	1.31			
H		0.93	1.15	0.96			
H		0.91	0.85	0.78			
H		0.84	0.81	0.82			

Functional	Solvent?		Basis Set		Type of Data	
mPW1PW91	PCM		6-311+G(d,p)		Unscaled Shifts	
	Isomer 1	Isomer 2	Isomer 3	Isomer 4	Isomer 5	Isomer 6
sDP4+ (H data)	100.00%	0.00%	-	-	-	-
sDP4+ (C data)	80.27%	19.73%	-	-	-	-
sDP4+ (all data)	100.00%	0.00%	-	-	-	-
uDP4+ (H data)	95.92%	4.08%	-	-	-	-
uDP4+ (C data)	99.25%	0.75%	-	-	-	-
uDP4+ (all data)	99.97%	0.03%	-	-	-	-
DP4+ (H data)	100.00%	0.00%	-	-	-	-
DP4+ (C data)	99.81%	0.19%	-	-	-	-
DP4+ (all data)	100.00%	0.00%	-	-	-	-

Table S20. ^{1}H (700 MHz) and ^{13}C (150 MHz) NMR data of (–)-sesterviolene E (**15**) in $CDCl_3$

Position	δ_H (ppm), J (Hz)	δ_C (ppm)
1a	1.44 m	39.8 CH_2
1b	1.35 m	
2a	2.04 m	30.4 CH_2
2b	1.76 m	
3		150.8 C
4a	2.06 m	37.4 CH_2
4b	2.01 m	
5	2.05 m	25.3 CH_2
6	4.96 t (5.1)	126.2 CH
7		133.3 C
8a	2.15 m	39.3 CH_2
8b	2.23 m	
9a	2.41 m	24.9 CH_2
9b	2.10 m	
10	5.10 dd (11.3, 3.8)	131.0 CH
11		133.6 C
12	5.96 d (15.4)	137.4 CH
13	5.53 dd (15.4, 10.8)	126.8 CH
14	2.36 t (10.8)	56.1 CH
15		46.2 C
16a	1.37 m	40.7 CH_2
16b	1.33 m	
17a	1.72 m	28.7 CH_2
17b	1.51 m	
18	1.82 m	49.7 CH
19	1.66 m	30.9 CH
20	0.90 d (6.4)	24.1 CH_3
21	4.70 s	107.7 CH_2
22	1.47 s	15.2 CH_3
23	1.69 s	12.5 CH_3
24	0.80 s	20.6 CH_3
25	0.85 d (6.5)	21.7 CH_3

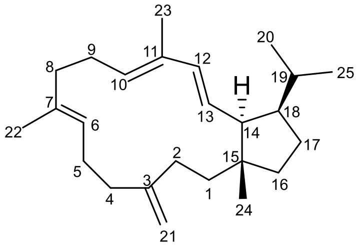

(-)-sesterviolene E (**15**)

Table S21. ^{1}H (700 MHz) and ^{13}C (150 MHz) NMR data of (+)-capbutetraene A (**16**) in $CDCl_3$

Position	δ_H (ppm), J (Hz)	δ_C (ppm)
1a	2.17 m	40.3 CH_2
1b	1.98 m	
2	5.24 t (6.9)	123.5 CH
3		134.3 C
4a	2.12 m	39.7 CH_2
4b	2.07 m	
5a	2.13 m	25.0 CH_2
5b	1.61 m	
6	5.02 t (6.3)	126.3 CH
7		133.7 C
8a	2.07 m	39.9 CH_2
8b	1.98 m	
9a	2.10 m	24.4 CH_2
9b	2.06 m	
10	5.05 t (6.2)	124.1 CH
11		135.7 C
12a	2.07 m	39.8 CH_2
12b	1.86 m	
13	2.09 m	25.7 CH_2
14		140.2 C
15		50.6 C
16a	1.57 m	39.7 CH_2
16b	1.51 m	
17	2.11 m	27.1 CH
18		141.6 C
19	2.62 hept (6.8)	27.3 CH
20	0.96 d (6.8)	21.6 CH_3
21	1.56 s	15.9 CH_3
22	1.55 s	15.7 CH_3
23	1.58 s	16.5 CH_3
24	1.04 s	25.4 CH_3
25	0.94 d (6.8)	21.5 CH_3

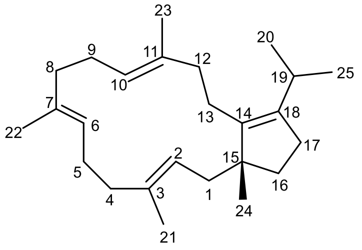

(+)-Capbutetraene A (**16**)

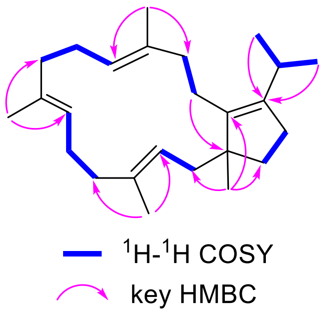

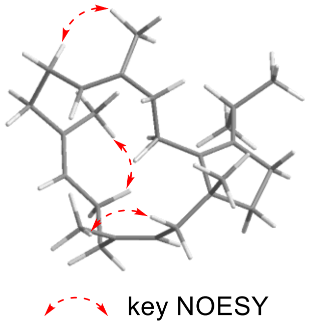

Table S22. ^{1}H (700 MHz) and ^{13}C (150 MHz) NMR data of (–)-capbupentaene A (**17**) in $CDCl_3$

Position	δ_H (ppm), *J* (Hz)	δ_C (ppm)
1a	1.50 ddd (12.7, 10.2, 6.2)	38.8 CH_2
1b	1.33 m	
2	1.90 m	31.1 CH_2
3		152.2 C
4	2.12 m	36.9 CH_2
5	2.17 m	28.7 CH_2
6	5.11 m	125.3 CH
7		134.0 C
8	2.06 m	39.1 CH_2
9	2.15 m	24.4 CH_2
10	5.03 t (7.0)	123.1 CH
11		135.0 C
12	2.62 m	42.1 CH_2
13	5.29 m	125.2 CH
14	5.19 d (15.8)	140.1 CH
15		38.8 C
16	1.30 m	42.5 CH_2
17	1.90 m	22.9 CH_2
18	5.11 m	125.4 CH
19		131.1 C
20	1.68 s	25.9 CH_3
21a	4.75 s	108.7 CH_2
21b	4.72 s	
22	1.54 s	15.6 CH_3
23	1.61 s	17.8 CH_3
24	0.97 s	23.4 CH_3
25	1.59 s	17.7 CH_3

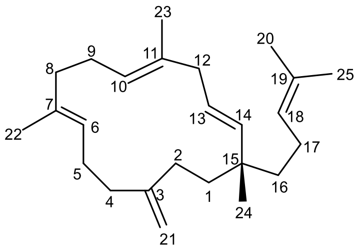

(-)-Capbupentaene A (**17**)

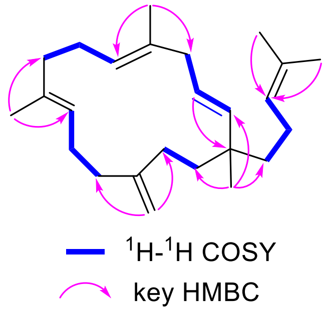

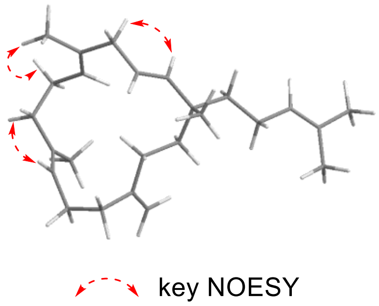

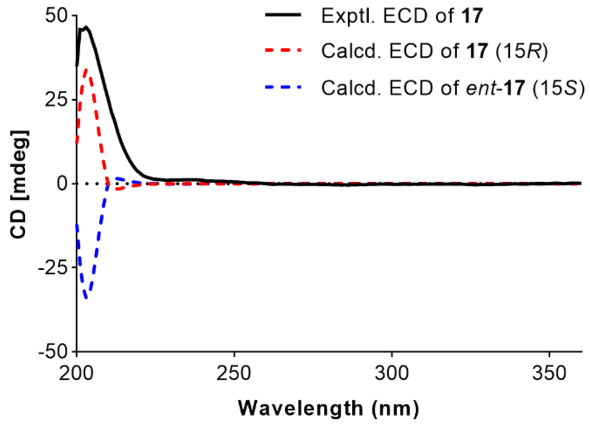

ECD calculations for the configuration determination of (–)-capbupentaene A (**17**)

Table S23. ^{1}H (700 MHz) and ^{13}C (150 MHz) NMR data of (+)-brassitetraene A (**18**) and (+)-brassitetraene B (**19**) in $CDCl_3$

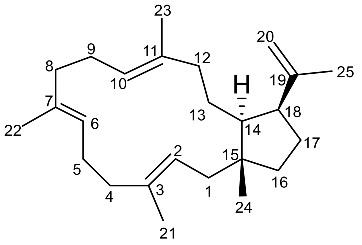

(+)-Brassitetraene A (**18**)

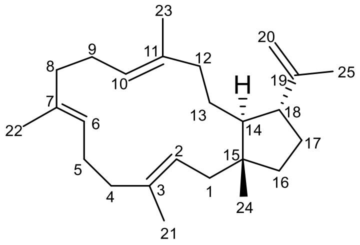

(+)-Brassitetraene B (**19**)

Position	**18**		**19**	
	δ_H (ppm), J (Hz)	δ_C (ppm)	δ_H (ppm), J (Hz)	δ_C (ppm)
1a	2.42 dd (15.3, 9.5)	41.3 CH_2	2.07 m	39.3 CH_2
1b	1.71 br d (15.3)			
2	5.15 m	122.5 CH	5.20 t (6.0)	121.2 CH
3		134.7 C		135.3 C
4a	2.22 m	40.2 CH_2	2.22 m	38.6 CH_2
4b	2.05 m		2.05 m	
5a	2.21 m	25.0 CH_2	2.21 m	24.9 CH_2
5b	1.61 m		1.61 m	
6	5.10 t (7.6)	124.7 CH	5.02 m	125.2 CH
7		133.6 C		133.9 C
8a	2.11 m	39.3 CH_2	2.06 m	38.9 CH_2
8b	1.95 m		2.01 m	
9a	2.20 m	24.5 CH_2	2.14 m	24.8 CH_2
9b	2.05 m		2.08 m	
10	5.14 m	125.8 CH	5.00 m	124.6 CH
11		134.2 C		134.7 C
12a	1.96 m	38.8 CH_2	2.01 m	37.1 CH_2
12b	1.86 ddd (13.4, 10.7, 4.0)		1.98 m	
13a	1.11 m	21.4 CH_2	1.52 m	27.2 CH_2
13b			1.30 m	
14	1.75 m	41.9 CH	1.69 m	43.6 CH
15		45.7 C		44.7 C
16a	1.54 m	38.6 CH_2	1.61 m	39.1 CH_2
16b	1.41 ddd (13.0, 8.9, 6.9)		1.25 m	
17a	2.17 m	24.8 CH_2	1.74 m	29.4 CH_2
17b	1.60 m		1.39 dddd (12.9, 9.2, 7.5, 5.3)	
18	2.61 dt (12.9, 6.8)	49.7 CH	2.30 m	54.4 CH
19		147.7 C		149.7 C
20a	4.80 s	109.7 CH_2	4.72 s	110.4 CH_2
20b	4.63 s		4.64 s	
21	1.57 s	15.9 CH_3	1.57 s	16.9 CH_3
22	1.58 s	15.8 CH_3	1.58 s	16.3 CH_3
23	1.51 s	15.7 CH_3	1.49 s	16.2 CH_3
24	1.07 s	23.8 CH_3	0.88 s	24.1 CH_3
25	1.59 s	23.7 CH_3	1.66 s	18.8 CH_3

Table S24. ^{1}H (700 MHz) and ^{13}C (150 MHz) NMR data of (–)-cericerne (**20**) in $CDCl_3$

Position	δ_H (ppm), *J* (Hz)	δ_C (ppm)
1a	2.03 m	33.0 CH_2
1b	1.93 m	
2	5.19 t (7.3)	124.3 CH
3		134.7 C
4a	2.18 m	39.0 CH_2
4b	2.12 m	
5a	2.26 m	24.9 CH_2
5b	2.18 m	
6	4.98 t (6.0)	126.0 CH
7		133.4 C
8	2.06 m	39.5 CH_2
9	2.12 m	23.7 CH_2
10	5.06 t (6.1)	121.8 CH
11		133.9 C
12a	1.95 m	34.1 CH_2
12b	1.79 m	
13a	1.72 m	28.8 CH_2
13b	1.43 m	
14	2.03 m	44.8 CH
15		153.5 C
16	1.99 m	34.3 CH_2
17	2.10 m	26.7 CH_2
18	5.14 tt (6.9, 1.4)	124.4 CH
19		131.5 C
20	1.61 s	17.7 CH_3
21	1.57 s	15.5 CH_3
22	1.59 s	15.3 CH_3
23	1.55 s	17.9 CH_3
24a	4.77 s	108.1 CH_2
24b	4.74 s	
25	1.69 s	25.7 CH_3

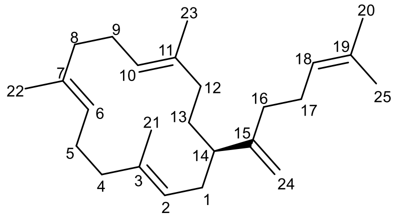

(-)-Cericerne (**20**)

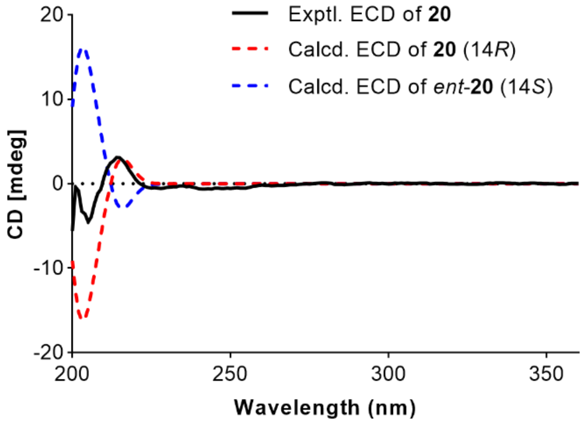

ECD calculations for the configuration determination of (–)-cericerne (**20**)

Table S25. Optimized lowest energy 3D conformers and energy analysis for **10**

**10**				**12-*epi*-10**			
No.	3D conformers	E (Hartree)	Distribution	No.	3D conformers	E (Hartree)	Distribution
1	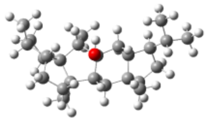	-1052.685378	38.14%	1	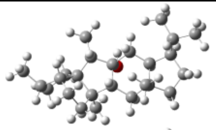	-1052.696093	9.12%
2	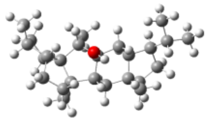	-1052.685097	28.33%	2	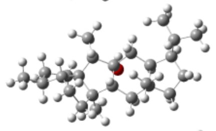	-1052.697684	49.14%
3	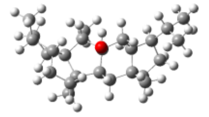	-1052.683380	4.60%	3	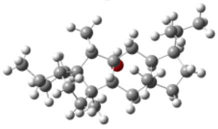	-1052.694035	1.03%
4	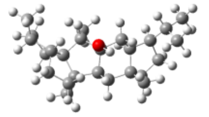	-1052.683106	3.44%	4	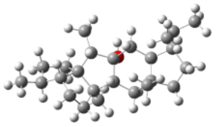	-1052.695838	6.96%
5	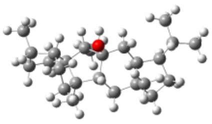	-1052.682919	2.82%	5	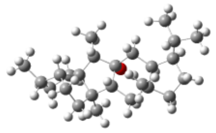	-1052.694967	2.77%
6	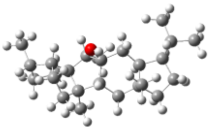	-1052.682028	1.10%	6	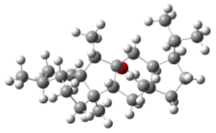	-1052.696690	17.16%
7	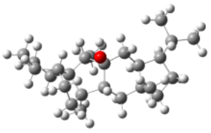	-1052.684400	13.54%	7	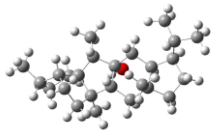	-1052.694489	1.67%
8	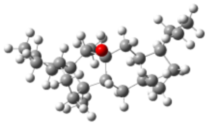	-1052.682687	2.21%	8	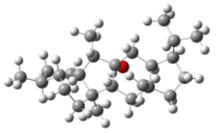	-1052.696192	10.13%
9	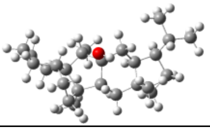	-1052.683601	5.81%	9	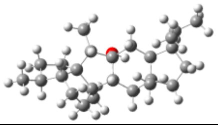	-1052.694669	2.02%

Table S26. Optimized lowest energy 3D conformers and energy analysis for **11**

No.	3D conformers	E (Hartree)	Distribution
1	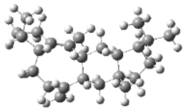	-976.251890	43.06%
2	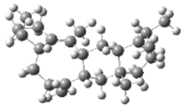	-976.249775	4.59%
3	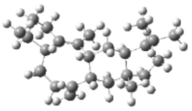	-976.251977	47.22%
4	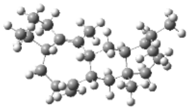	-976.249880	5.13%

Table S27. Optimized lowest energy 3D conformers and energy analysis for **12**

**12**				10-*epi*-**12**			
No.	3D conformers	E (Hartree)	Distribution	No.	3D conformers	E (Hartree)	Distribution
1	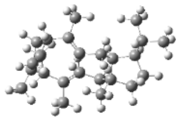	-976.246697	35.70%	1	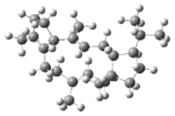	-976.249253	8.72%
2	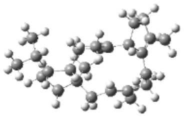	-976.246547	30.46%	2	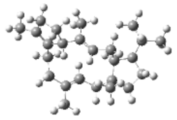	-976.250413	29.78%
3	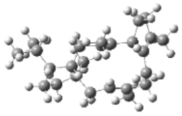	-976.246546	30.43%	3	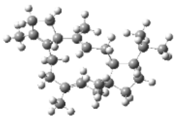	-976.251098	61.50%
4	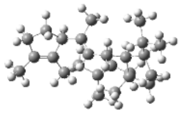	-976.24448	3.41%				

Table S28. Optimized lowest energy 3D conformers and energy analysis for **13**

No.	3D conformers	E (Hartree)	Distribution
1	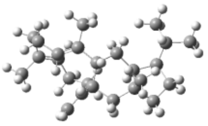	-976.214136	78.13%
2	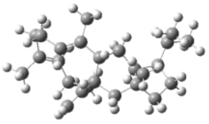	-976.212876	20.58%
3	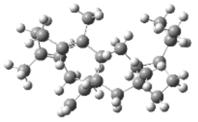	-976.210256	1.28%

Table S29. Optimized lowest energy 3D conformers and energy analysis for **14**

No.	3D conformers	E (Hartree)	Distribution	No.	3D conformers	E (Hartree)	Distribution
**14** (1)	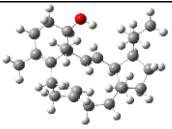	-1051.447579	25.76%	10-*epi*-**14** (1)	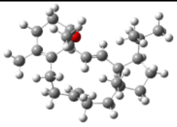	-1051.444537	4.06%
**14** (2)	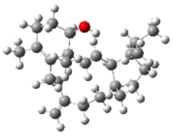	-1051.448363	59.07%	10-*epi*-**14** (2)	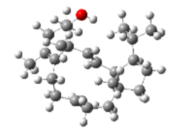	-1051.447052	58.19%
**14** (3)	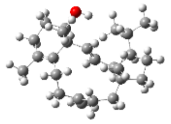	-1051.447079	15.17%	10-*epi*-**14** (3)	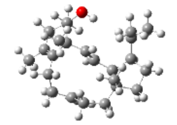	-1051.445951	18.14%
11-*epi*-**14** (1)	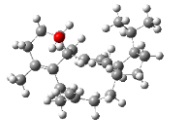	-1051.446410	3.38%	10-*epi*-**14** (4)	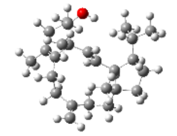	-1051.444441	3.67%
11-*epi*-**14** (2)	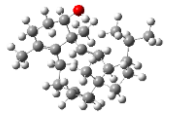	-1051.447820	15.03%	10-*epi*-**14** (5)	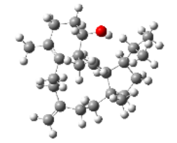	-1051.445222	8.38%
11-*epi*-**14** (3)	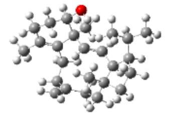	-1051.447644	12.47%	10-*epi*-**14** (6)	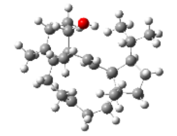	-1051.444302	3.17%
11-*epi*-**14** (4)	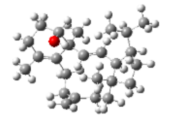	-1051.447008	6.36%	10-*epi*-**14** (7)	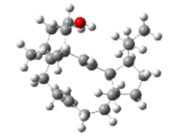	-1051.444613	4.40%
11-*epi*-**14** (5)	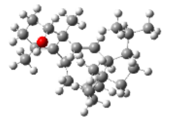	-1051.446401	3.35%	10,11-*diepi*-**14** (1)	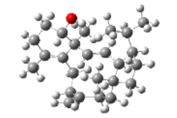	-1051.449885	8.58%
11-*epi*-**14** (6)	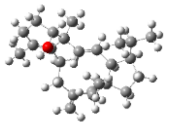	-1051.447346	9.10%	10,11-diepi-**14** (2)	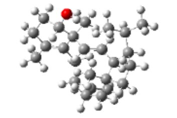	-1051.448272	1.55%
11-*epi*-**14** (7)	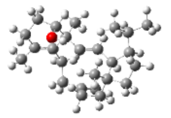	-1051.445656	1.52%	10,11-*diepi*-**14** (3)	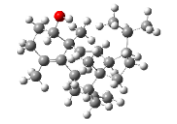	-1051.449998	9.67%
11-*epi*-**14** (8)	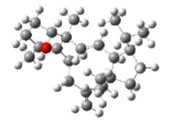	-1051.448614	34.83%	10,11-*diepi*-**14** (4)	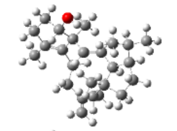	-1051.450703	20.39%
11-*epi*-**14** (9)	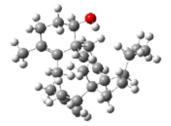	-1051.447150	7.39%	10,11-*diepi*-**14** (5)	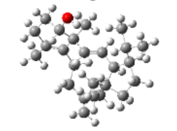	-1051.451678	57.23%
11-*epi*-**14** (10)	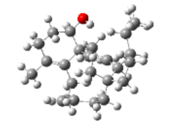	-1051.447040	6.58%	10,11-*diepi*-**14** (6)	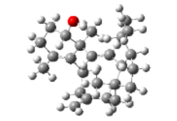	-1051.448752	2.58%

Table S30. Optimized lowest energy 3D conformers and energy analysis for **17**

No.	3D conformers	E (Hartree)	Distribution
1	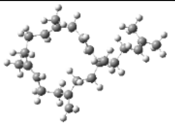	-976.237343	20.71%
2	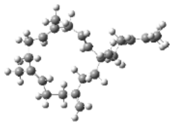	-976.237393	21.83%
3	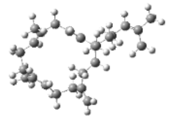	-976.235642	3.42%
4	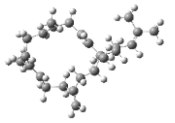	-976.235859	4.30%
5	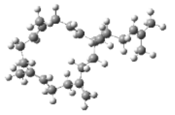	-976.235786	3.98%
6	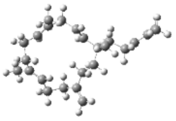	-976.235692	3.61%
7	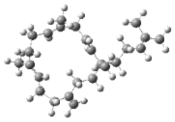	-976.236907	13.05%
8	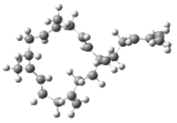	-976.236859	12.40%
9	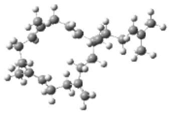	-976.235785	3.98%
10	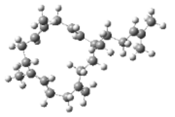	-976.235309	2.40%
11	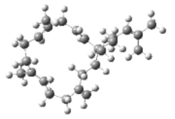	-976.235193	2.13%
12	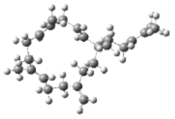	-976.235692	3.61%
13	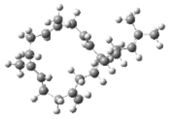	-976.235132	1.99%
14	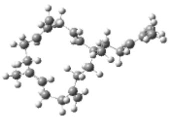	-976.235380	2.59%

Table S31. Optimized lowest energy 3D conformers and energy analysis for **20**

No.	3D conformers	E (Hartree)	Distribution
1	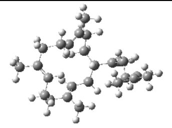	-976.237014	4.83%
2	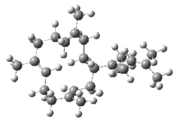	-976.237226	6.04%
3	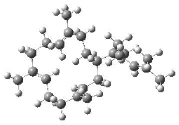	-976.235684	1.18%
4	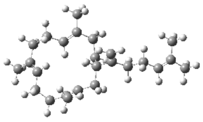	-976.238627	26.62%
5	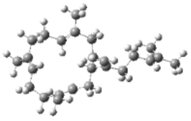	-976.236977	4.64%
6	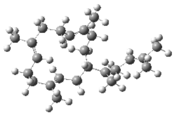	-976.236239	2.12%
7	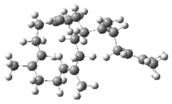	-976.238031	14.17%
8	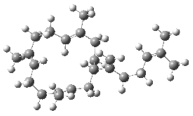	-976.236172	1.98%
9	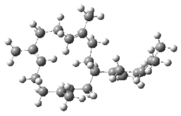	-976.237820	11.33%
10	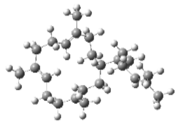	-976.235686	1.18%
11	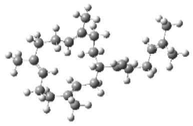	-976.236398	2.51%
12	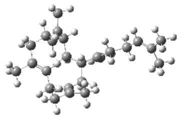	-976.238017	13.96%
13	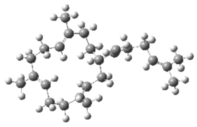	-976.236347	2.38%
14	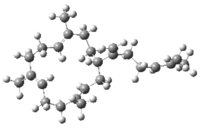	-976.236962	4.57%
15	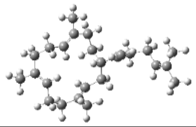	-976.236385	2.48%

Table S32. The yield of sesterterpenes produced by CbTPS1 and its variants

Enzyme	Sesterterpene yield (mg/L) [a]																	
	Total	**1**	**2**	**3**	**4**	**5** and **6**	**7**	**8** and **18**	**9**	**10**	**11**	**12**	**13** and **15**	**14**	**16**	**17**	**19**	**20**
CbTPS1	285.98±4.68	51.26±0.83	53.30±0.41	70.23±1.22	6.85±0.03	22.38±0.39	5.73±0.14	30.52±0.90	6.02±0.21	UQ	6.96±0.14	UQ	6.86±0.18	UQ	11.08±0.20	UQ	14.78±0.02	UQ
L354M	1131.94±11.38	103.36±0.41	119.97±0.79	112.15±0.79	21.90±0.52	141.01±1.61	61.75±0.98	275.05±2.54	79.49±0.92	UQ	21.50±0.56	UQ	48.72±0.84	UQ	70.26±0.77	UQ	76.78±0.66	UQ
L354A	153.64±2.31	19.52±0.14	18.36±0.10	16.36±0.15	2.27±0.06	12.76±0.17	8.72±0.09	40.79±0.23	9.32±0.05	UQ	5.17±0.06	UQ	6.11±0.07	UQ	6.49±0.05	UQ	7.77±0.14	UQ
L354I	135.22±1.17	24.33±0.12	15.38±0.11	20.28±0.13	3.24±0.04	17.80±0.29	8.33±0.05	15.44±0.16	5.29±0.03	UQ	8.27±0.07	UQ	5.63±0.08	UQ	4.29±0.06	UQ	6.94±0.03	UQ
L354C	55.89±0.54	8.28±0.03	9.39±0.07	12.60±0.09	2.58±0.02	2.93±0.03	1.95±0.02	10.56±0.07	UQ	UD	1.43±0.05	UD	1.10±0.06	UD	2.13±0.03	UQ	2.94±0.07	UQ
L354F	54.82±0.43	7.78±0.07	9.10±0.03	10.68±0.05	UQ	5.61±0.05	UQ	10.10±0.03	3.59±0.02	UD	UQ	UD	3.05±0.07	UD	1.95±0.08	UQ	2.96±0.03	UD
L354N	44.65±0.52	7.27±0.08	7.46±0.07	9.41±0.08	UQ	3.48±0.05	UQ	9.98±0.10	2.79±0.07	UD	UQ	UD	1.80±0.03	UD	UQ	UQ	2.46±0.04	UD
L354S	43.35±0.41	5.92±0.04	6.16±0.01	5.21±0.03	UQ	6.16±0.08	4.01±0.06	12.14±0.13	UQ	UD	UQ	UD	UQ	UD	3.75±0.06	UQ	UQ	UD
L354D	27.56±0.31	6.18±0.07	3.92±0.05	5.63±0.03	UQ	4.83±0.02	1.89±0.07	3.40±0.05	UQ	UD	UQ	UD	UQ	UD	UQ	UD	1.71±0.02	UD
L354V	25.21±0.26	5.59±0.07	4.45±0.02	5.15±0.06	UQ	5.36±0.09	UQ	4.66±0.02	UQ	UD	UQ	UD	UQ	UD	UQ	UD	UQ	UD
L354G	25.12±0.23	5.65±0.03	1.26±0.02	10.65±0.13	UQ	UQ	UQ	7.56±0.05	UQ	UD	UQ	UD	UQ	UD	UQ	UD	UQ	UD
L354Q	8.22±0.17	2.10±0.02	2.02±0.07	1.78±0.06	UQ	UQ	UQ	2.32±0.02	UQ	UD	UQ	UD	UQ	UD	UQ	UD	UQ	UD
L354E	7.62±0.24	1.58±0.06	1.57±0.07	1.29±0.06	UQ	UQ	UQ	3.18±0.05	UQ	UD	UQ	UD	UQ	UD	UQ	UD	UQ	UD
L354T	UQ	UQ	UQ	UQ	UQ	UQ	UQ	UQ	UQ	UD	UQ	UD	UQ	UD	UQ	UD	UQ	UD
L354H	UQ	UQ	UQ	UQ	UQ	UQ	UQ	UQ	UQ	UD	UQ	UD	UQ	UD	UQ	UD	UQ	UD
L354K	UD	UD	UD	UD	UD	UD	UD	UD	UD	UD	UD	UD	UD	UD	UD	UD	UD	UD
L354P	UD	UD	UD	UD	UD	UD	UD	UD	UD	UD	UD	UD	UD	UD	UD	UD	UD	UD
L354R	UD	UD	UD	UD	UD	UD	UD	UD	UD	UD	UD	UD	UD	UD	UD	UD	UD	UD
L354W	UD	UD	UD	UD	UD	UD	UD	UD	UD	UD	UD	UD	UD	UD	UD	UD	UD	UD
L354Y	UD	UD	UD	UD	UD	UD	UD	UD	UD	UD	UD	UD	UD	UD	UD	UD	UD	UD

[a] UD indicates that the compound was undetectable. UQ indicates that the compound was detectable but unquantifiable.

Supplementary Figures

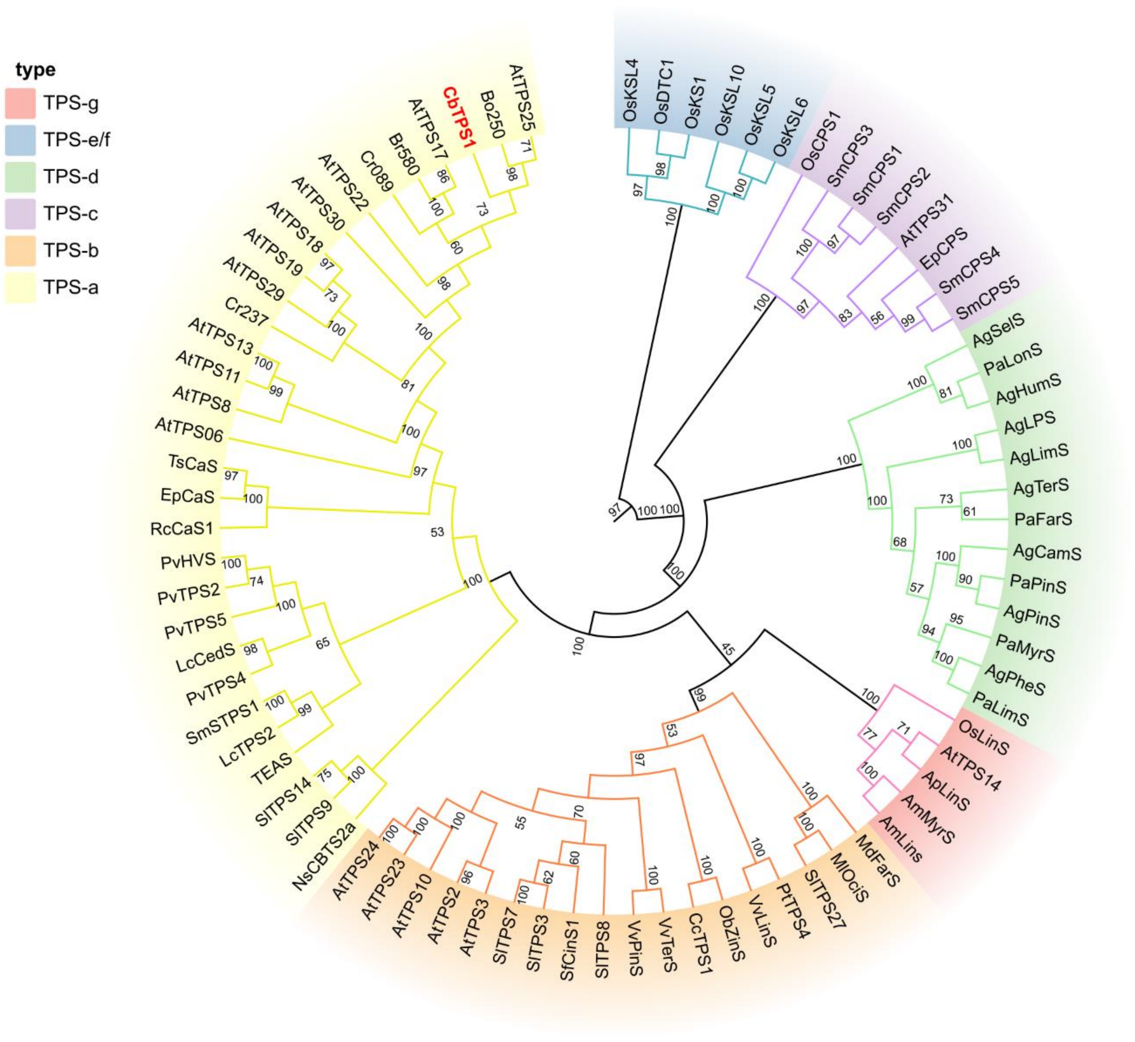

Figure S1. Phylogenetic tree of the plant TPSs listed in Table S1 using the maximum-likelihood method

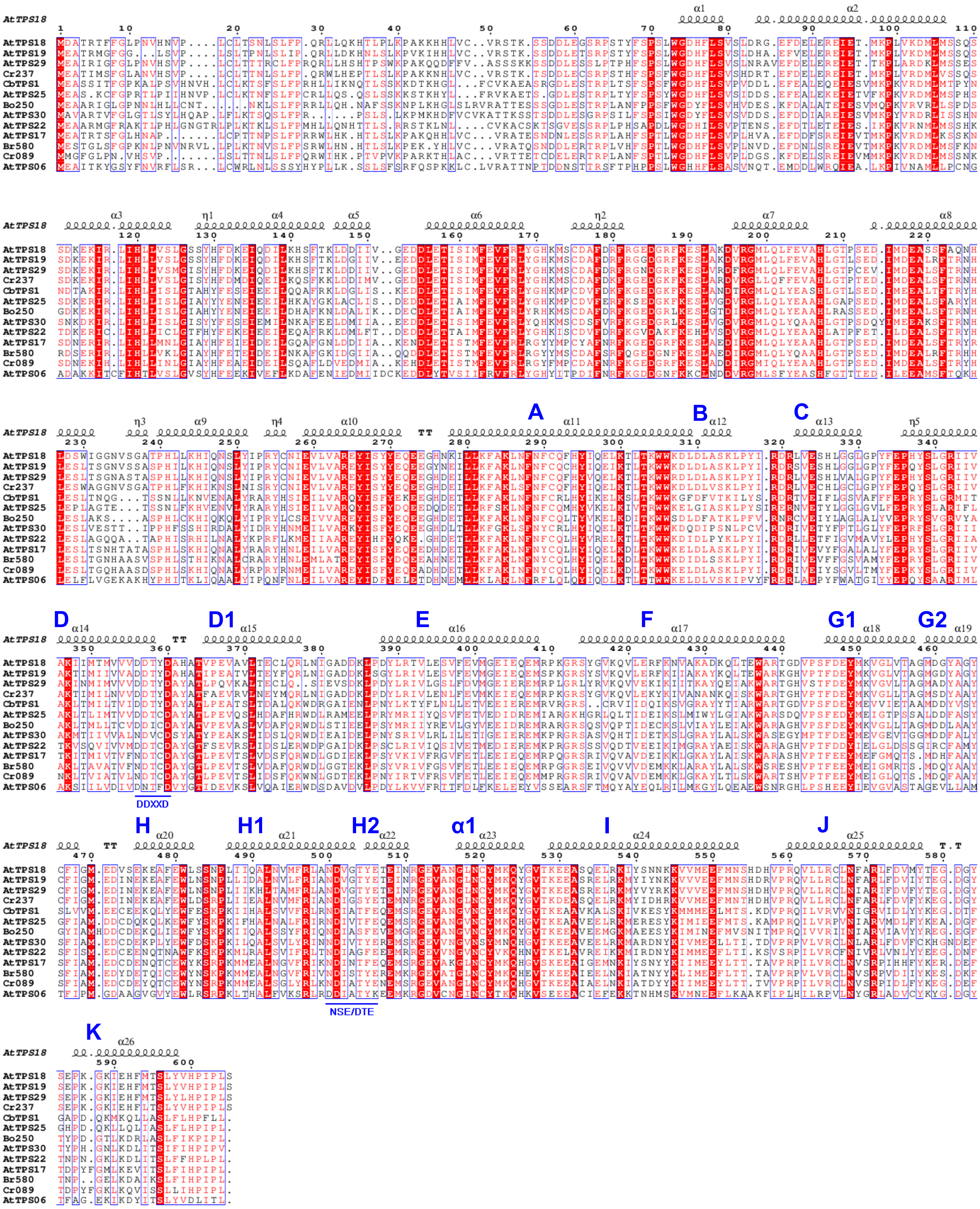

Figure S2. Multiple sequence alignment of CbTPS1 with the known plant StTSs from the Brassicaceae

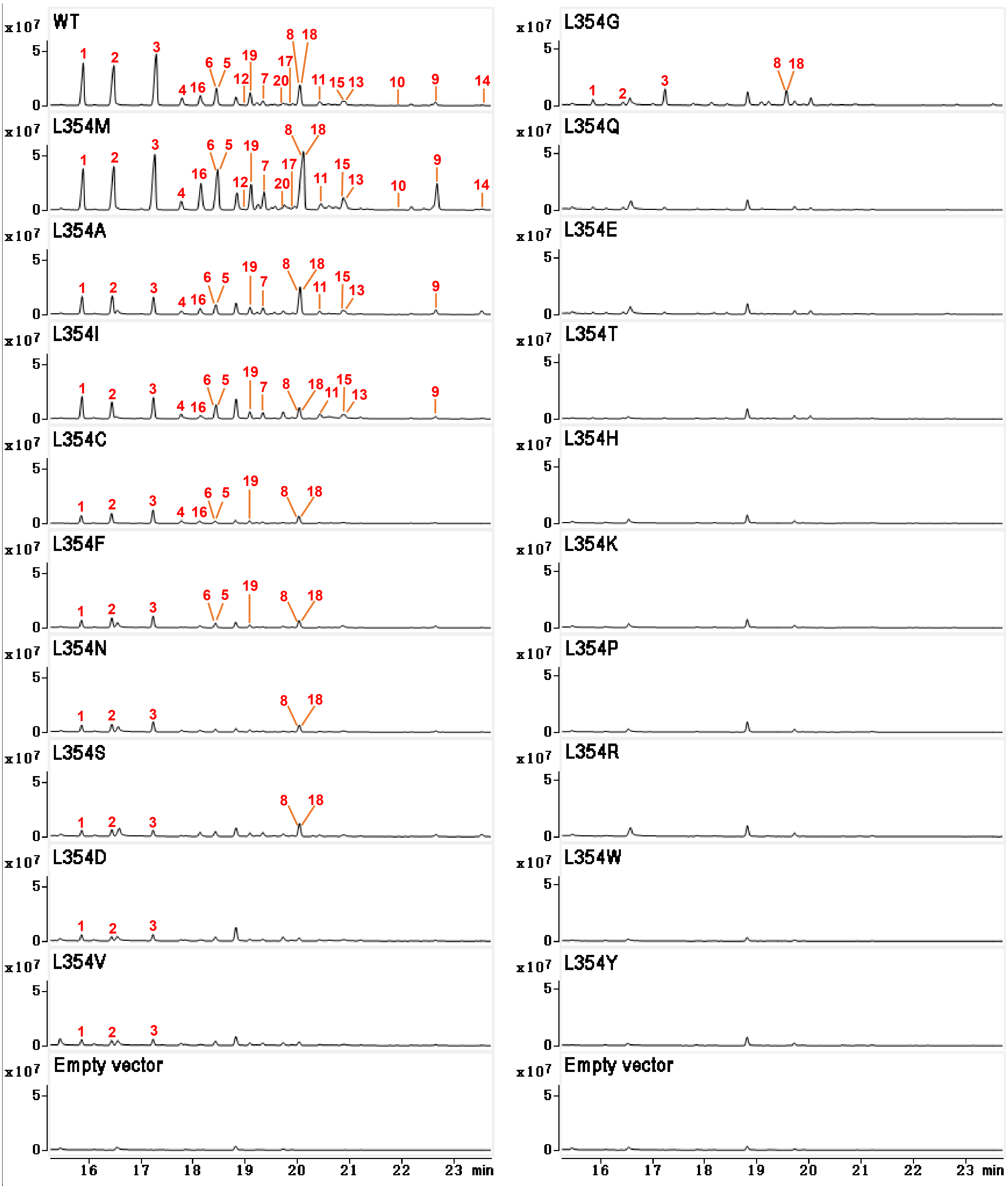

Figure S3. Total ion chromatograms (TICs) of GC-MS analysis of the metabolites produced in engineered *E. coli* heterologously expressing CbTPS1 and its variants

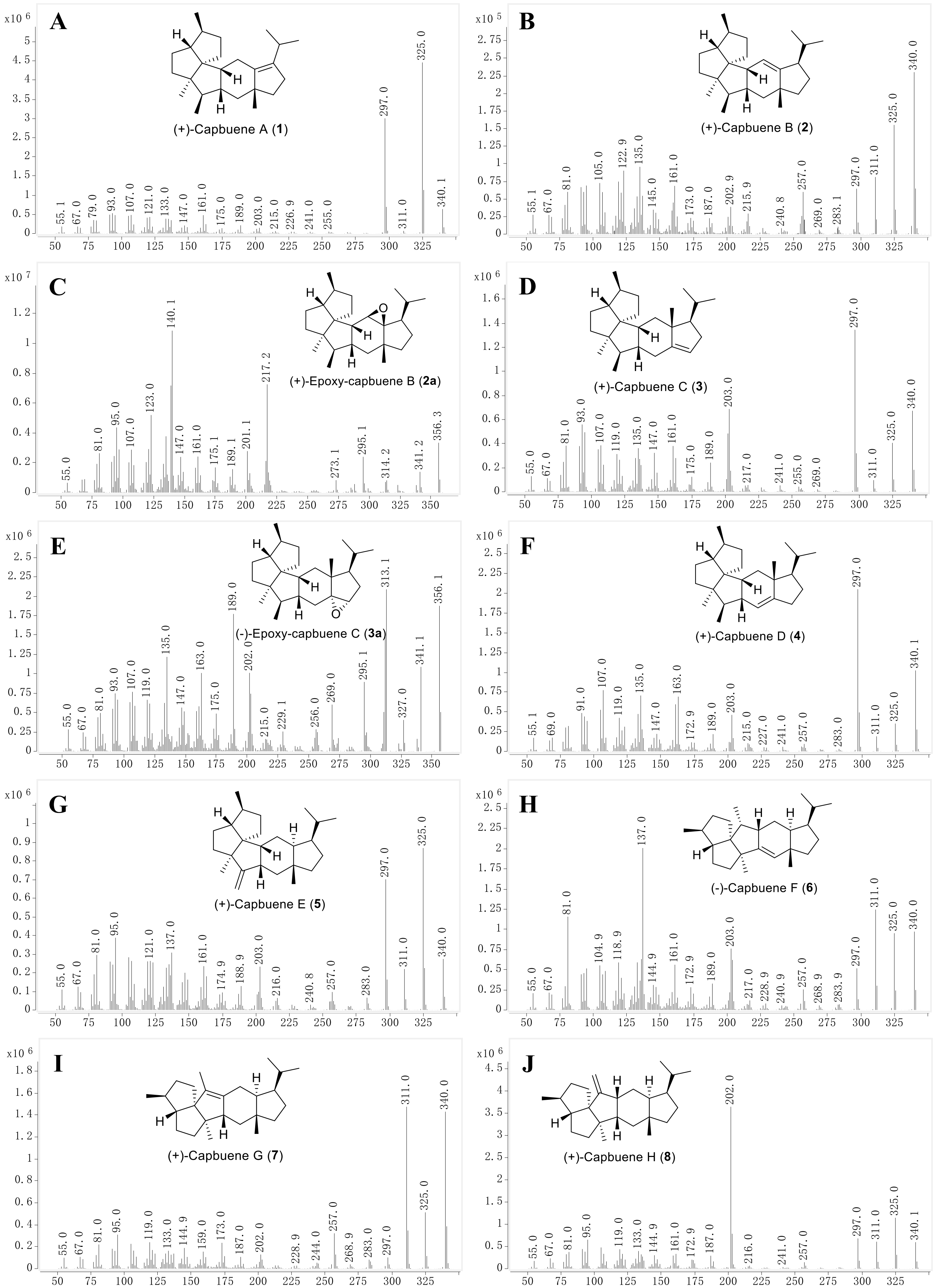

Figures S4A–J. EI-MS spectra of compounds **1–8**, **2a** and **3a**

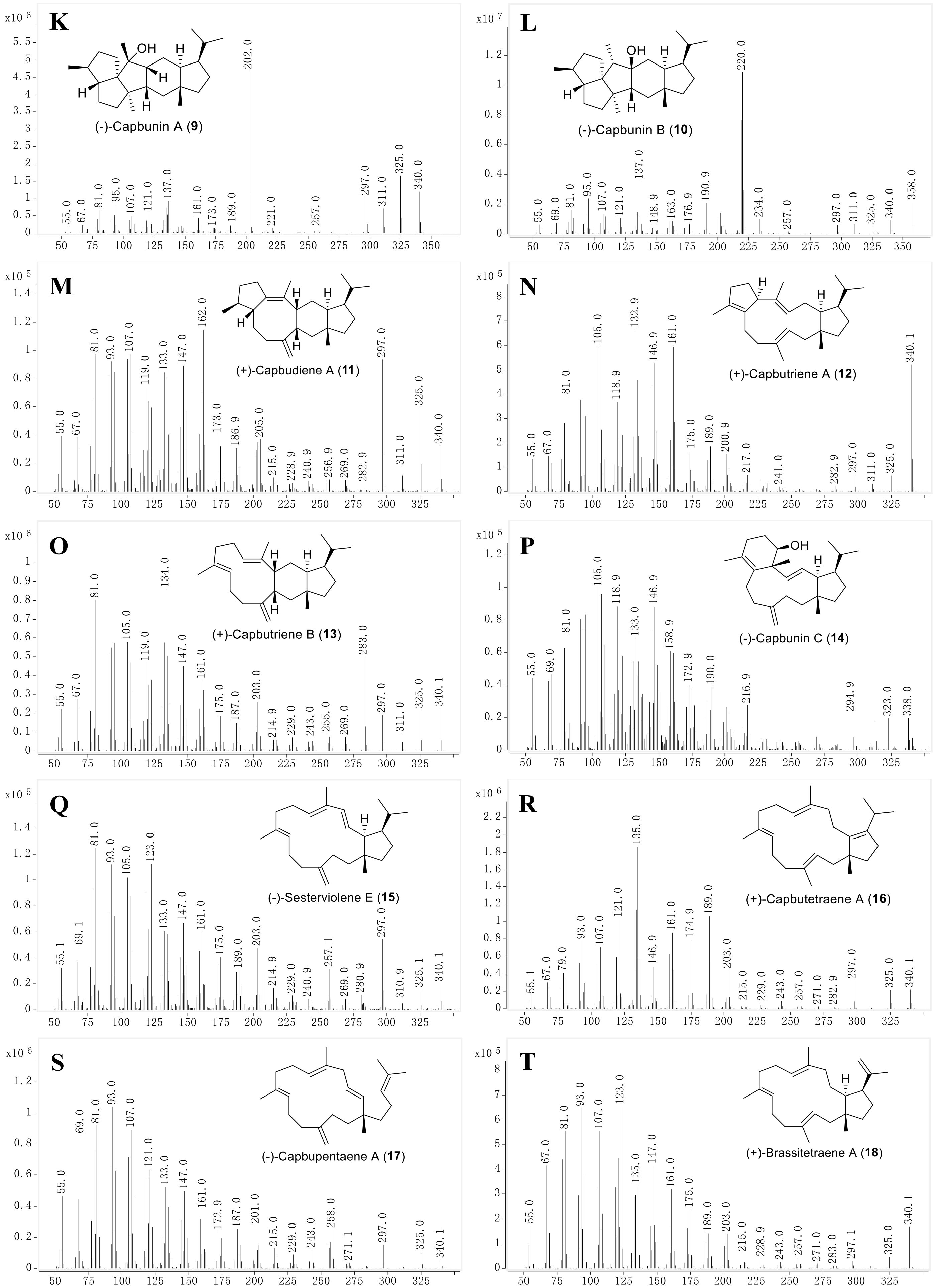

Figures S4K–T. EI-MS spectra of compounds **9–18**

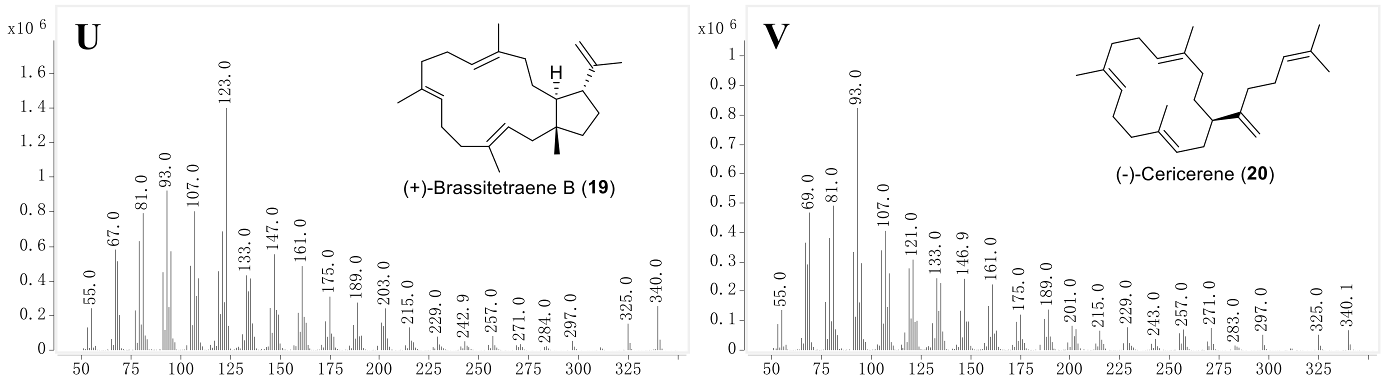

Figures S4U–V. EI-MS spectra of compounds **19** and **20**

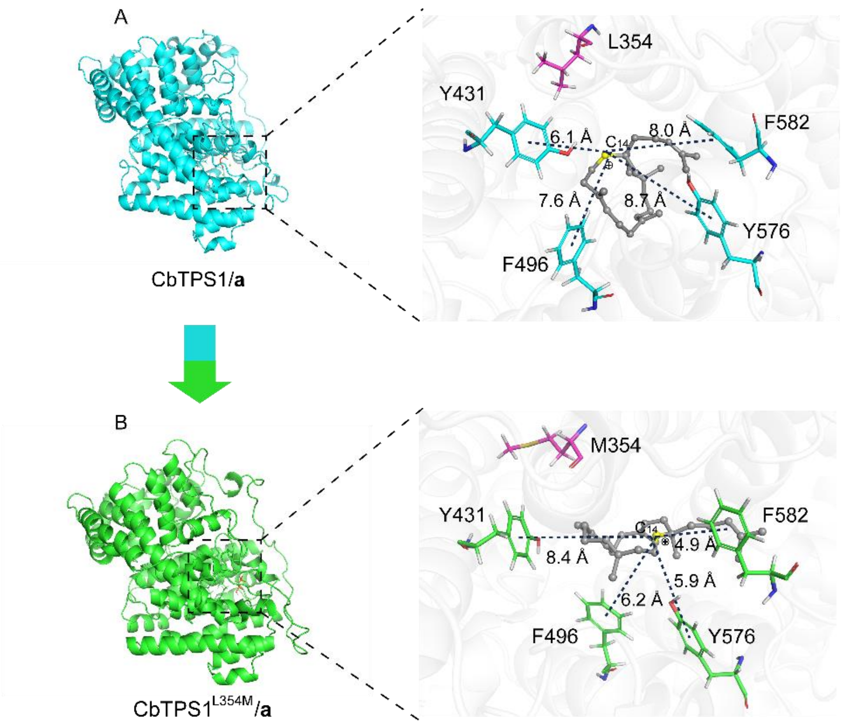

Figures S5. Representative MD snapshots of A) CbTPS1/**a** (in blue) and B) CbTPS1^{L354M}/**a** (in green) and key aromatic residues surrounding **a**

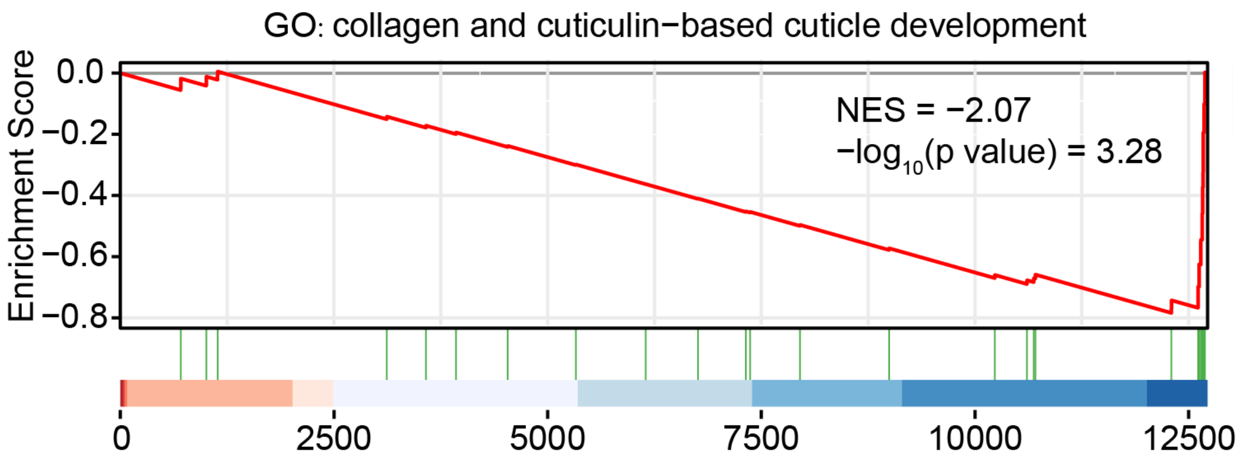

Figures S6. GSEA result of collagen and cuticulin-based cuticle development

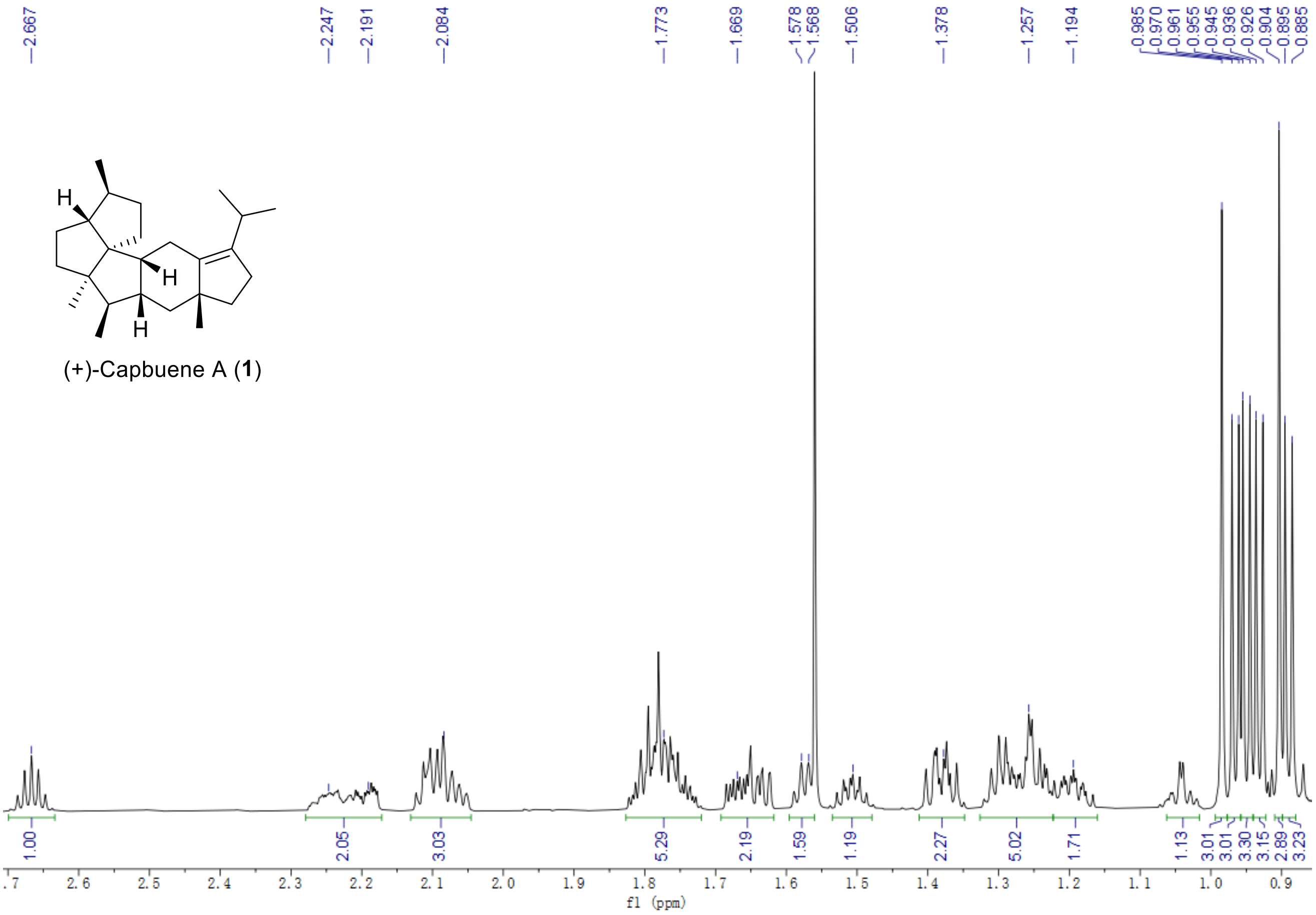

Figure S7. ^{1}H NMR spectrum of compound **1** in $CDCl_3$ (700 MHz)

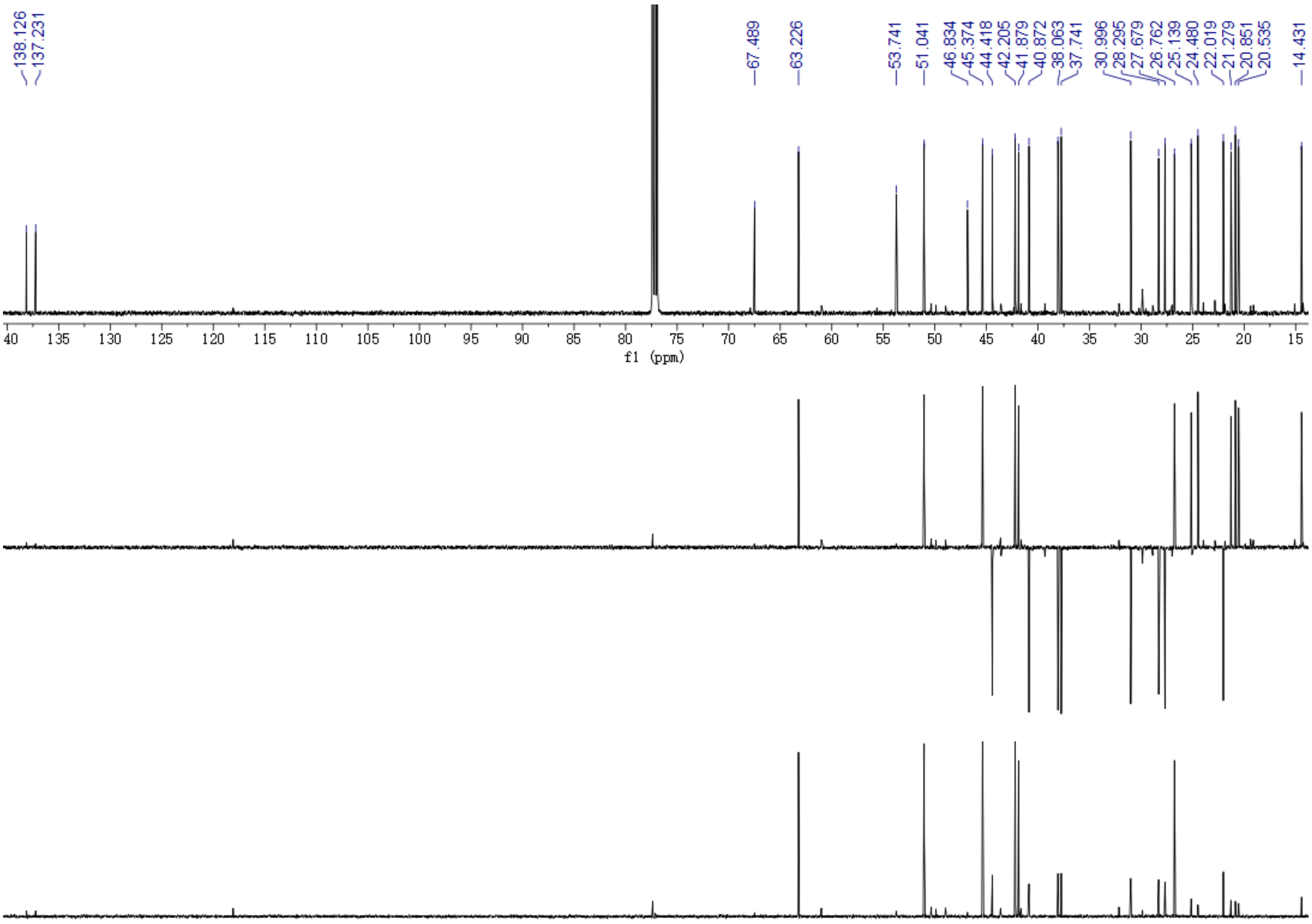

Figure S8. ^{13}C NMR and DEPT spectra of compound **1** in $CDCl_3$ (150 MHz)

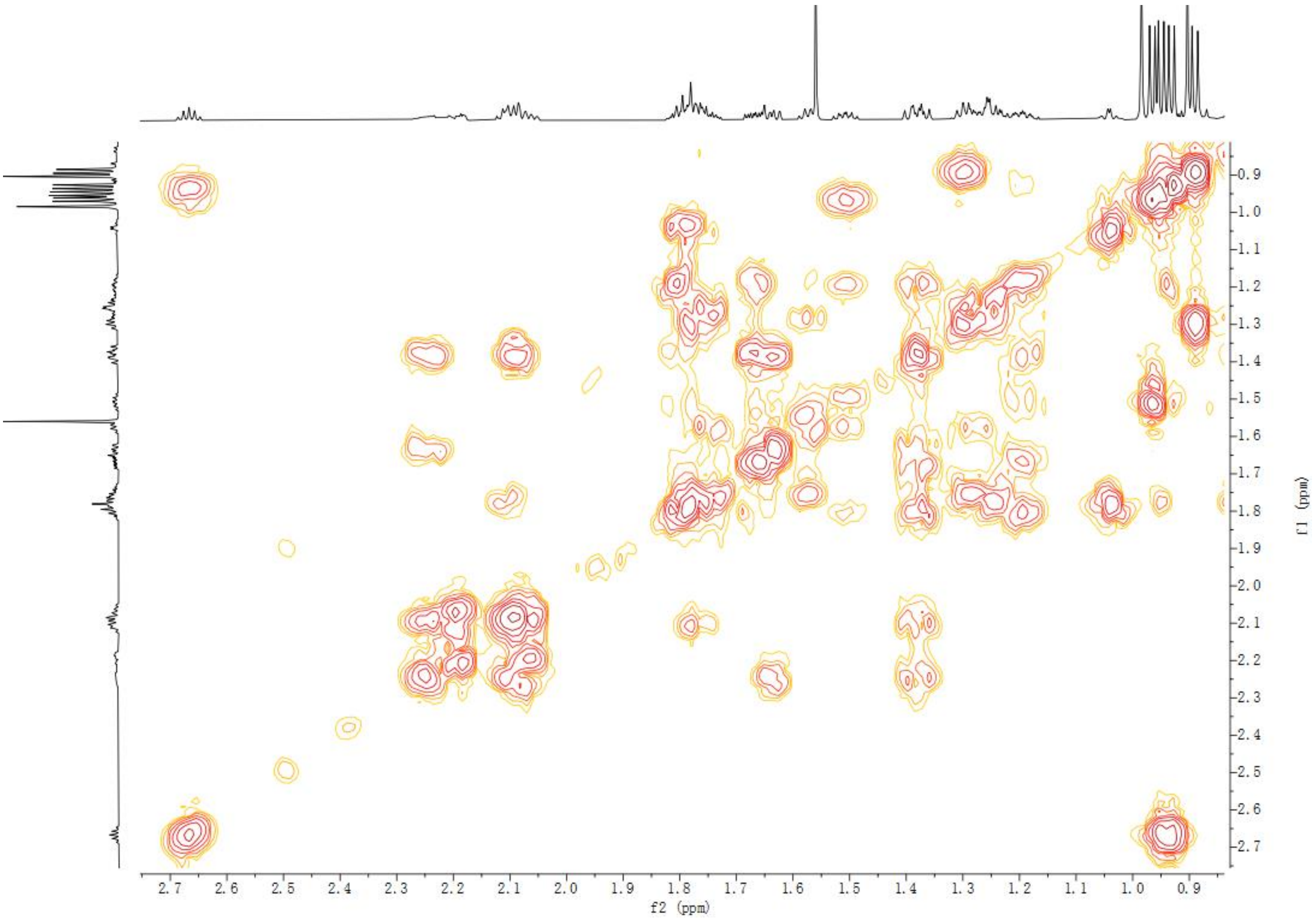

Figure S9. ^{1}H-^{1}H COSY spectrum of compound **1** in $CDCl_3$

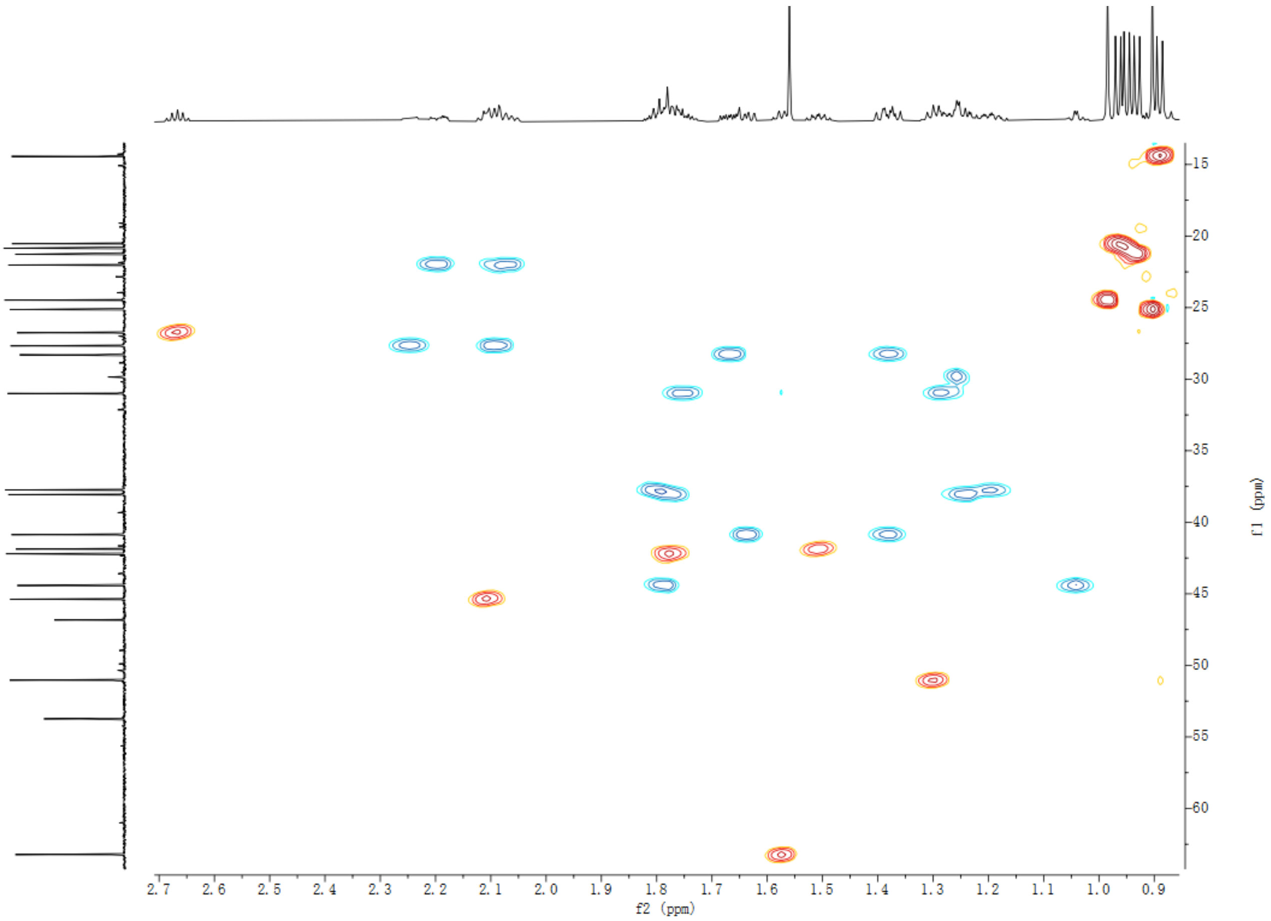

Figure S10. HSQC spectrum of compound **1** in $CDCl_3$

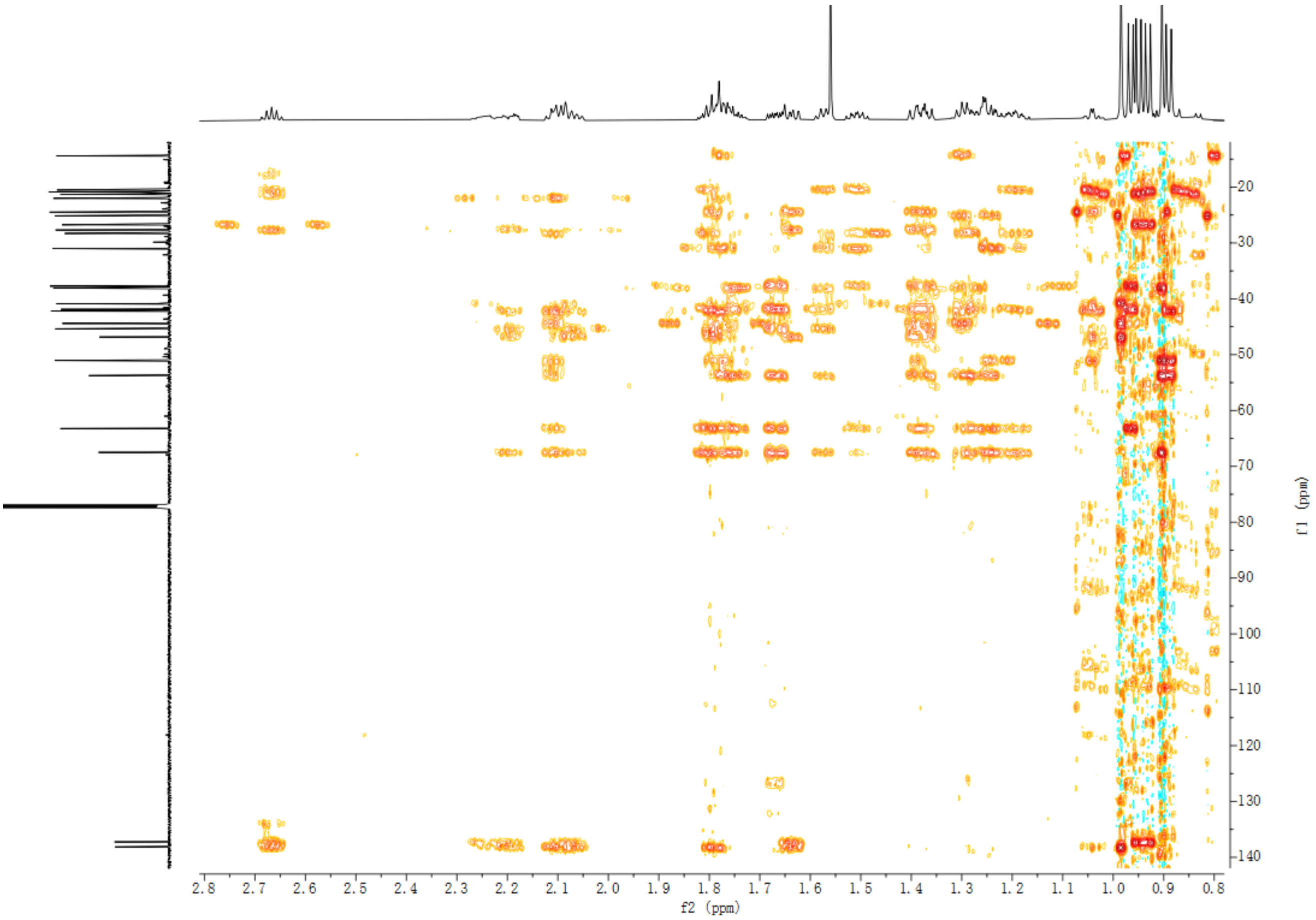

Figure S11. HMBC spectrum of compound **1** in $CDCl_3$

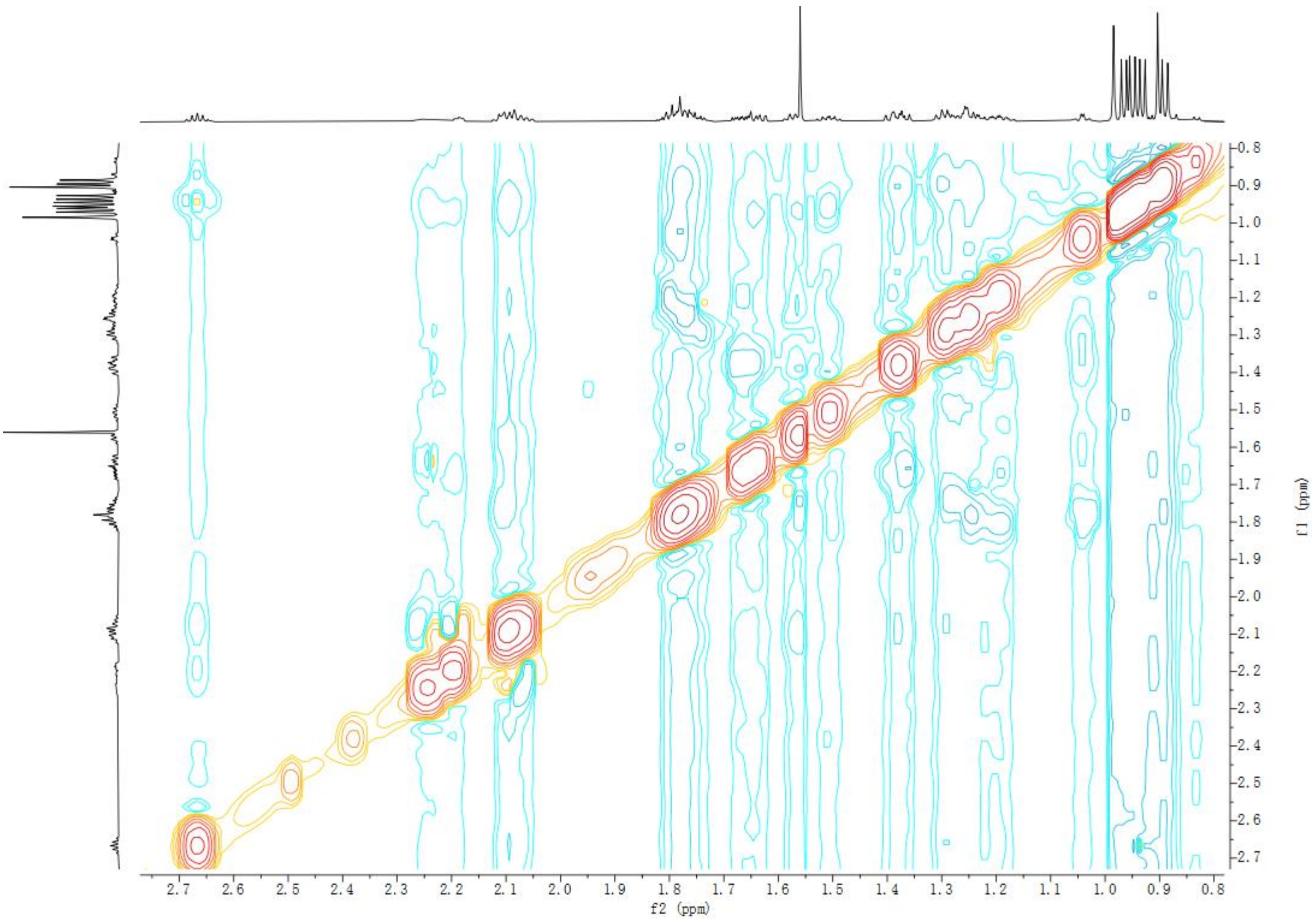

Figure S12. NOESY spectrum of compound **1** in $CDCl_3$

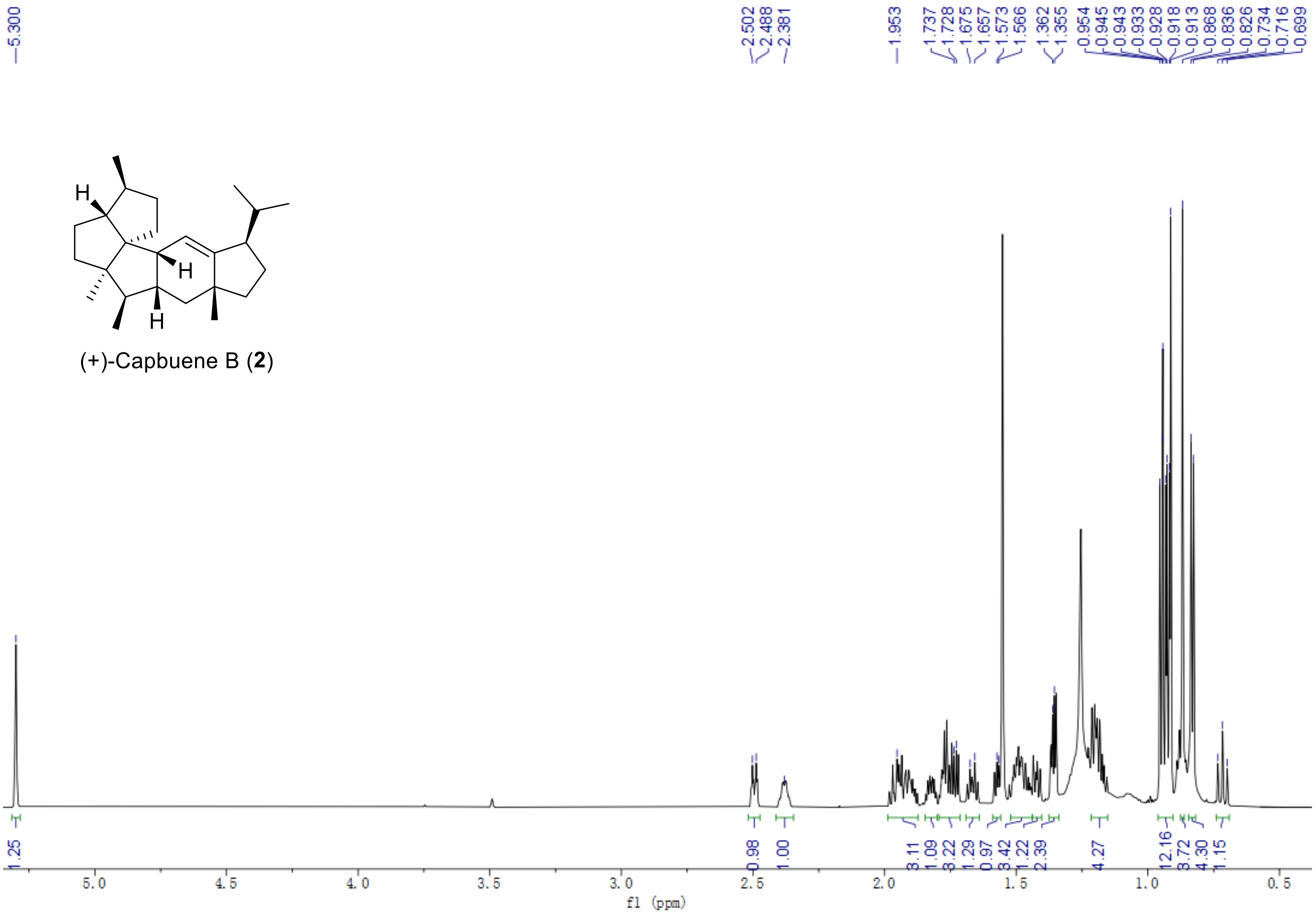

Figure S13. ^{1}H NMR spectrum of compound **2** in $CDCl_3$ (700 MHz)

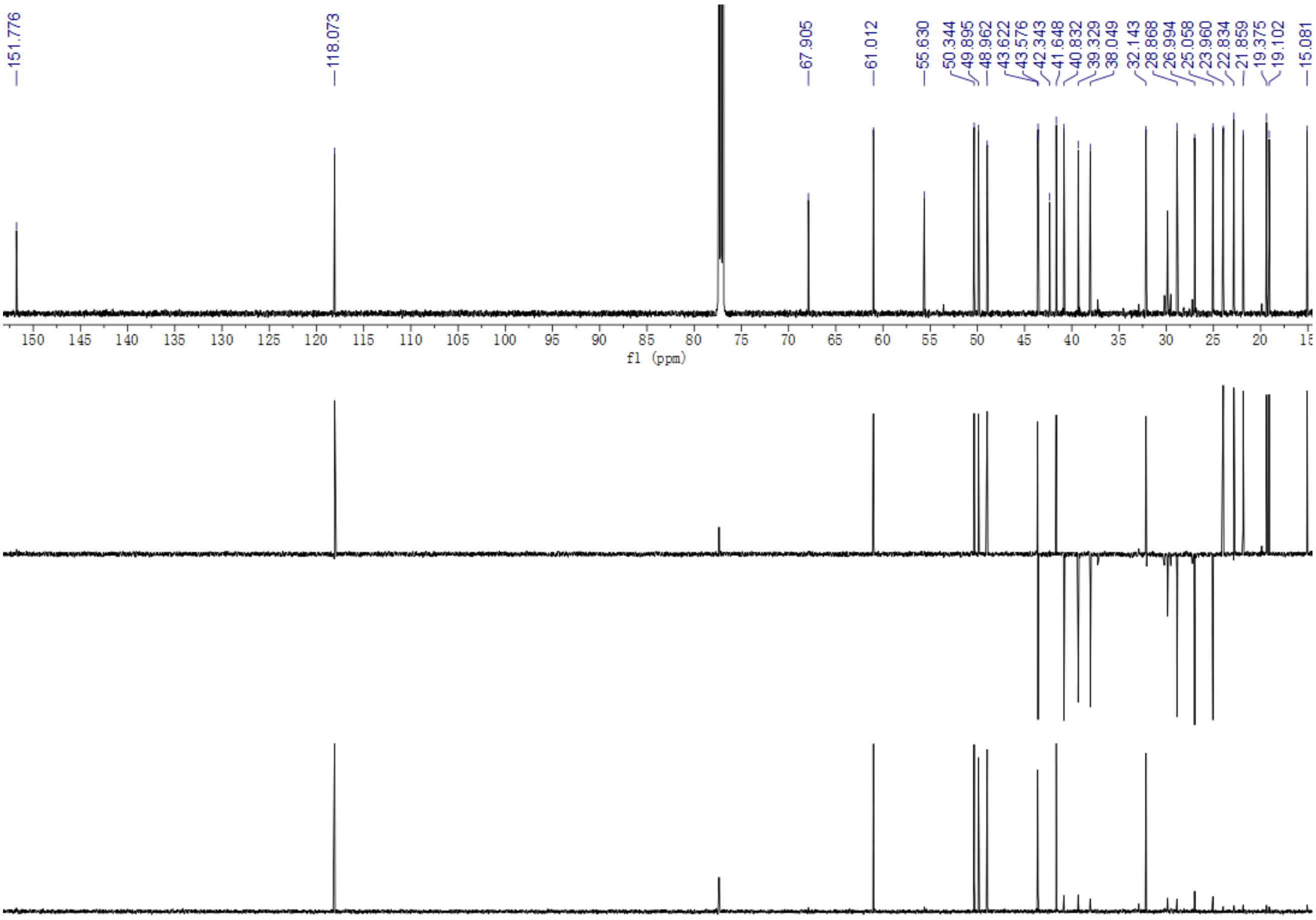

Figure S14. ^{13}C NMR and DEPT spectra of compound **2** in $CDCl_3$ (150 MHz)

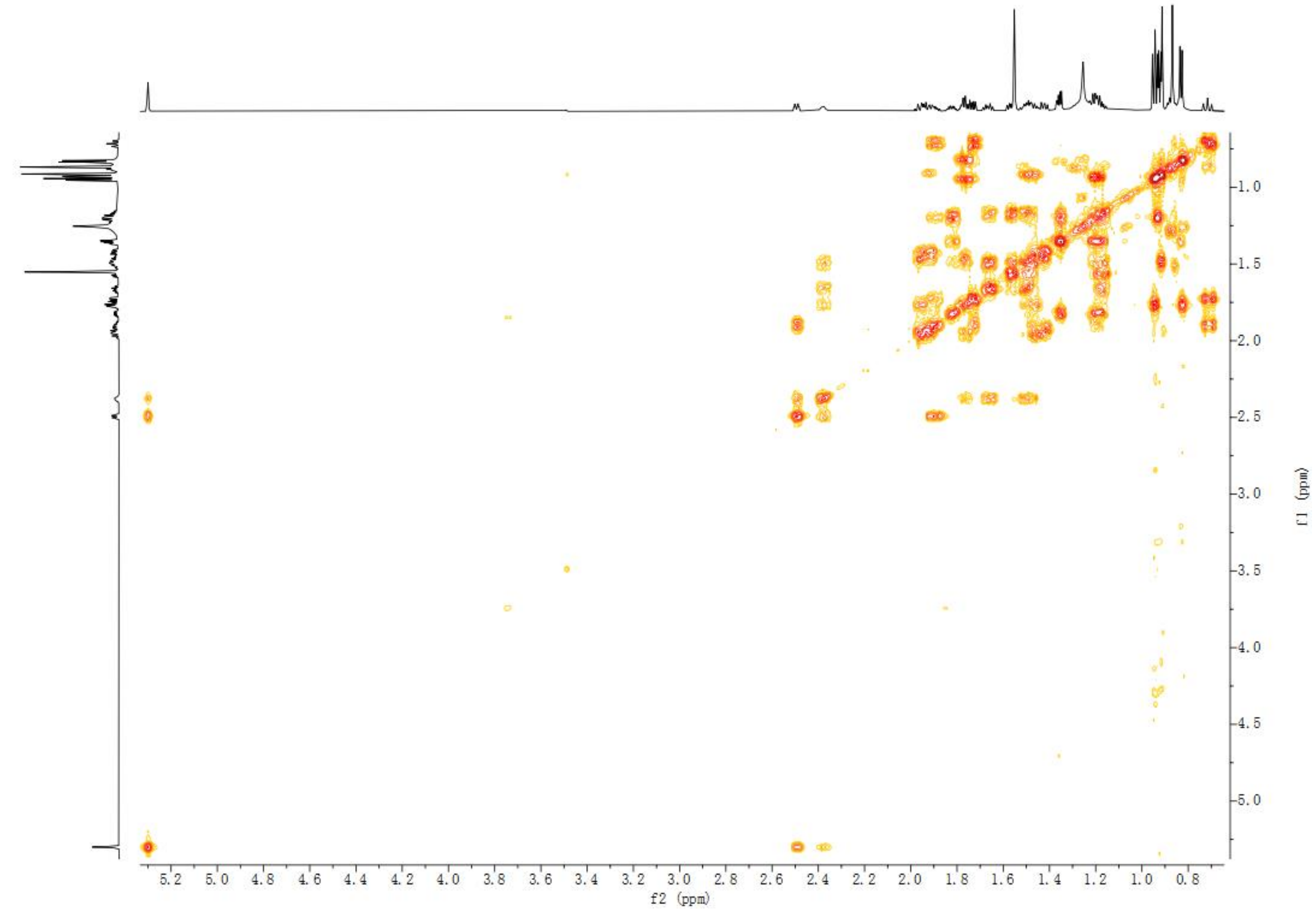

Figure S15. ^{1}H-^{1}H COSY spectrum of compound **2** in $CDCl_3$

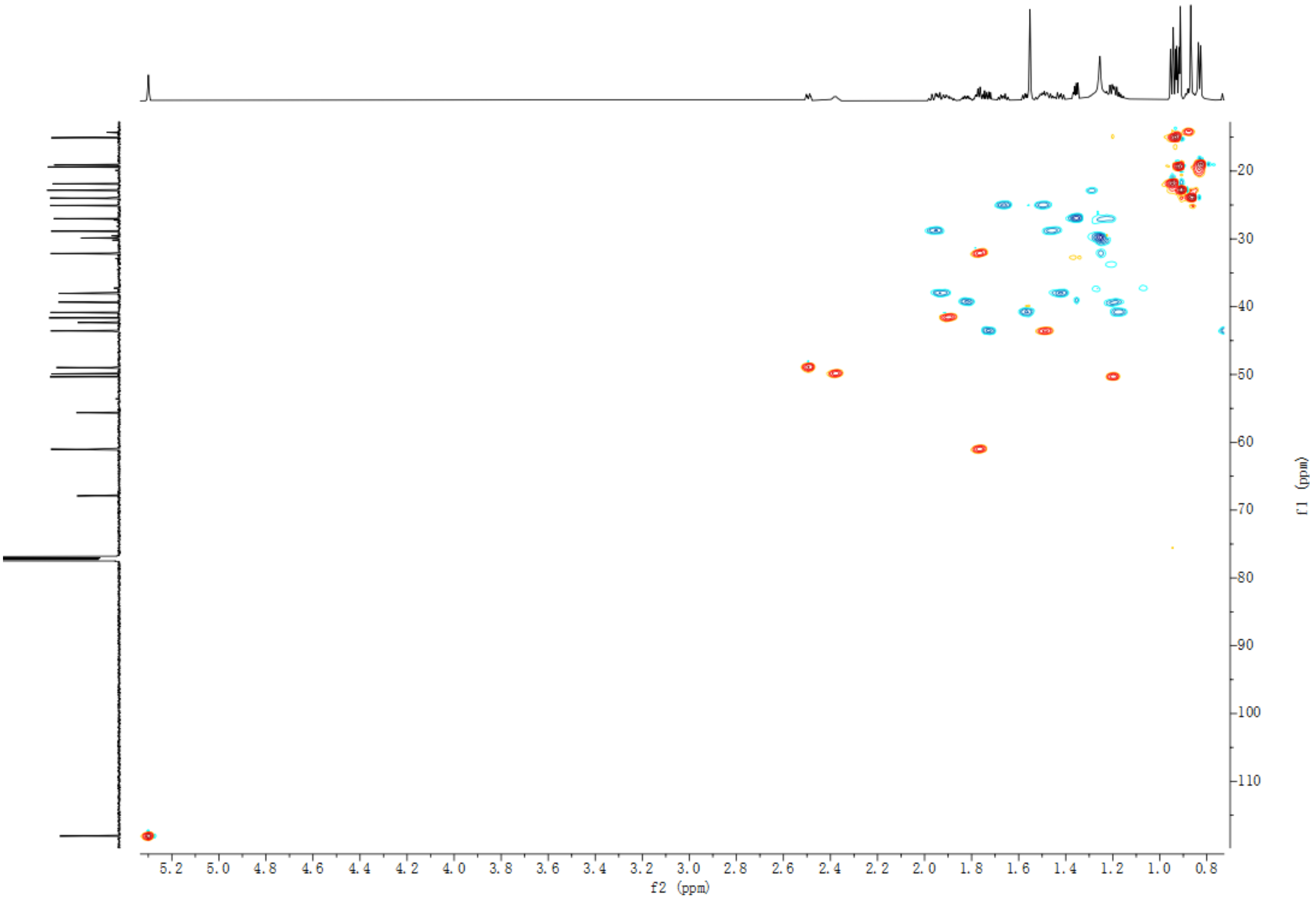

Figure S16. HSQC spectrum of compound **2** in $CDCl_3$

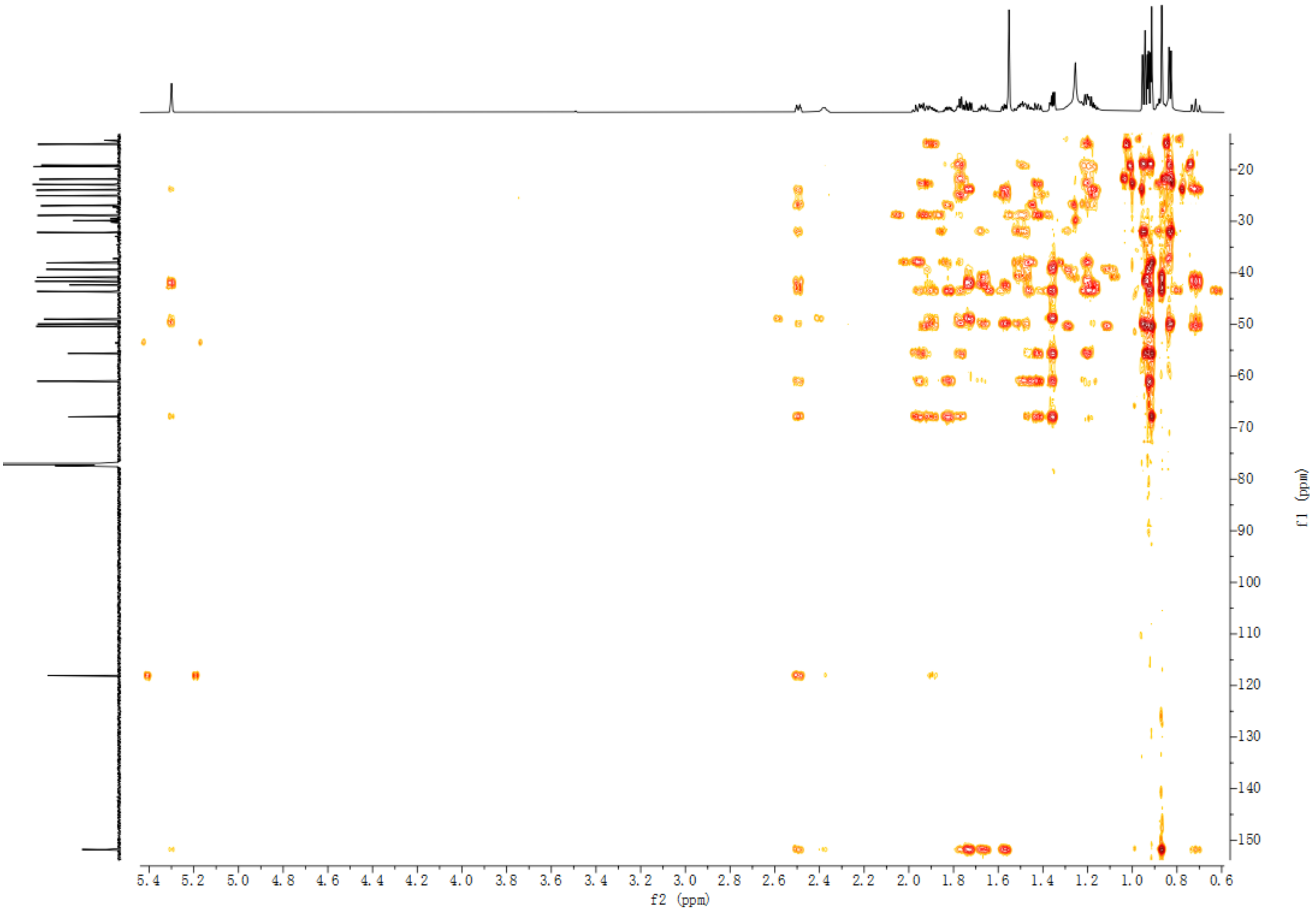

Figure S17. HMBC spectrum of compound **2** in $CDCl_3$

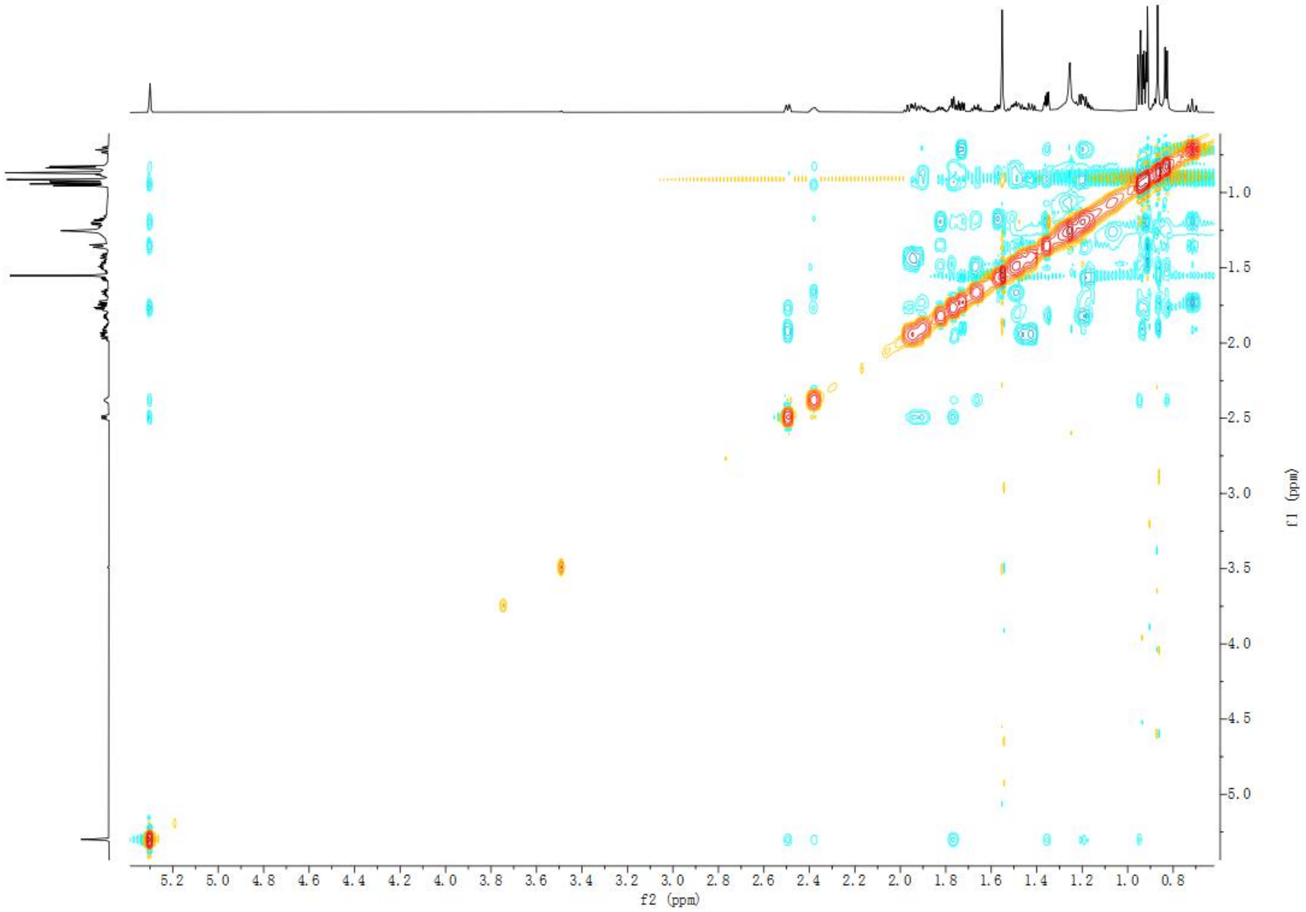

Figure S18. NOESY spectrum of compound **2** in $CDCl_3$

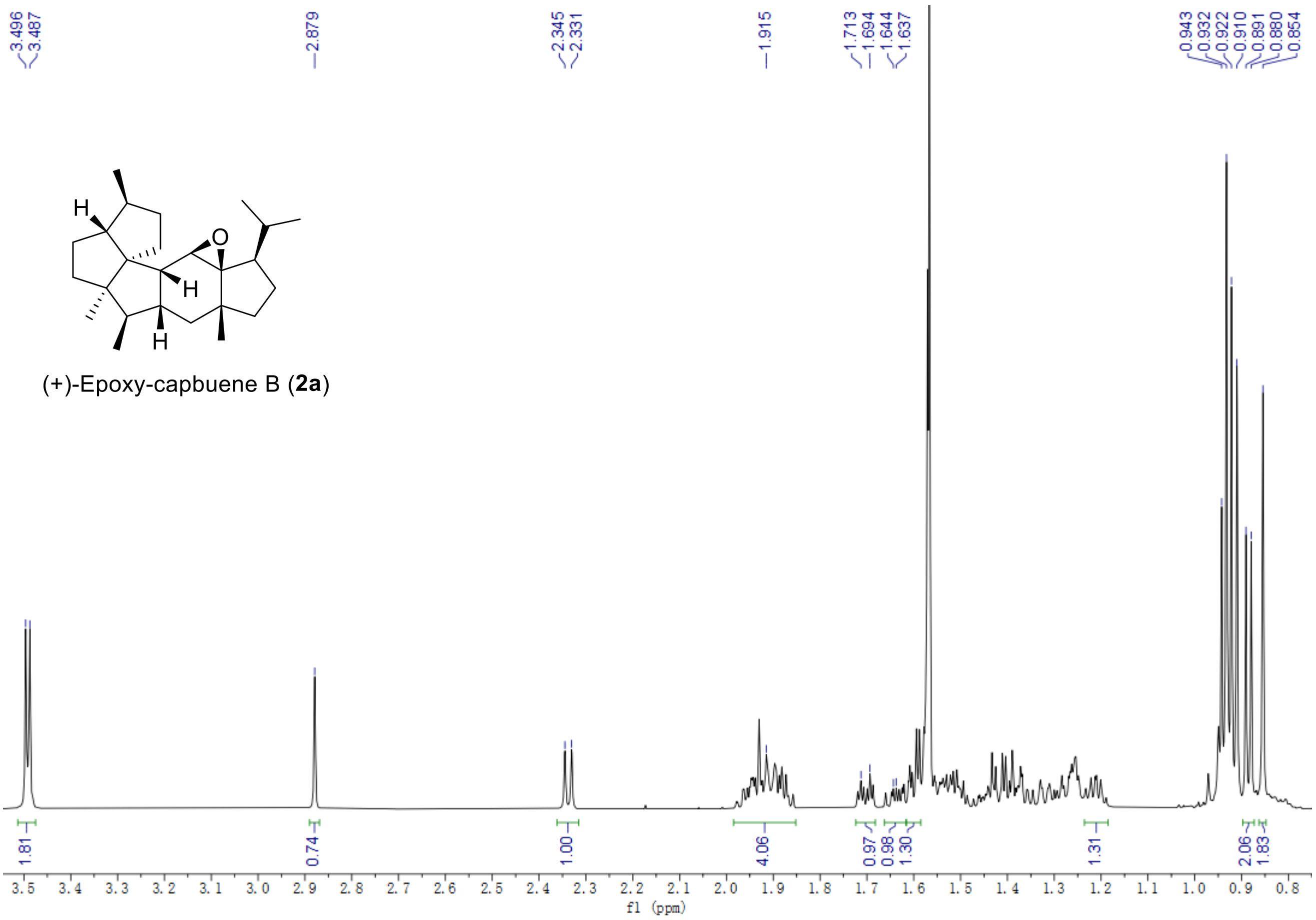

Figure S19. ^{1}H NMR spectrum of compound **2a** in $CDCl_3$ (700 MHz)

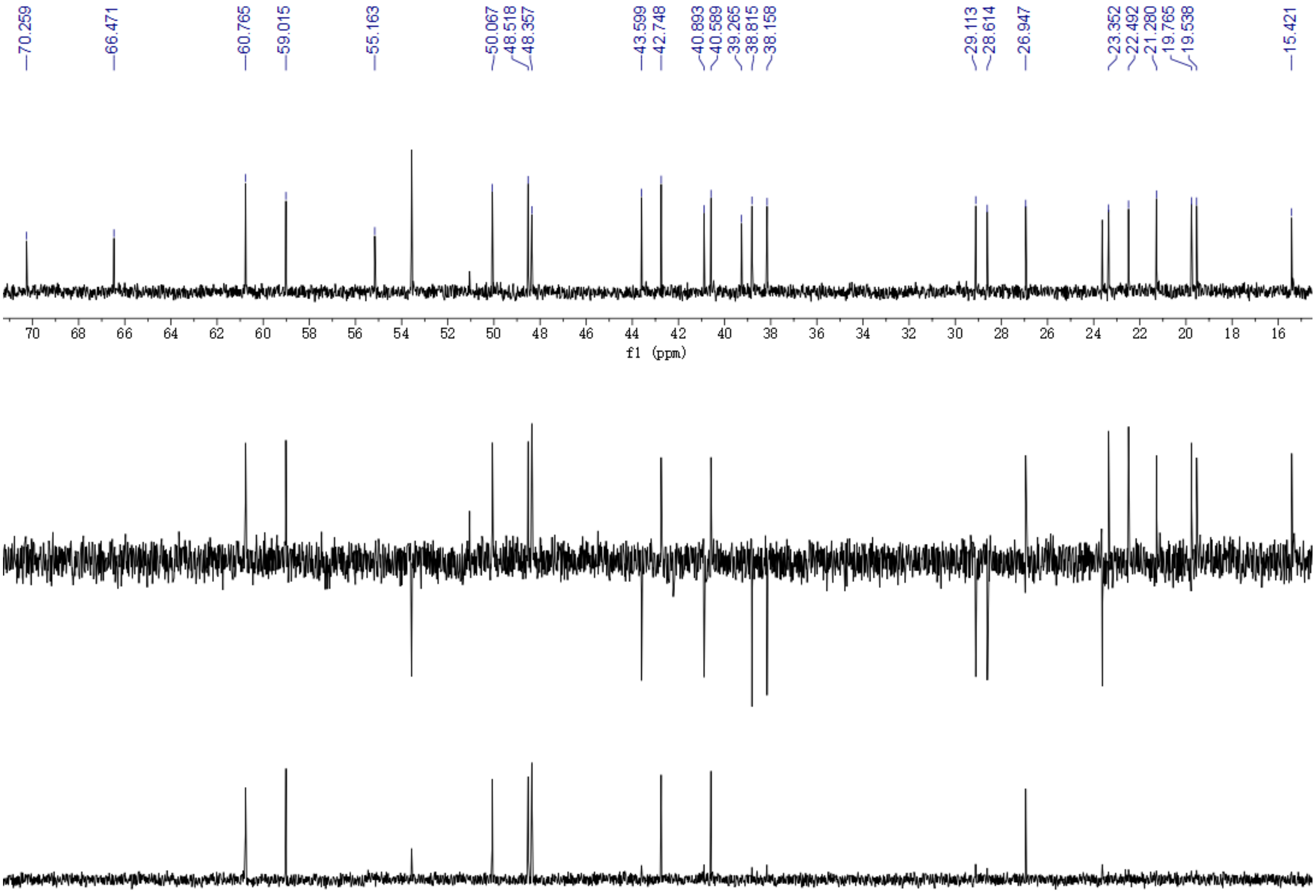

Figure S20. ^{13}C NMR and DEPT spectra of compound **2a** in $CDCl_3$ (150 MHz)

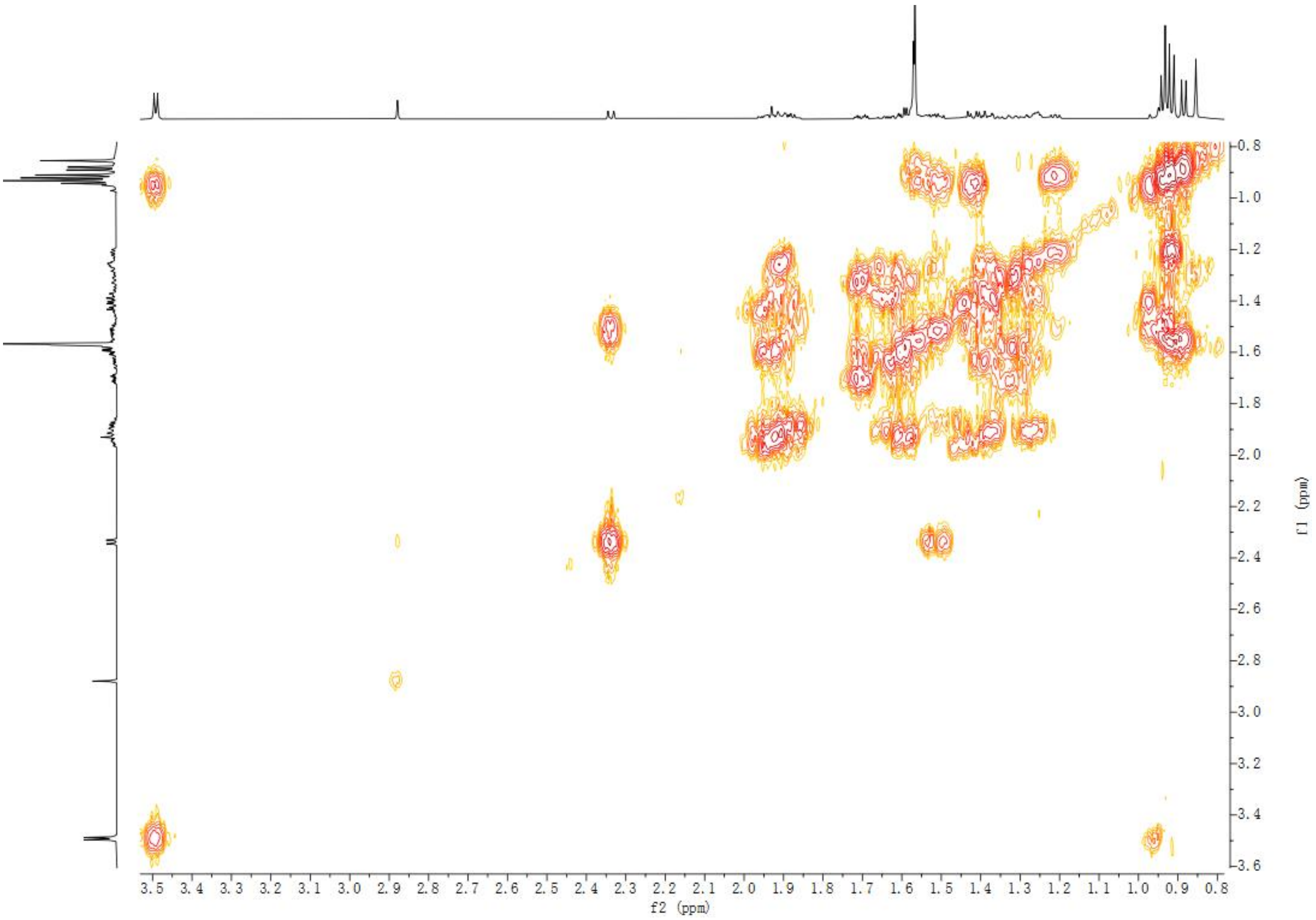

Figure S21. ^{1}H-^{1}H COSY spectrum of compound **2a** in $CDCl_3$

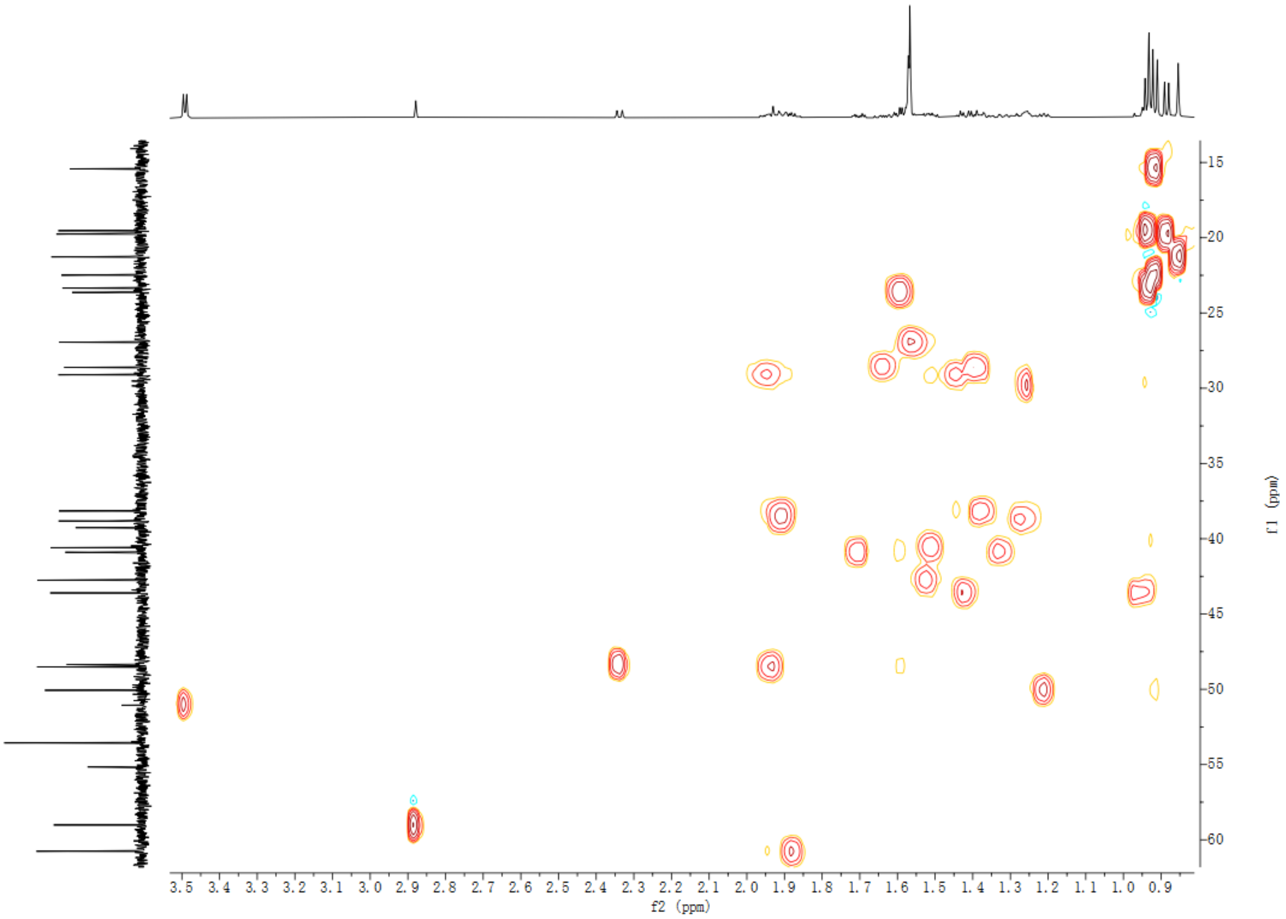

Figure S22. HSQC spectrum of compound **2a** in $CDCl_3$

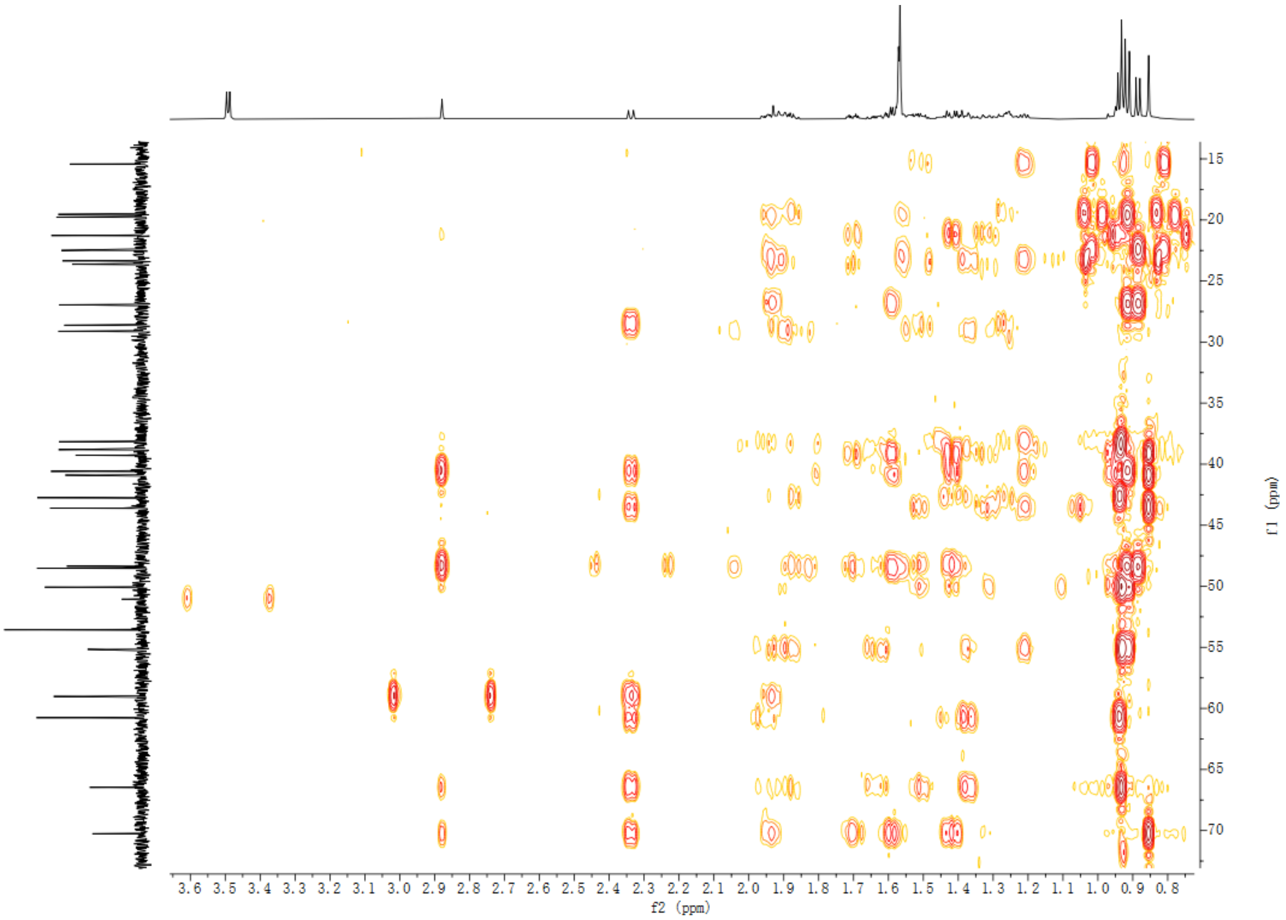

Figure S23. HMBC spectrum of compound **2a** in $CDCl_3$

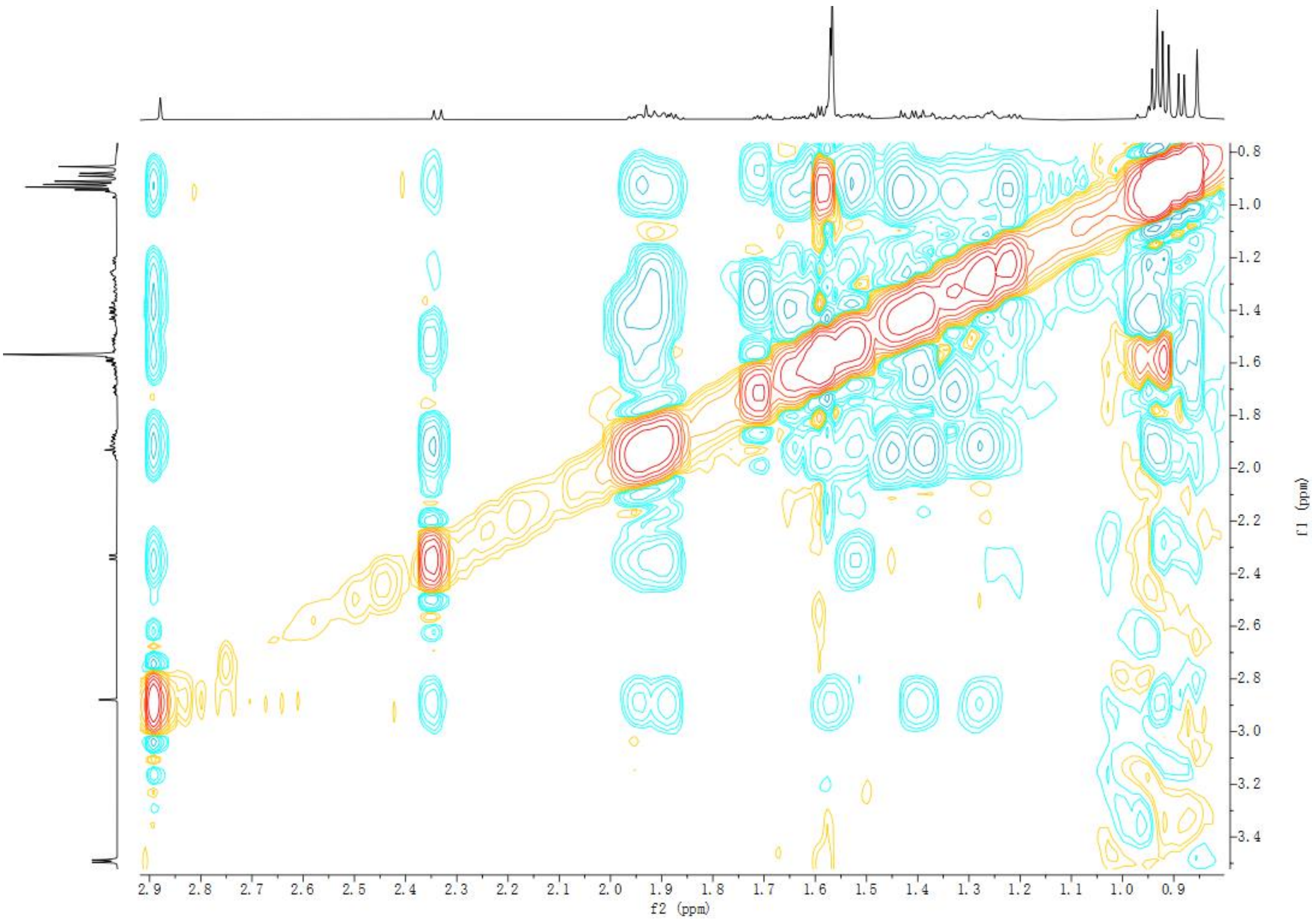

Figure S24. NOESY spectrum of compound **2a** in $CDCl_3$

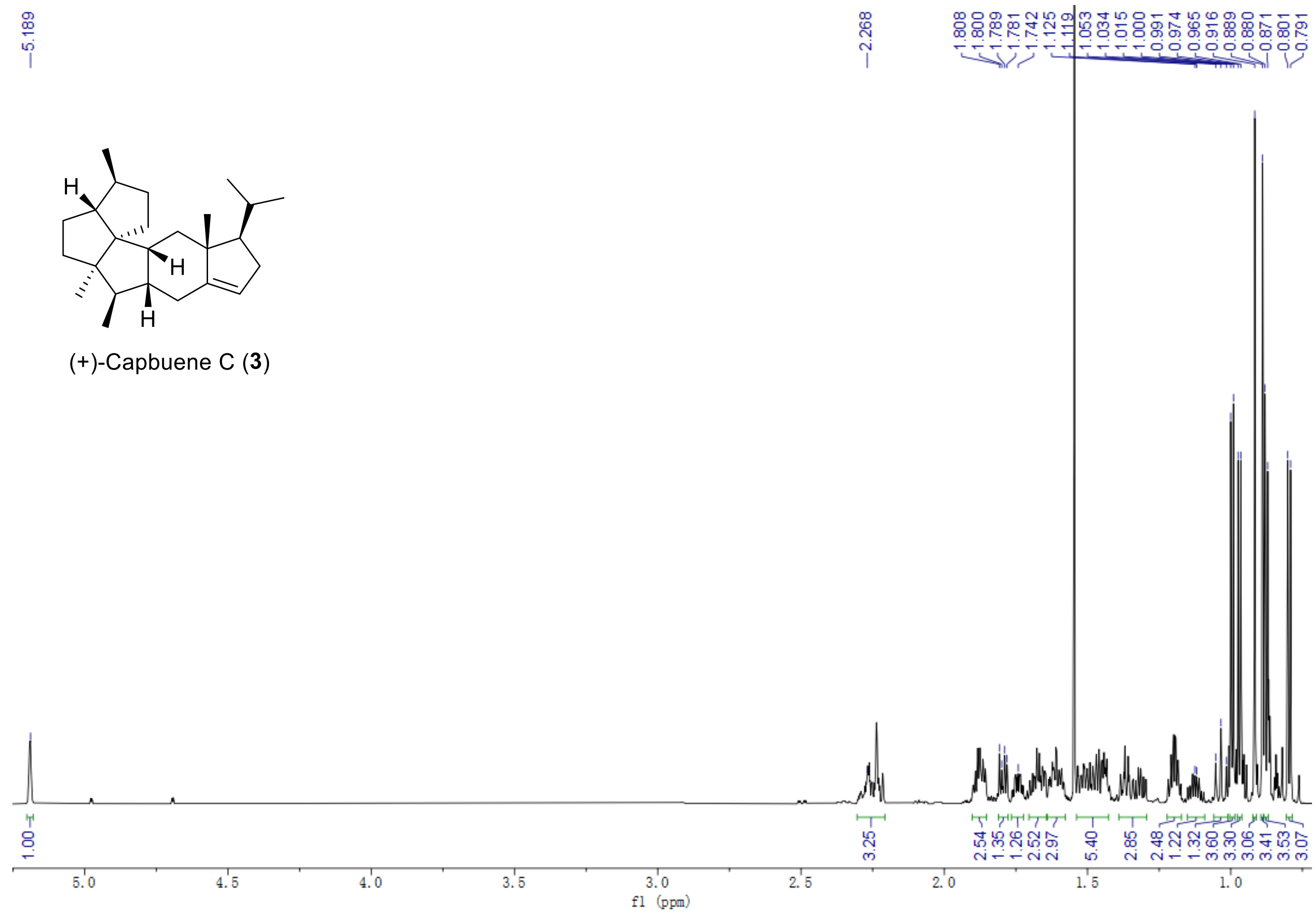

Figure S25. ^{1}H NMR spectrum of compound **3** in $CDCl_3$ (700 MHz)

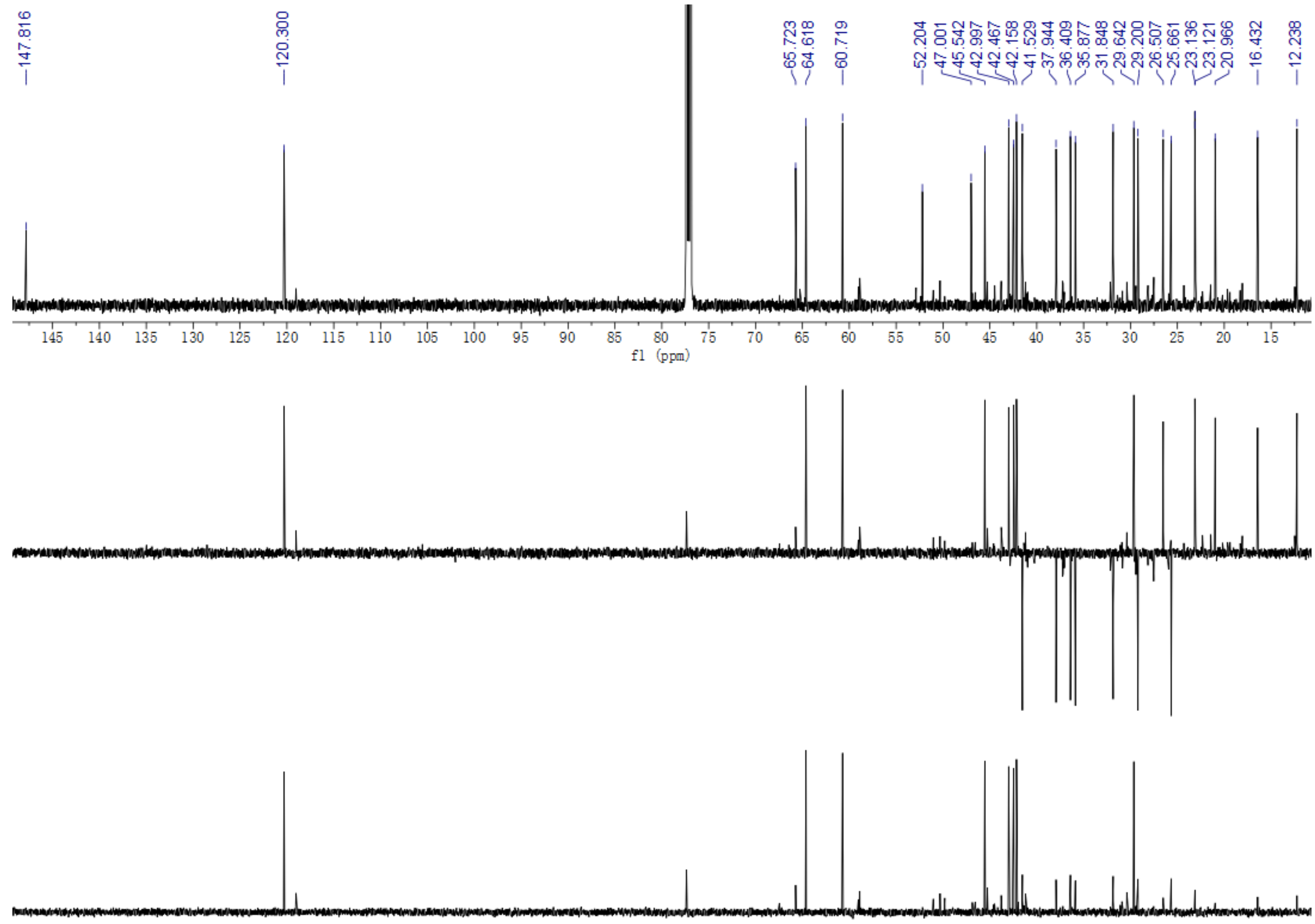

Figure S26. ^{13}C NMR and DEPT spectra of compound **3** in $CDCl_3$ (150 MHz)

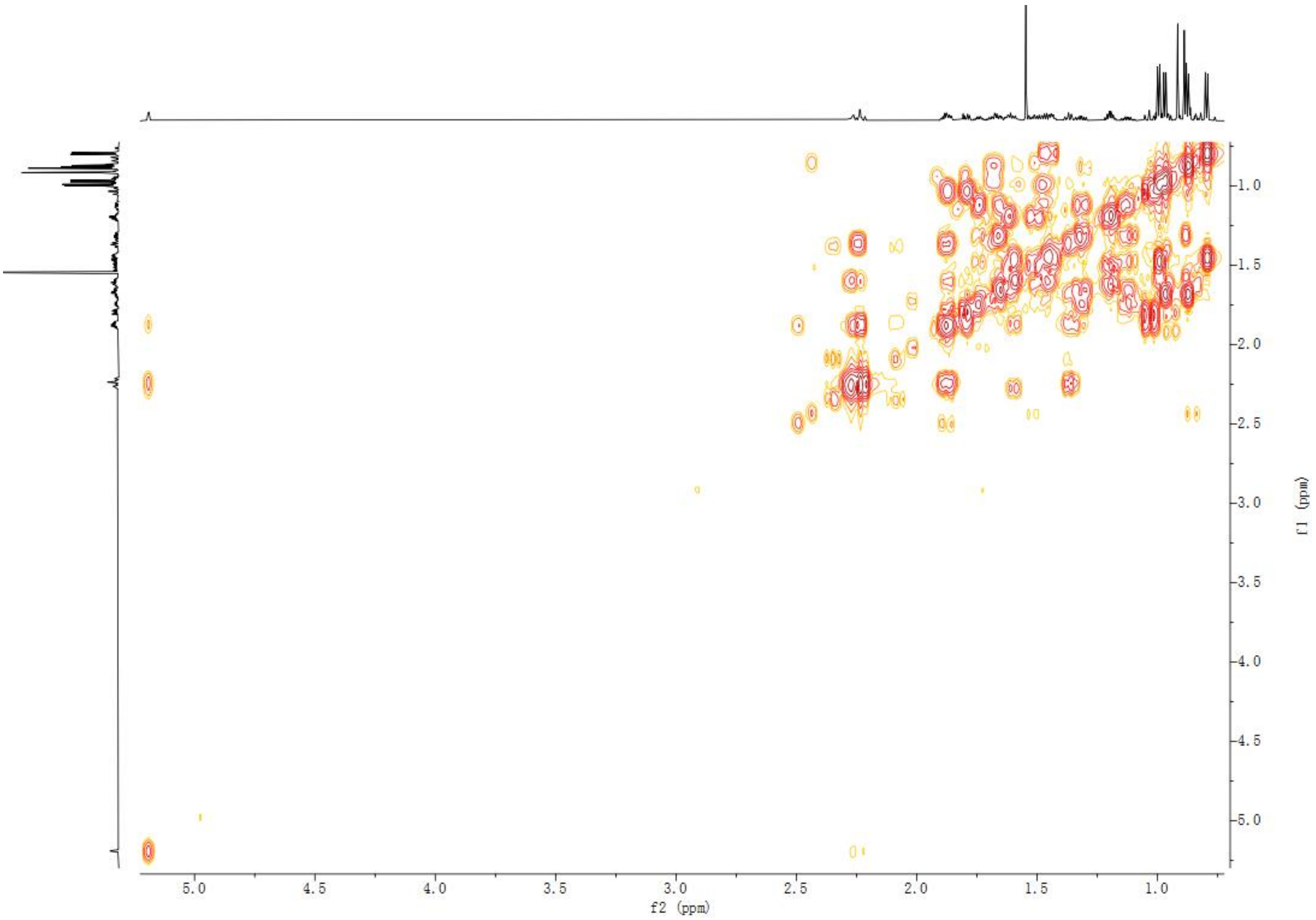

Figure S27. ^{1}H-^{1}H COSY spectrum of compound **3** in $CDCl_3$

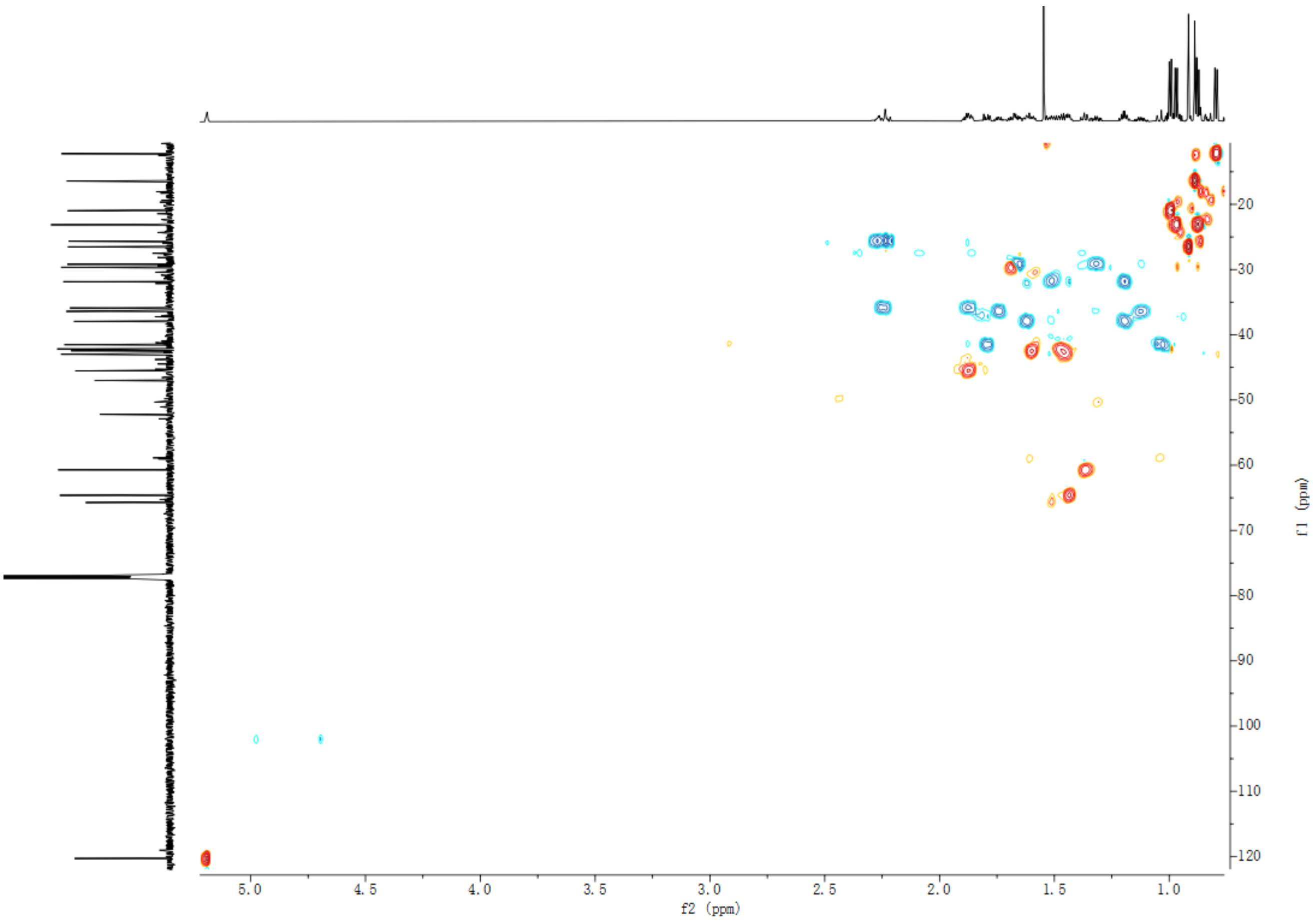

Figure S28. HSQC spectrum of compound **3** in $CDCl_3$

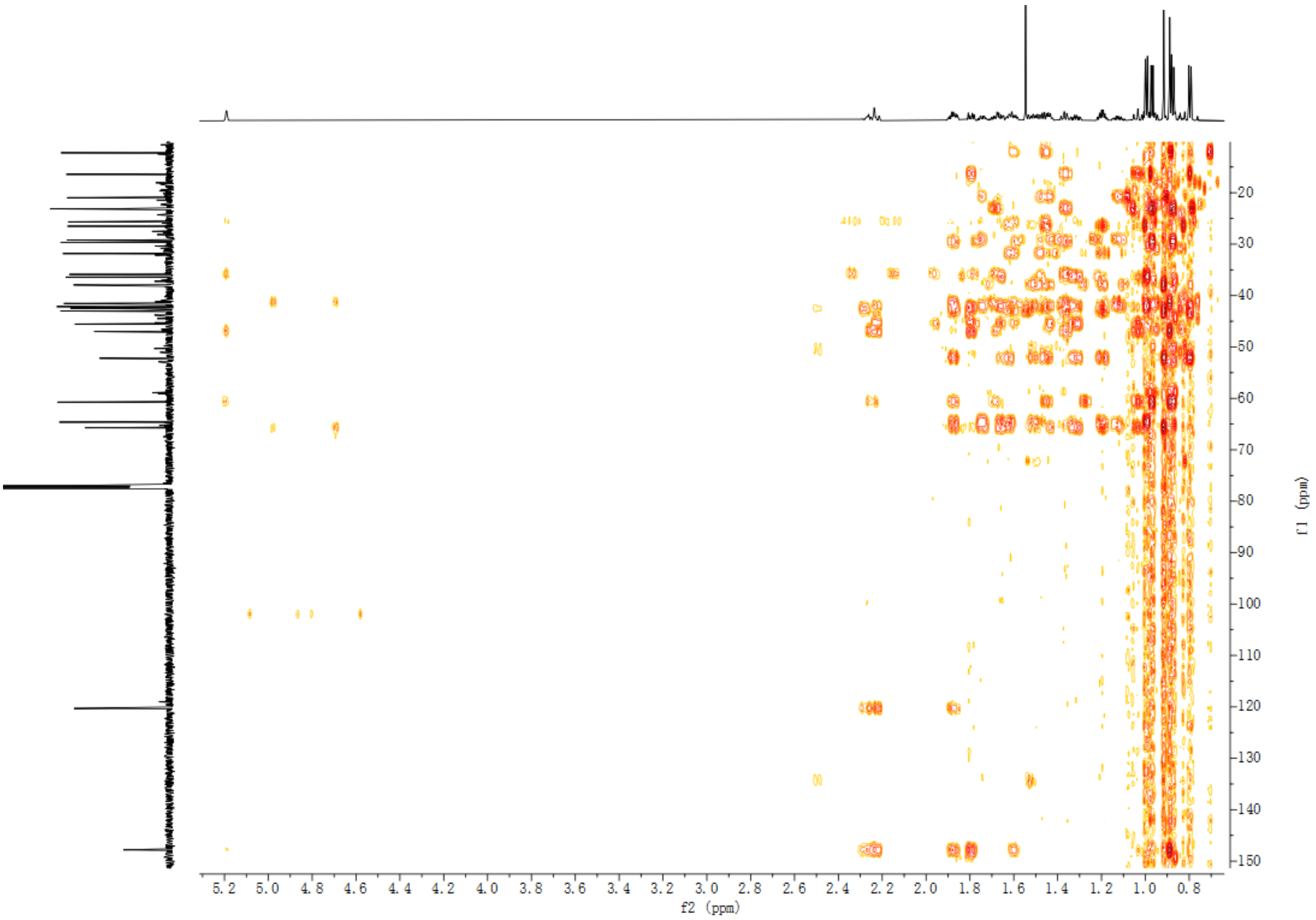

Figure S29. HMBC spectrum of compound **3** in $CDCl_3$

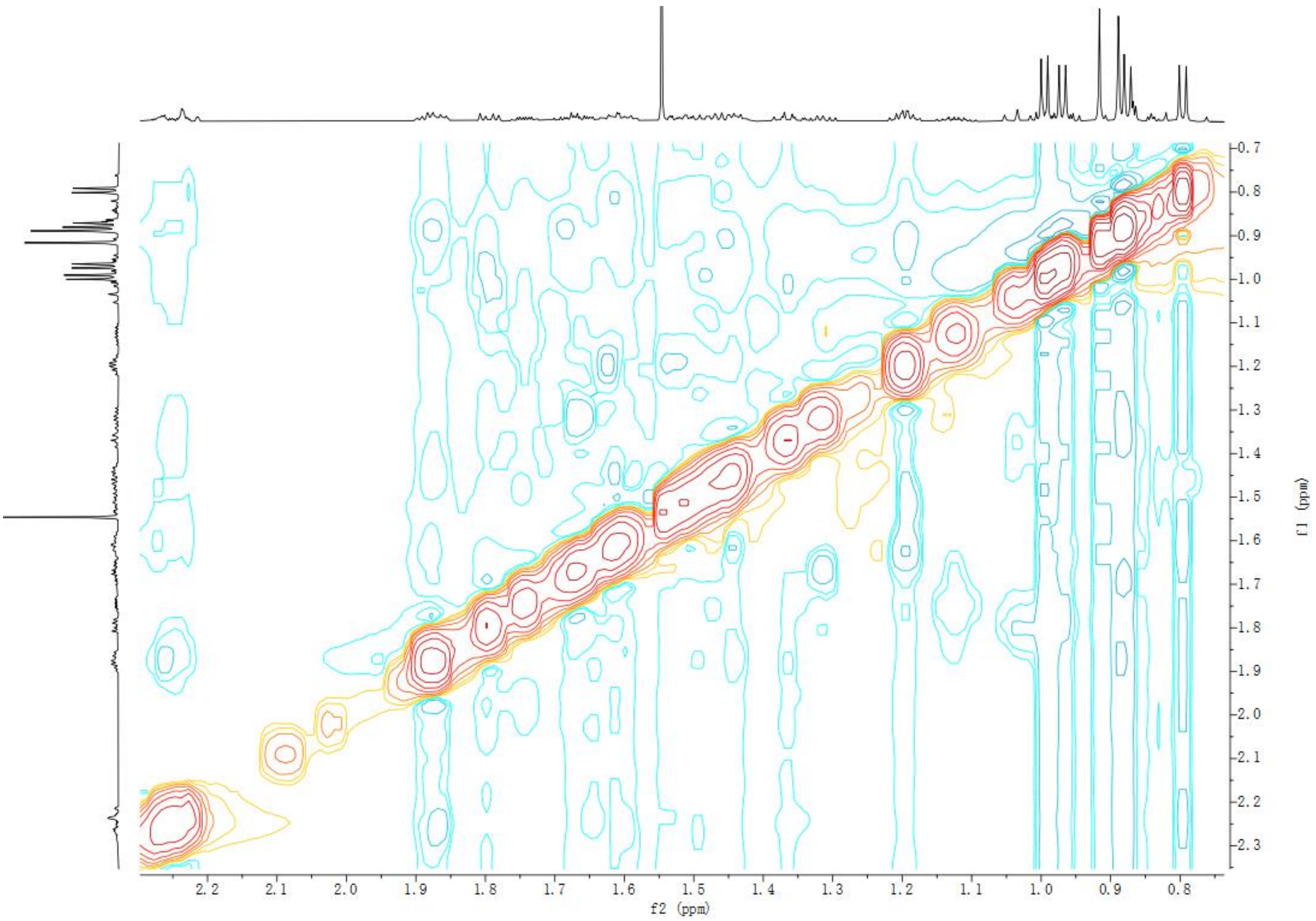

Figure S30. NOESY spectrum of compound **3** in $CDCl_3$

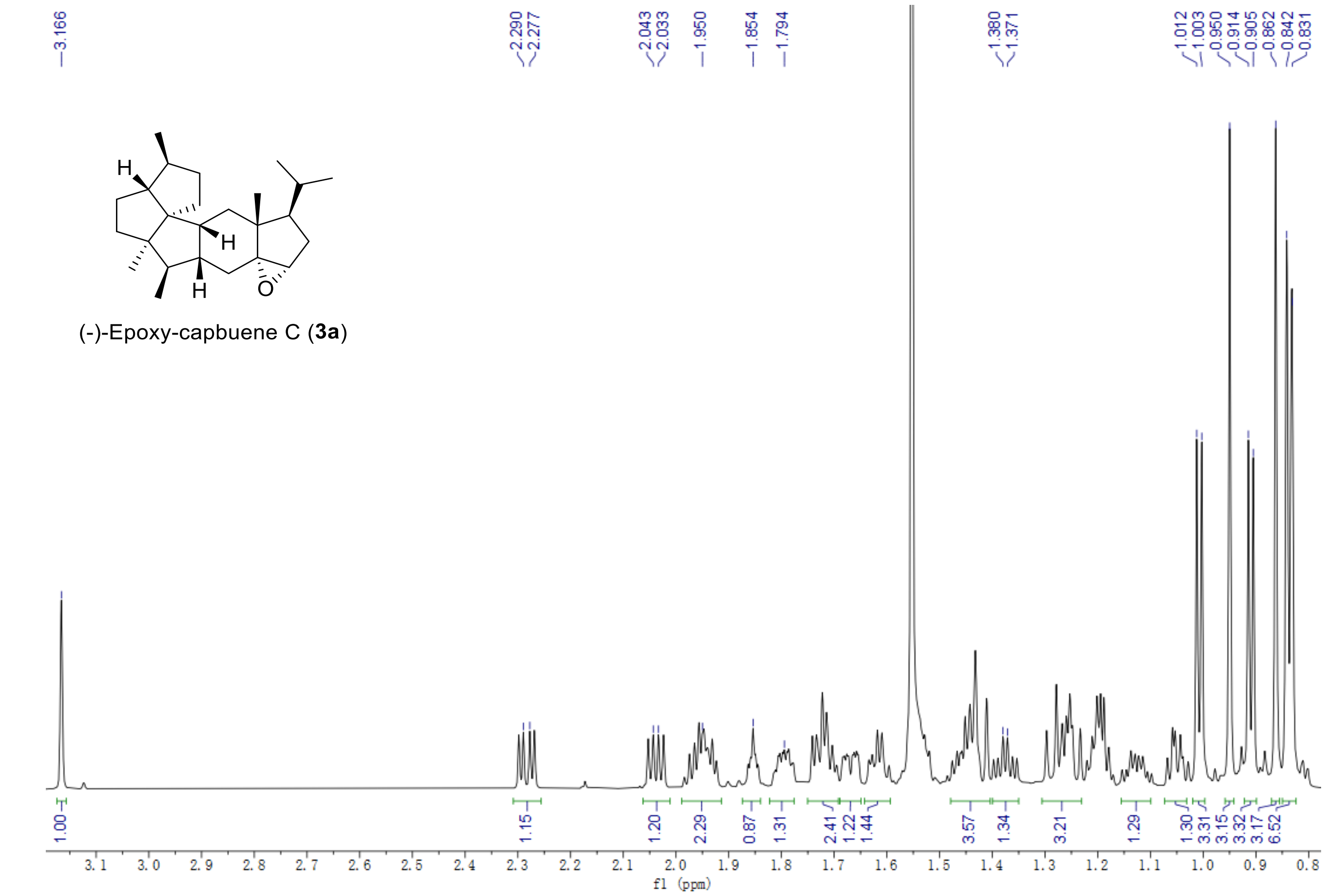

Figure S31. ^{1}H NMR spectrum of compound **3a** in $CDCl_3$ (700 MHz)

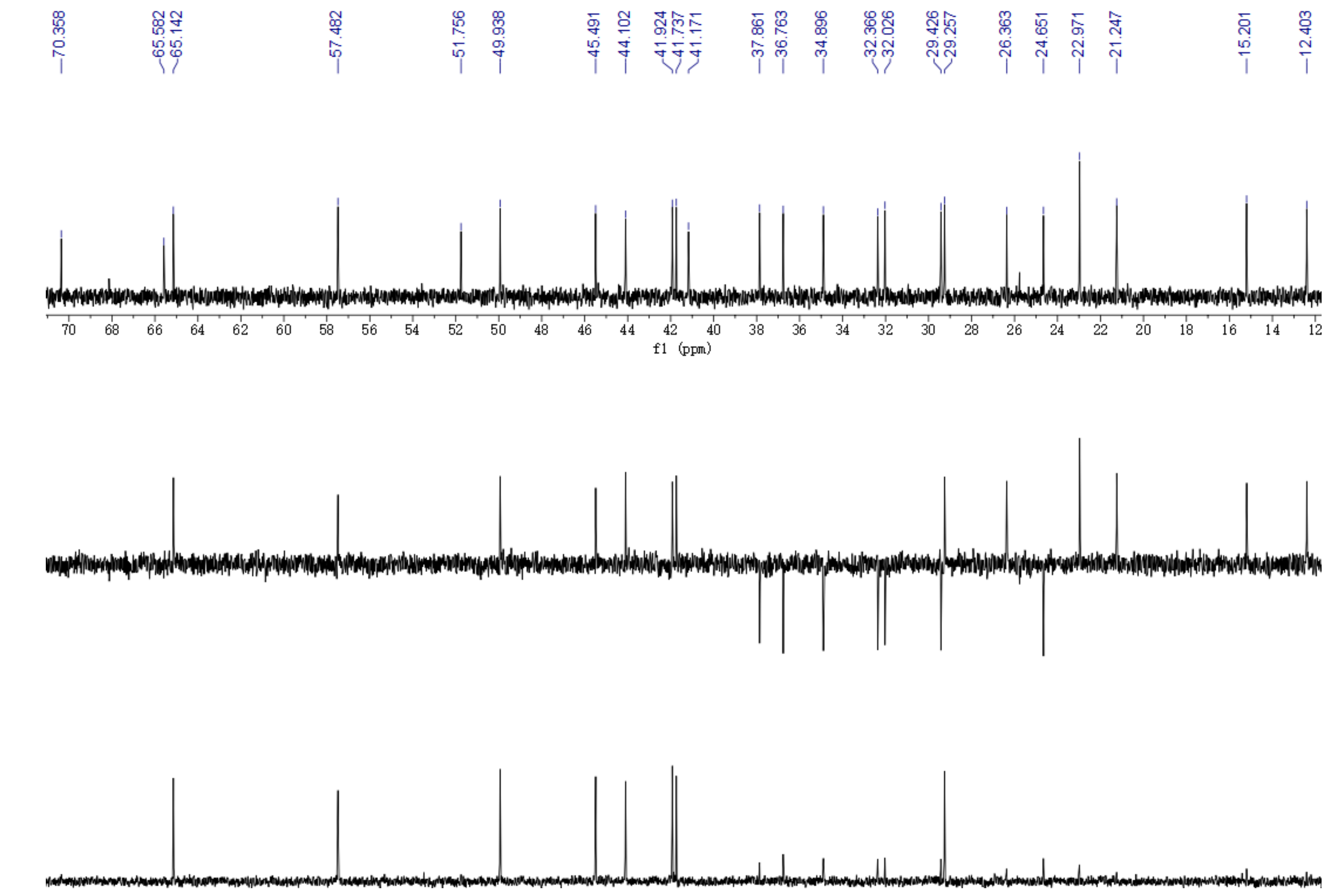

Figure S32. ^{13}C NMR and DEPT spectra of compound **3a** in $CDCl_3$ (150 MHz)

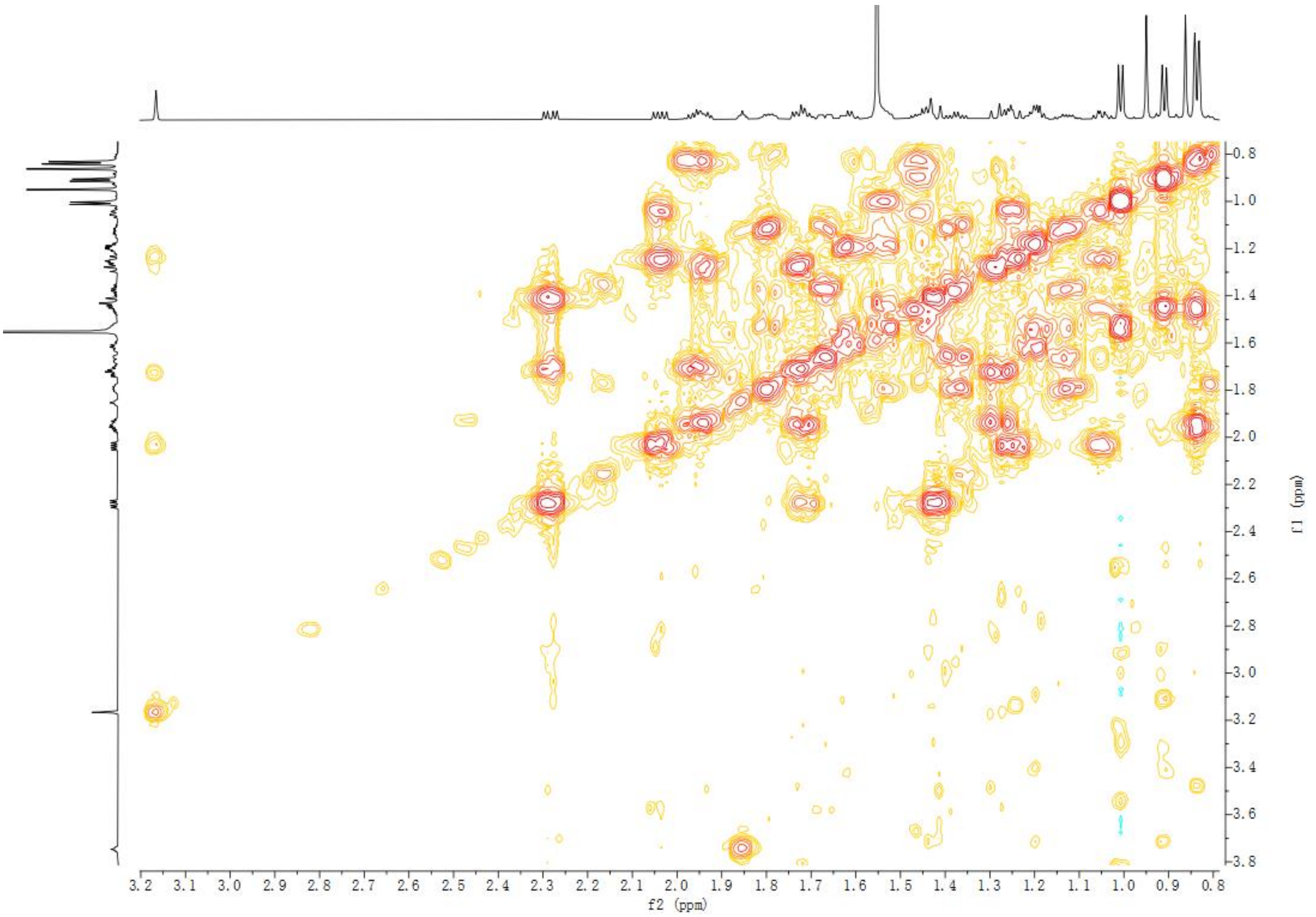

Figure S33. ^{1}H-^{1}H COSY spectrum of compound **3a** in $CDCl_3$

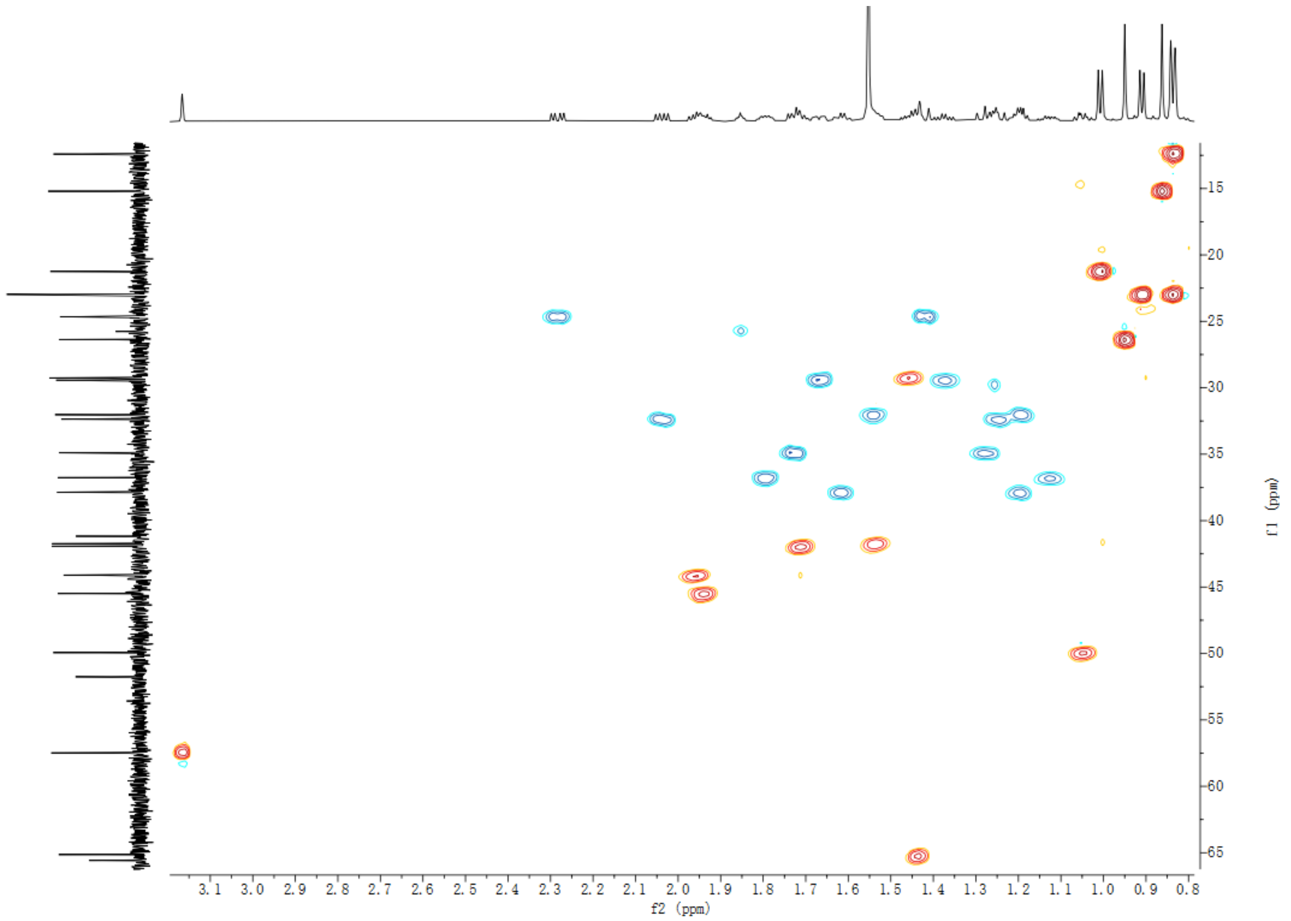

Figure S34. HSQC spectrum of compound **3a** in $CDCl_3$

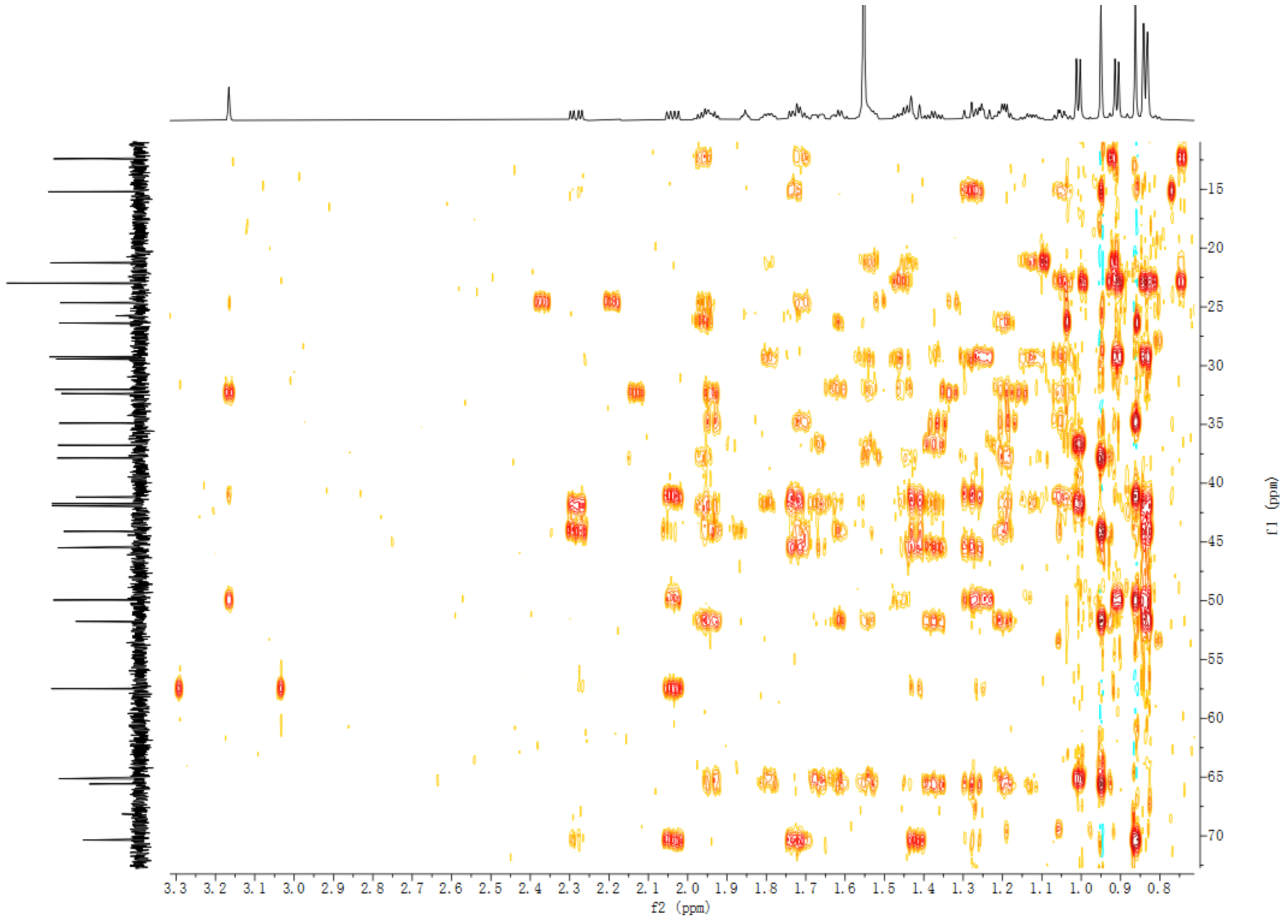

Figure S35. HMBC spectrum of compound **3a** in $CDCl_3$

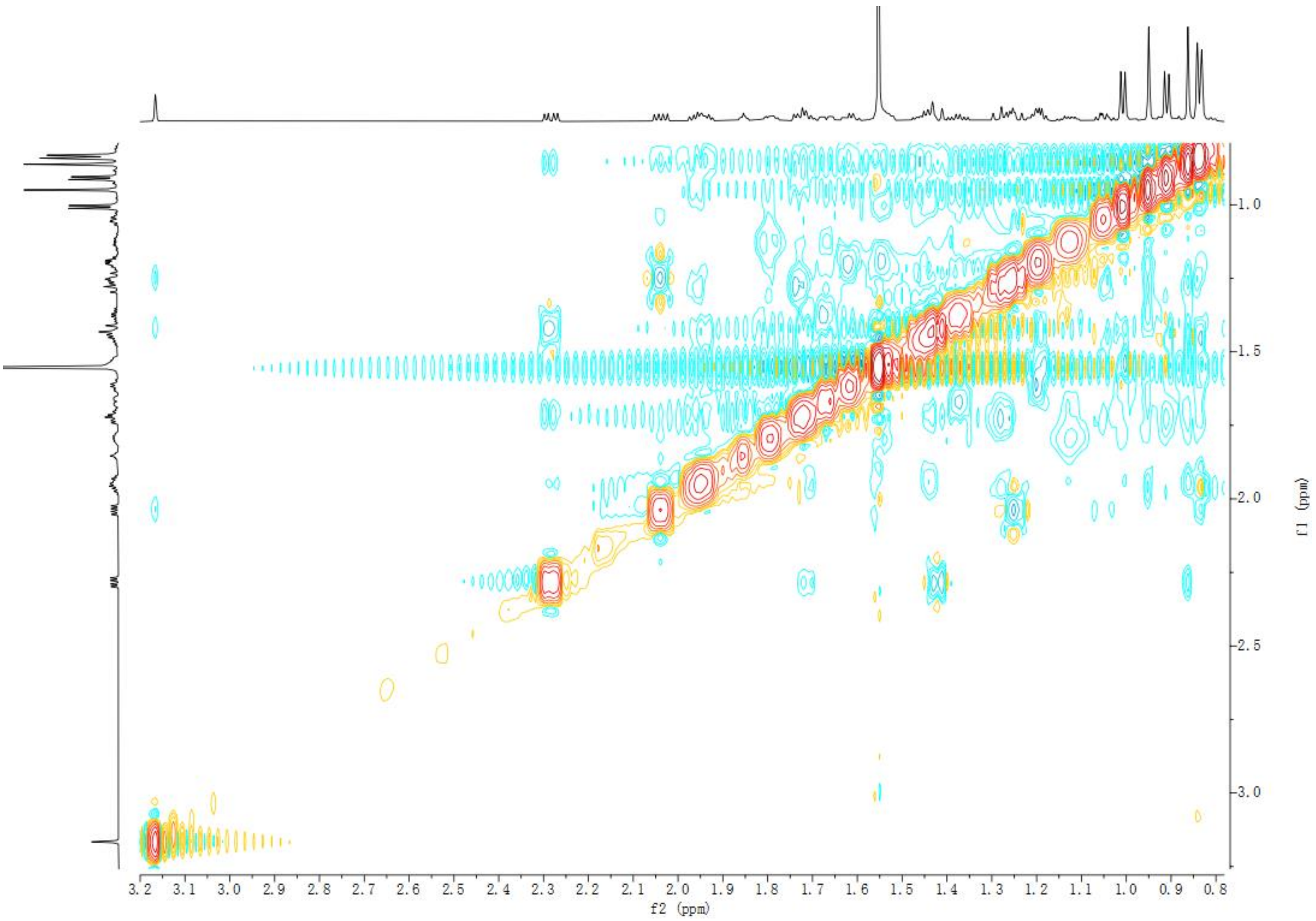

Figure S36. NOESY spectrum of compound **3a** in $CDCl_3$

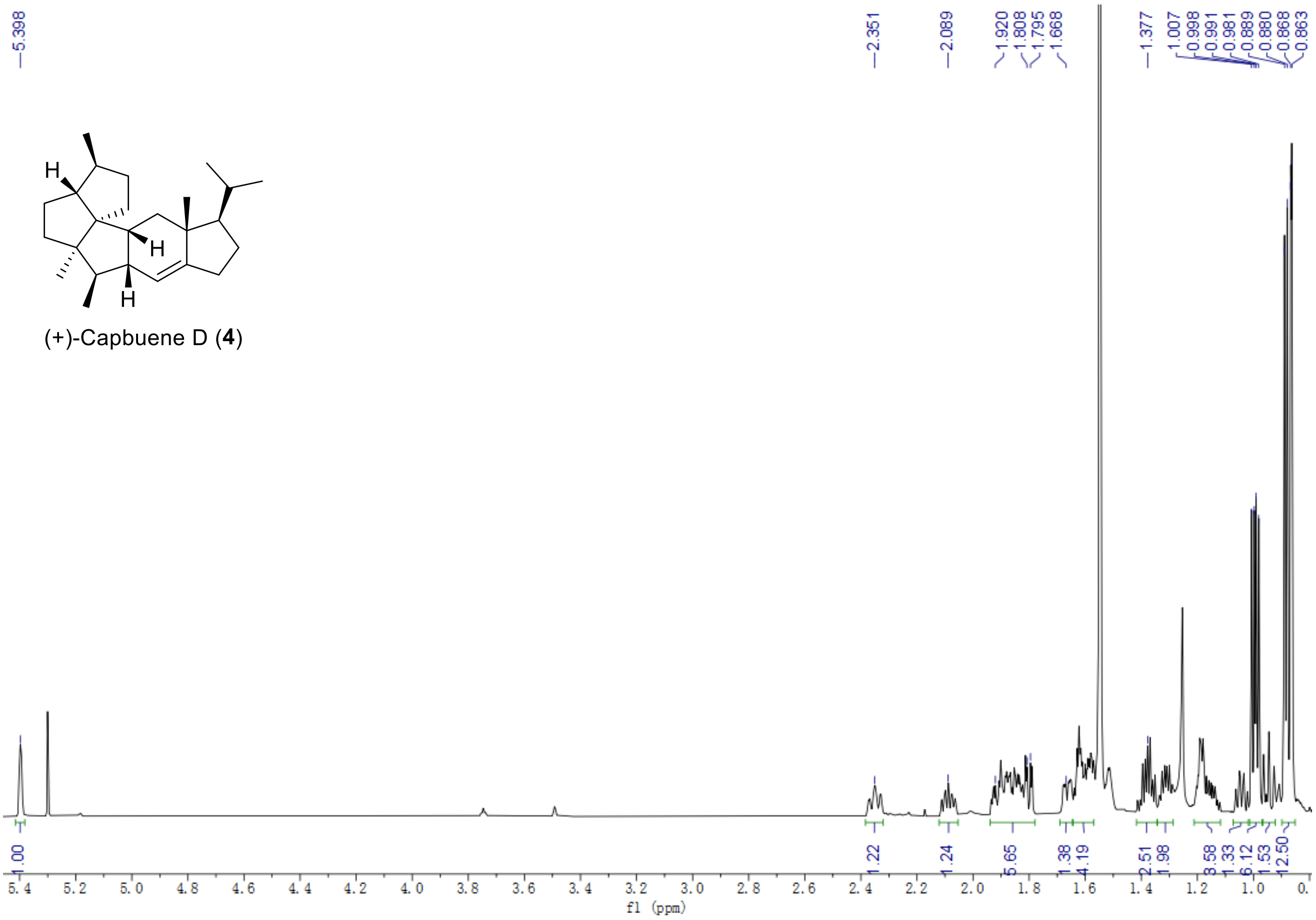

Figure S37. ^{1}H NMR spectrum of compound **4** in $CDCl_3$ (700 MHz)

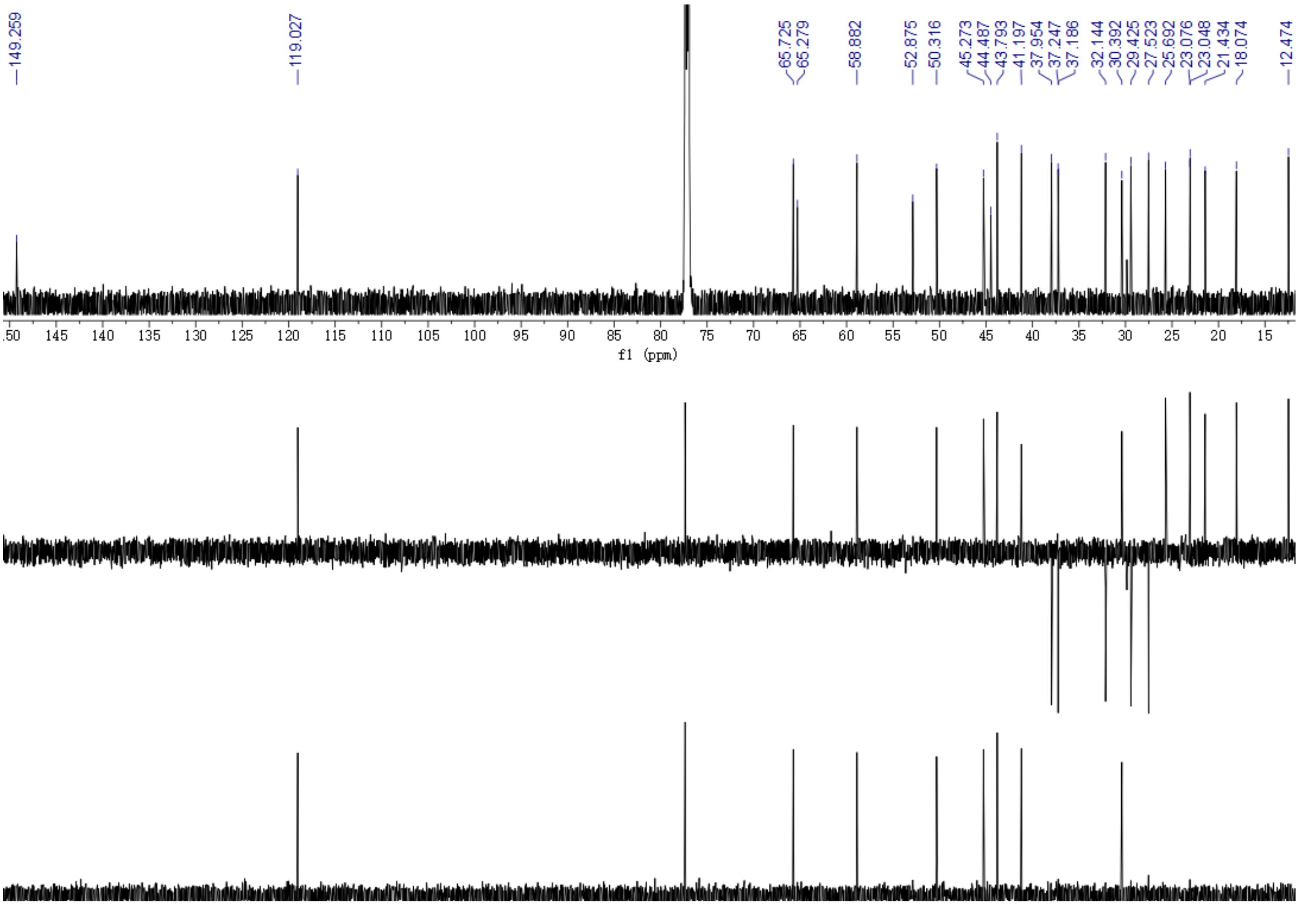

Figure S38. ^{13}C NMR and DEPT spectra of compound **4** in $CDCl_3$ (150 MHz)

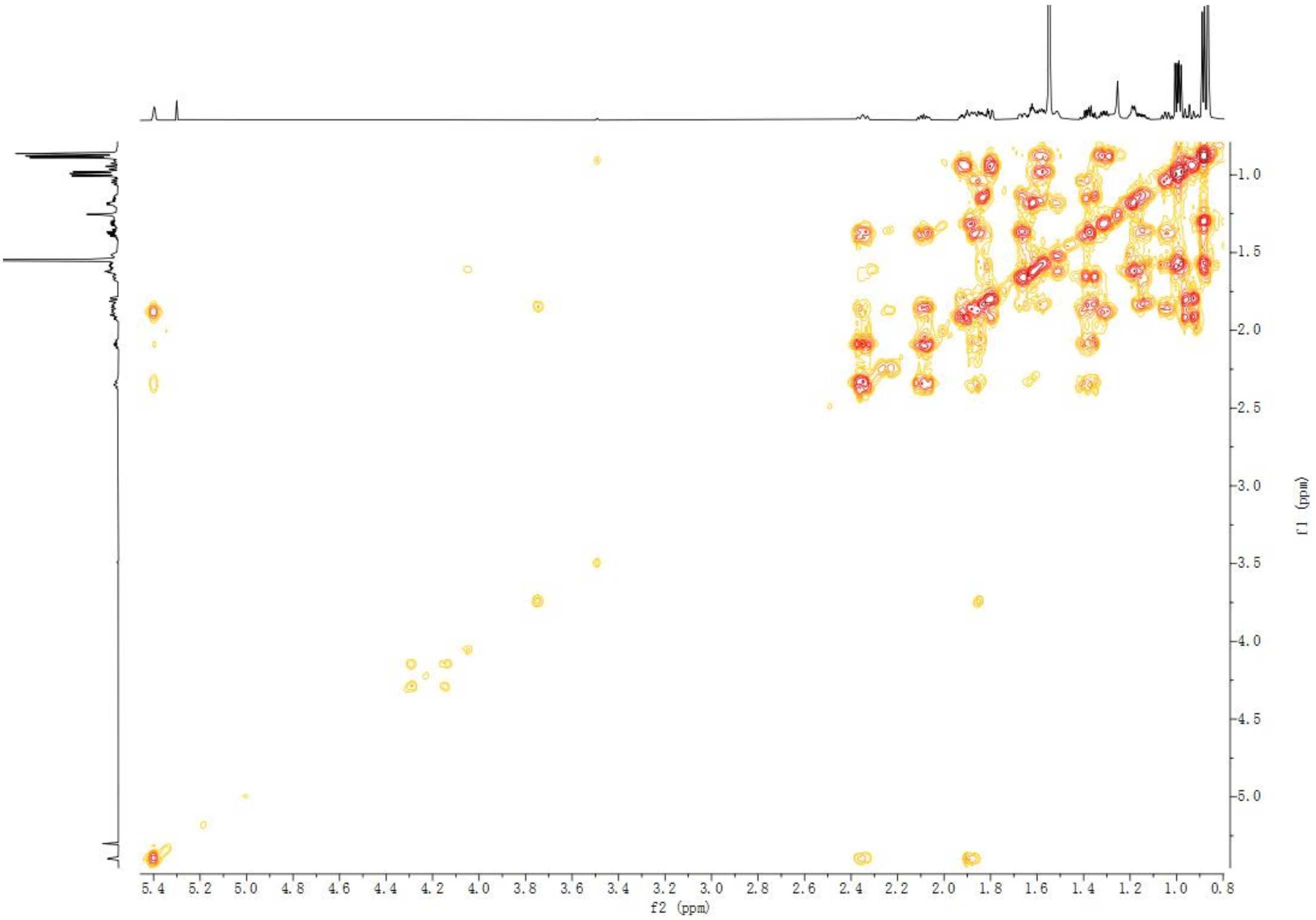

Figure S39. ^{1}H-^{1}H COSY spectrum of compound **4** in $CDCl_3$

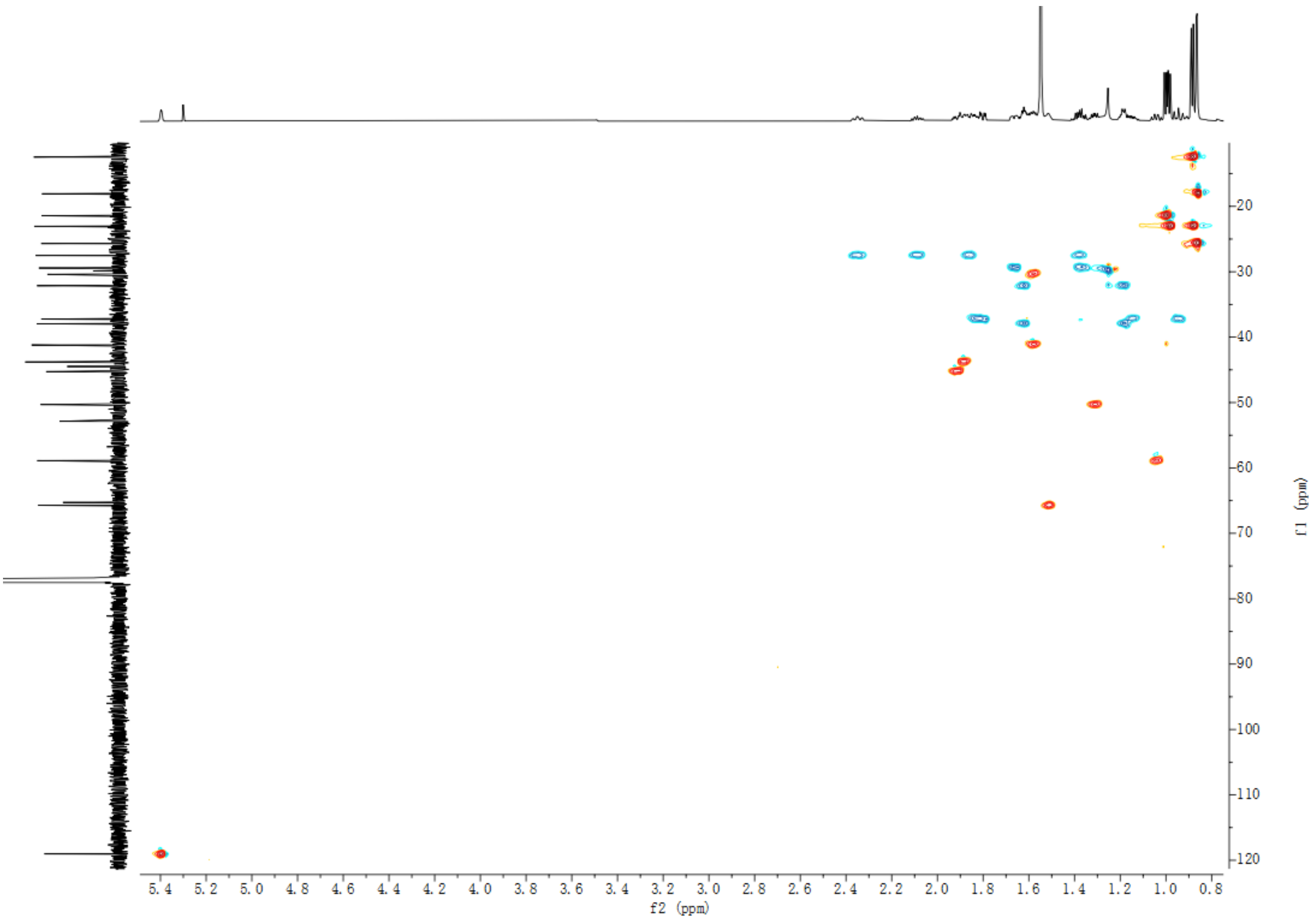

Figure S40. HSQC spectrum of compound **4** in $CDCl_3$

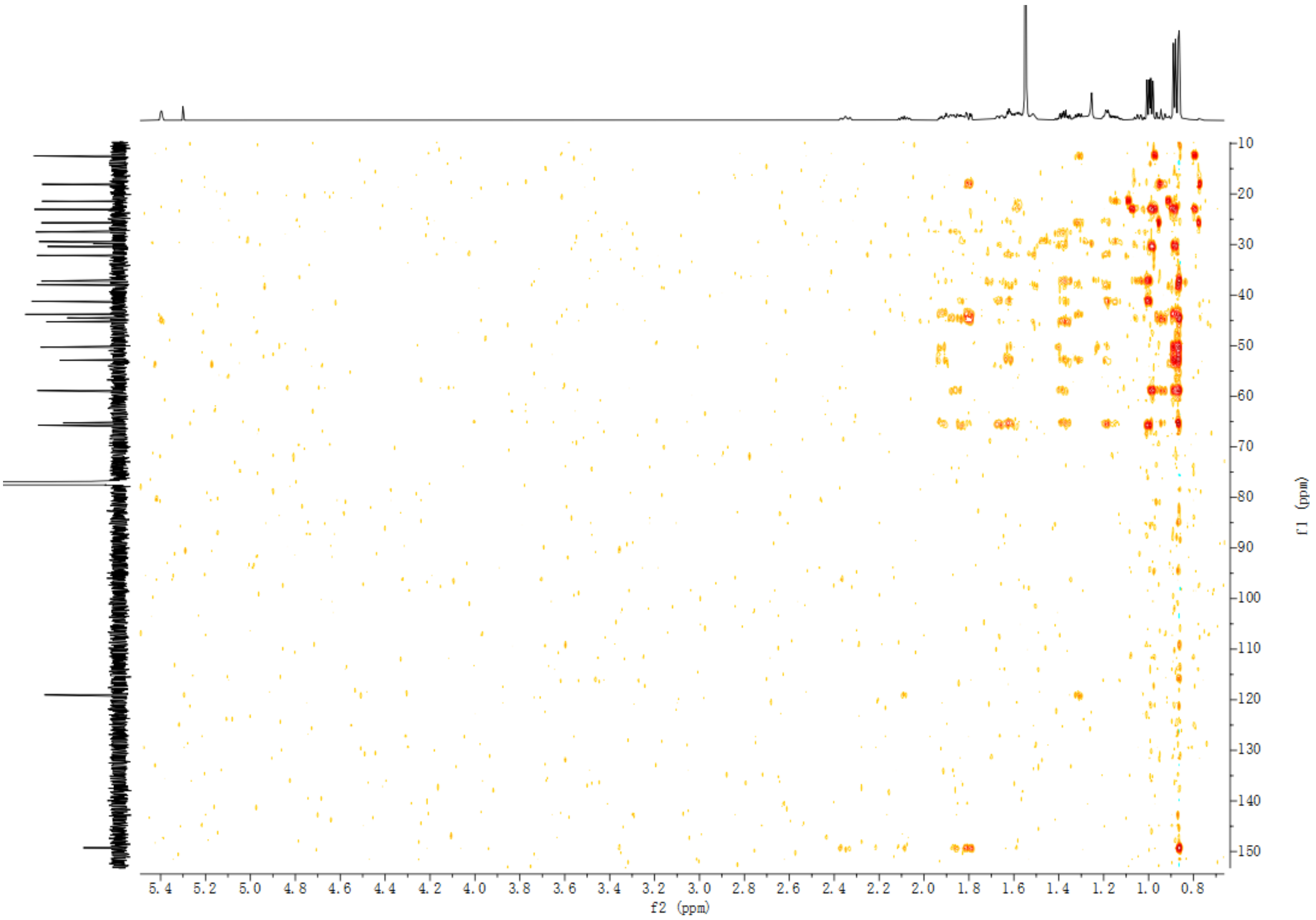

Figure S41. HMBC spectrum of compound **4** in $CDCl_3$

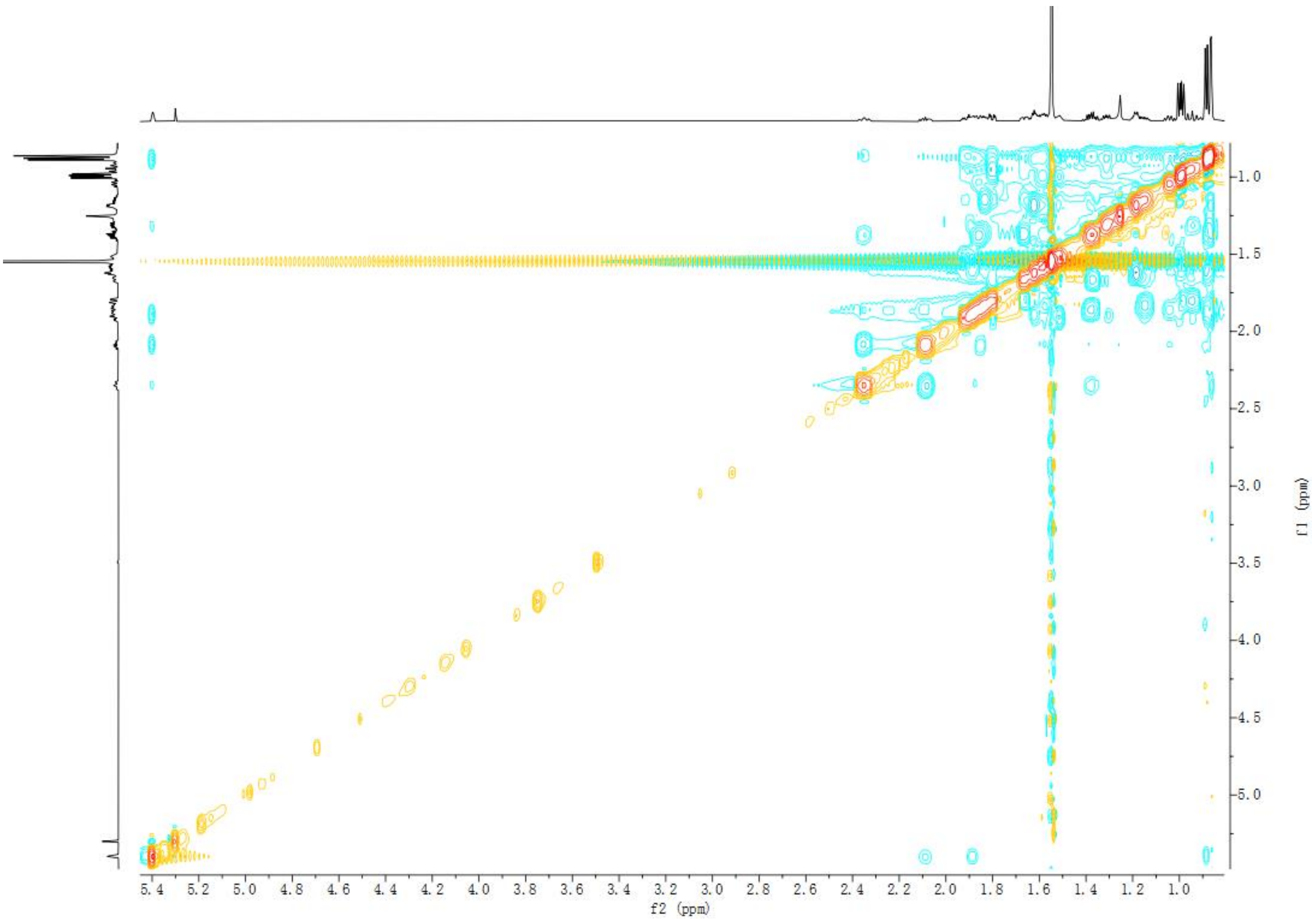

Figure S42. NOESY spectrum of compound **4** in $CDCl_3$

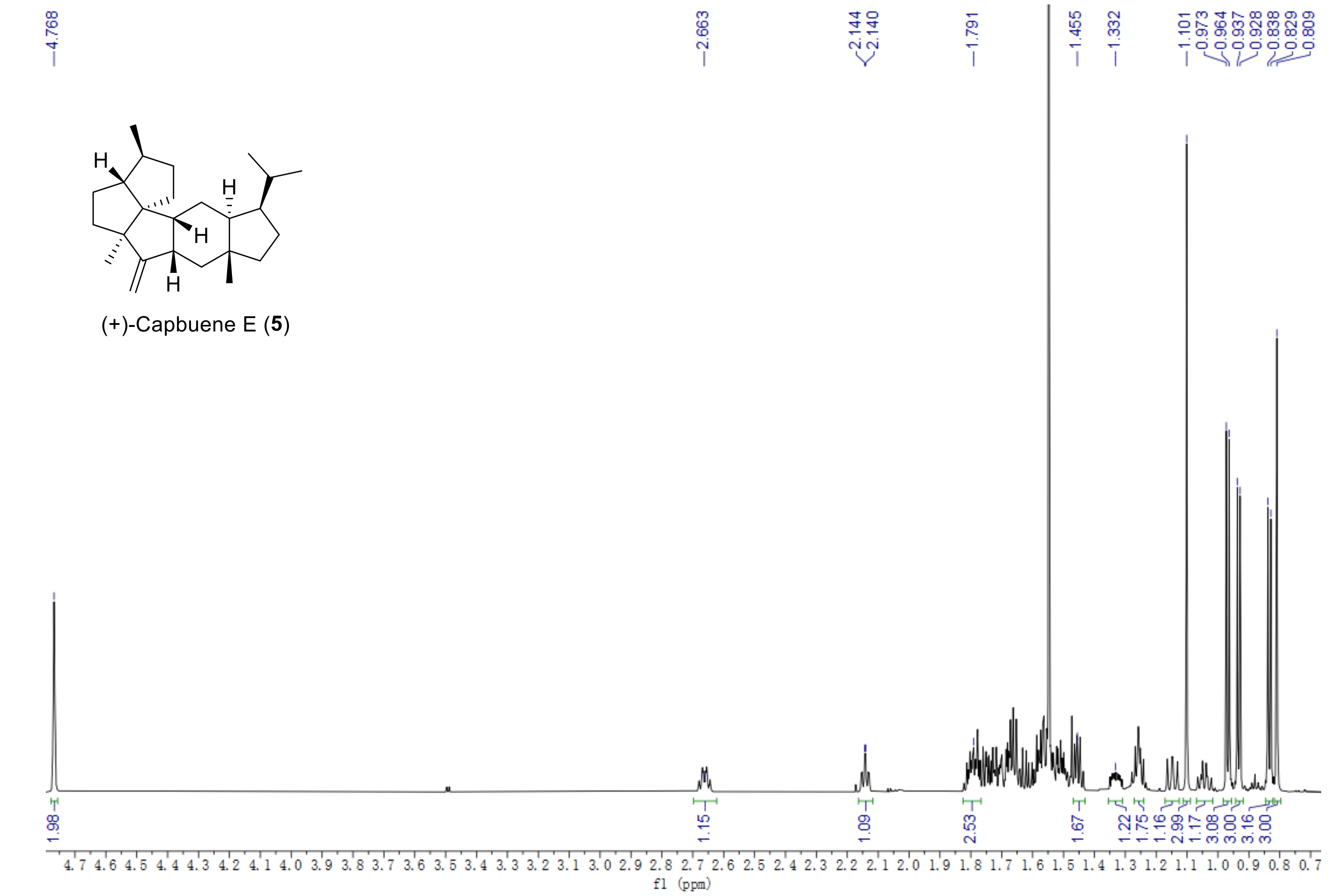

Figure S43. ^{1}H NMR spectrum of compound **5** in $CDCl_3$ (700 MHz)

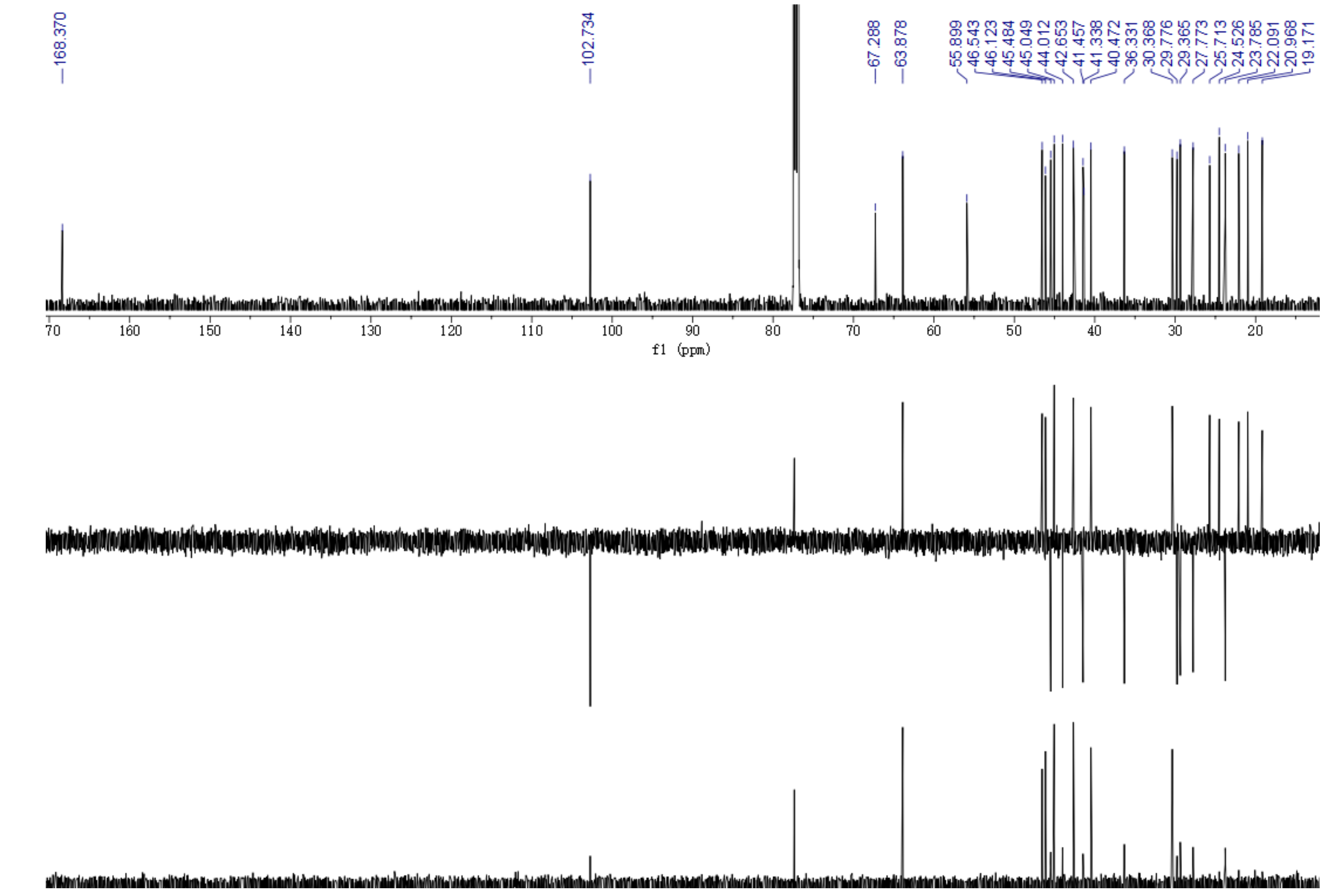

Figure S44. ^{13}C NMR and DEPT spectra of compound **5** in $CDCl_3$ (150 MHz)

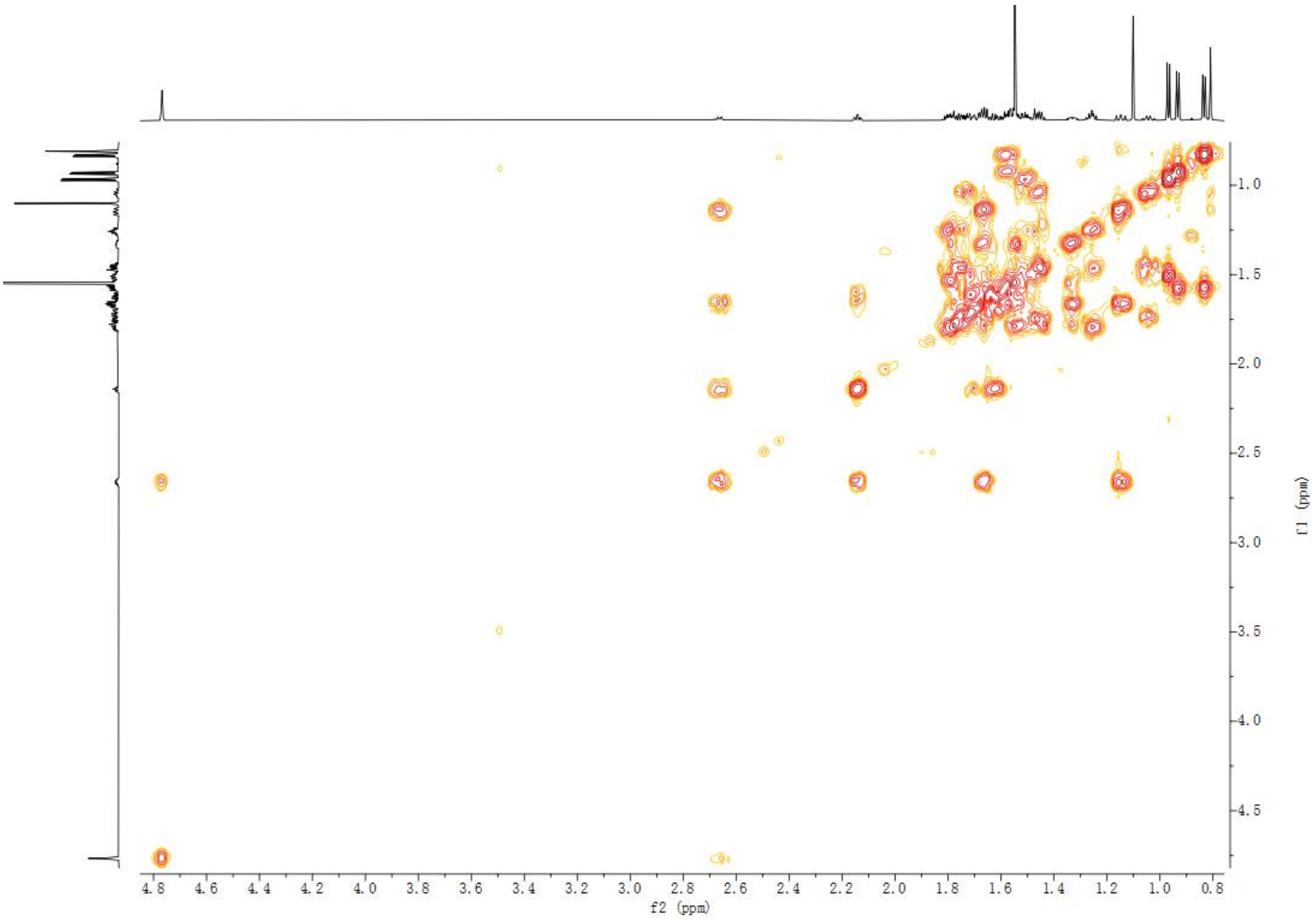

Figure S45. ^{1}H-^{1}H COSY spectrum of compound **5** in $CDCl_3$

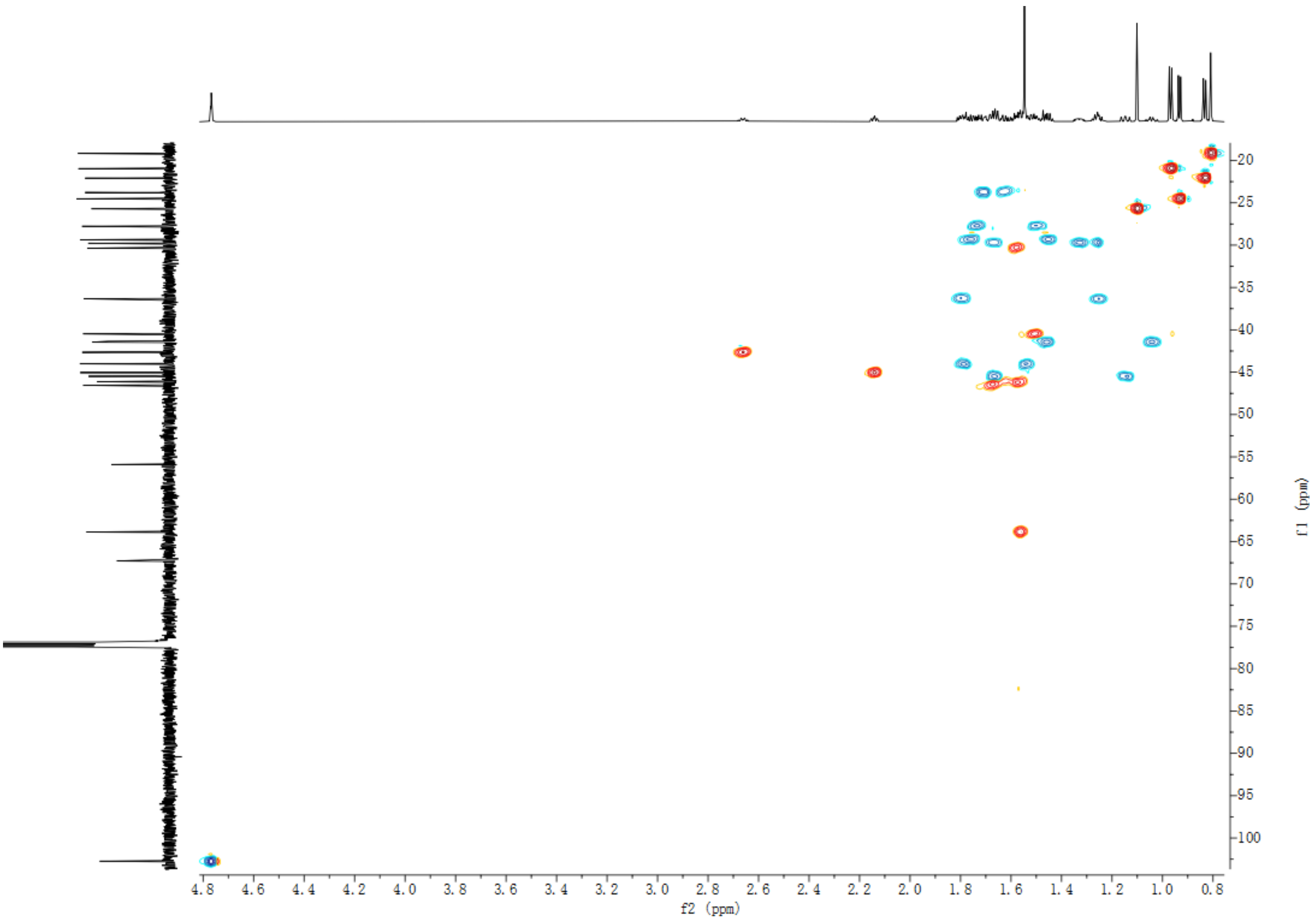

Figure S46. HSQC spectrum of compound **5** in $CDCl_3$

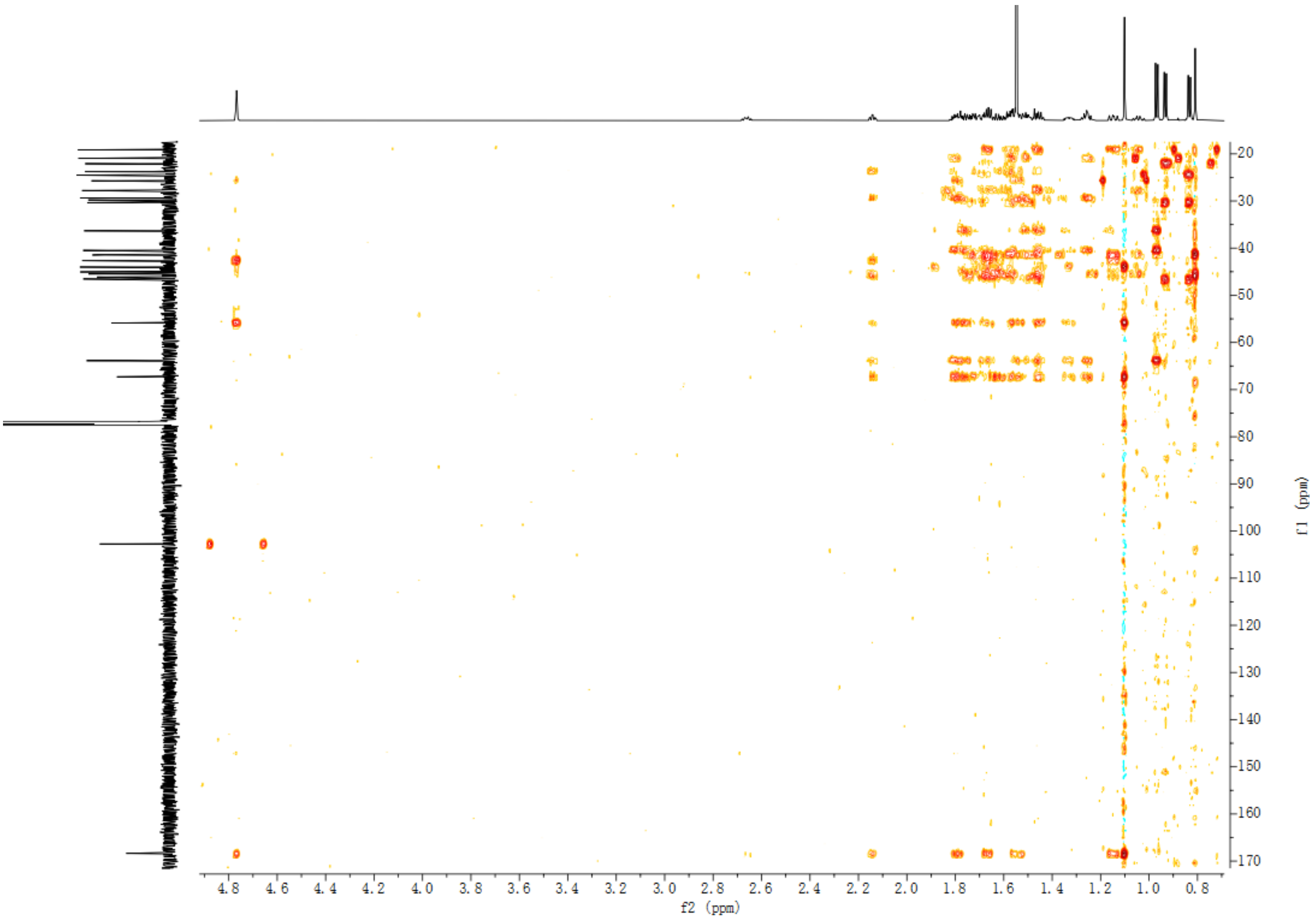

Figure S47. HMBC spectrum of compound **5** in $CDCl_3$

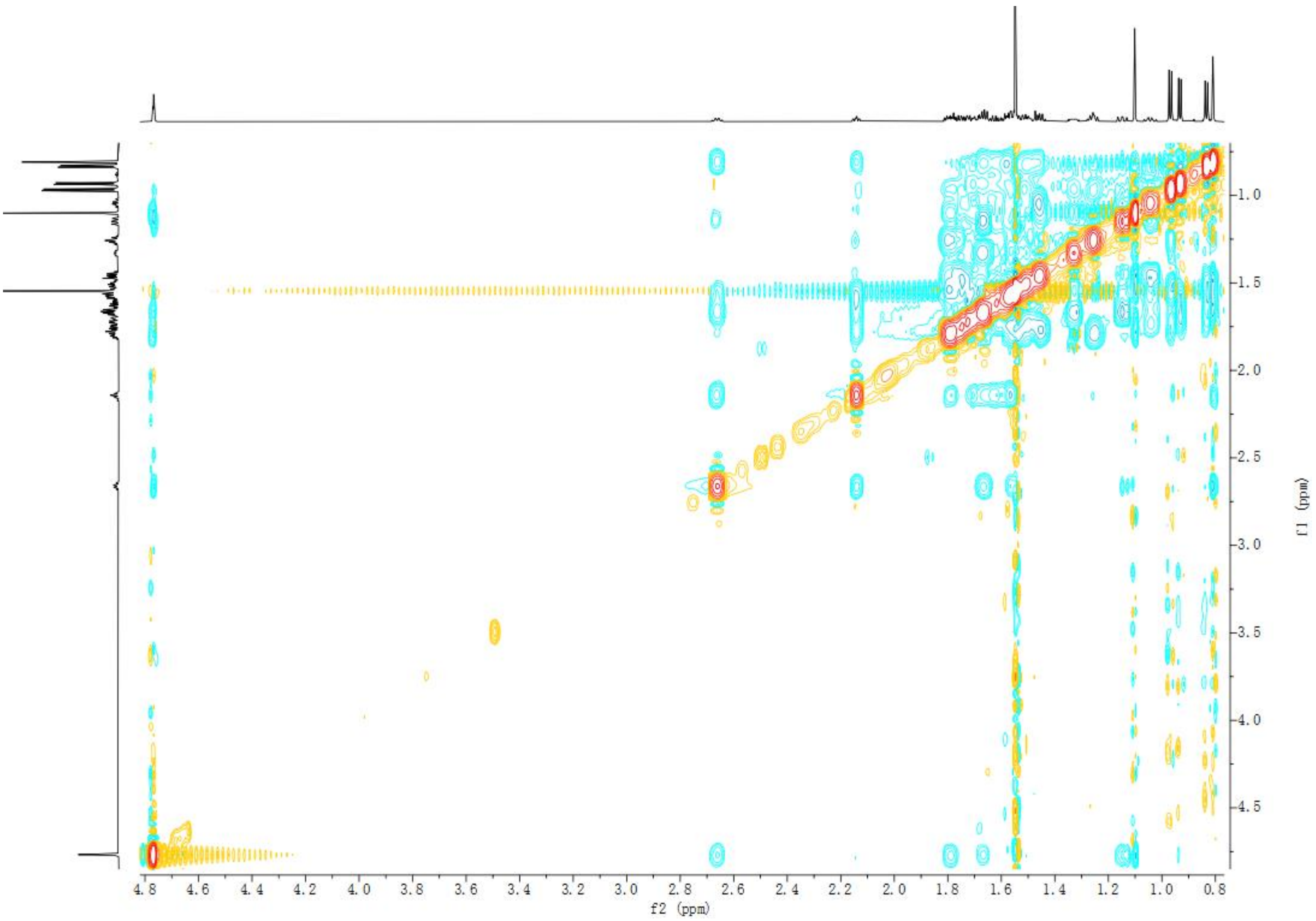

Figure S48. NOESY spectrum of compound **5** in $CDCl_3$

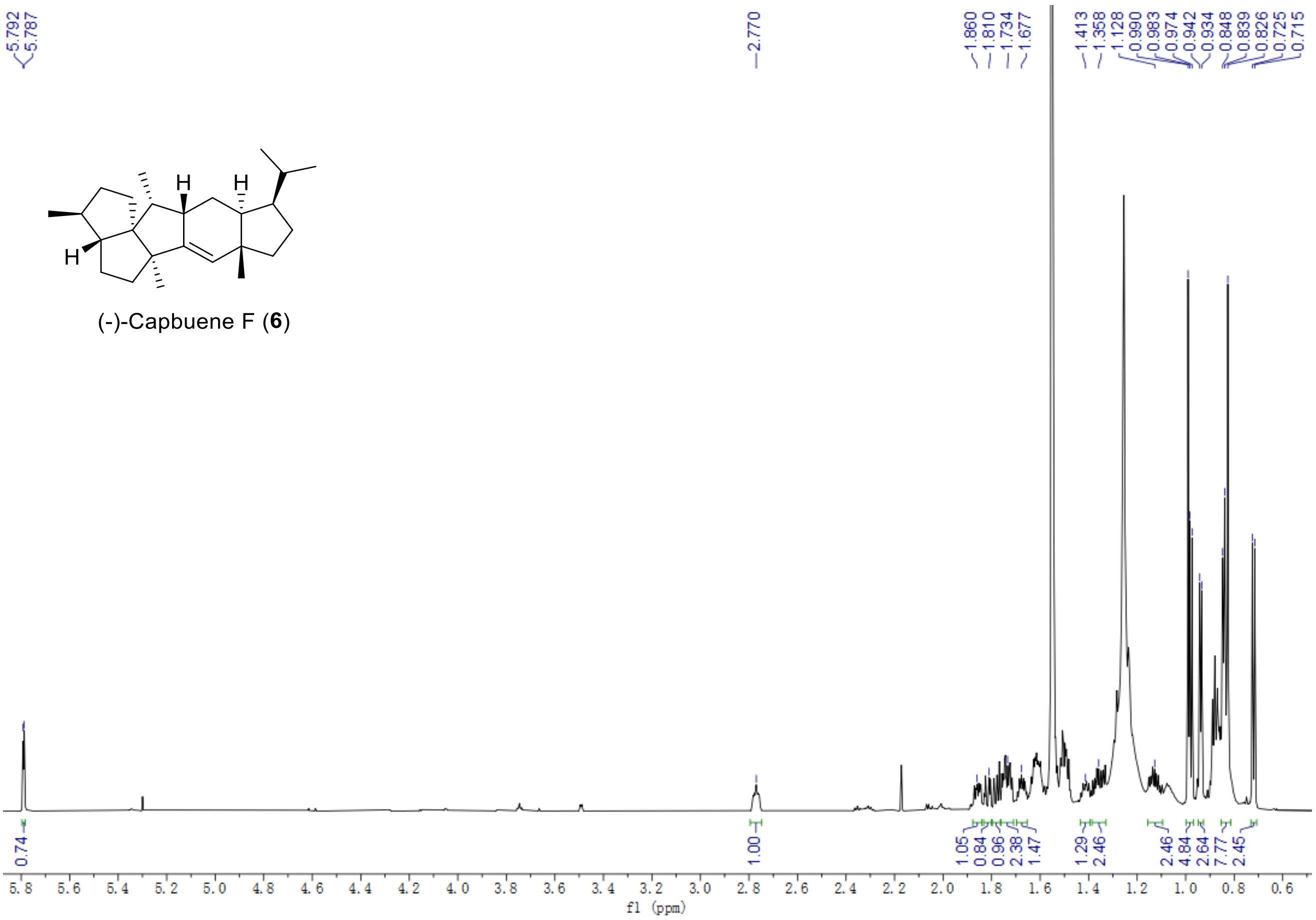

Figure S49. ^{1}H NMR spectrum of compound **6** in $CDCl_3$ (700 MHz)

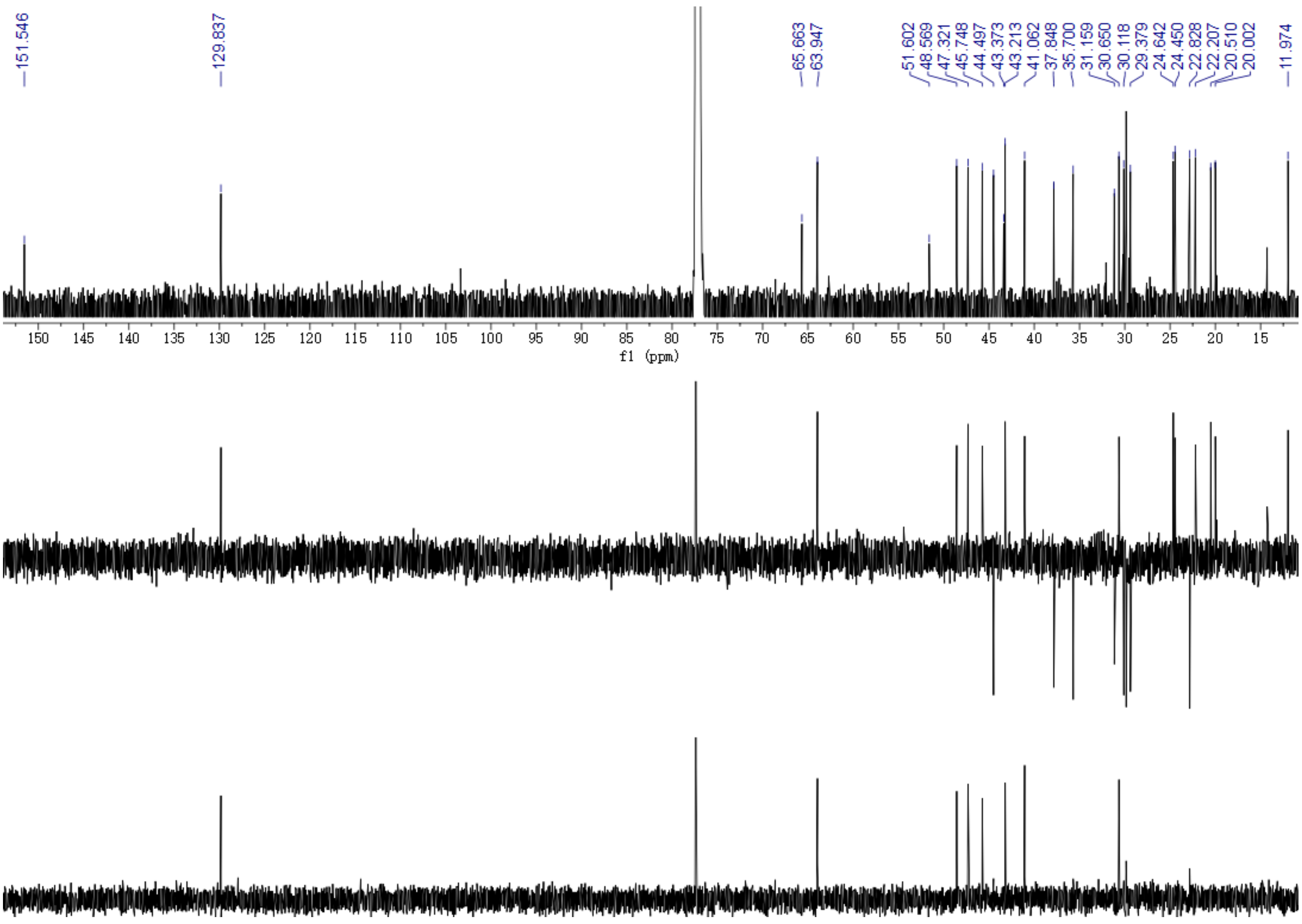

Figure S50. ^{13}C NMR and DEPT spectra of compound **6** in $CDCl_3$ (150 MHz)

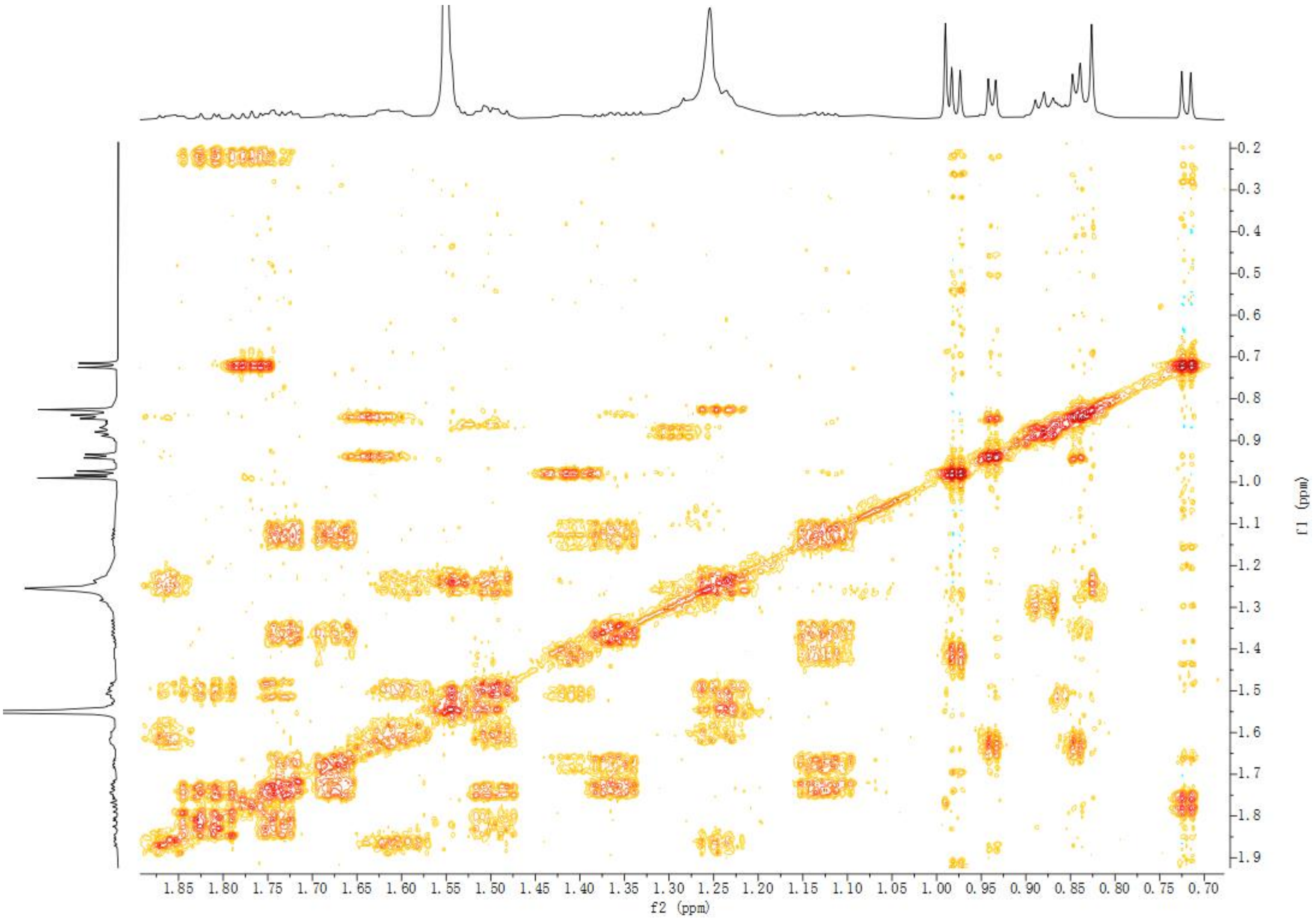

Figure S51. ^{1}H-^{1}H COSY spectrum of compound **6** in $CDCl_3$

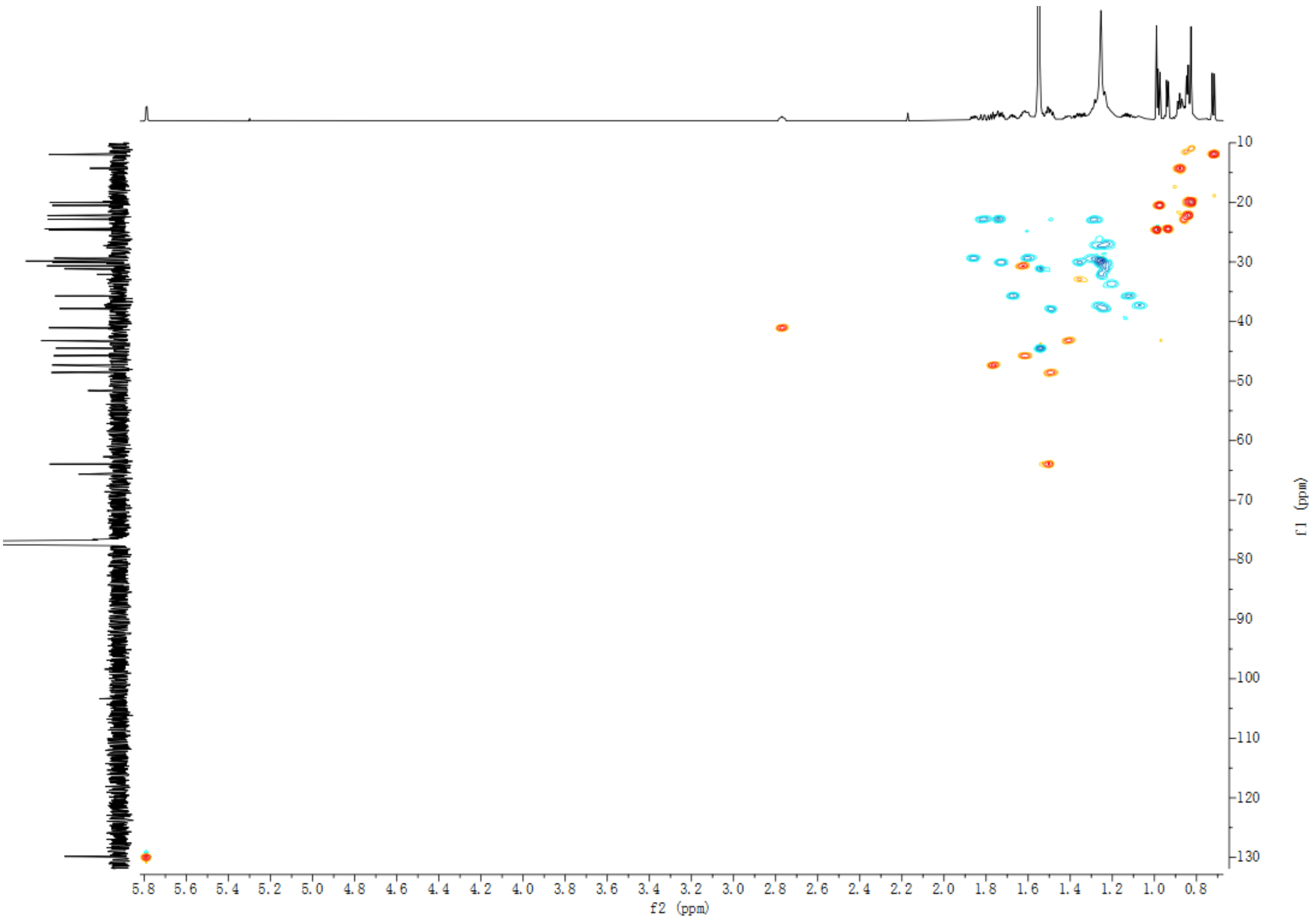

Figure S52. HSQC spectrum of compound **6** in $CDCl_3$

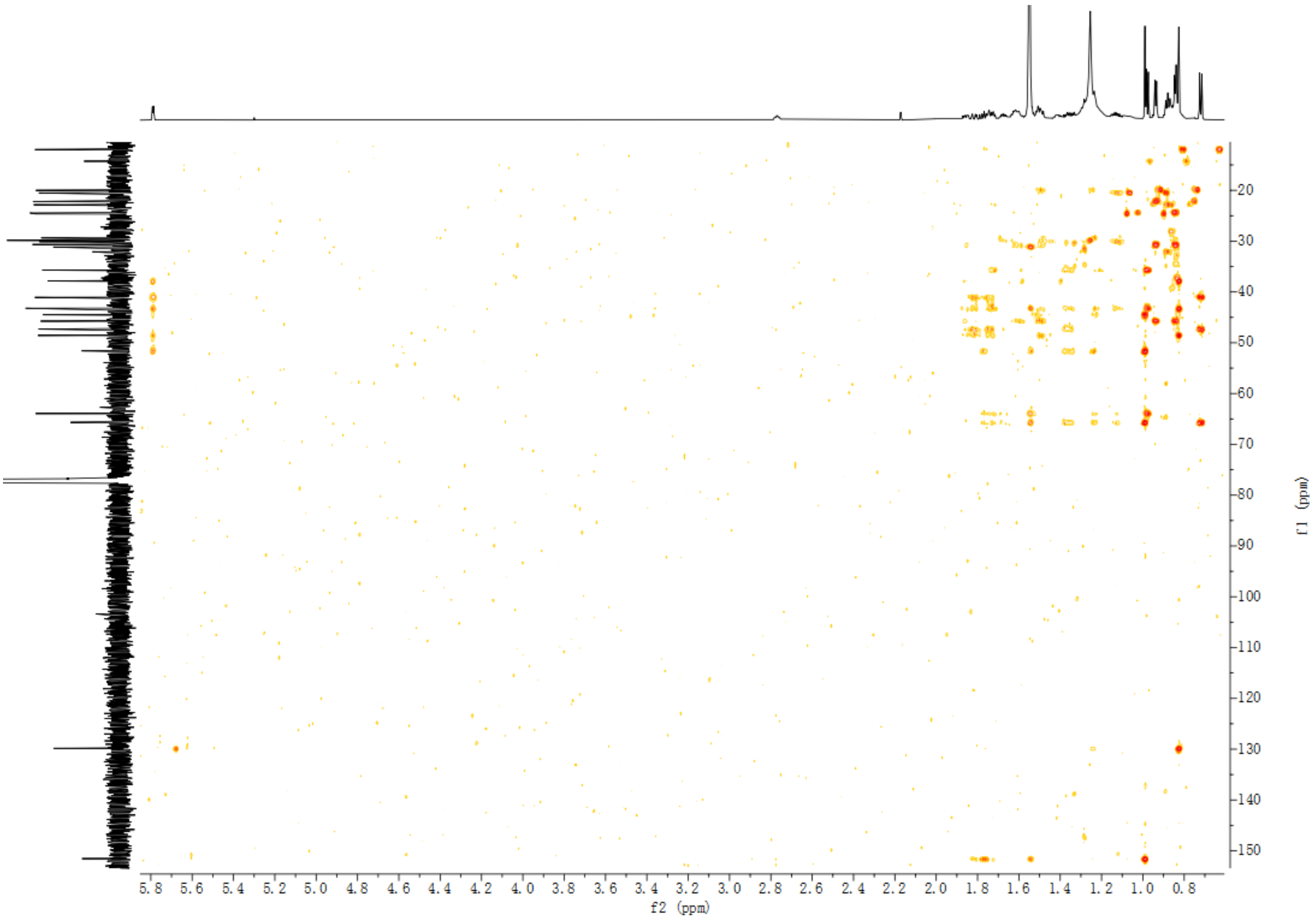

Figure S53. HMBC spectrum of compound **6** in $CDCl_3$

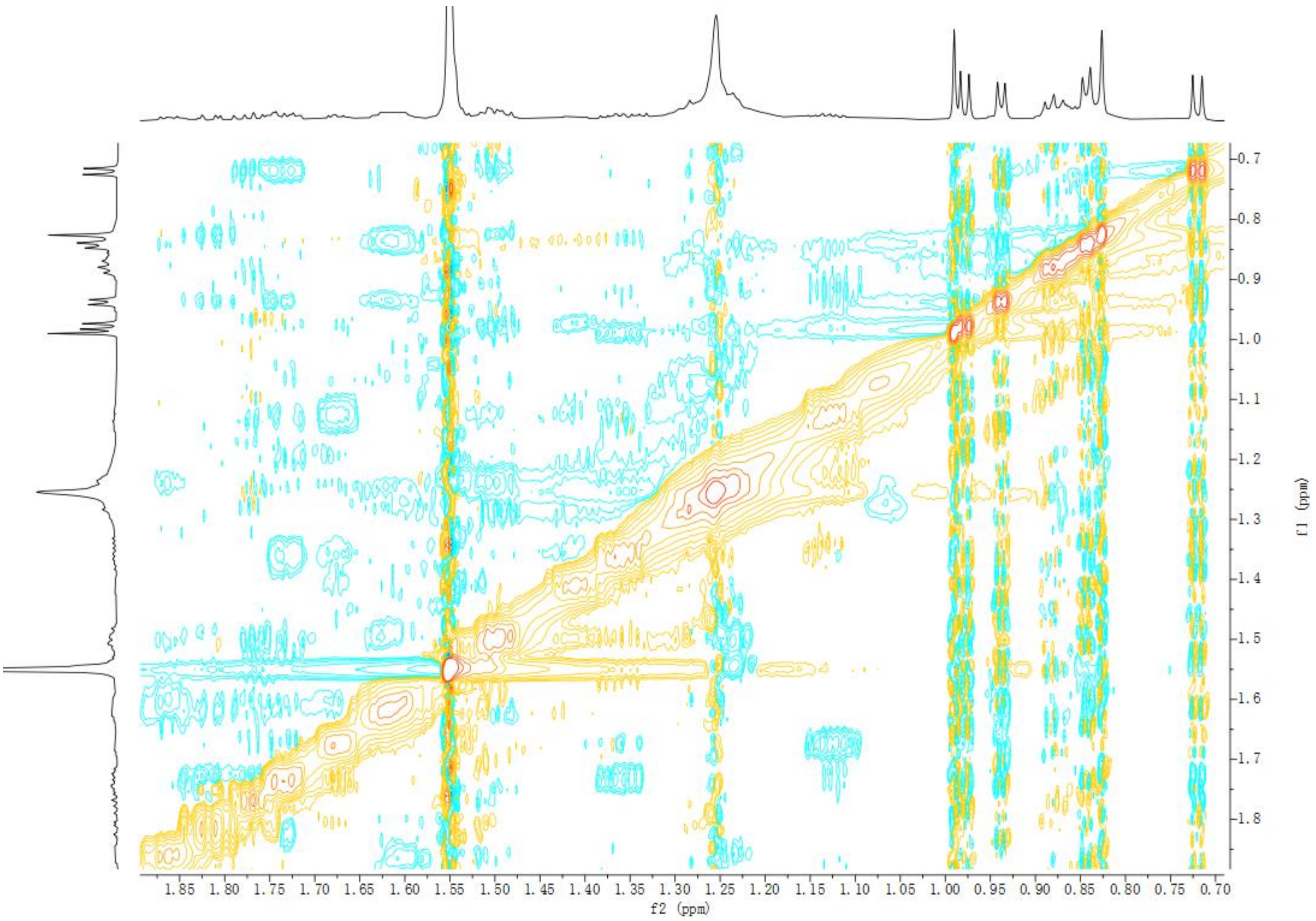

Figure S54. NOESY spectrum of compound **6** in $CDCl_3$

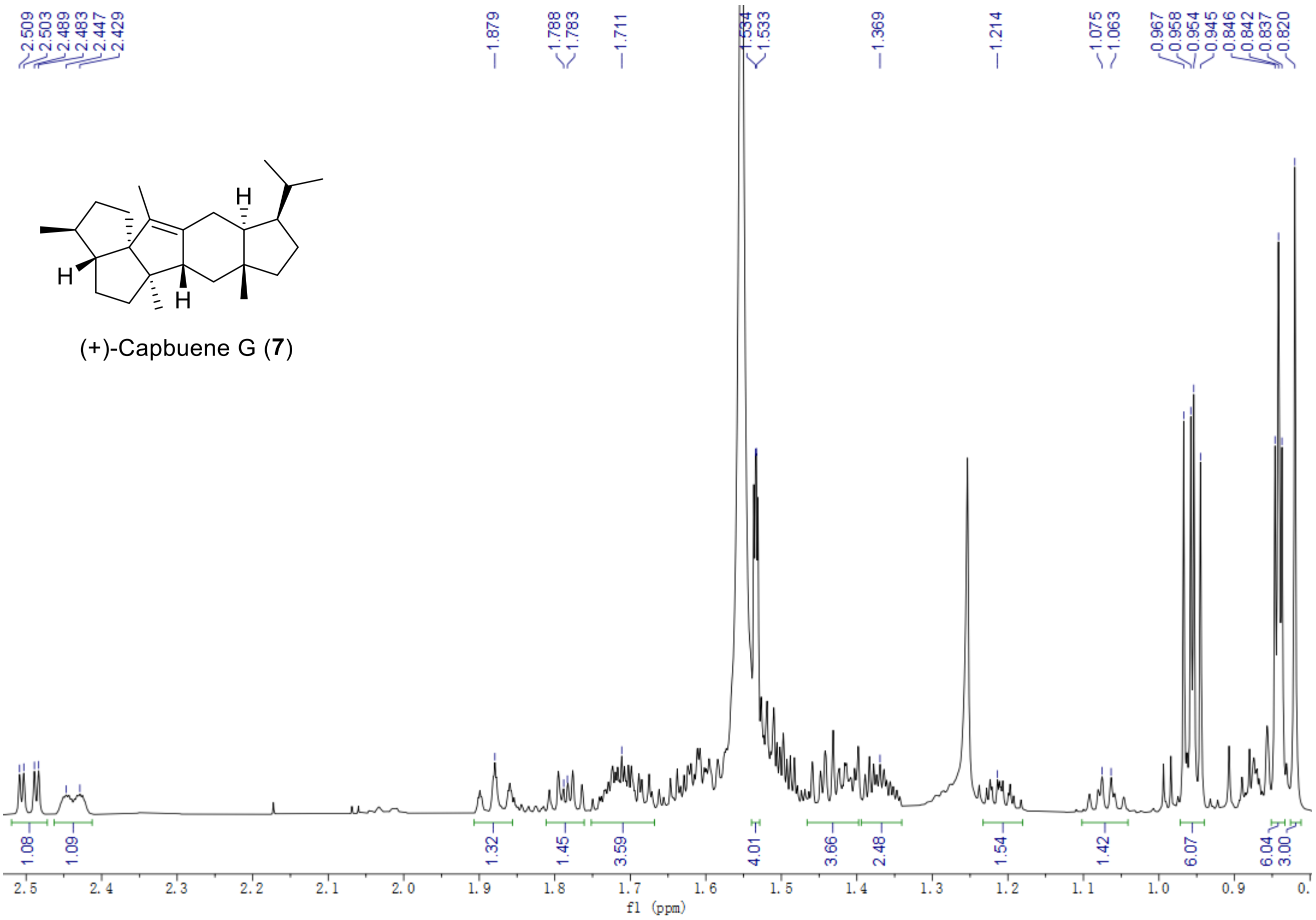

Figure S55. ¹H NMR spectrum of compound **7** in $CDCl_3$ (700 MHz)

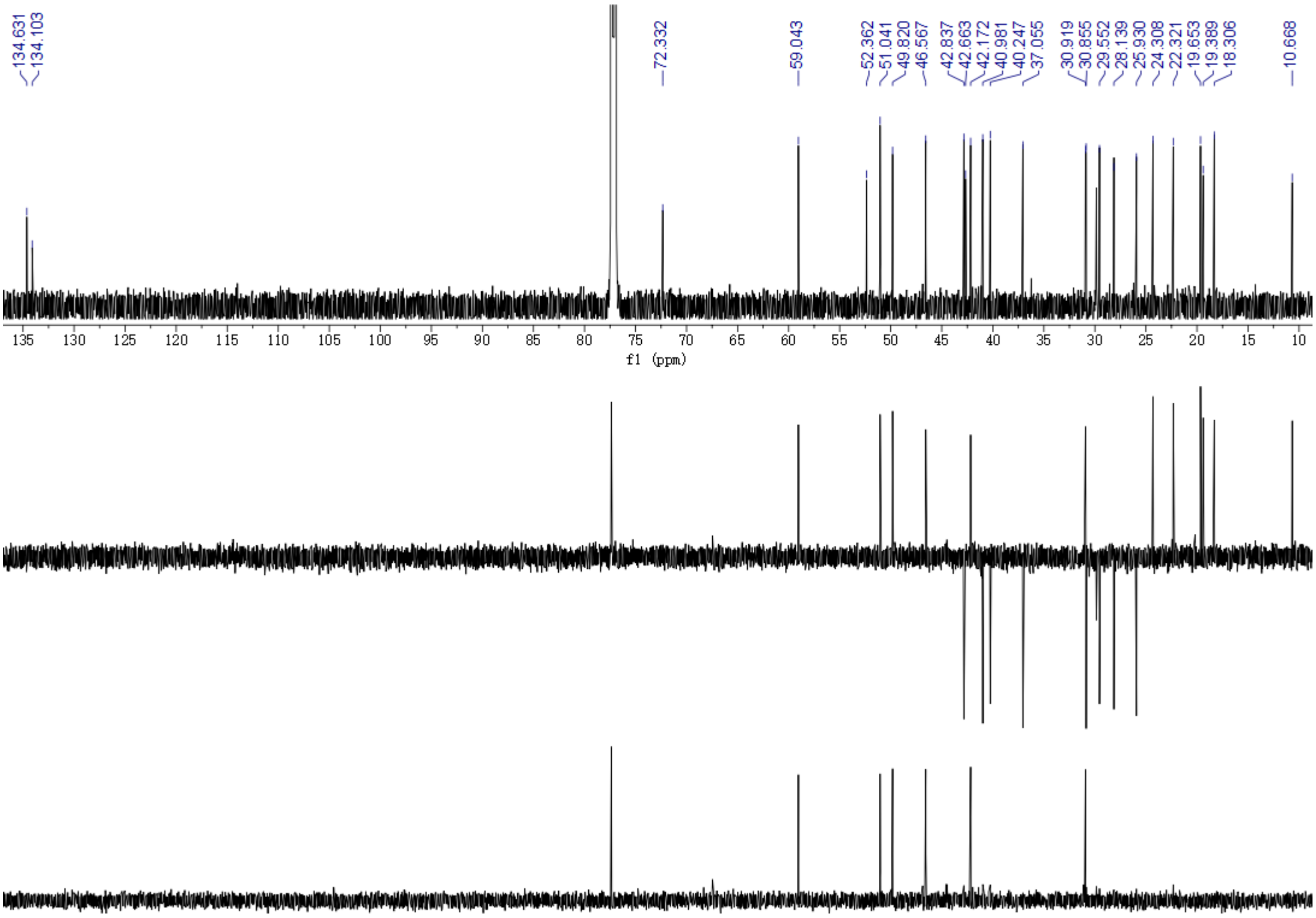

Figure S56. ¹³C NMR and DEPT spectra of compound **7** in $CDCl_3$ (150 MHz)

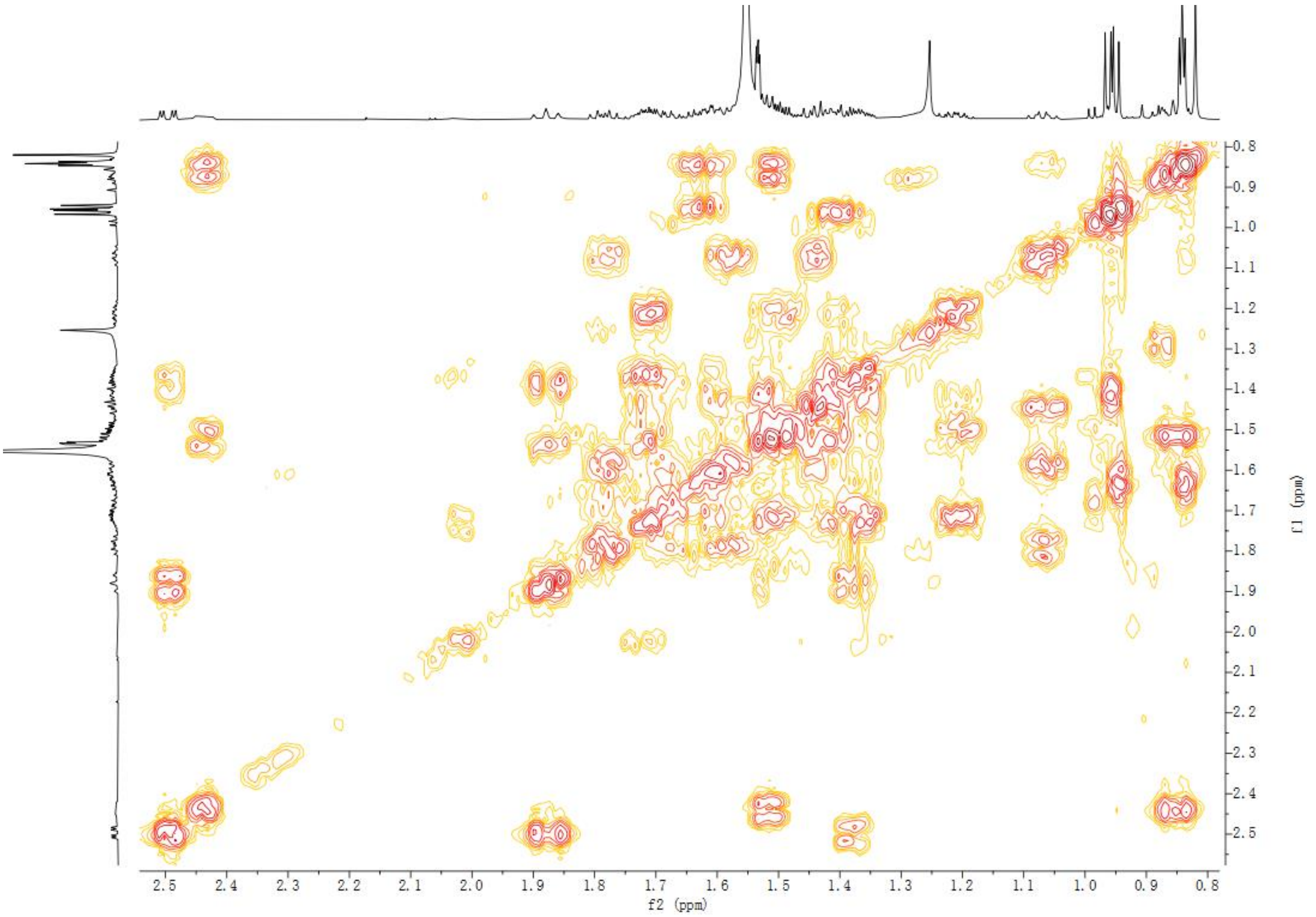

Figure S57. ^{1}H-^{1}H COSY spectrum of compound **7** in $CDCl_3$

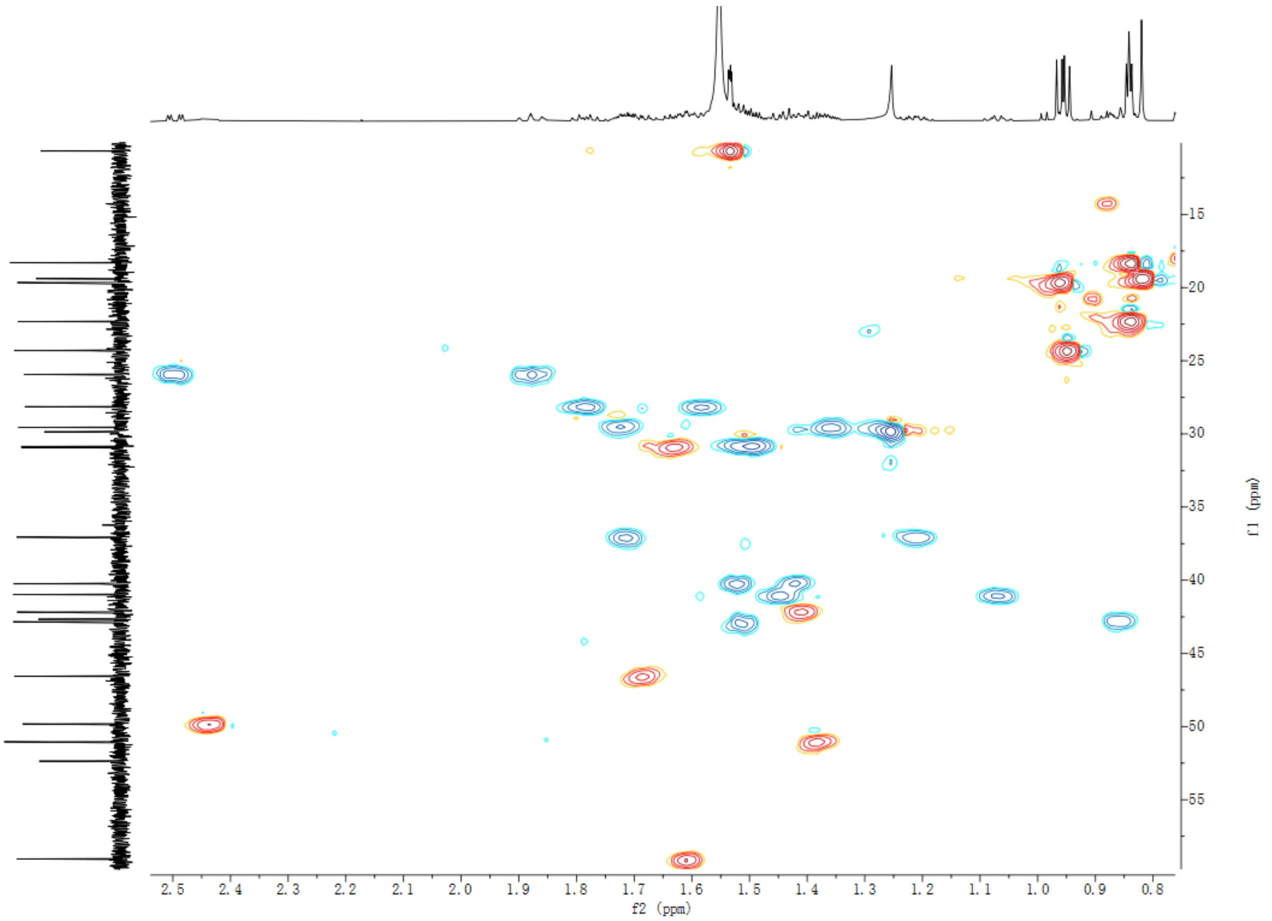

Figure S58. HSQC spectrum of compound **7** in $CDCl_3$

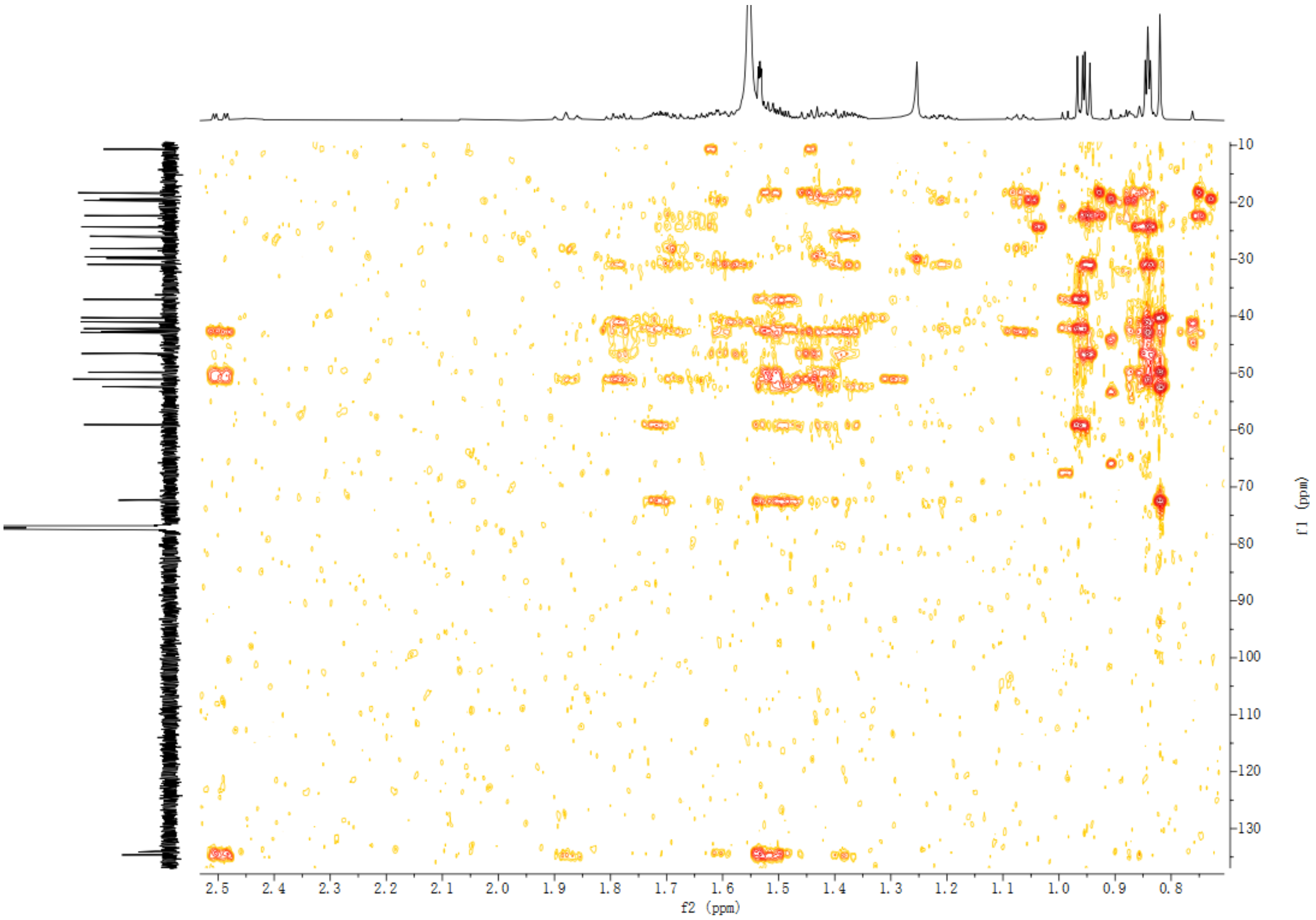

Figure S59. HMBC spectrum of compound **7** in $CDCl_3$

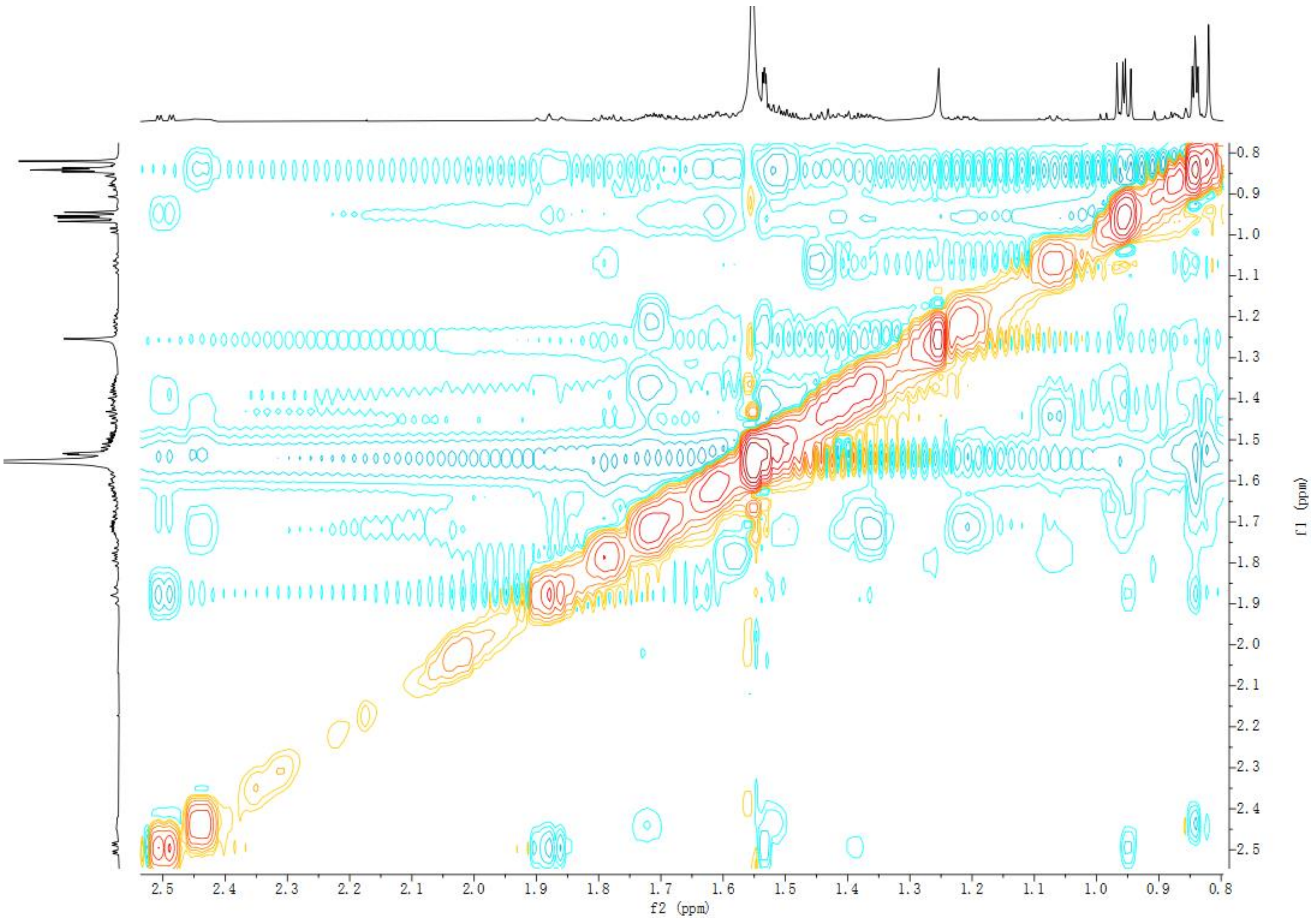

Figure S60. NOESY spectrum of compound **7** in $CDCl_3$

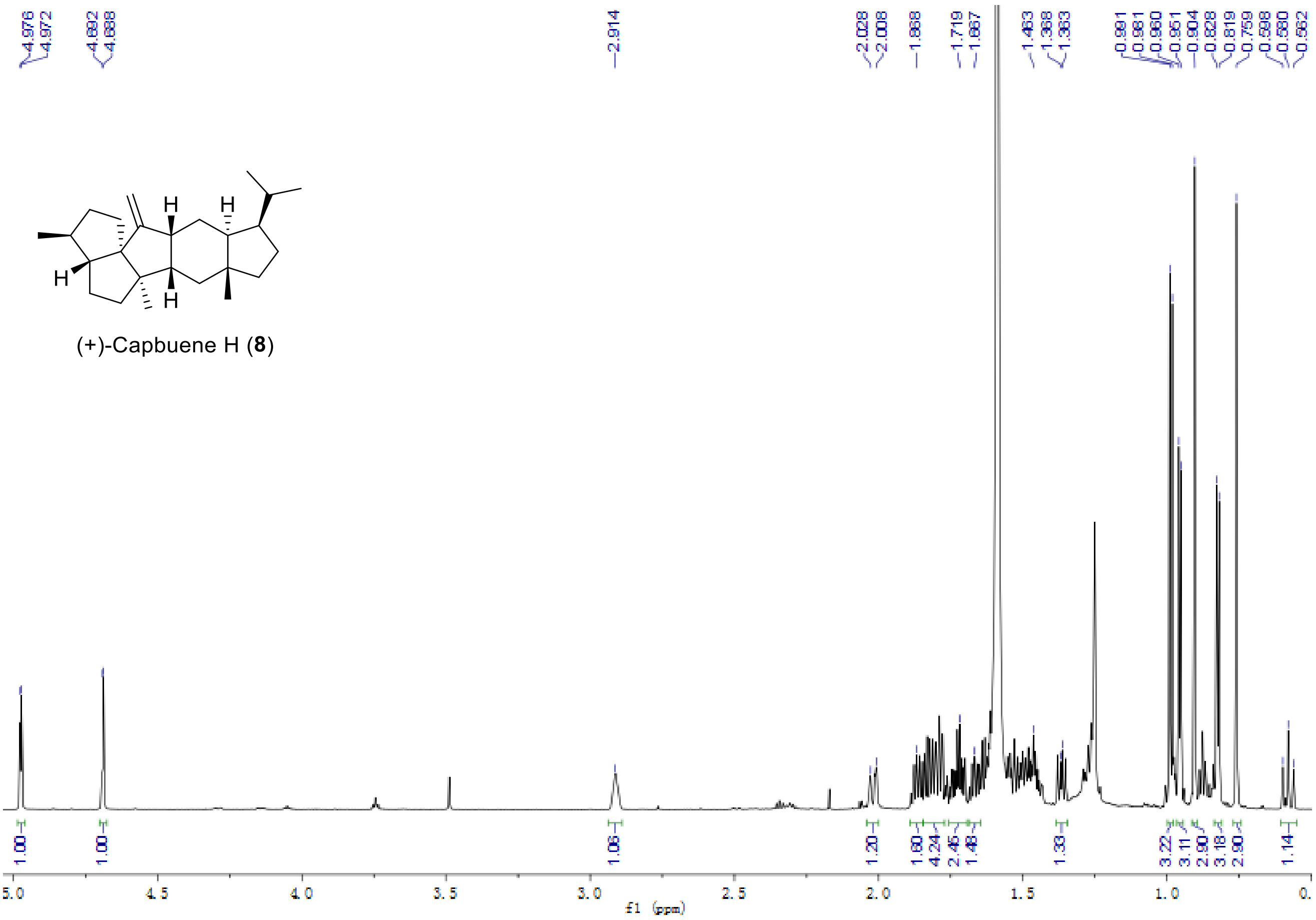

Figure S61. ^{1}H NMR spectrum of compound **8** in $CDCl_3$ (700 MHz)

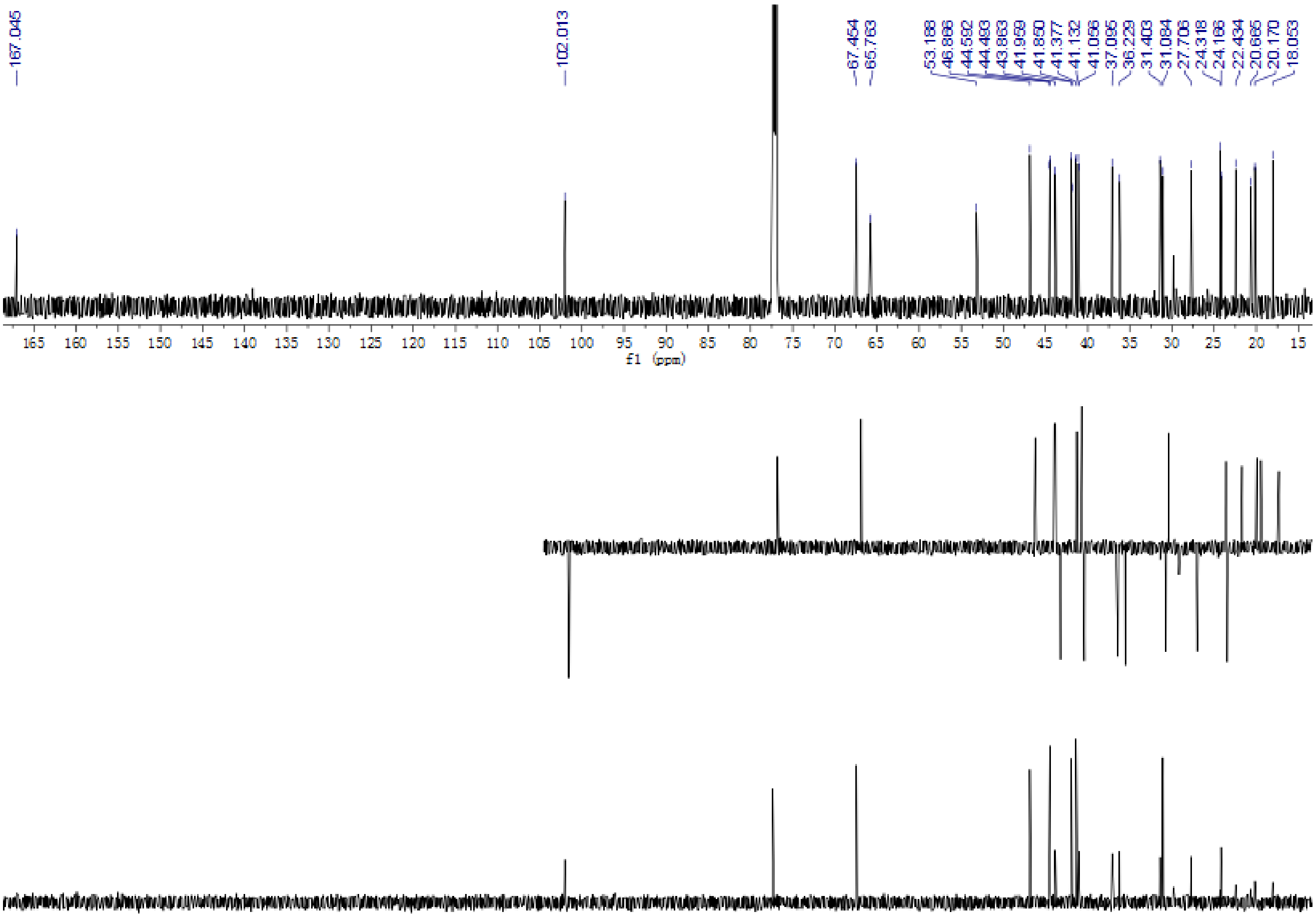

Figure S62. ^{13}C NMR and DEPT spectra of compound **8** in $CDCl_3$ (150 MHz)

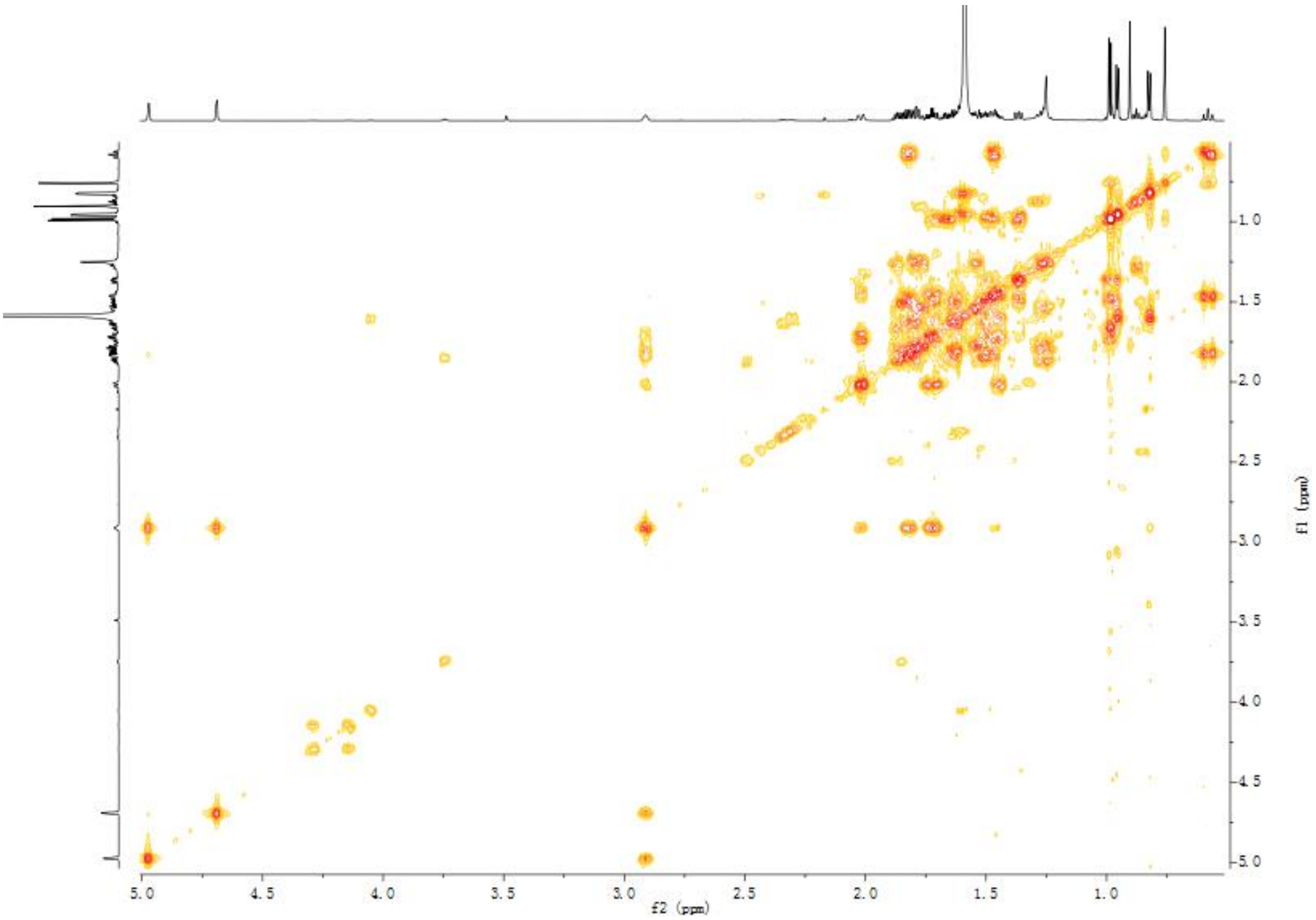

Figure S63. ^{1}H-^{1}H COSY spectrum of compound **8** in $CDCl_3$

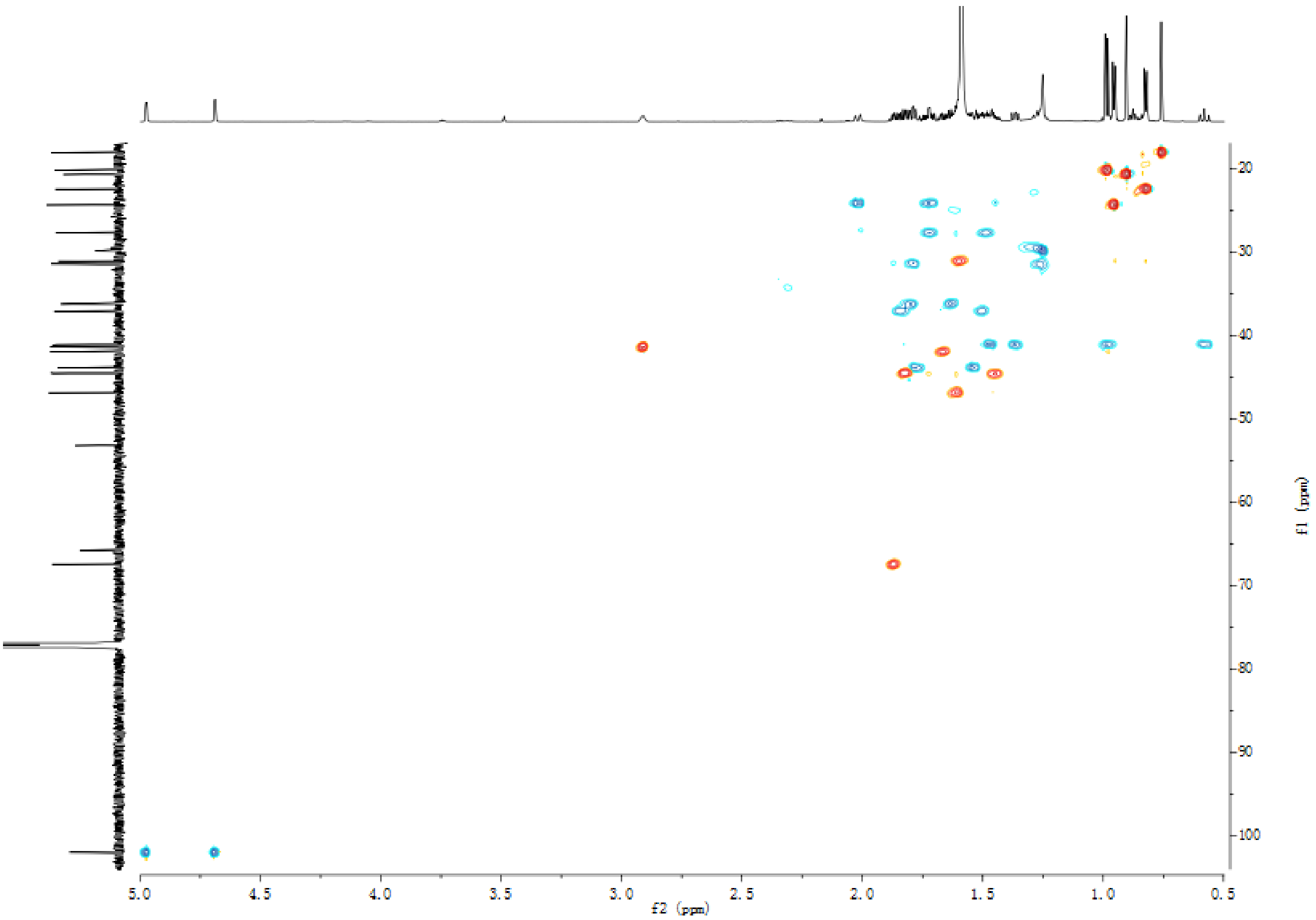

Figure S64. HSQC spectrum of compound **8** in $CDCl_3$

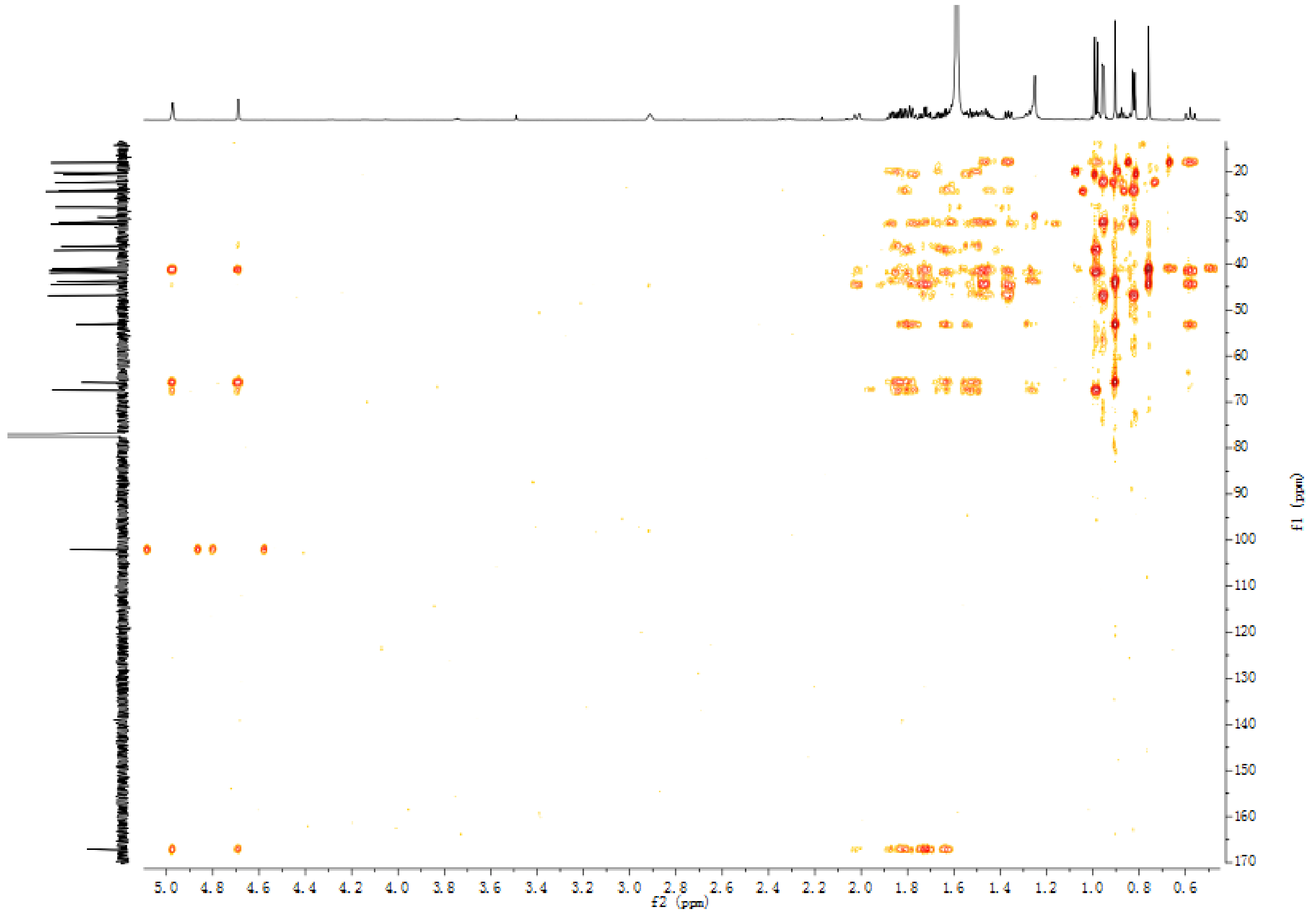

Figure S65. HMBC spectrum of compound **8** in $CDCl_3$

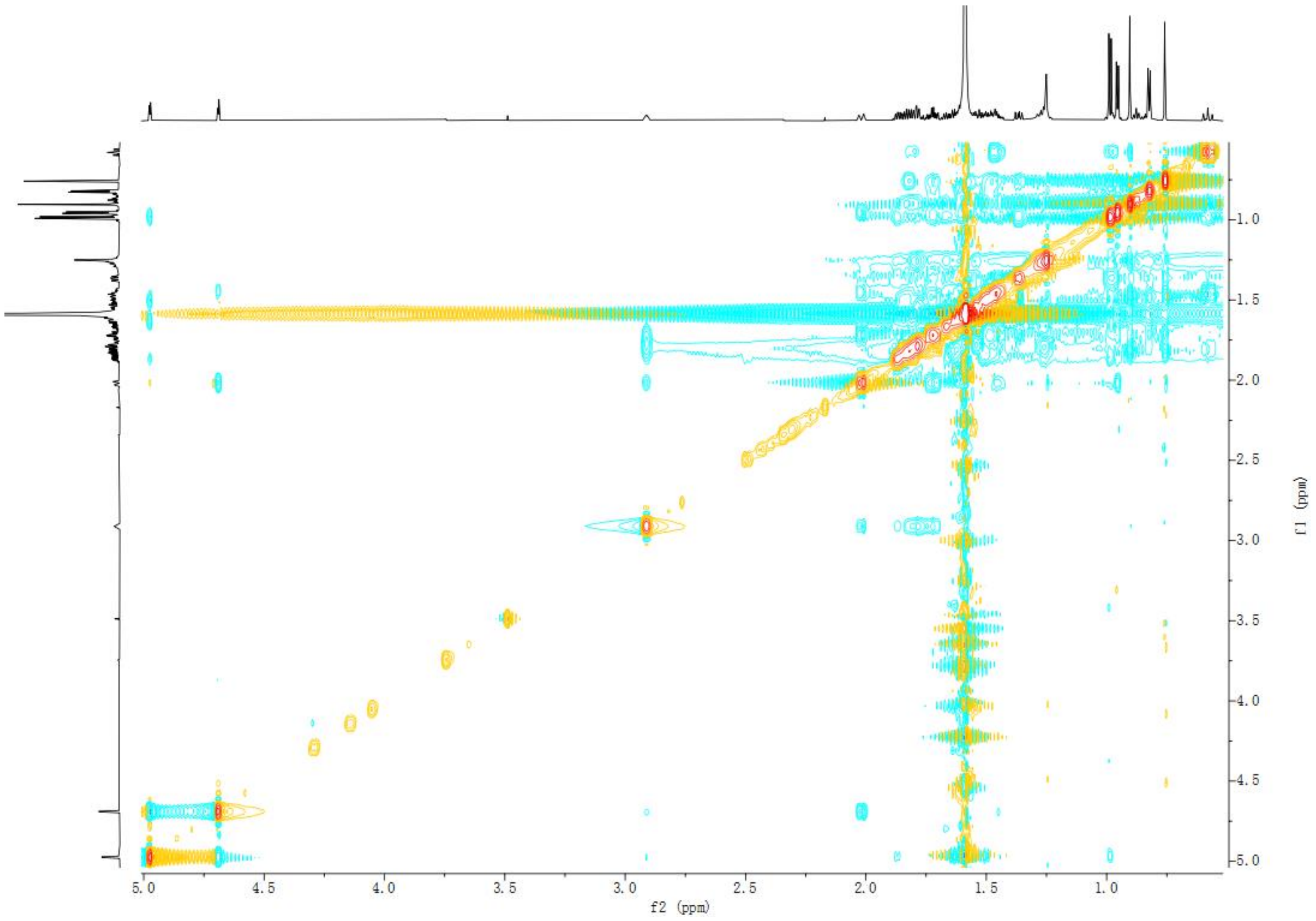

Figure S66. NOESY spectrum of compound **8** in $CDCl_3$

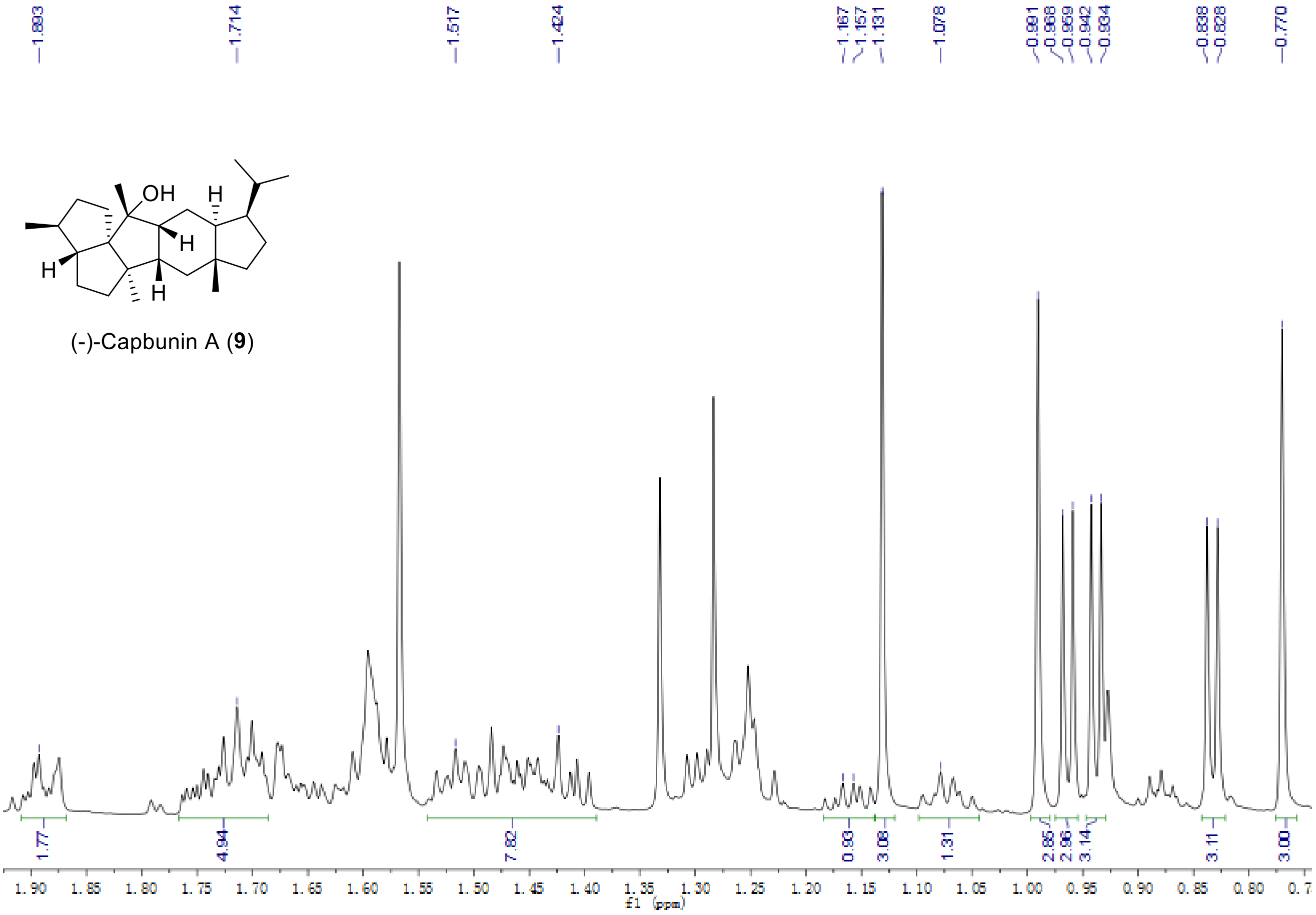

Figure S67. ^{1}H NMR spectrum of compound **9** in $CDCl_3$ (700 MHz)

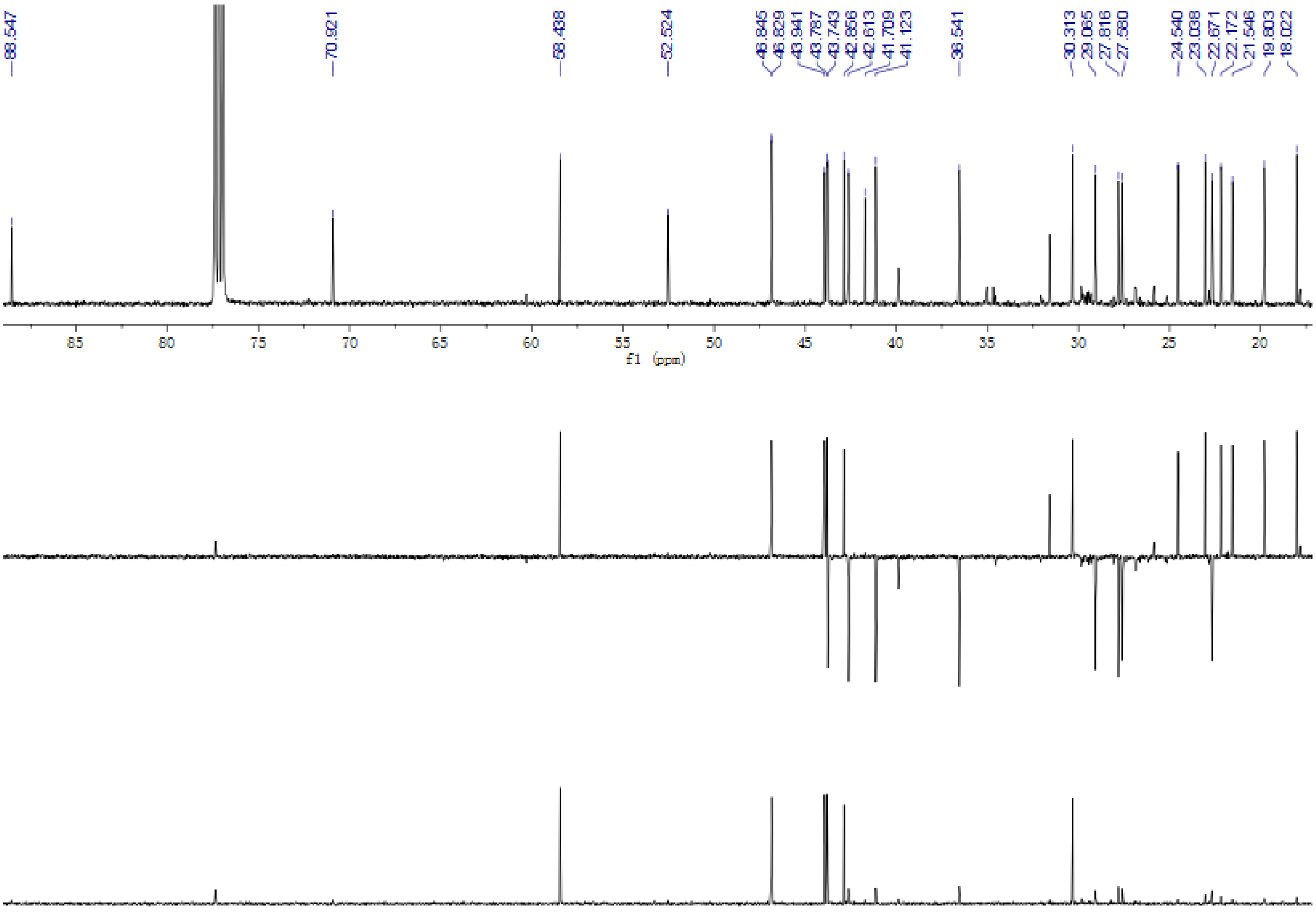

Figure S68. ^{13}C NMR and DEPT spectra of compound **9** in $CDCl_3$ (150 MHz)

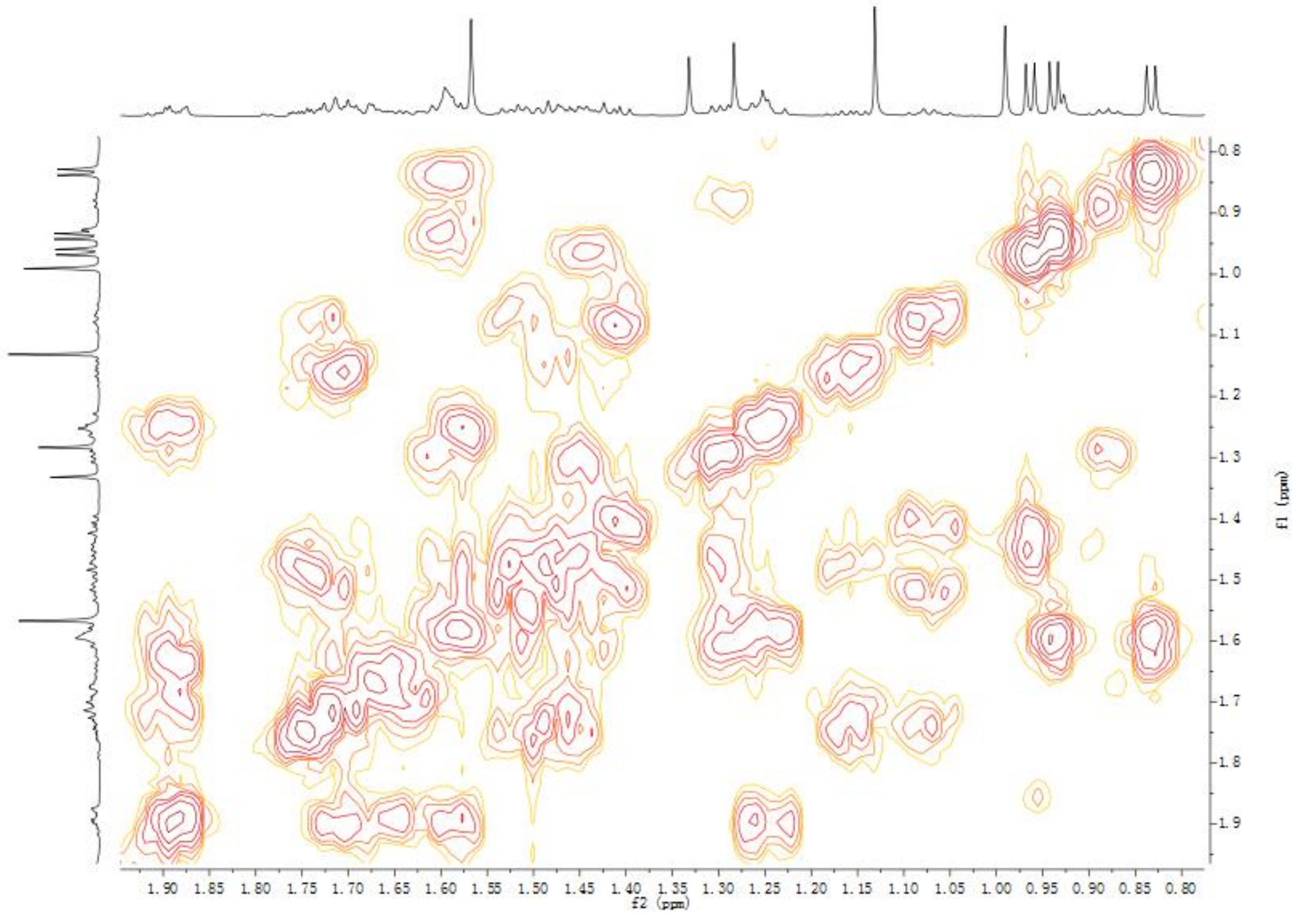

Figure S69. ^{1}H-^{1}H COSY spectrum of compound **9** in $CDCl_3$

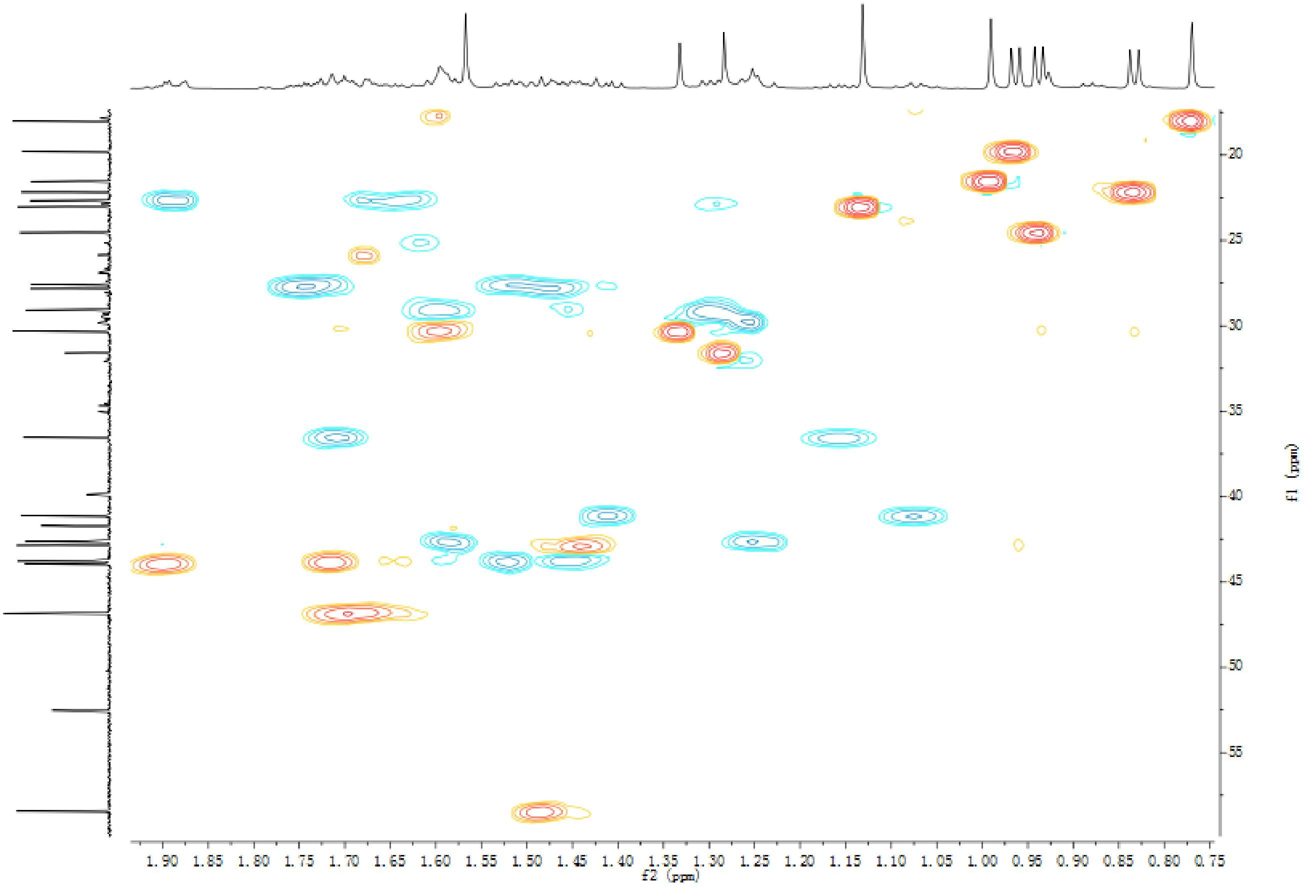

Figure S70. HSQC spectrum of compound **9** in $CDCl_3$

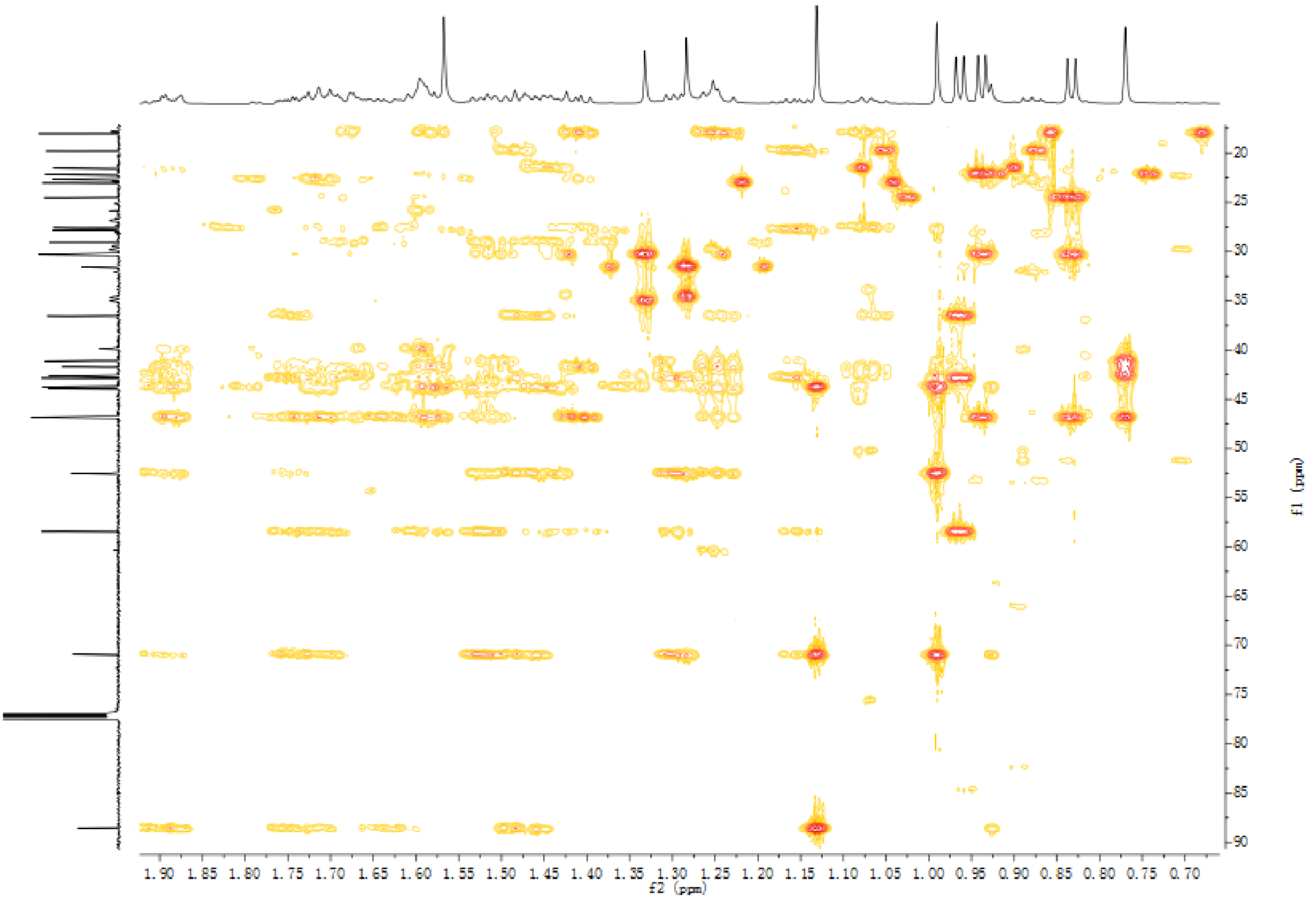

Figure S71. HMBC spectrum of compound **9** in $CDCl_3$

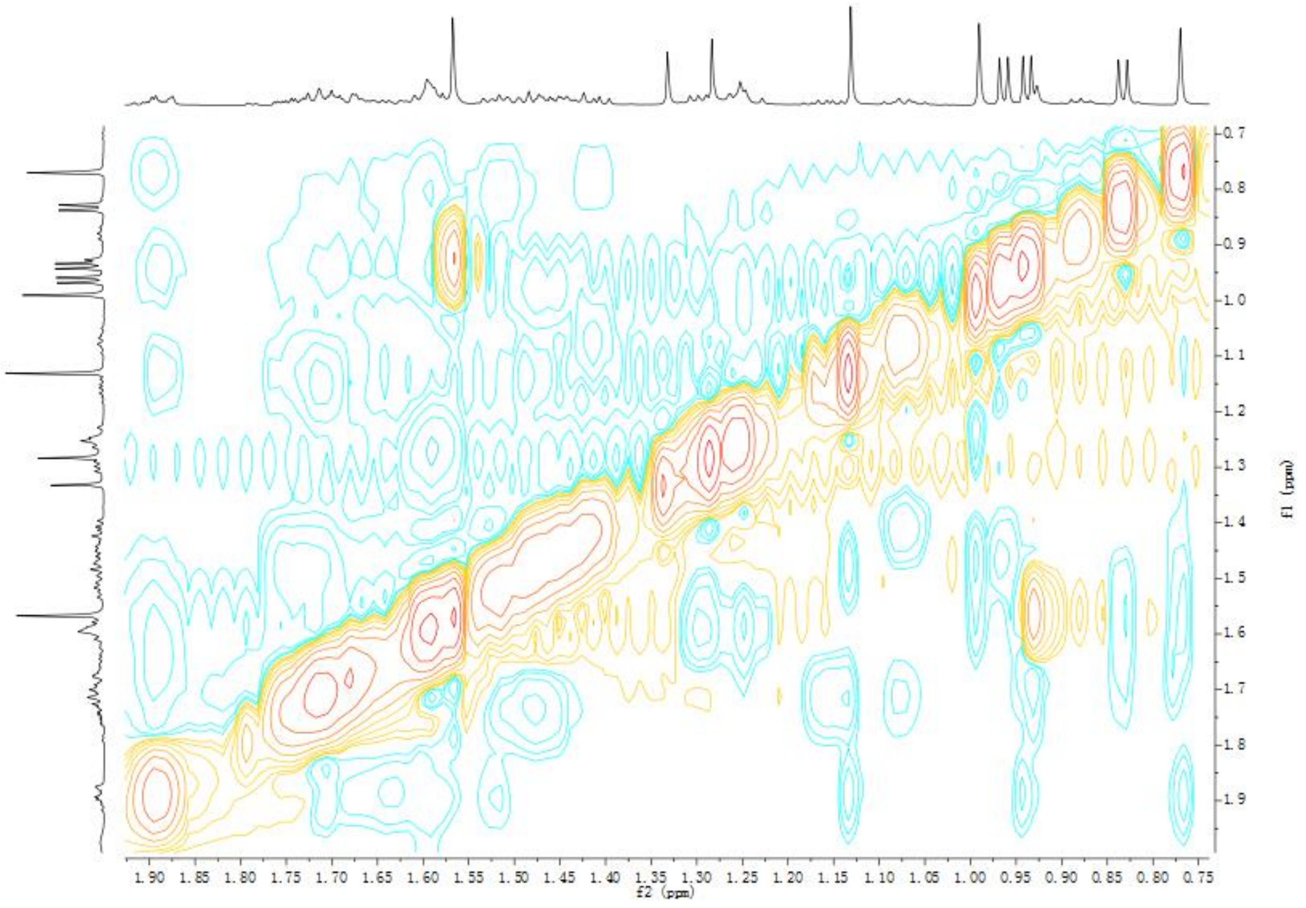

Figure S72. NOESY spectrum of compound **9** in $CDCl_3$

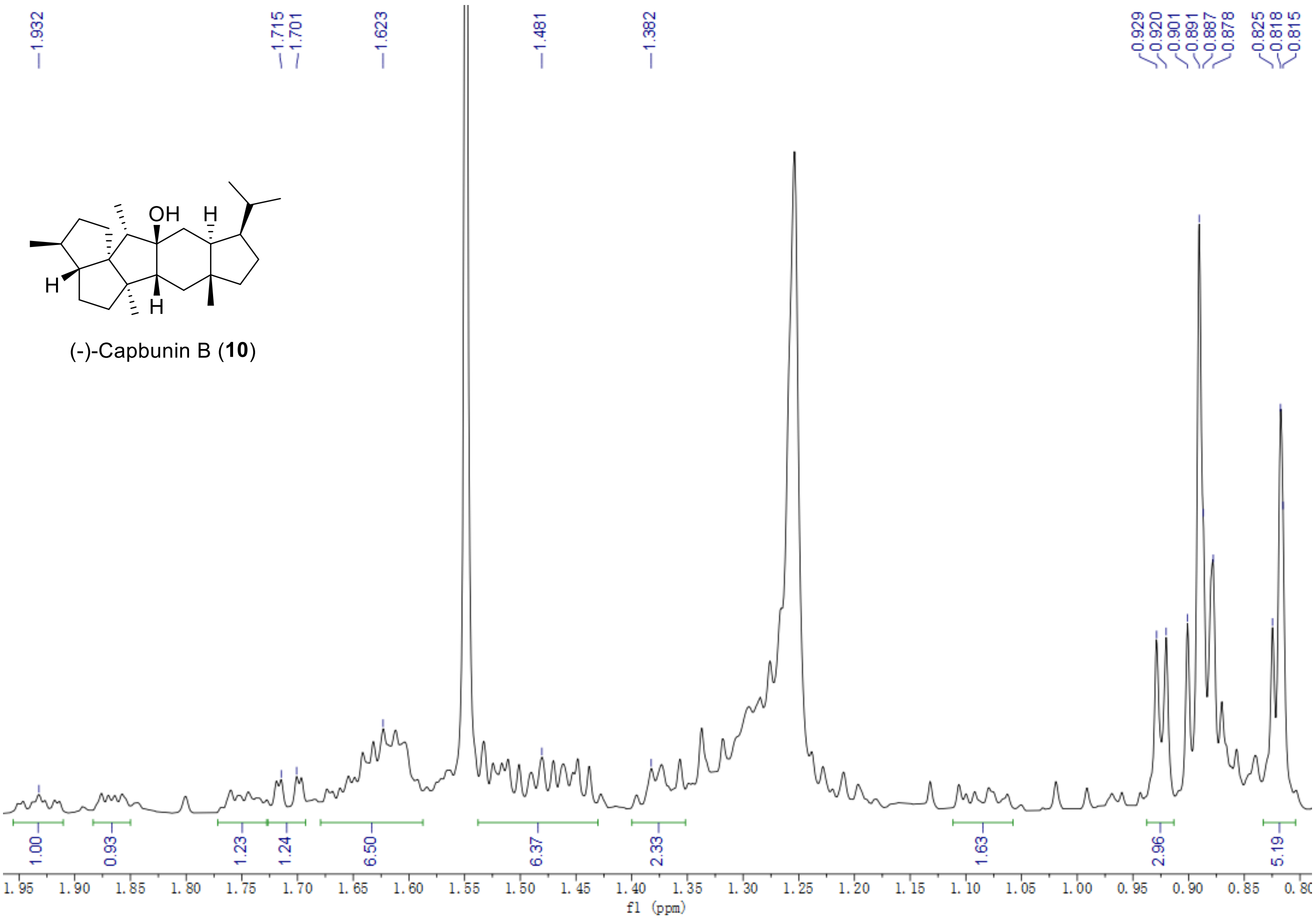

Figure S73. ^{1}H NMR spectrum of compound **10** in $CDCl_3$ (700 MHz)

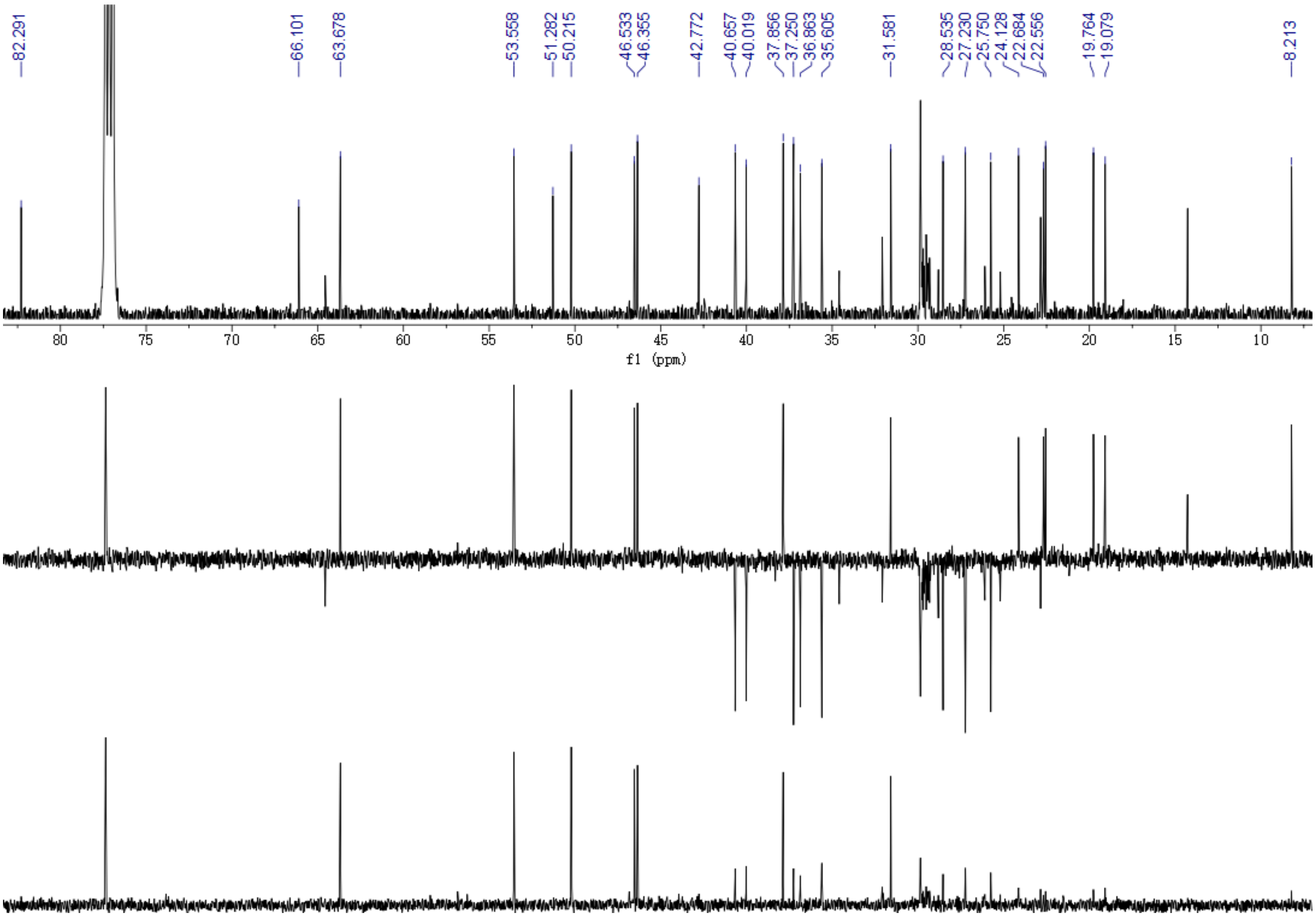

Figure S74. ^{13}C NMR and DEPT spectra of compound **10** in $CDCl_3$ (150 MHz)

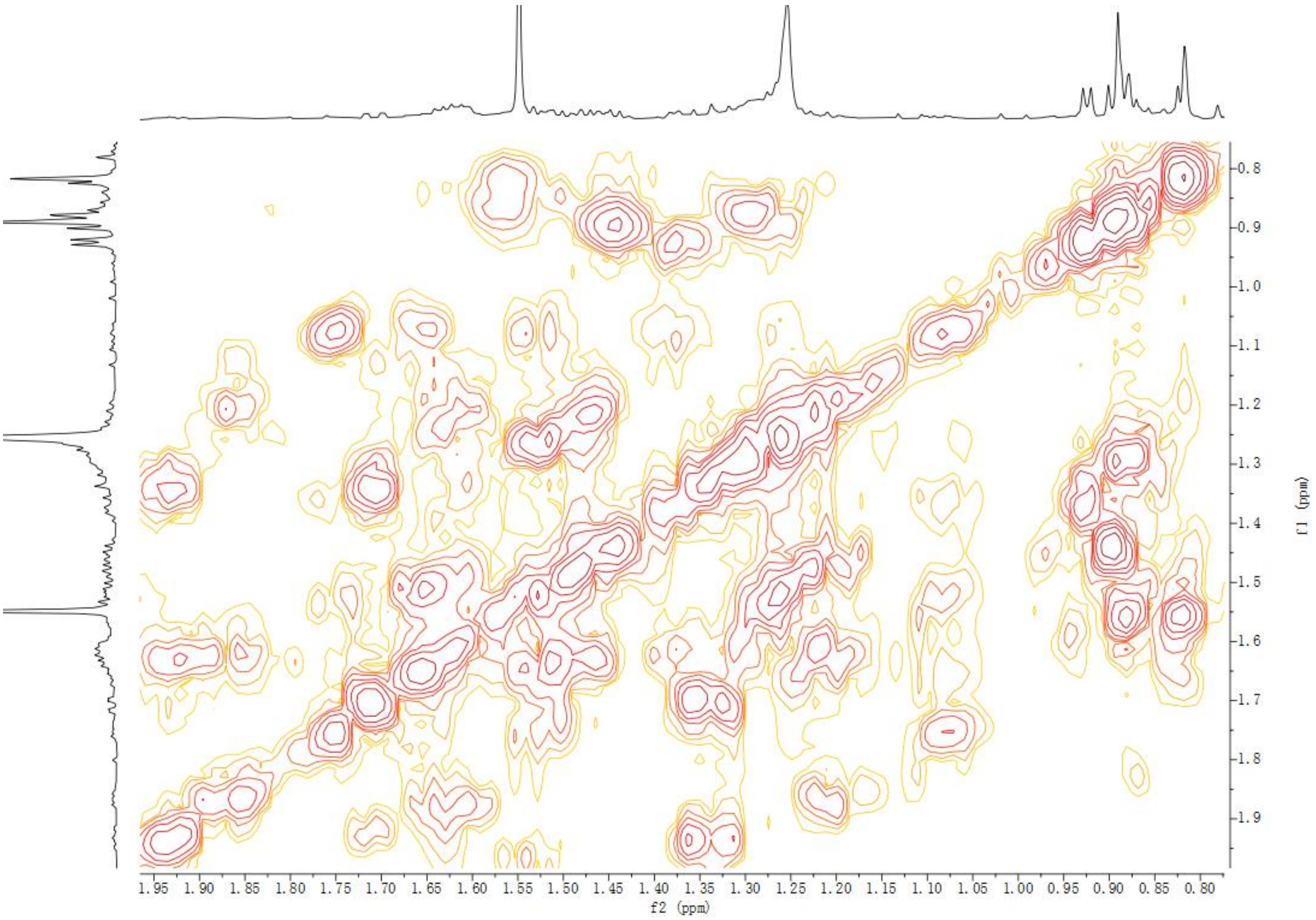

Figure S75. ^{1}H-^{1}H COSY spectrum of compound **10** in $CDCl_3$

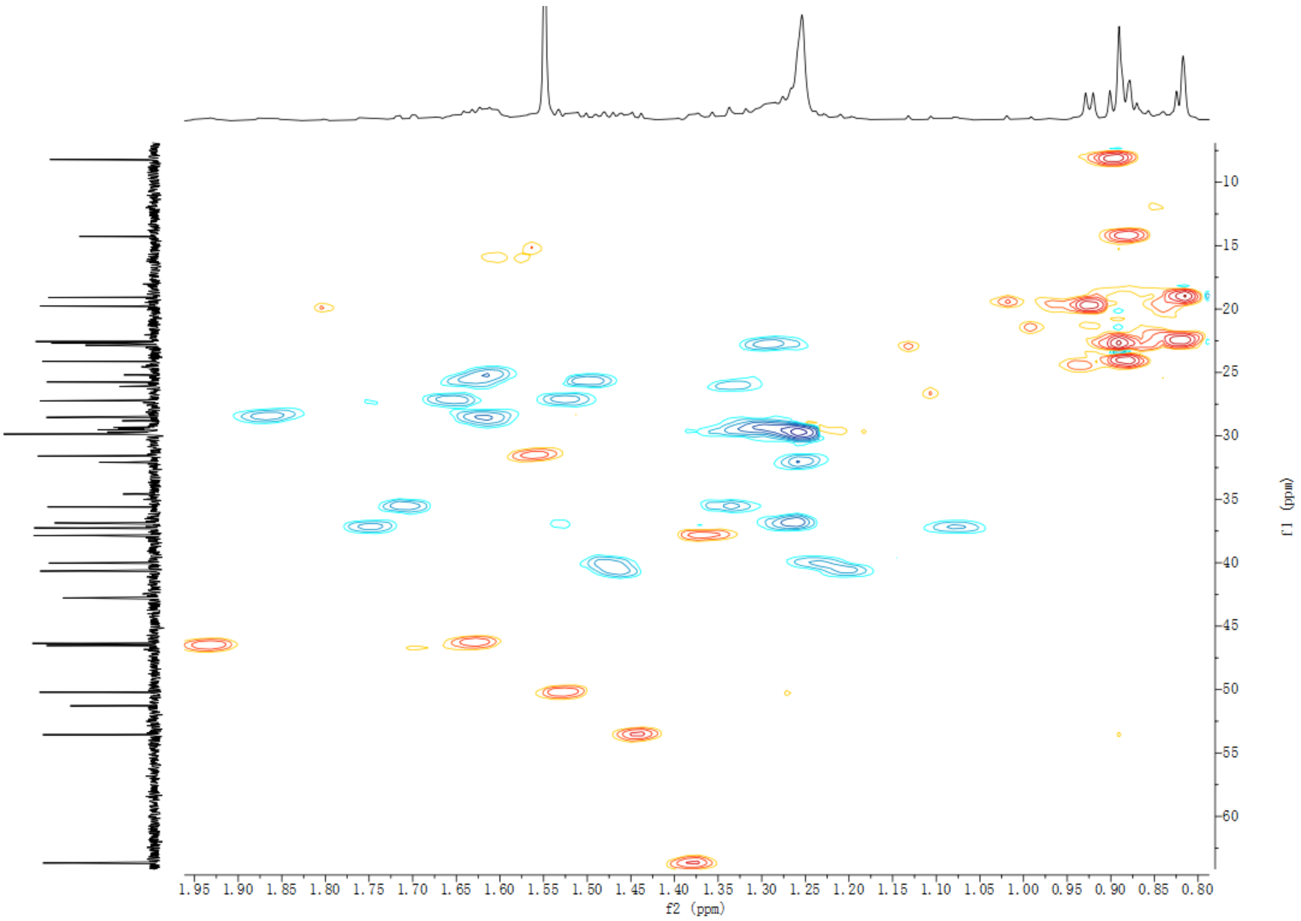

Figure S76. HSQC spectrum of compound **10** in $CDCl_3$

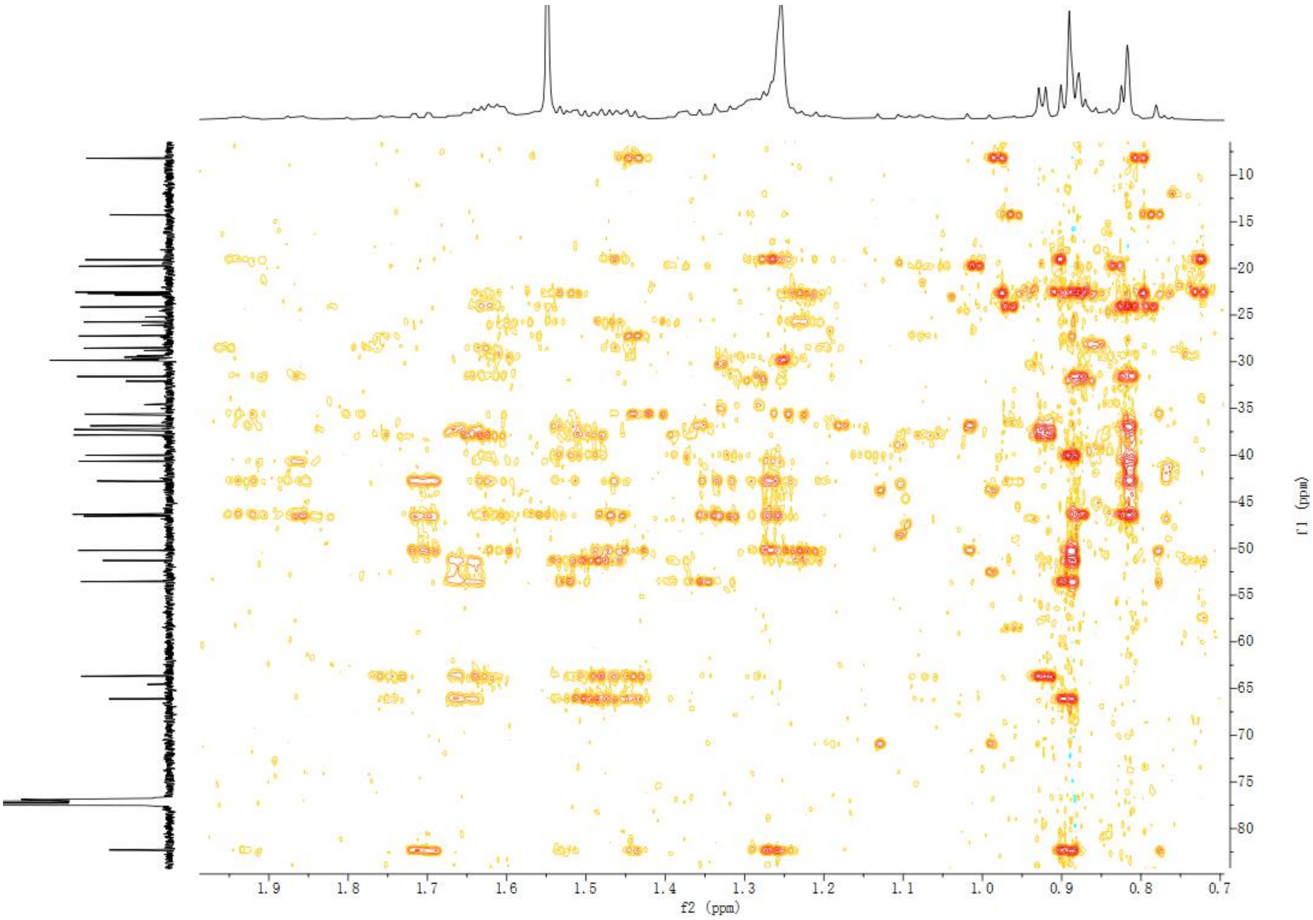

Figure S77. HMBC spectrum of compound **10** in $CDCl_3$

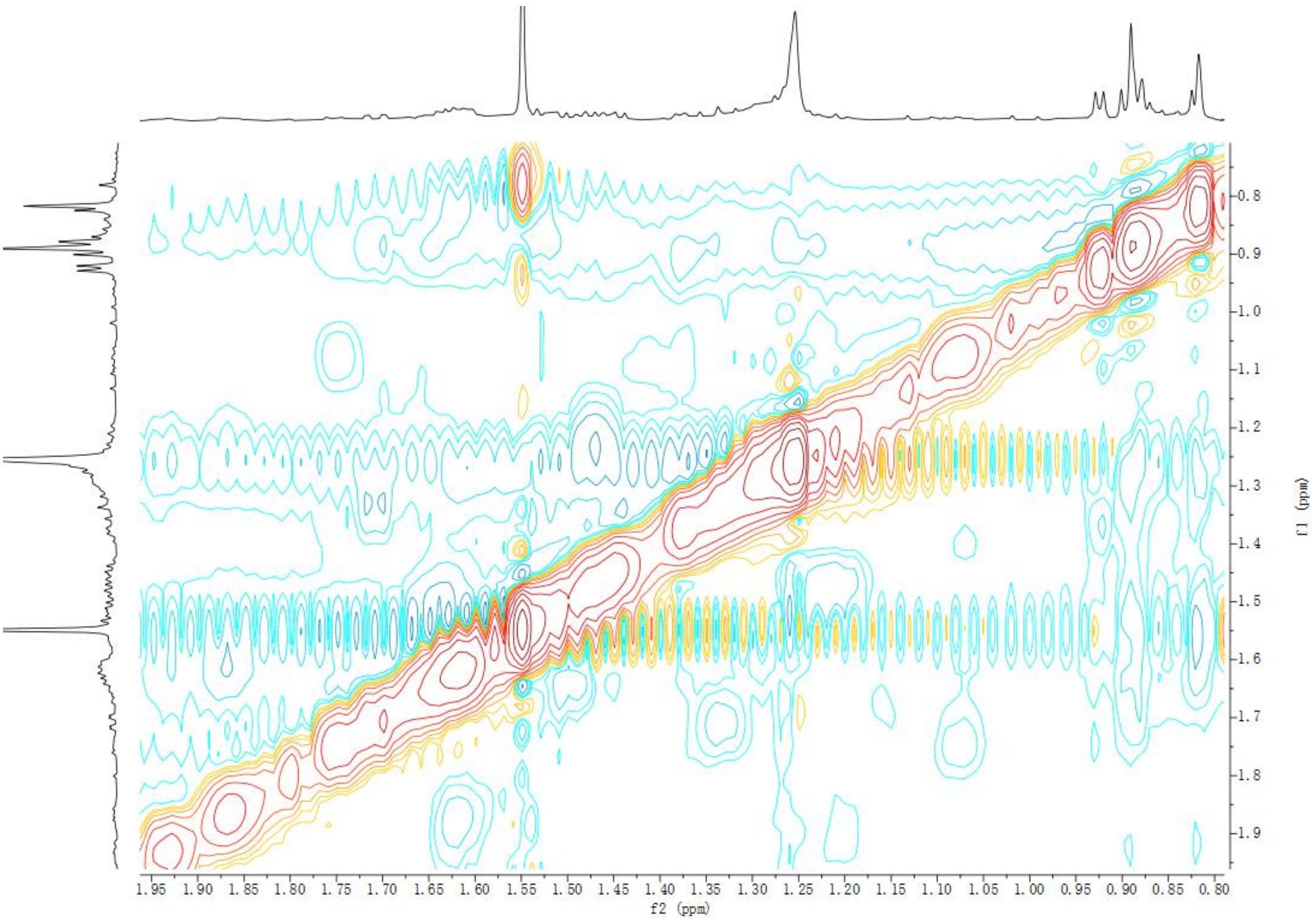

Figure S78. NOESY spectrum of compound **10** in $CDCl_3$

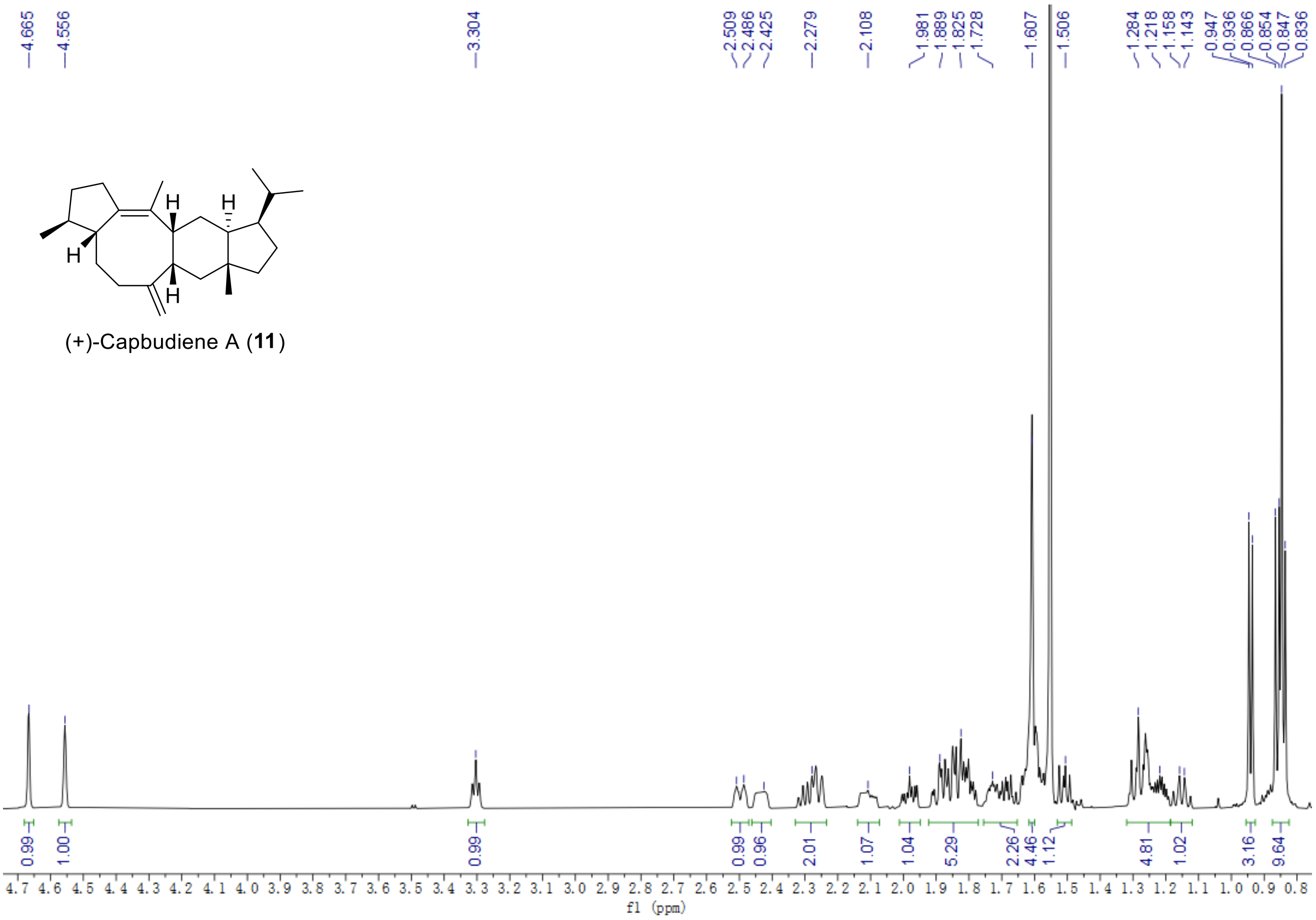

Figure S79. ^{1}H NMR spectrum of compound **11** in $CDCl_3$ (700 MHz)

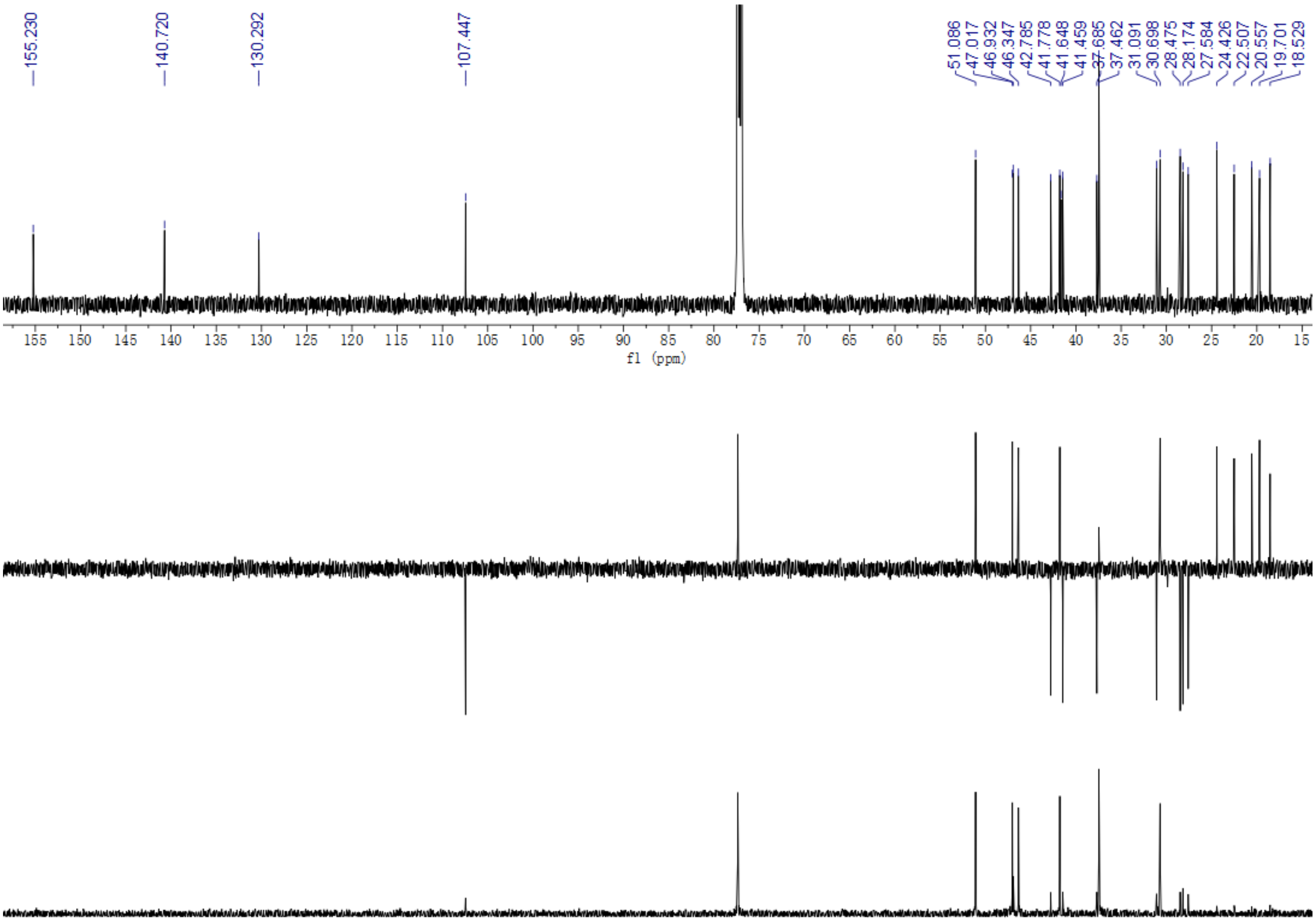

Figure S80. ^{13}C NMR and DEPT spectra of compound **11** in $CDCl_3$ (150 MHz)

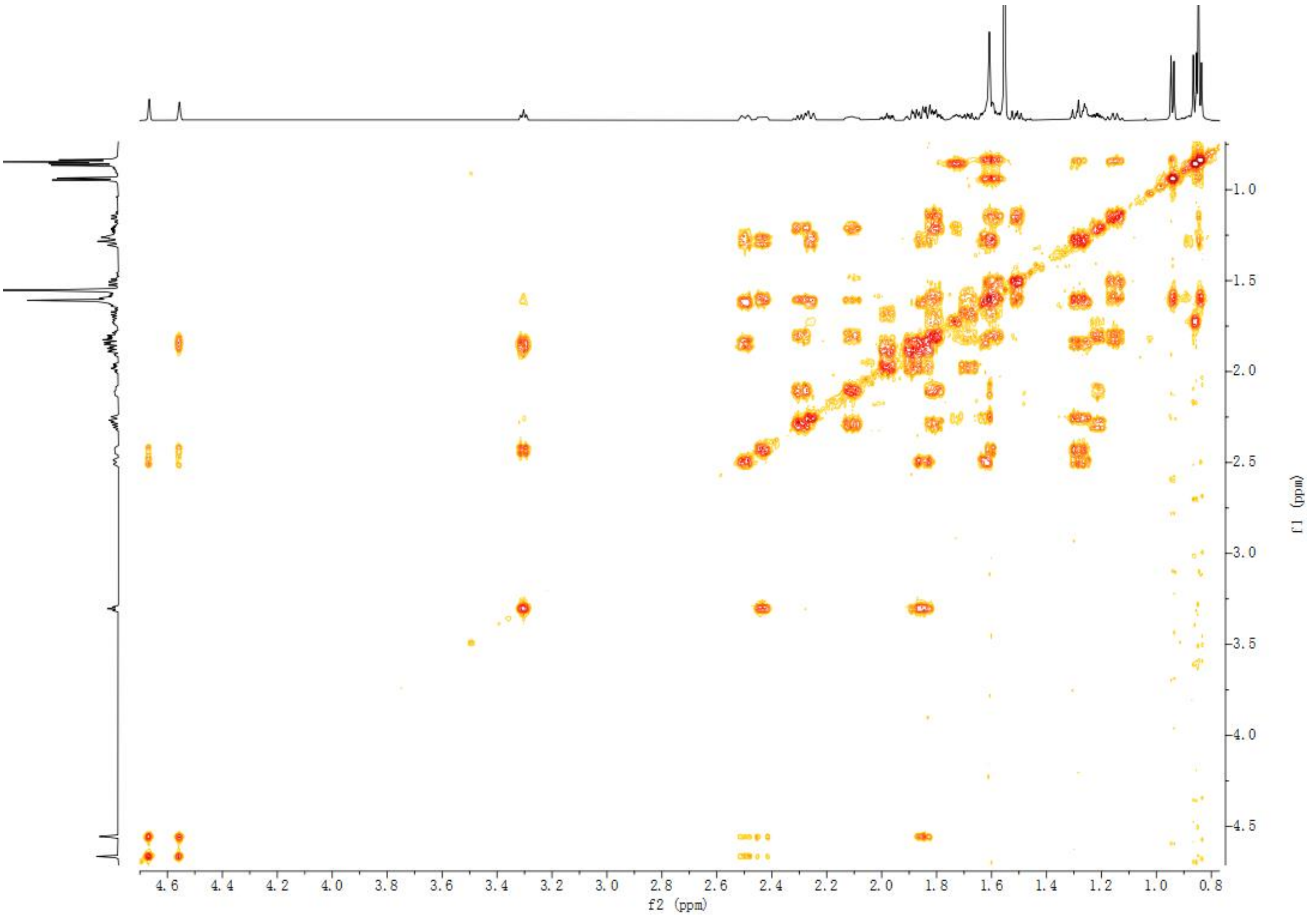

Figure S81. ^{1}H-^{1}H COSY spectrum of compound **11** in $CDCl_3$

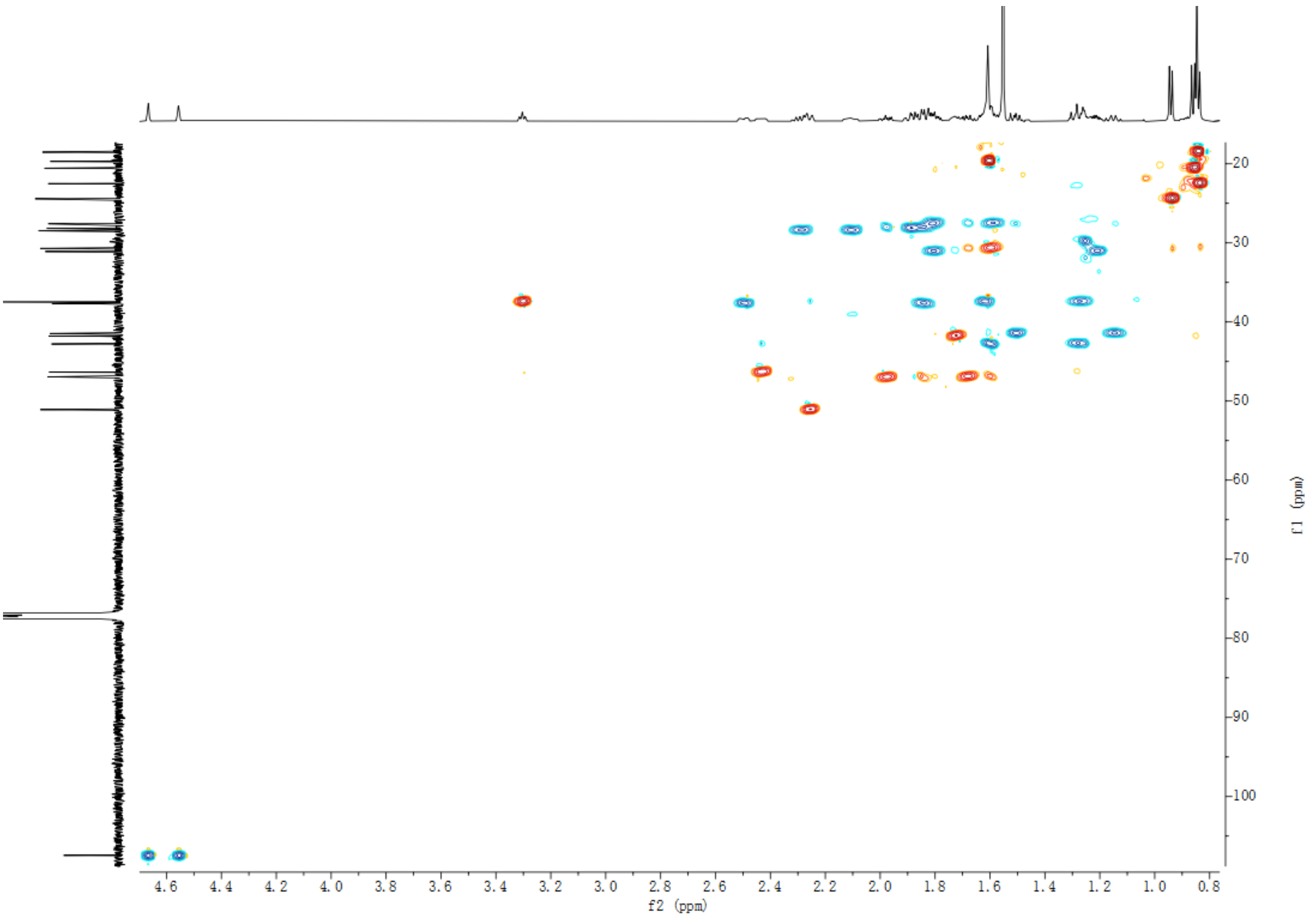

Figure S82. HSQC spectrum of compound **11** in $CDCl_3$

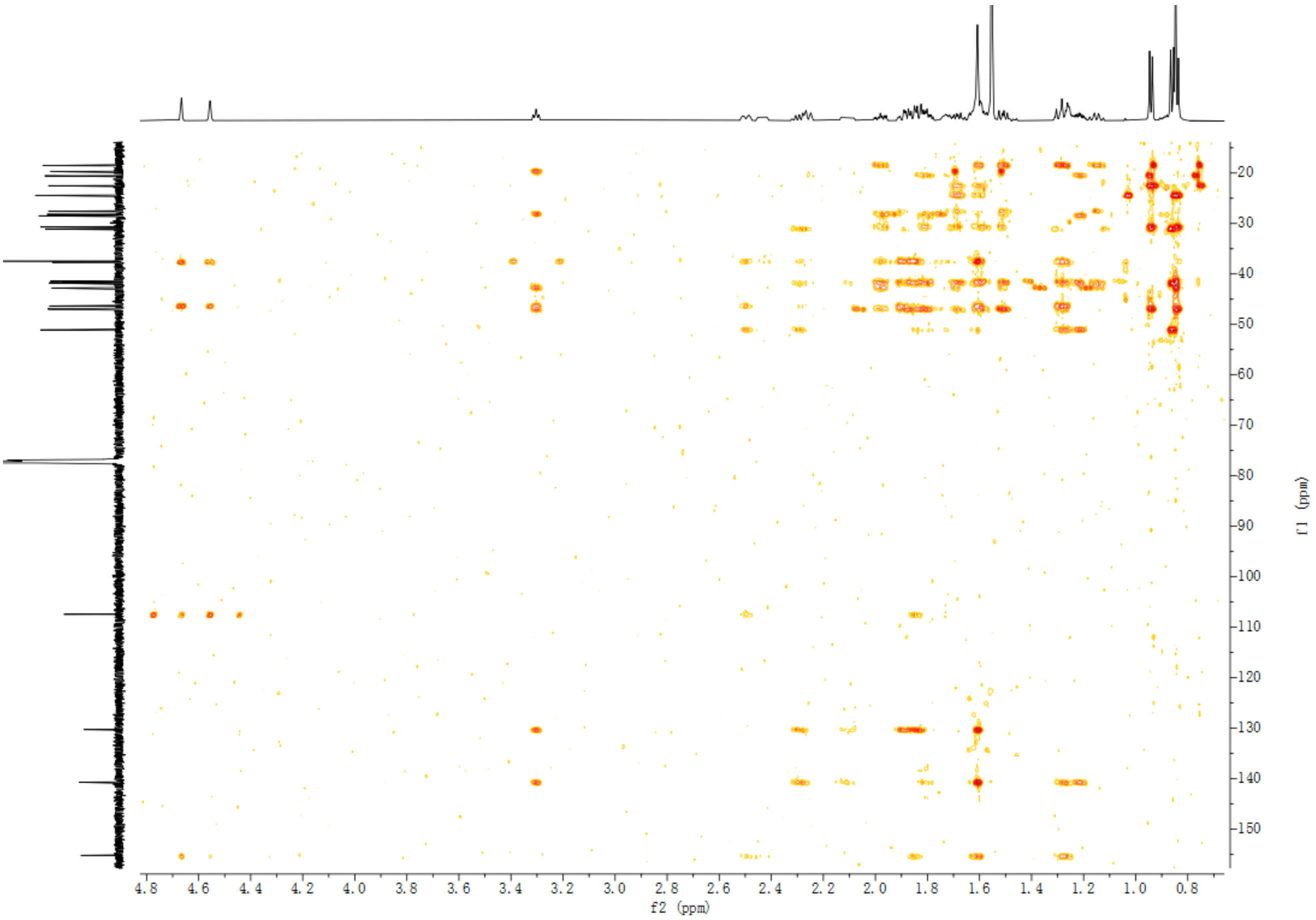

Figure S83. HMBC spectrum of compound **11** in $CDCl_3$

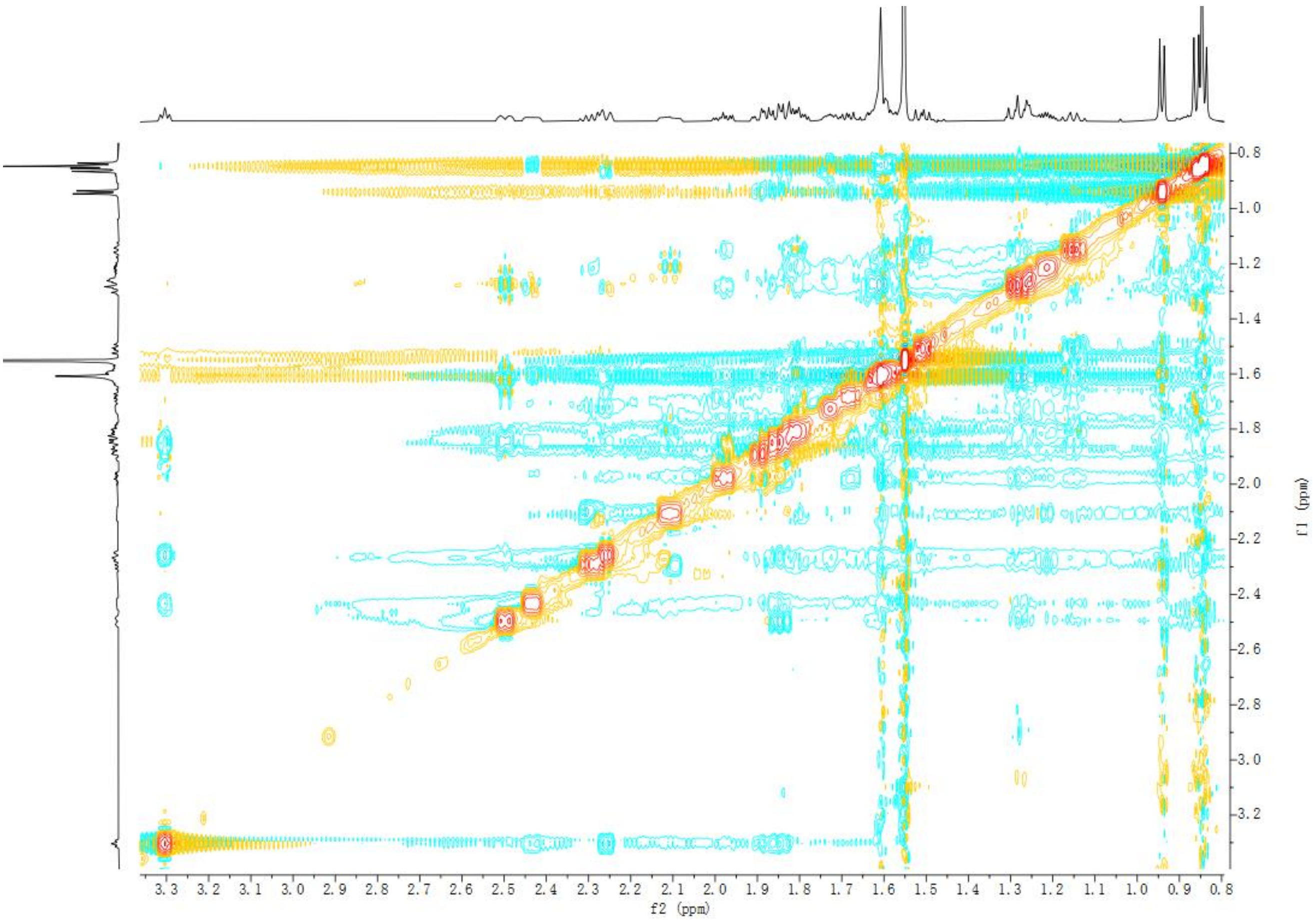

Figure S84. NOESY spectrum of compound **11** in $CDCl_3$

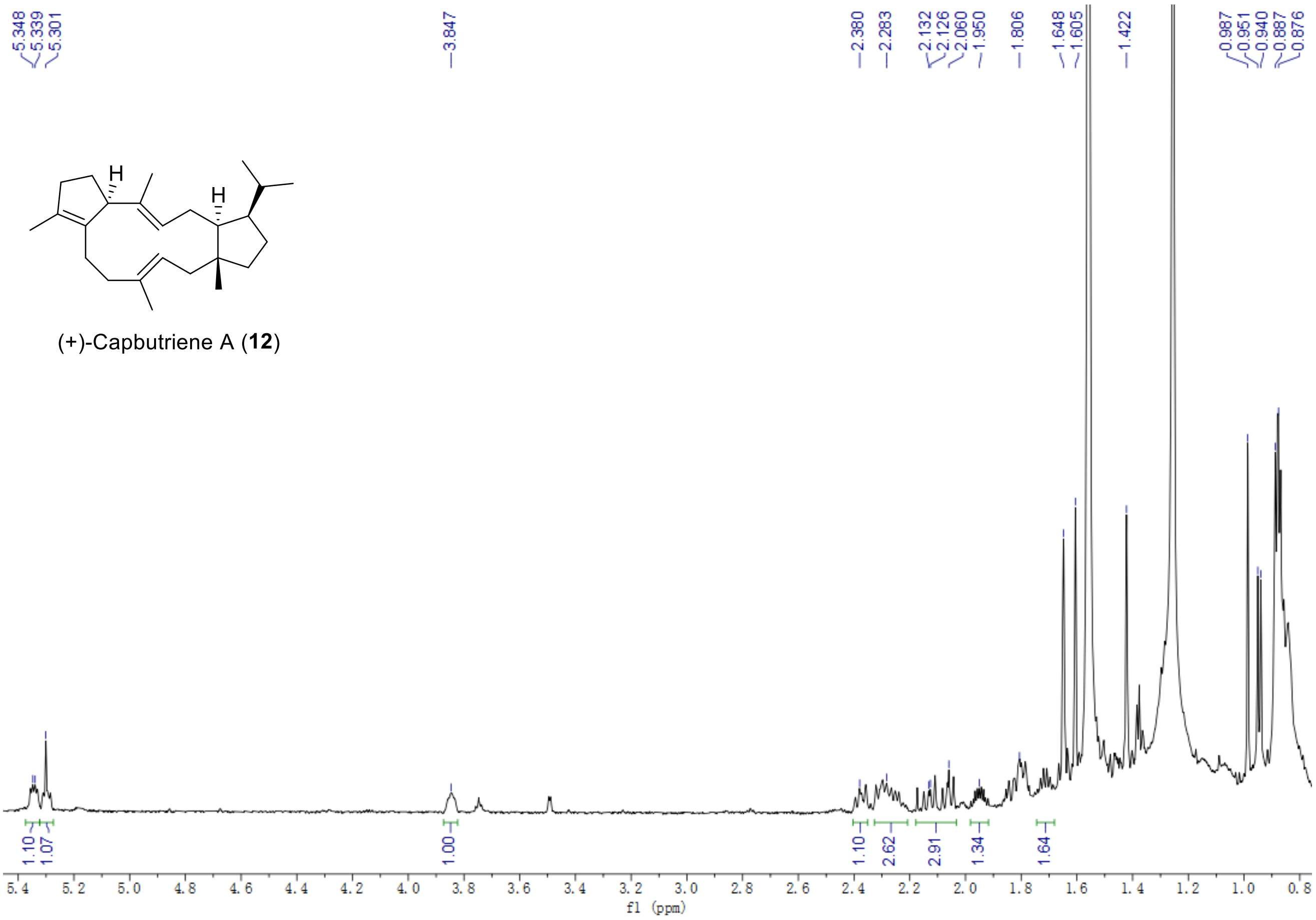

Figure S85. ^{1}H NMR spectrum of compound **12** in $CDCl_3$ (700 MHz)

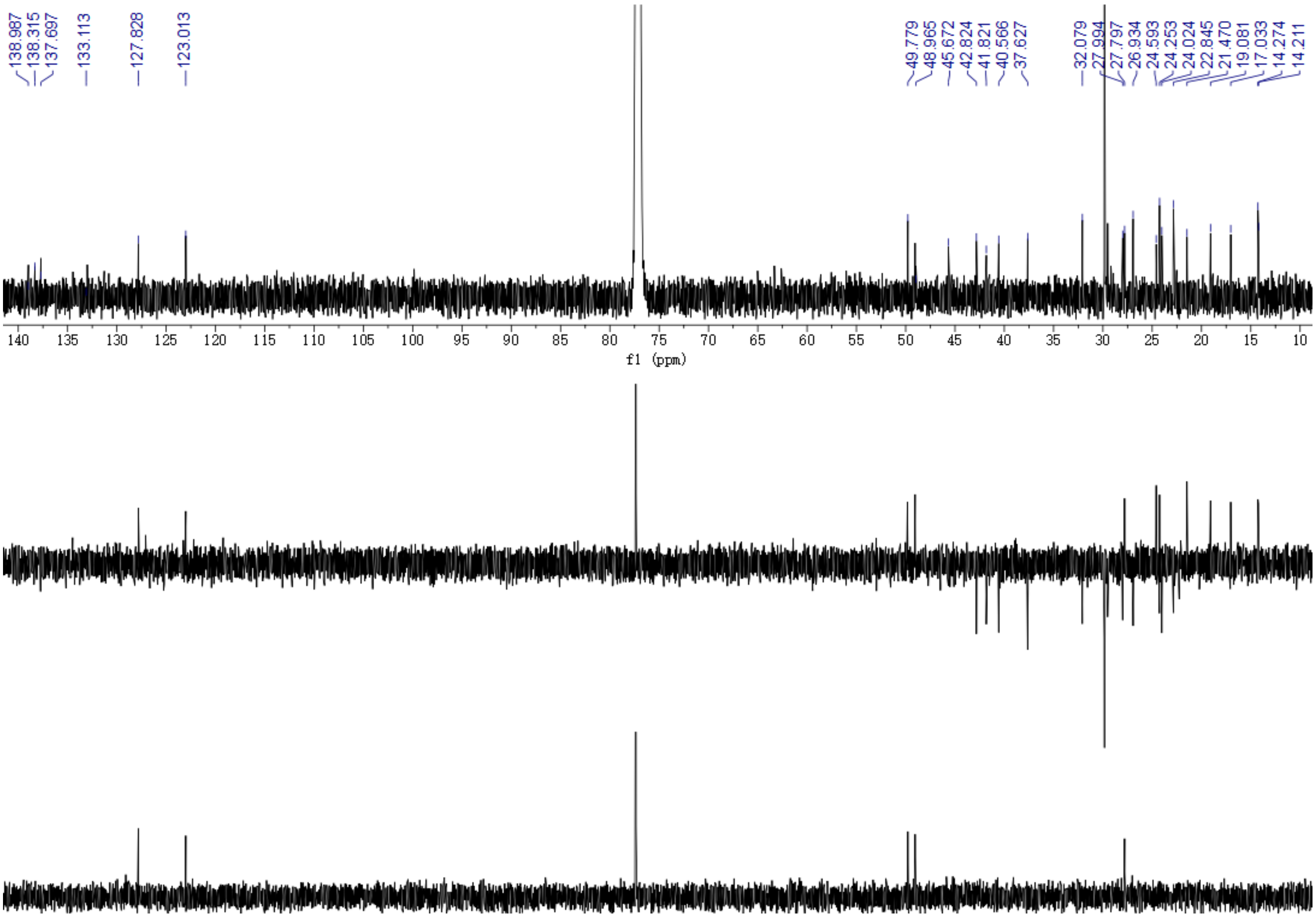

Figure S86. ^{13}C NMR and DEPT spectra of compound **12** in $CDCl_3$ (150 MHz)

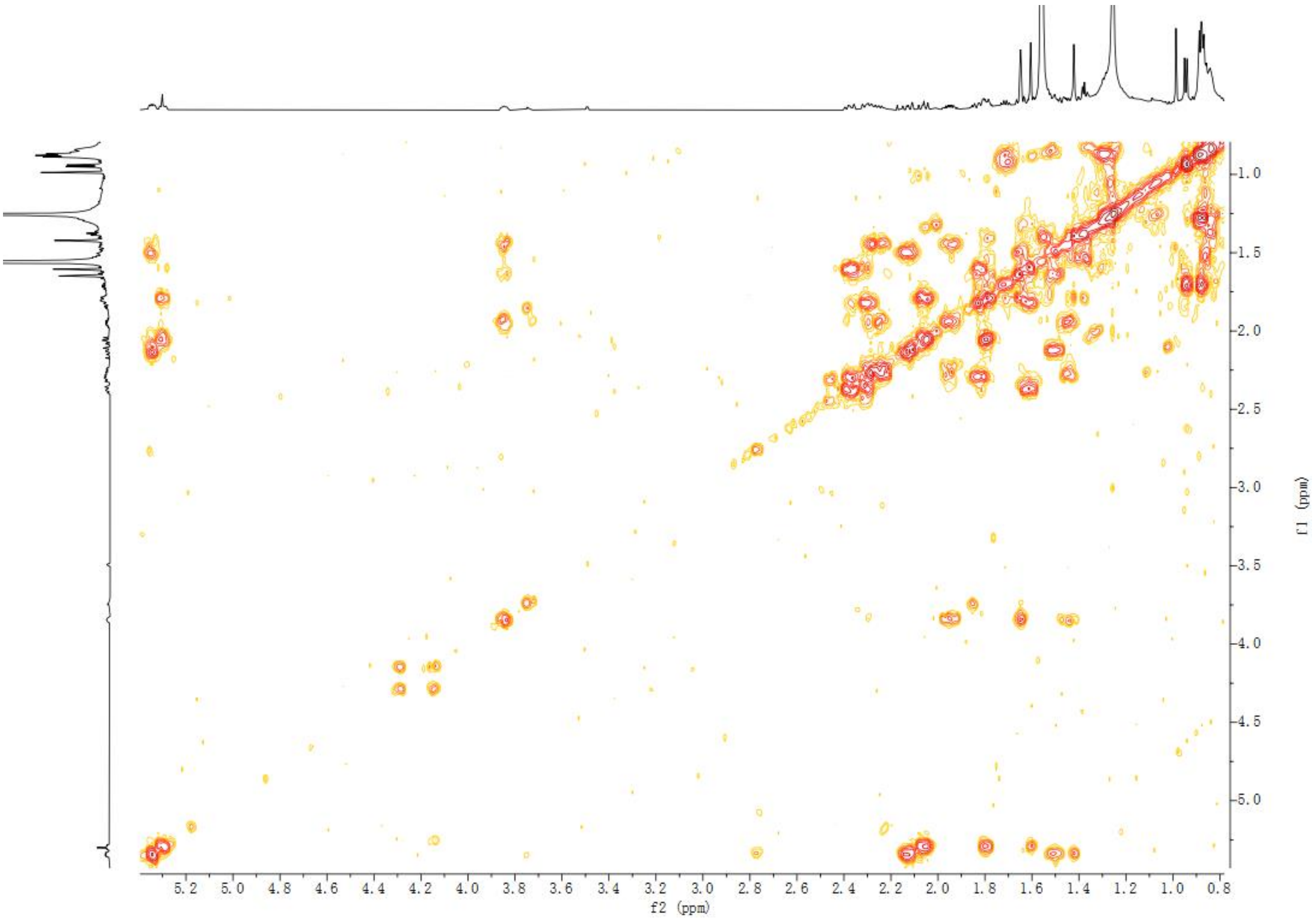

Figure S87. ^{1}H-^{1}H COSY spectrum of compound **12** in $CDCl_3$

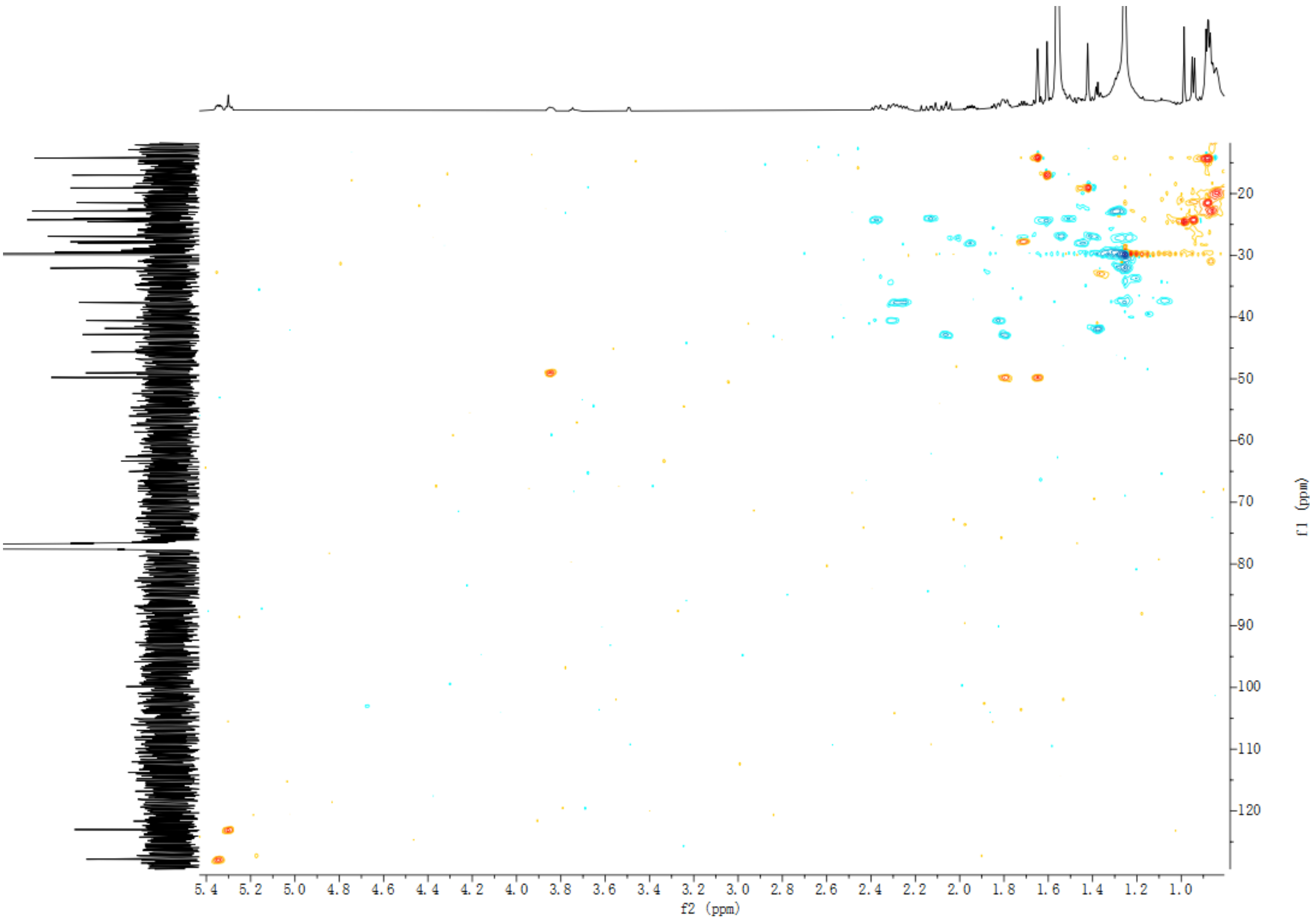

Figure S88. HSQC spectrum of compound **12** in $CDCl_3$

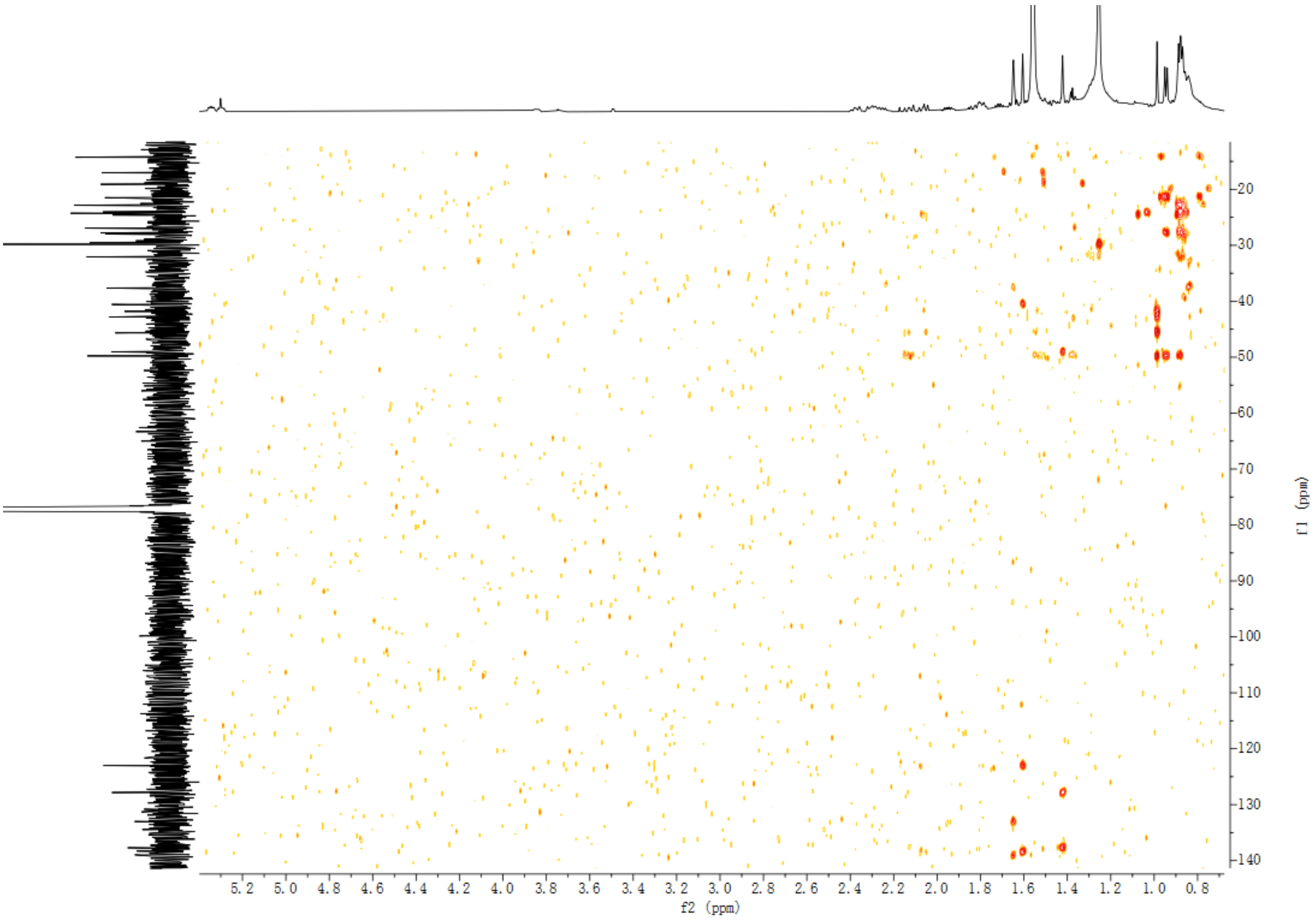

Figure S89. HMBC spectrum of compound **12** in $CDCl_3$

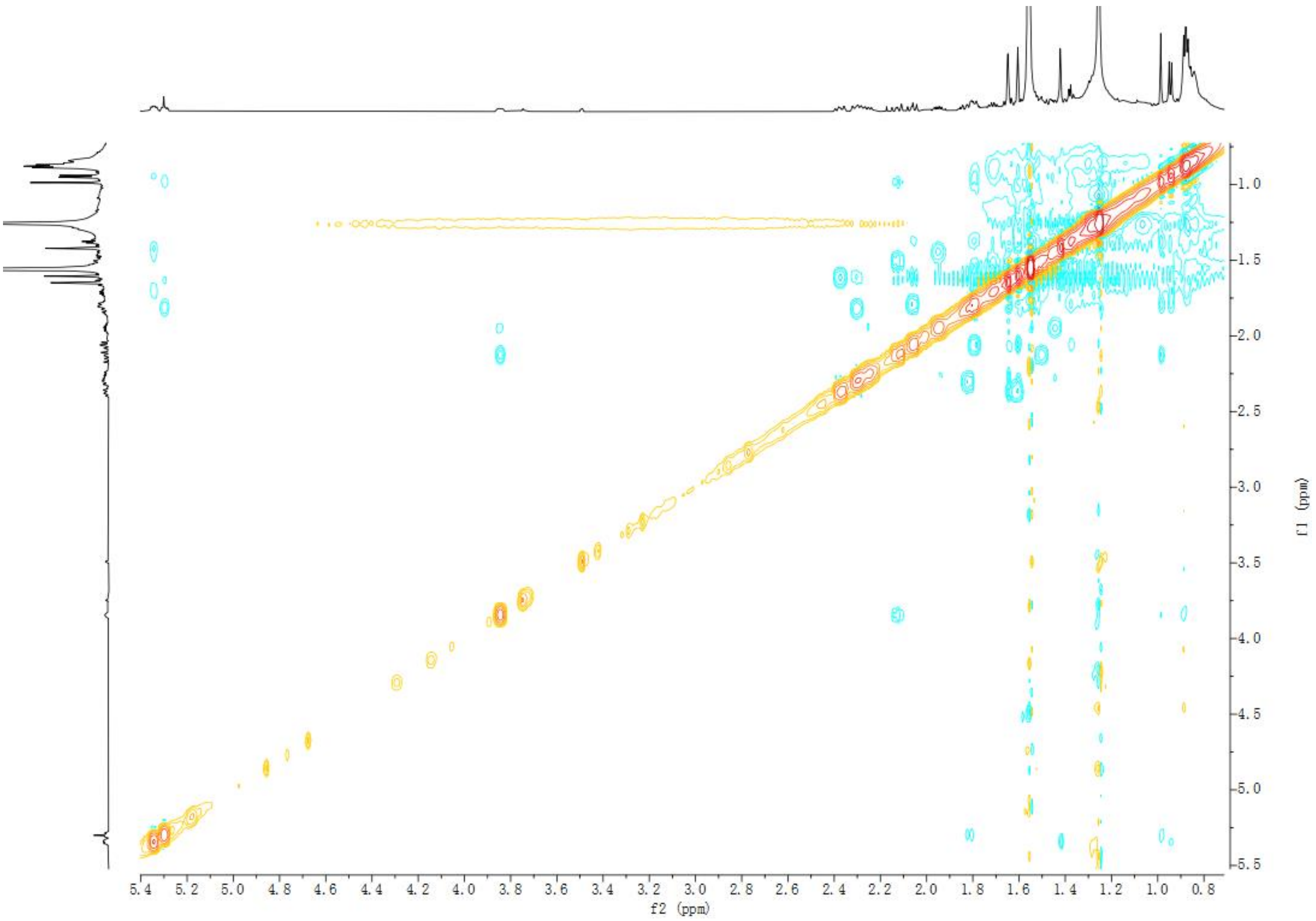

Figure S90. NOESY spectrum of compound **12** in $CDCl_3$

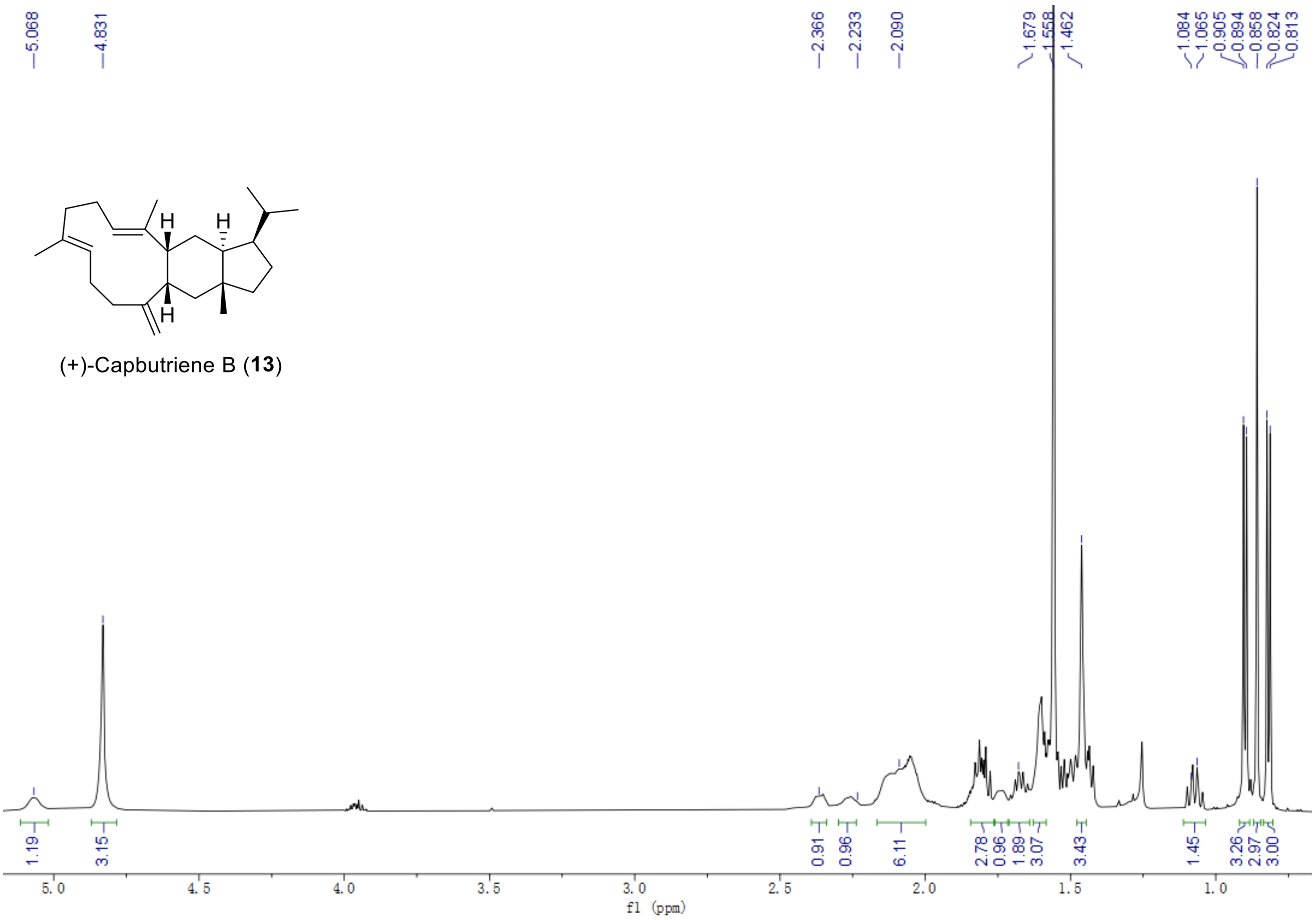

Figure S91. ^{1}H NMR spectrum of compound **13** in $CDCl_3$ (700 MHz)

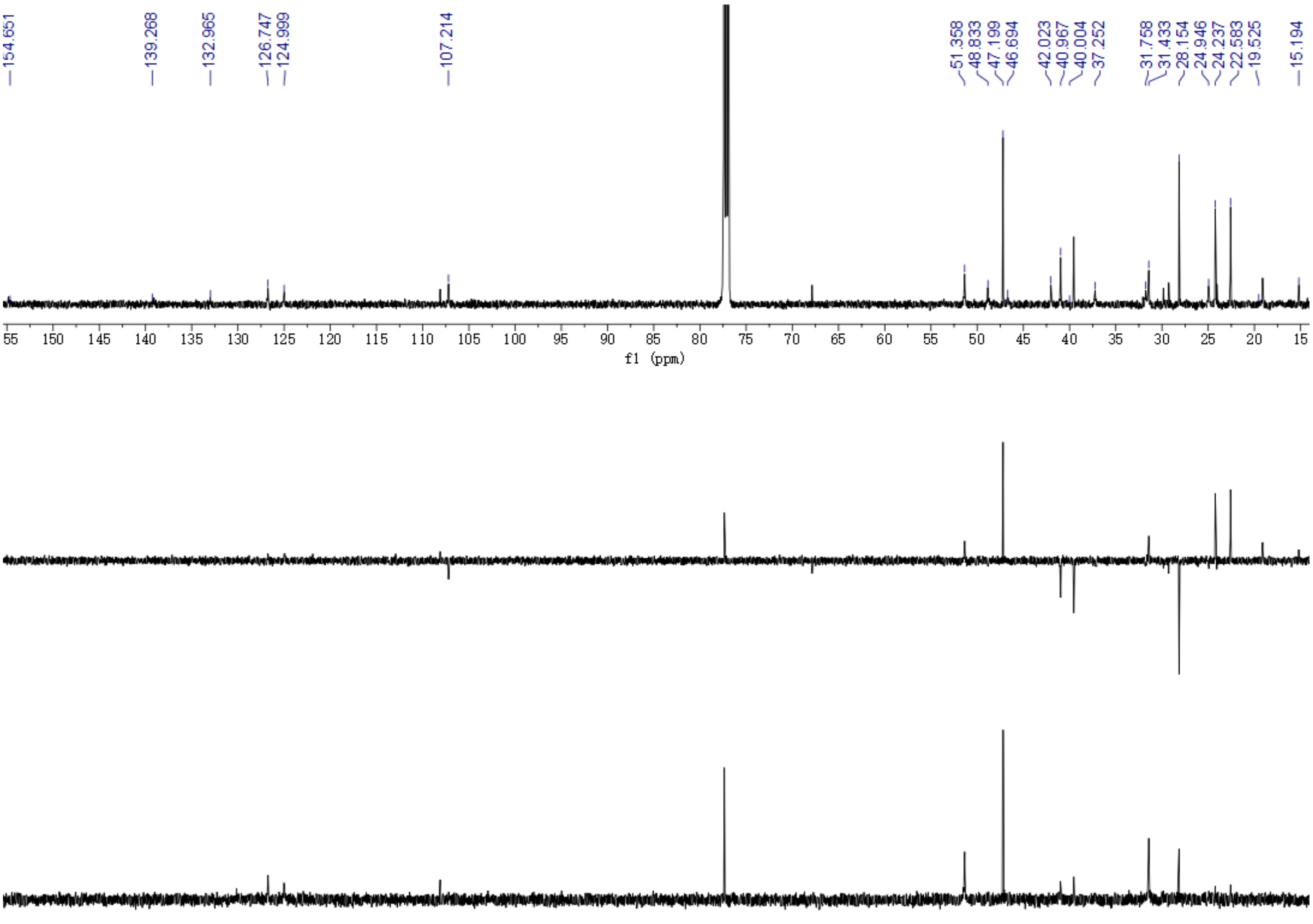

Figure S92. ^{13}C NMR and DEPT spectra of compound **13** in $CDCl_3$ (150 MHz)

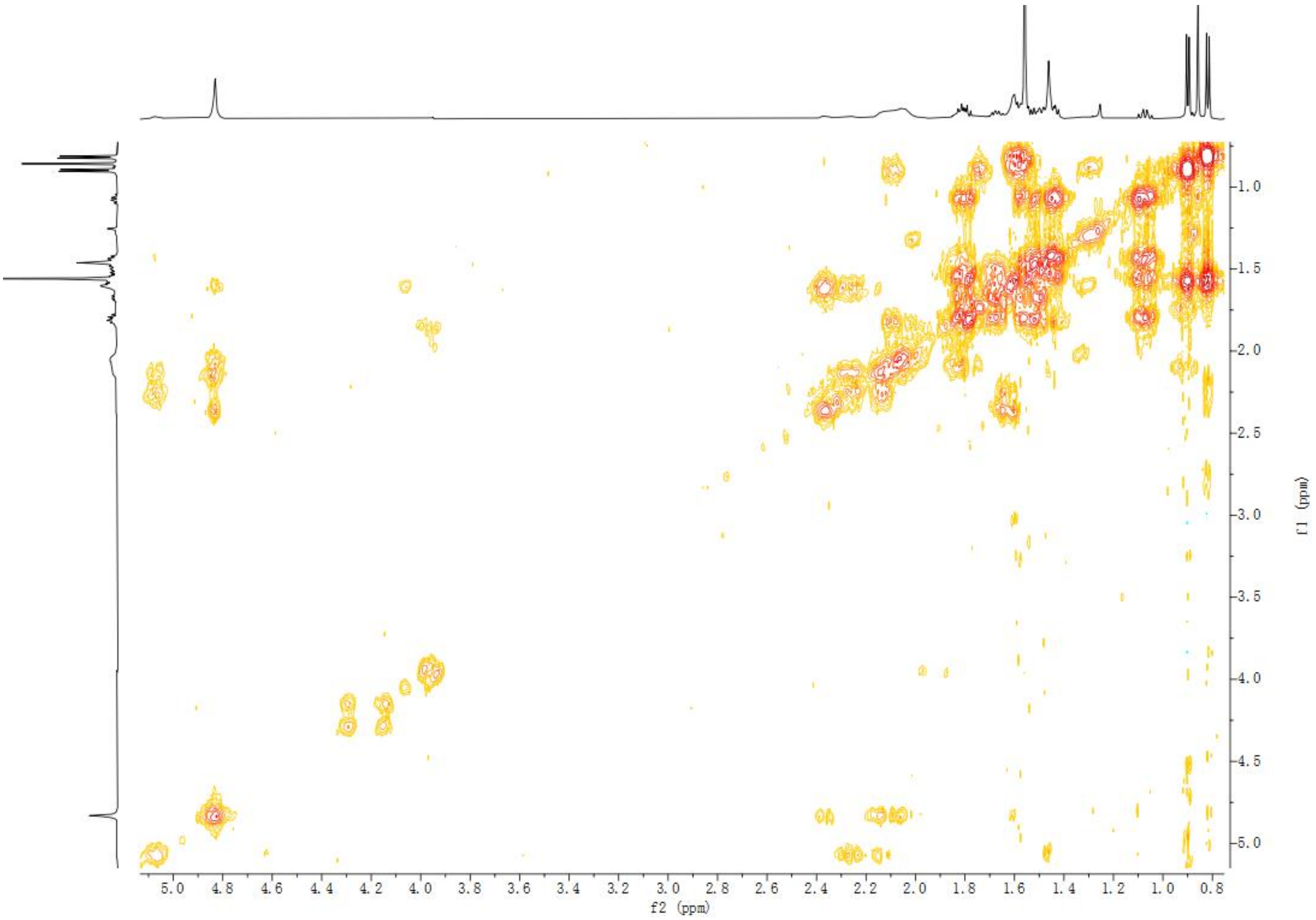

Figure S93. ^{1}H-^{1}H COSY spectrum of compound **13** in $CDCl_3$

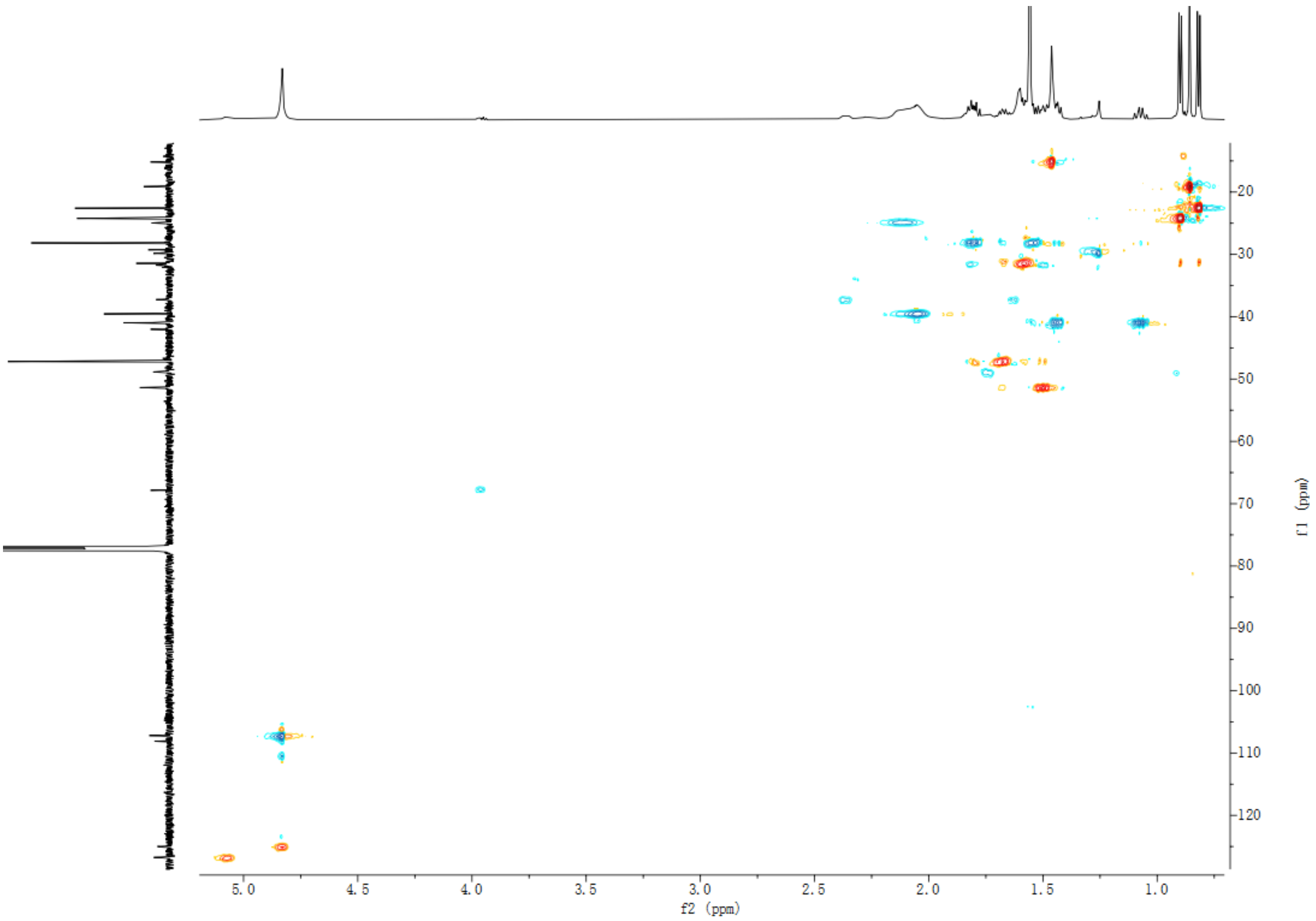

Figure S94. HSQC spectrum of compound **13** in $CDCl_3$

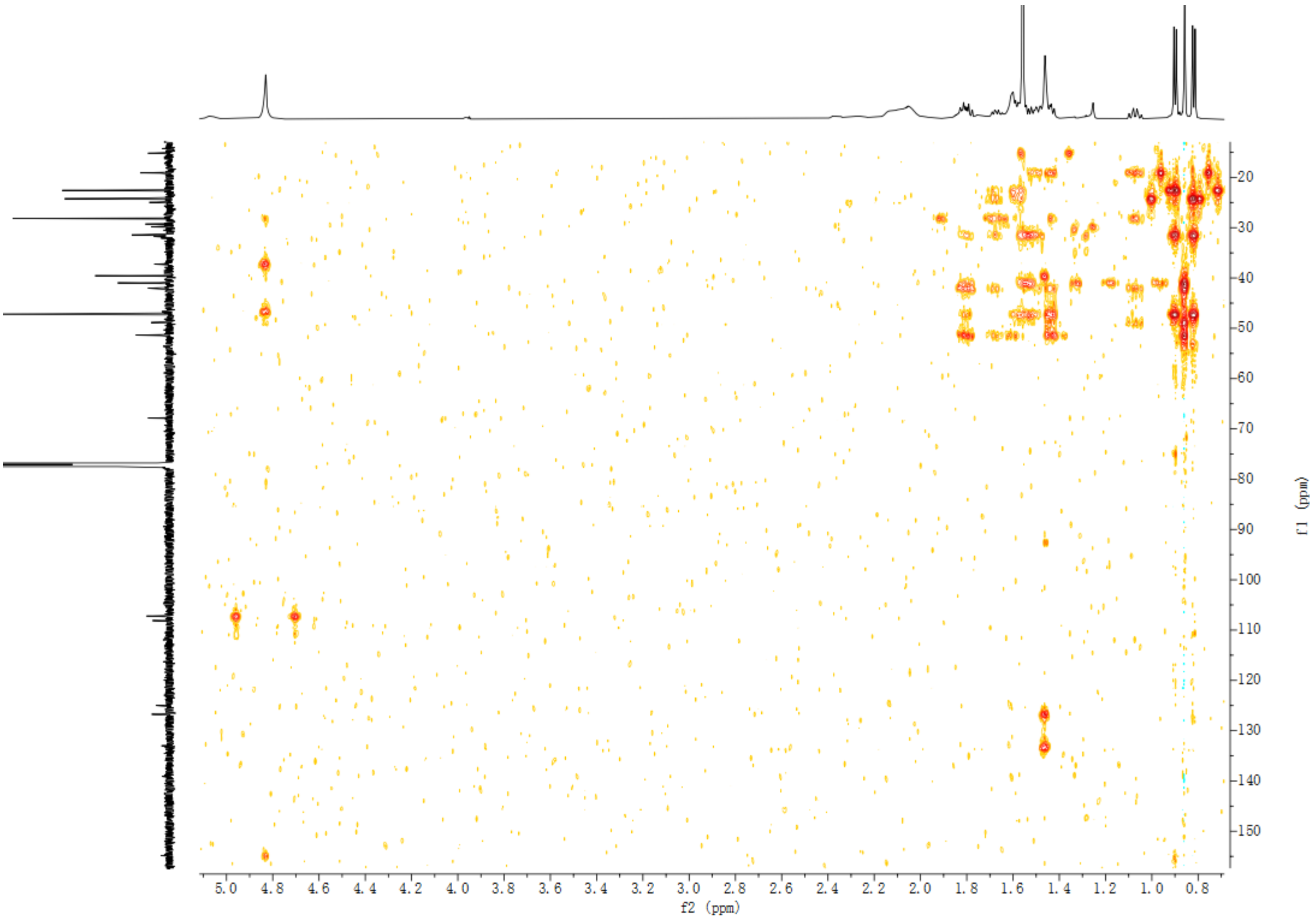

Figure S95. HMBC spectrum of compound **13** in $CDCl_3$

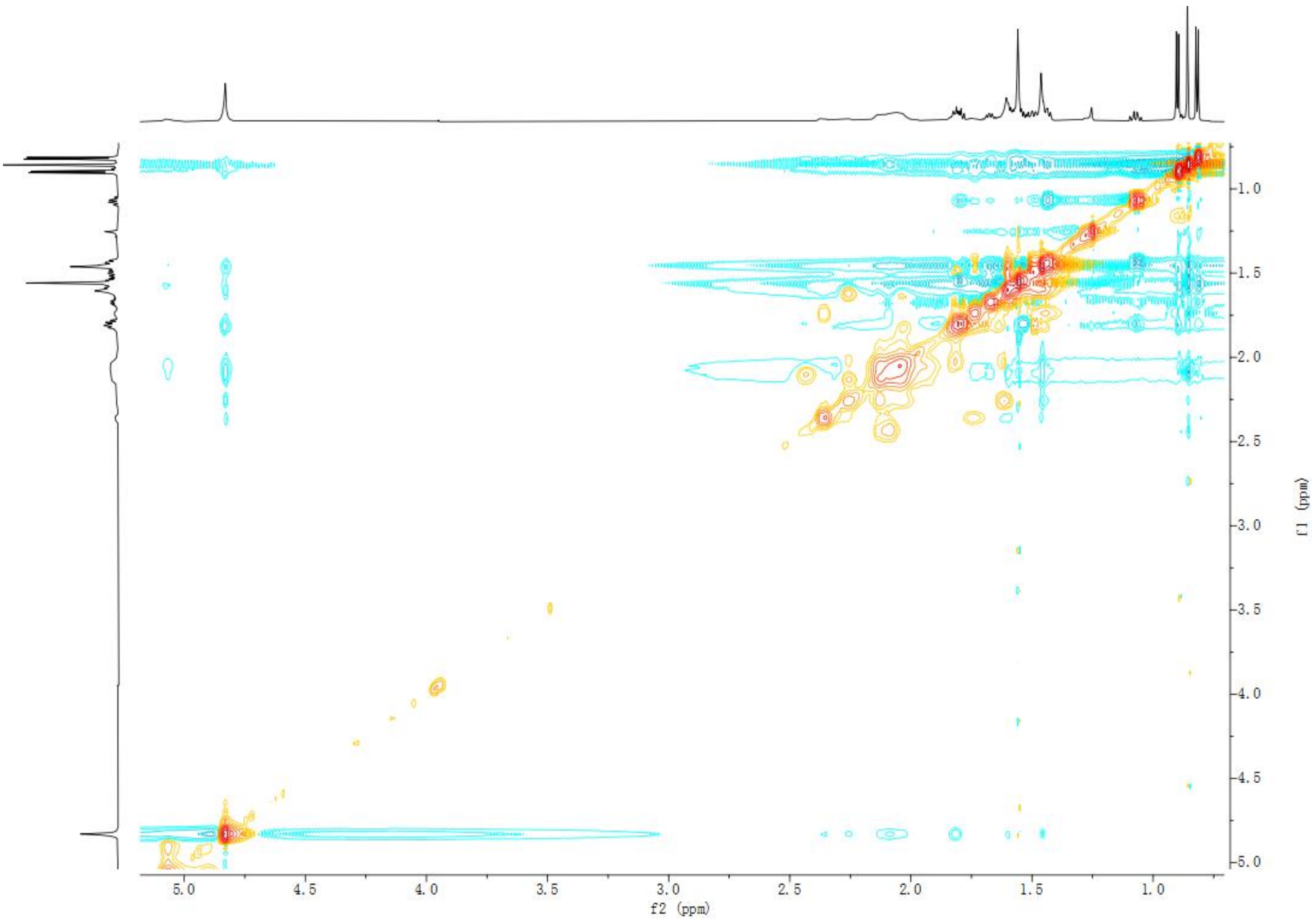

Figure S96. NOESY spectrum of compound **13** in $CDCl_3$

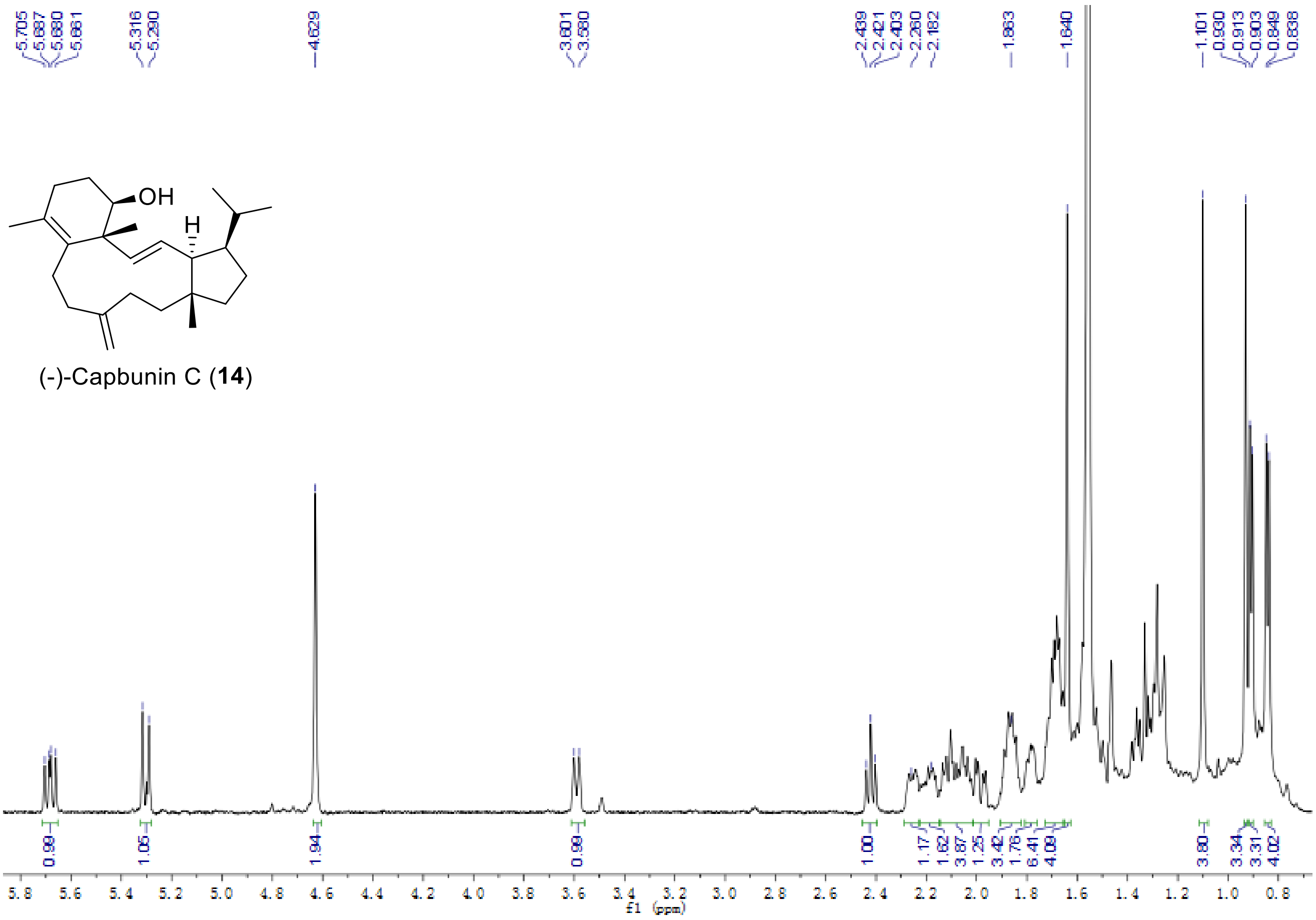

Figure S97. ^{1}H NMR spectrum of compound **14** in $CDCl_3$ (700 MHz)

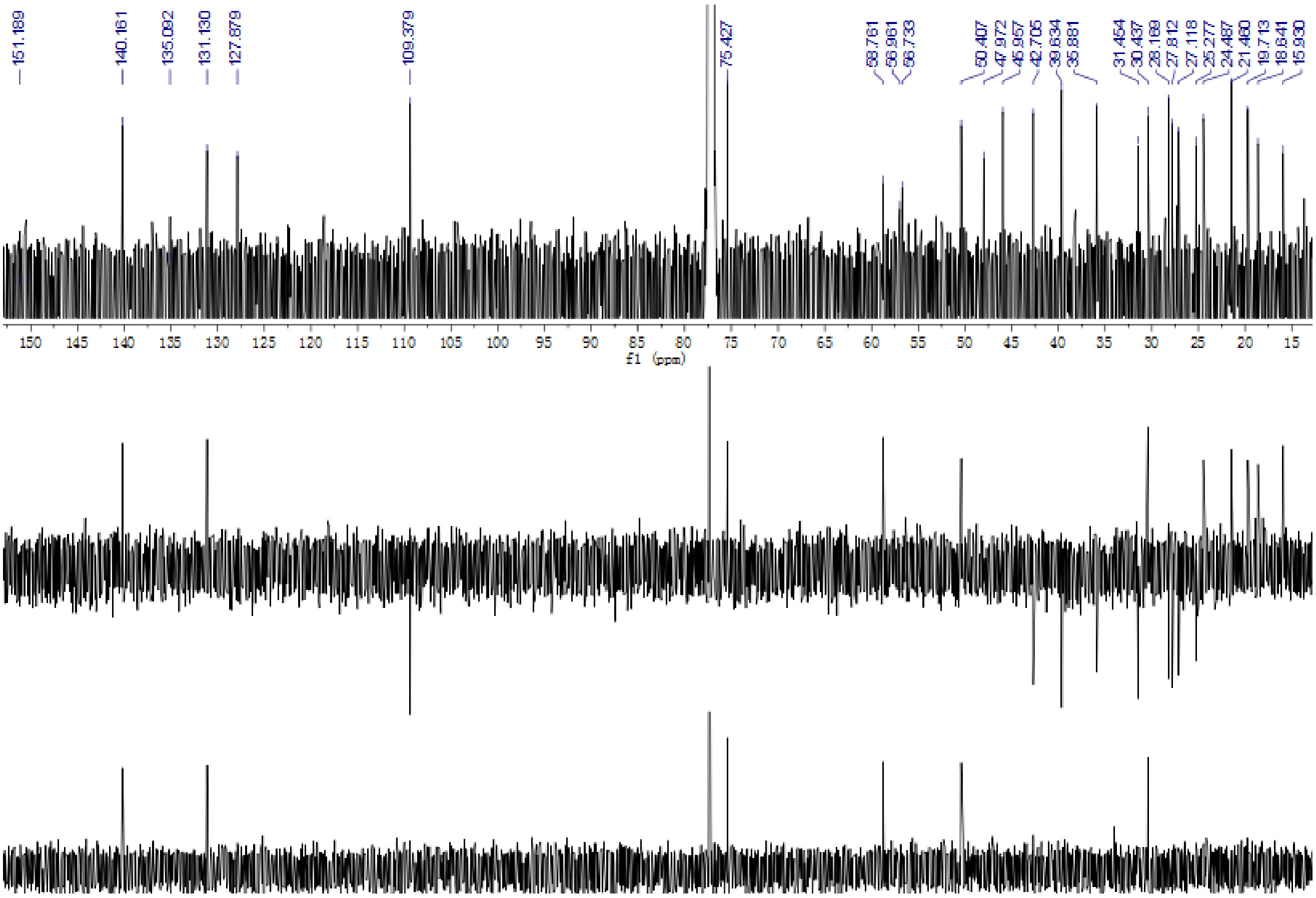

Figure S98. ^{13}C NMR and DEPT spectra of compound **14** in $CDCl_3$ (150 MHz)

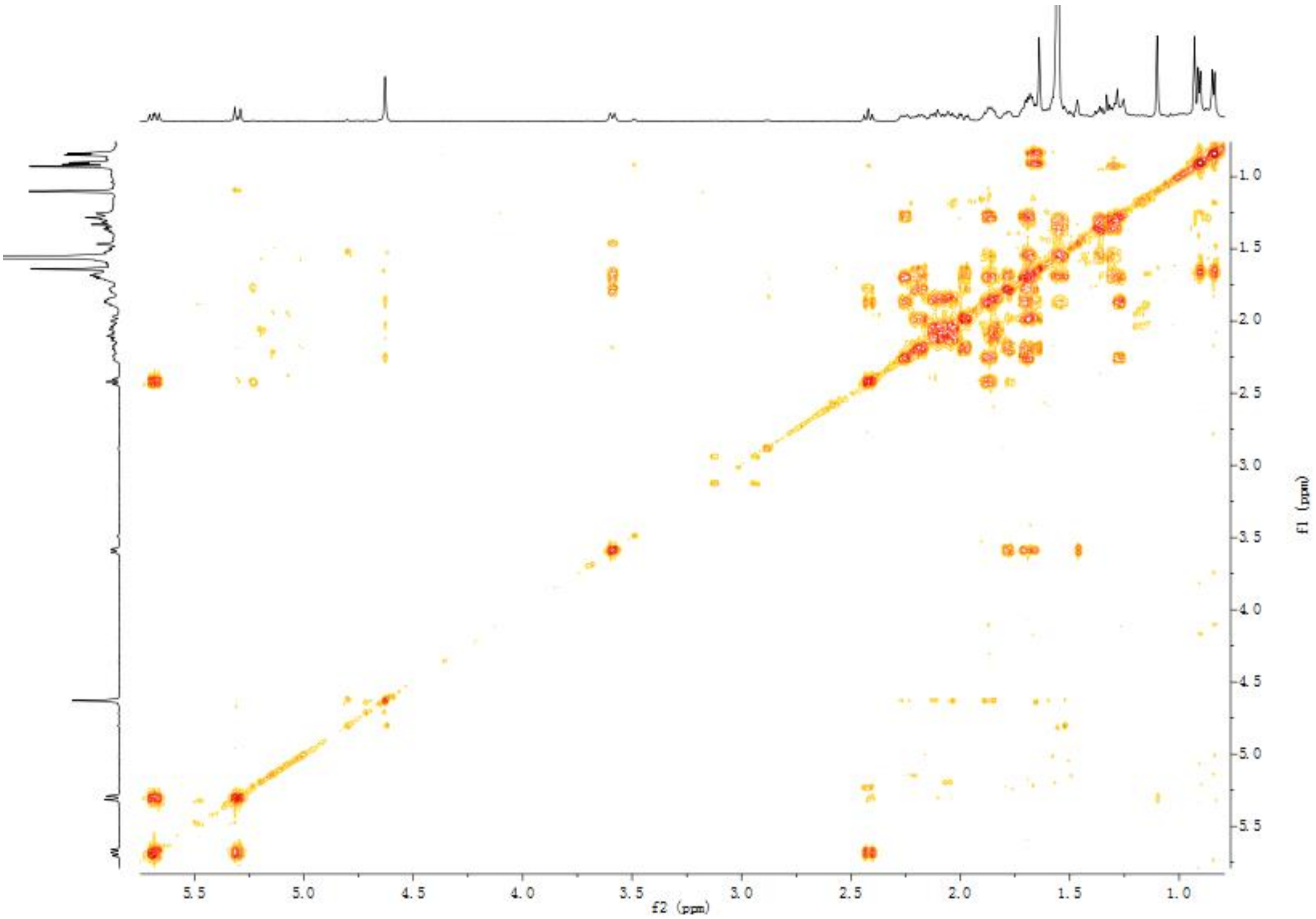

Figure S99. ^{1}H-^{1}H COSY spectrum of compound **14** in $CDCl_3$

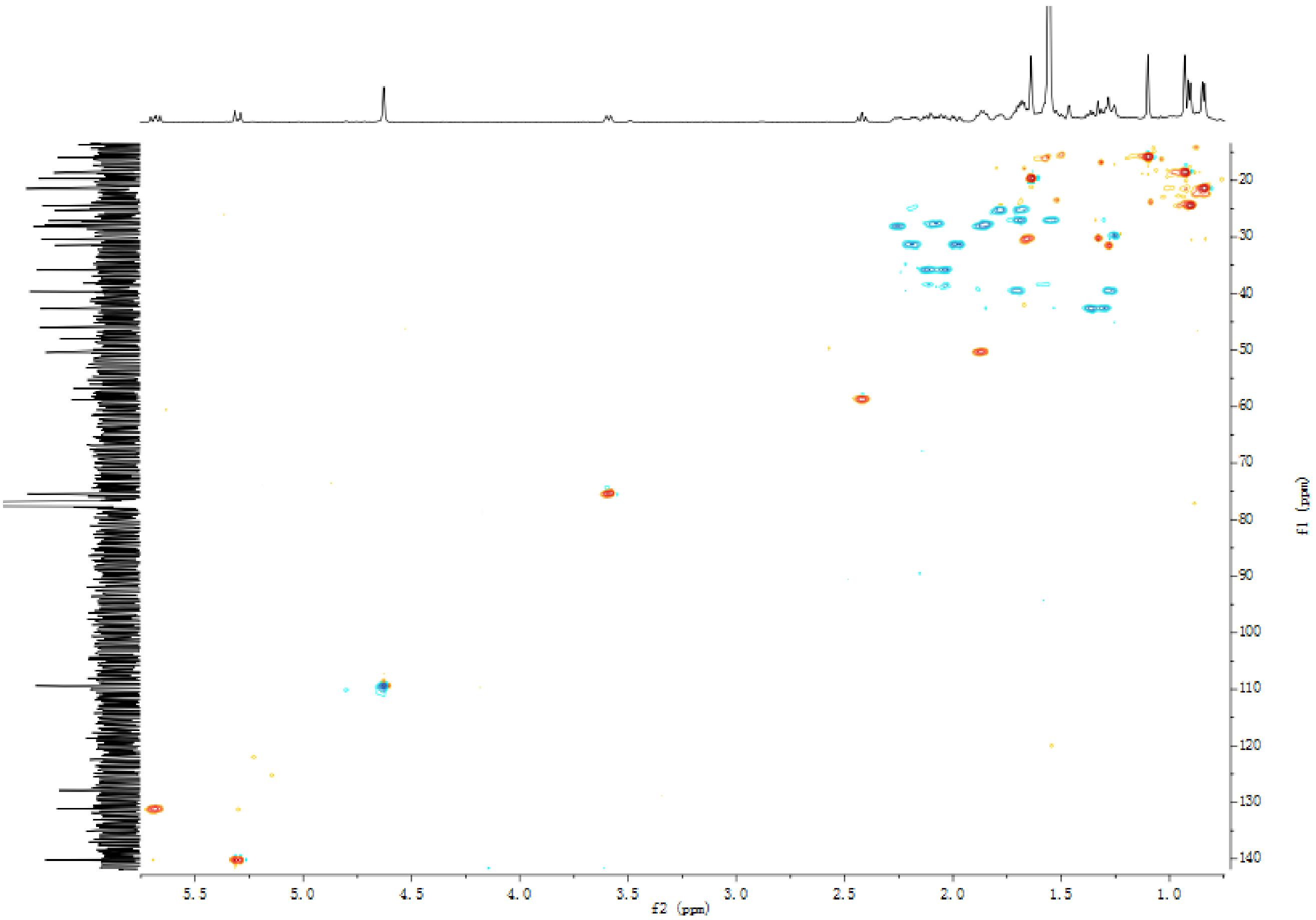

Figure S100. HSQC spectrum of compound **14** in $CDCl_3$

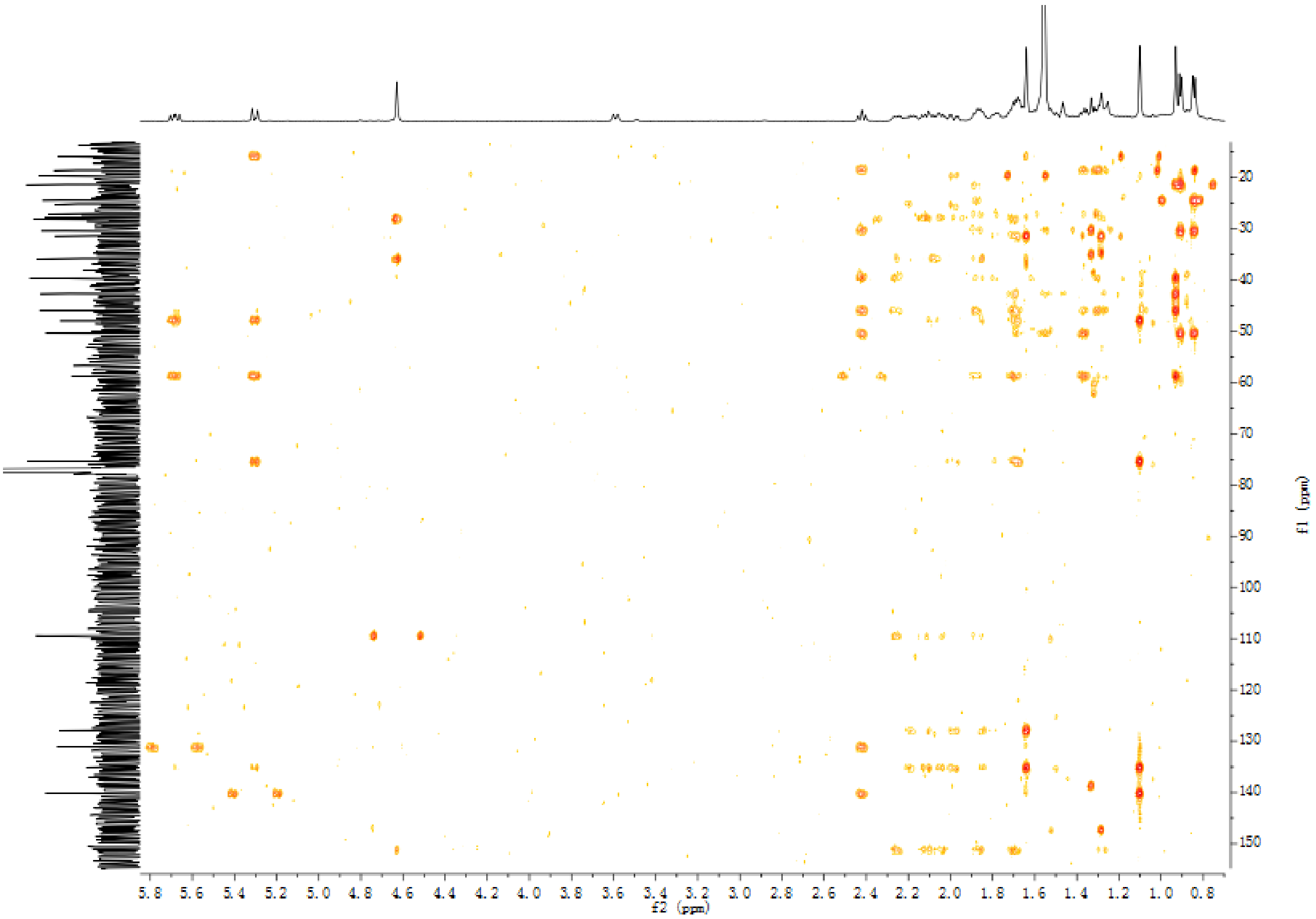

Figure S101. HMBC spectrum of compound **14** in $CDCl_3$

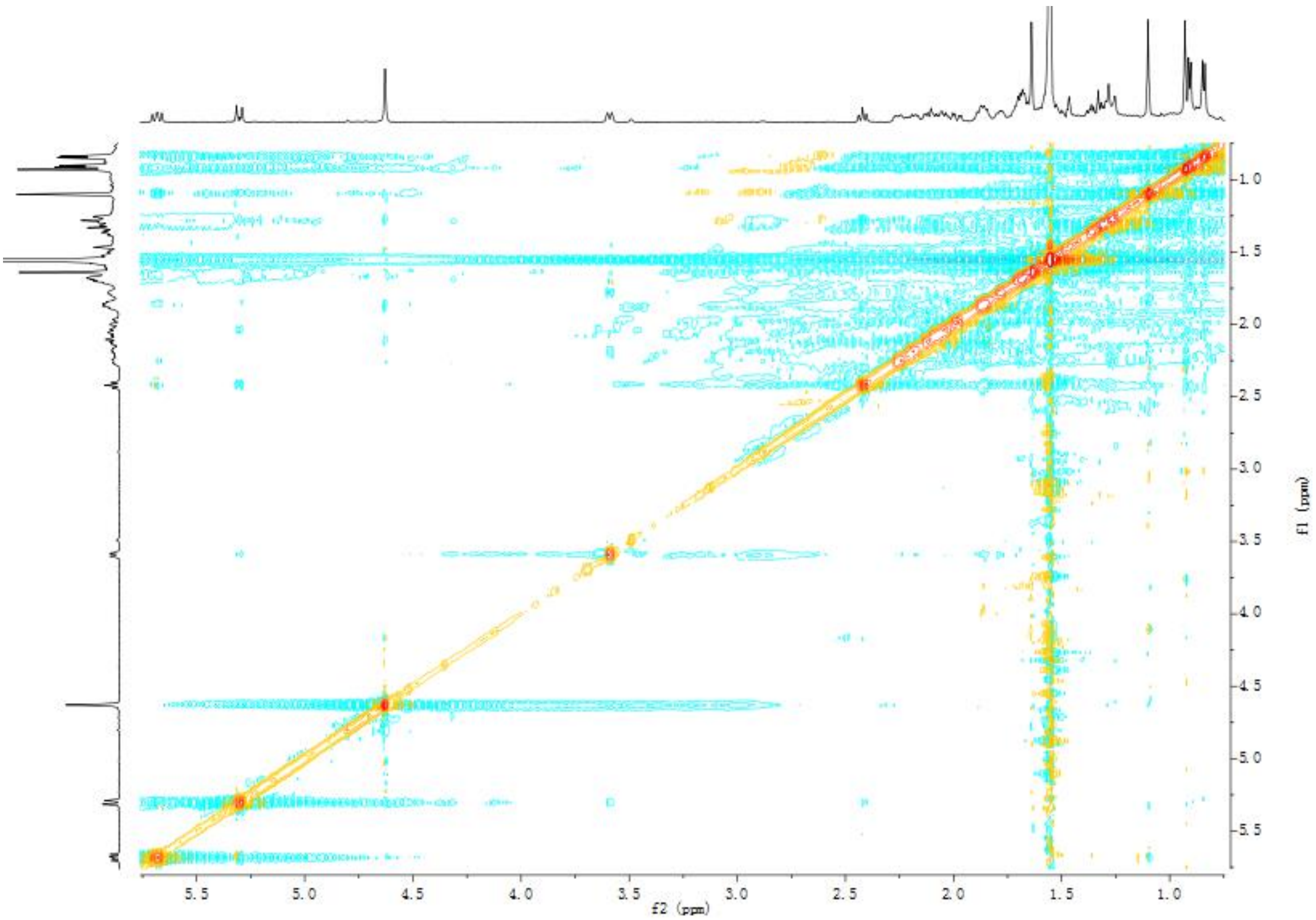

Figure S102. NOESY spectrum of compound **14** in $CDCl_3$

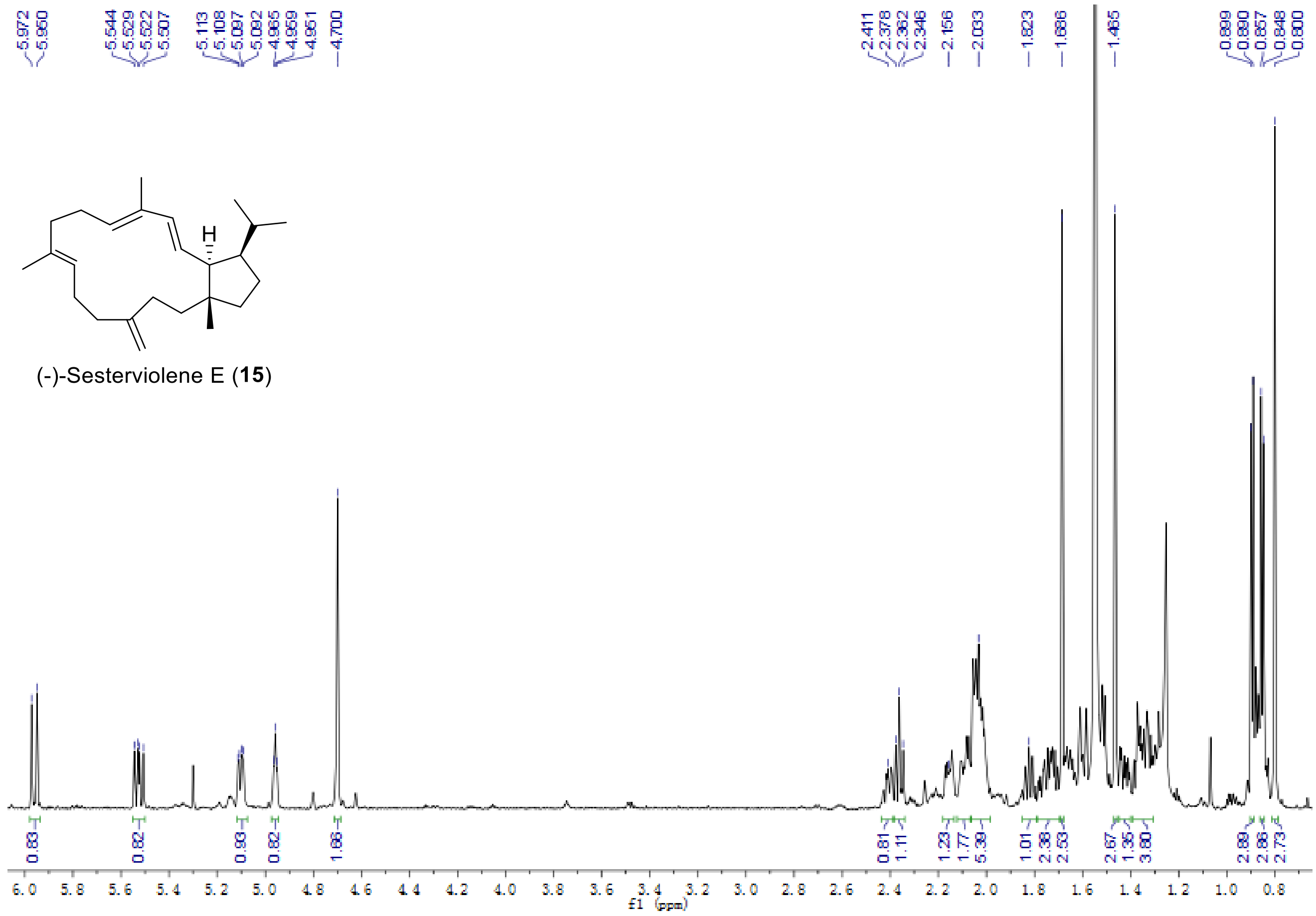

Figure S103. ^{1}H NMR spectrum of compound **15** in $CDCl_3$ (700 MHz)

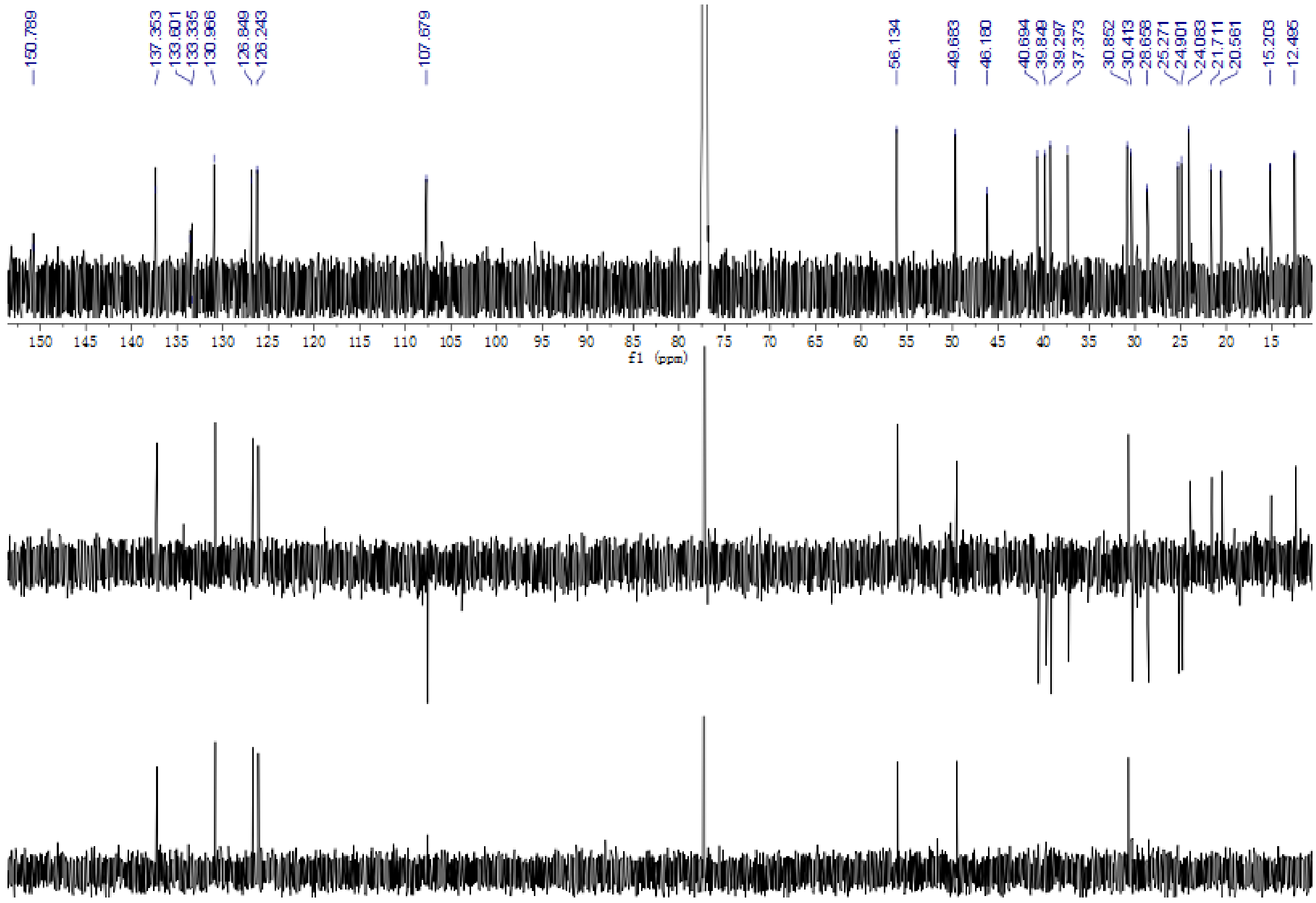

Figure S104. ^{13}C NMR and DEPT spectra of compound **15** in $CDCl_3$ (150 MHz)

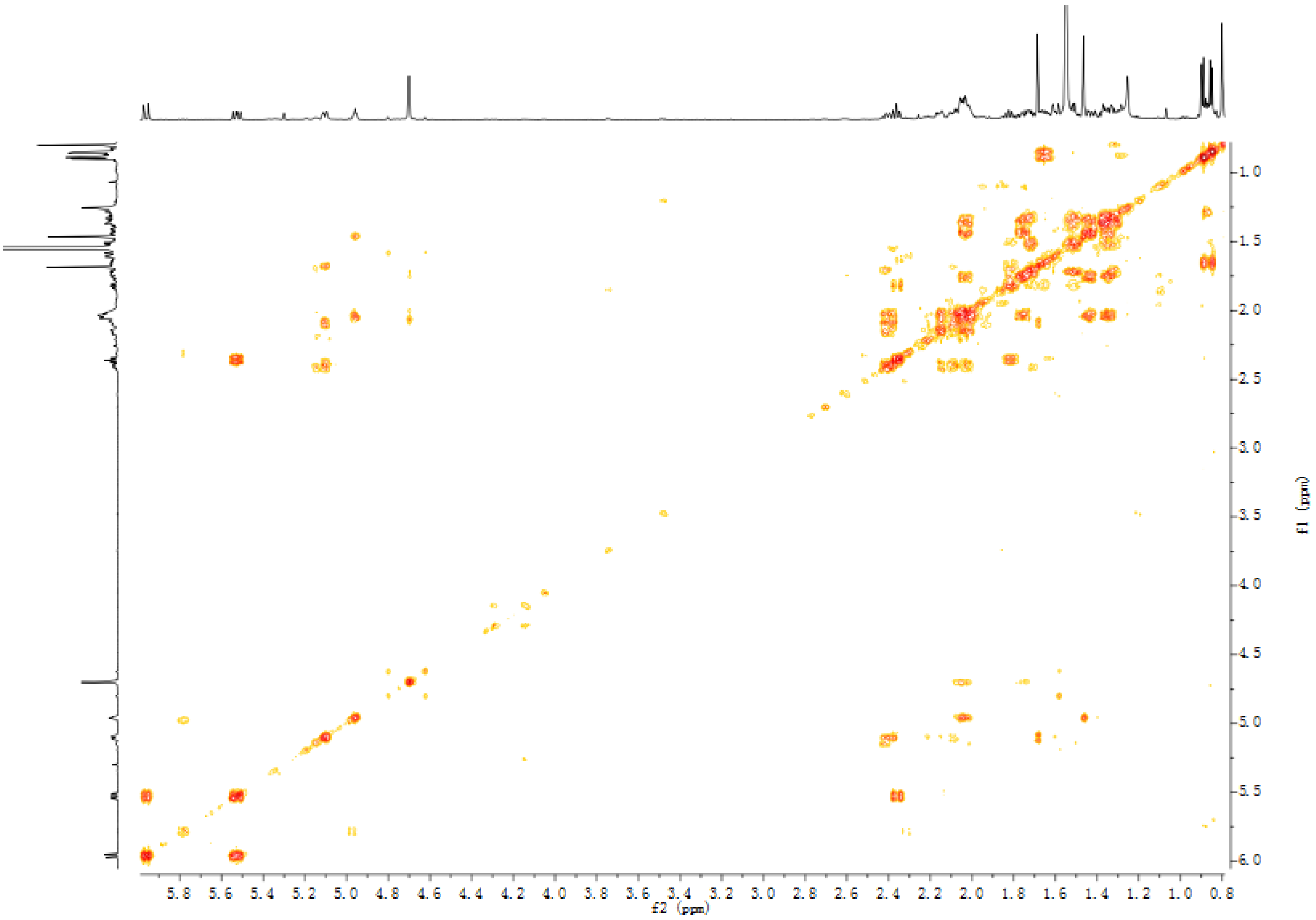

Figure S105. ^1H-^1H COSY spectrum of compound **15** in $CDCl_3$

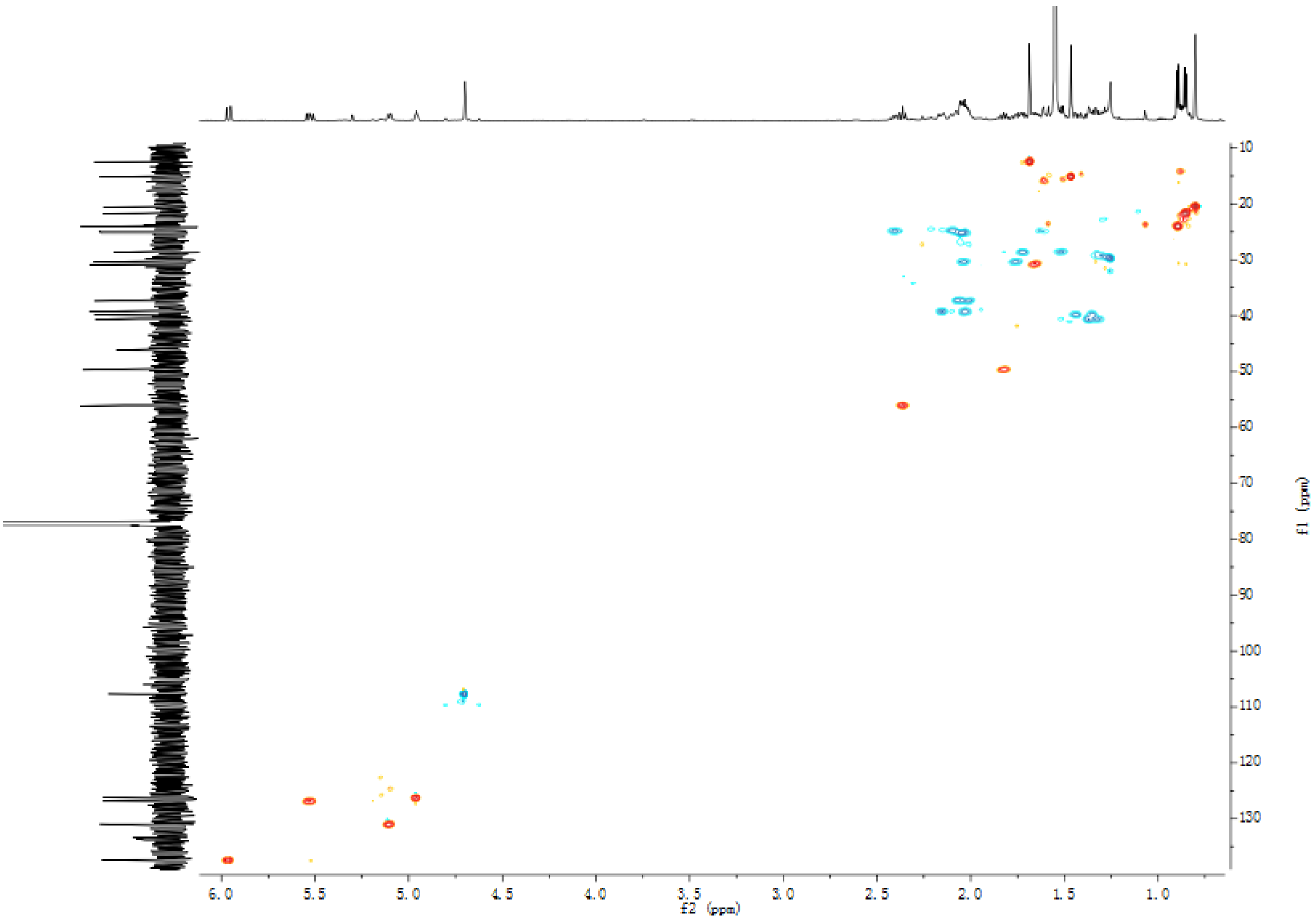

Figure S106. HSQC spectrum of compound **15** in $CDCl_3$

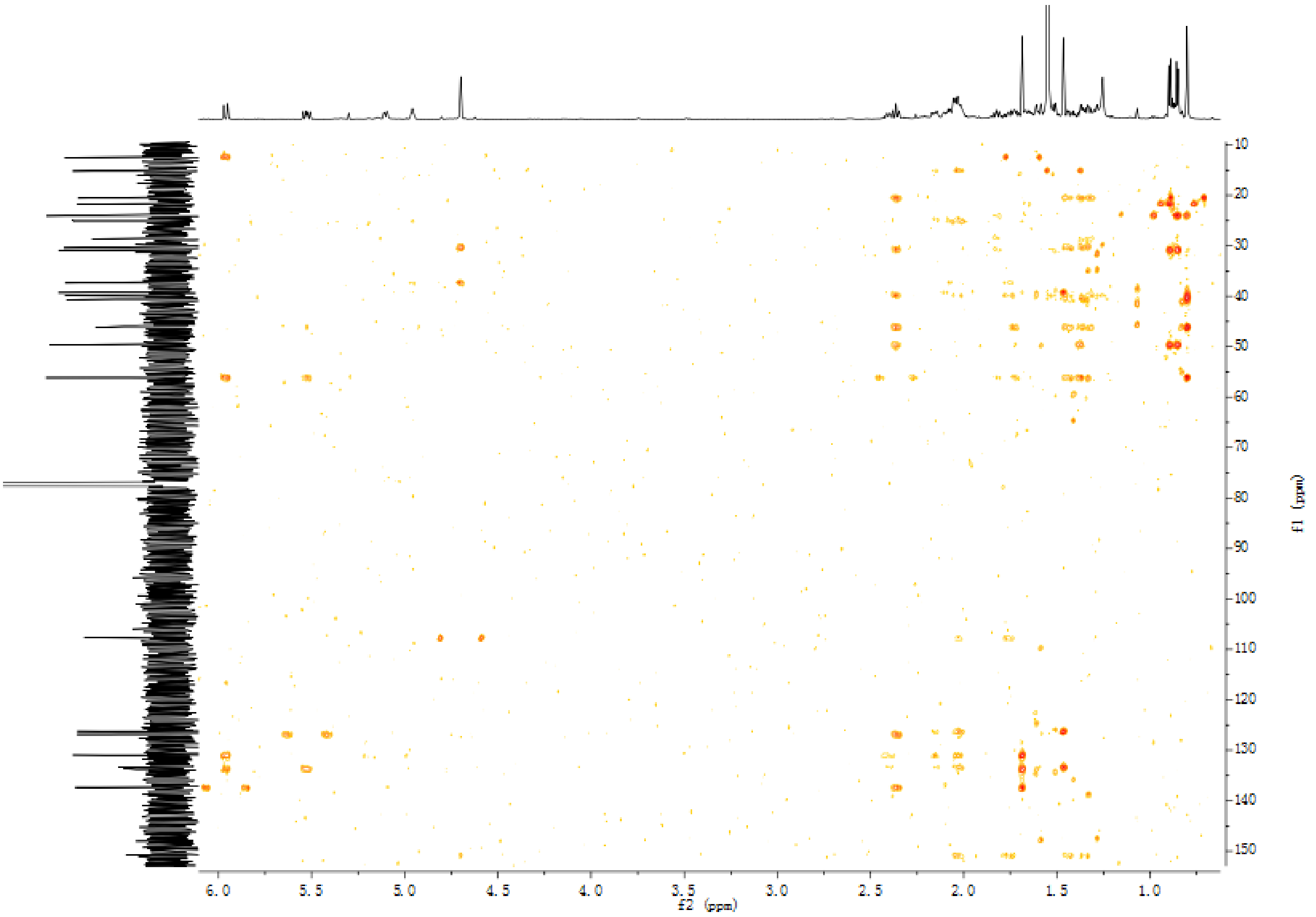

Figure S107. HMBC spectrum of compound **15** in $CDCl_3$

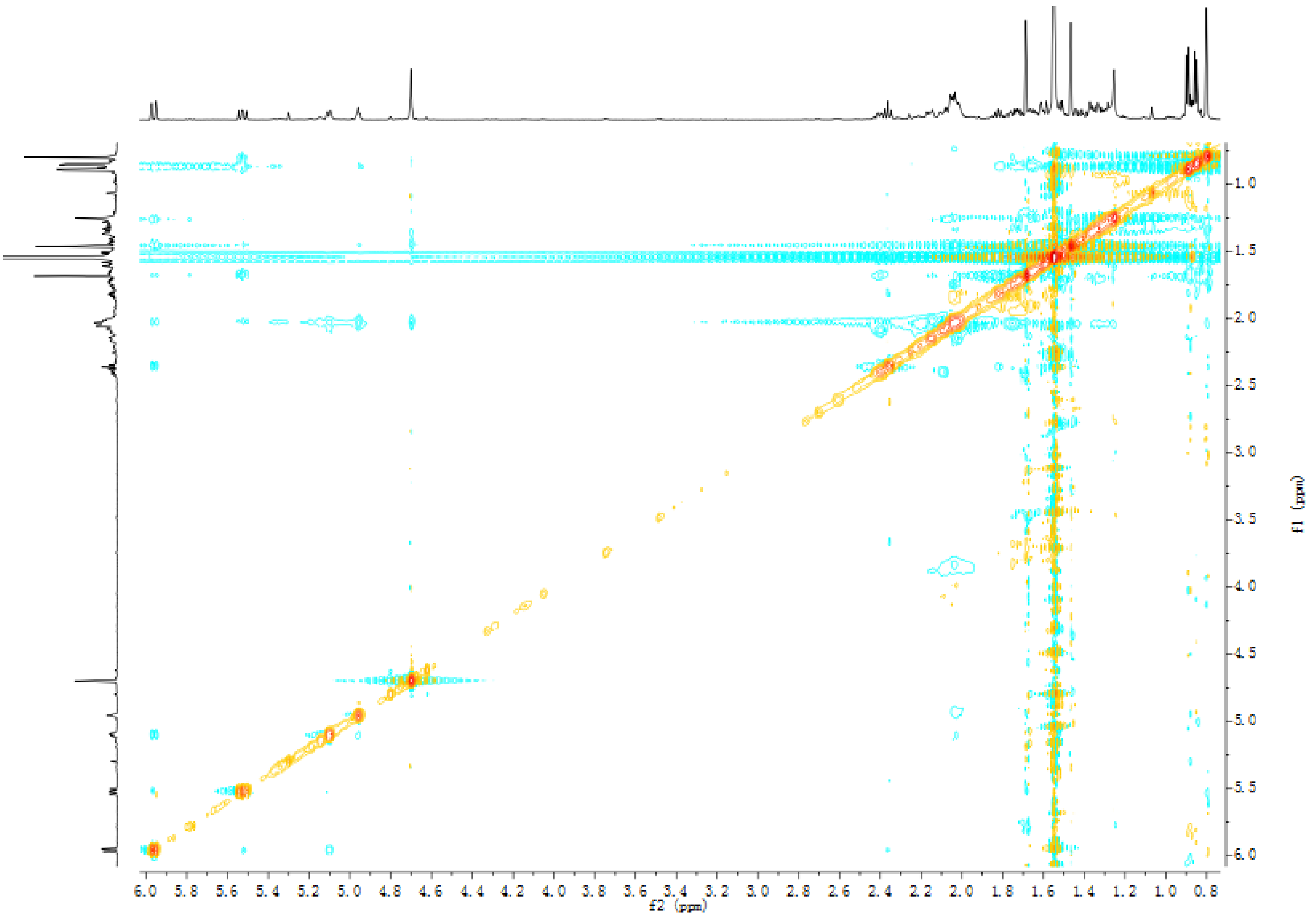

Figure S108. NOESY spectrum of compound **15** in $CDCl_3$

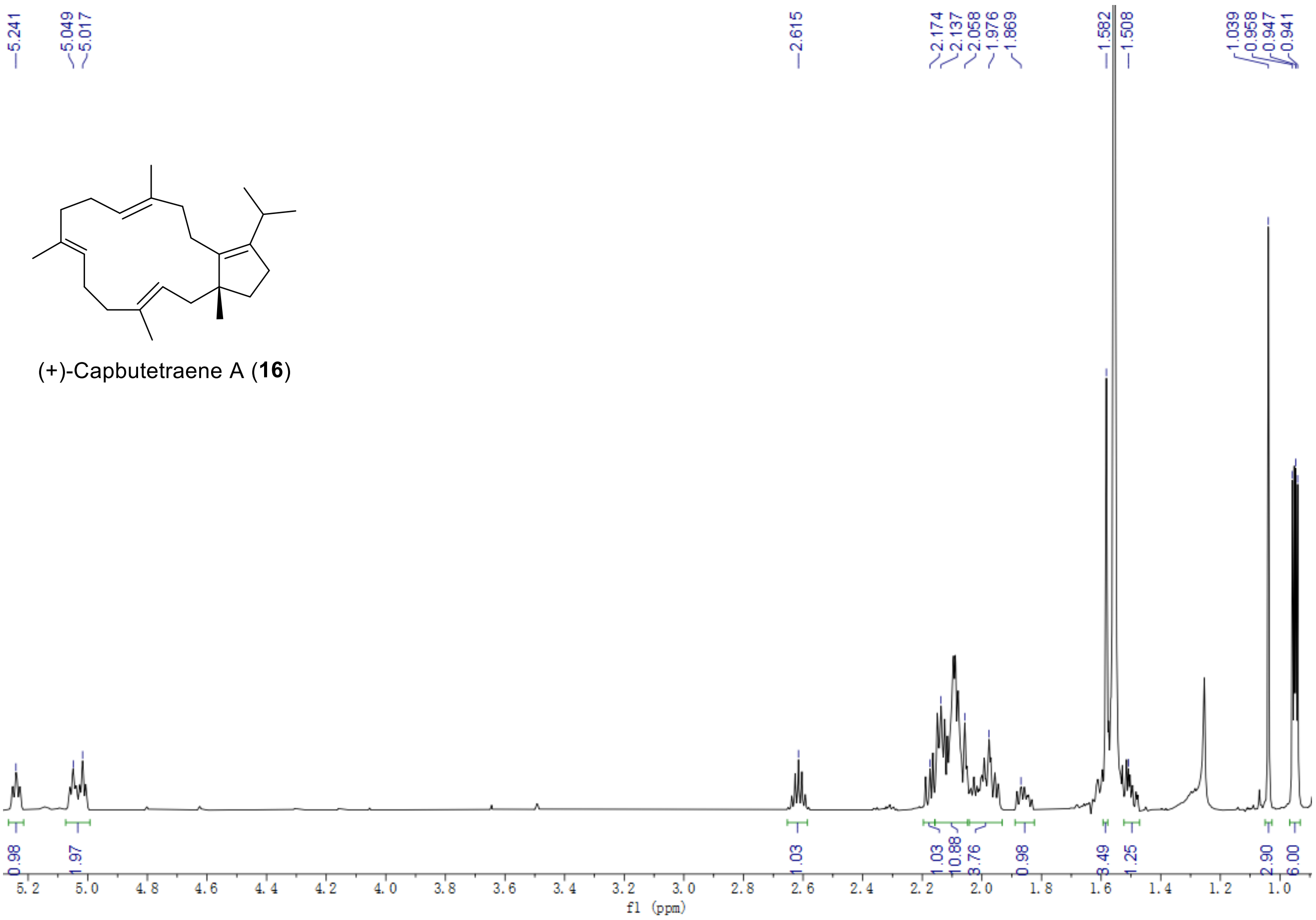

Figure S109. ^{1}H NMR spectrum of compound **16** in $CDCl_3$ (700 MHz)

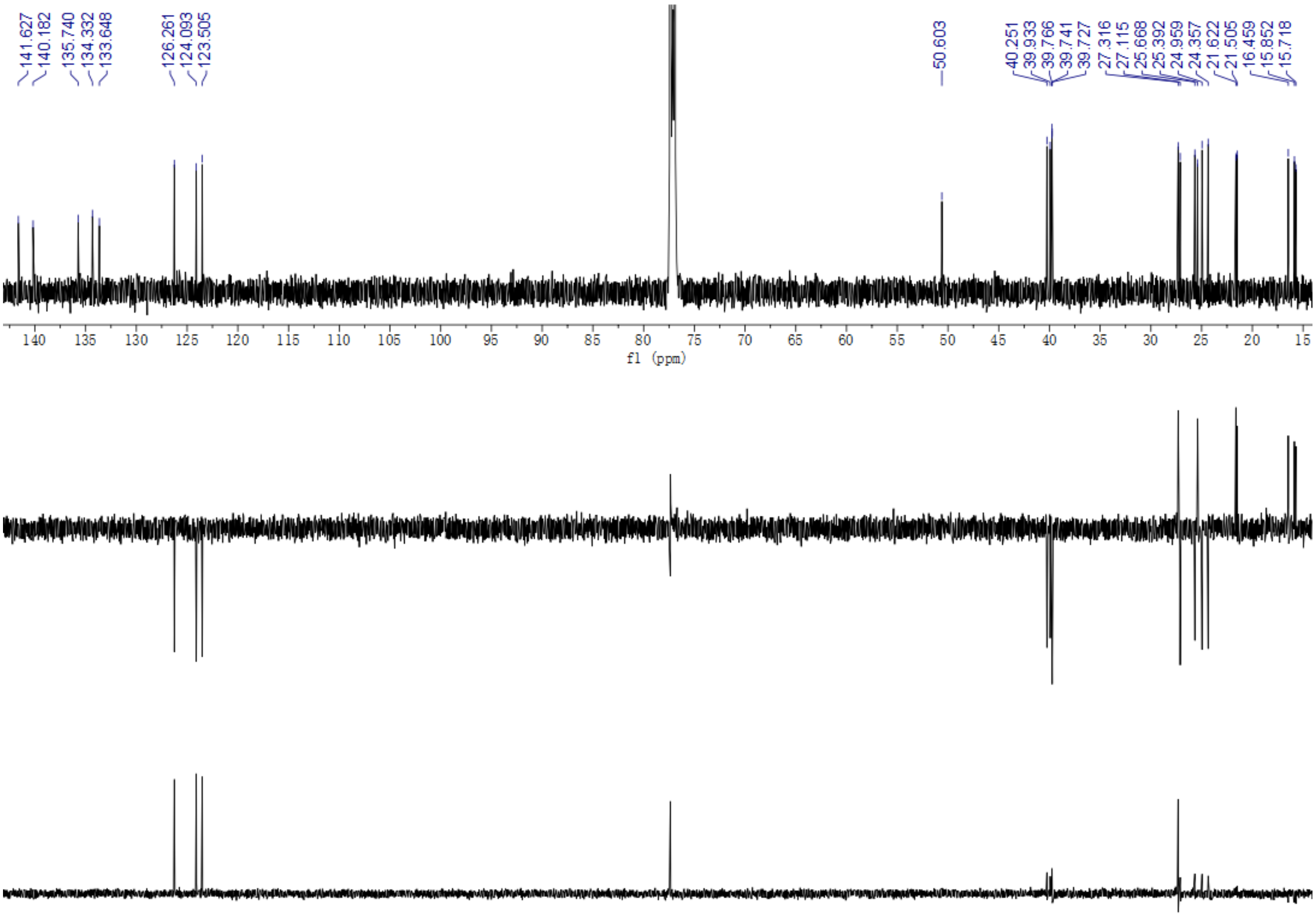

Figure S110. ^{13}C NMR and DEPT spectra of compound **16** in $CDCl_3$ (150 MHz)

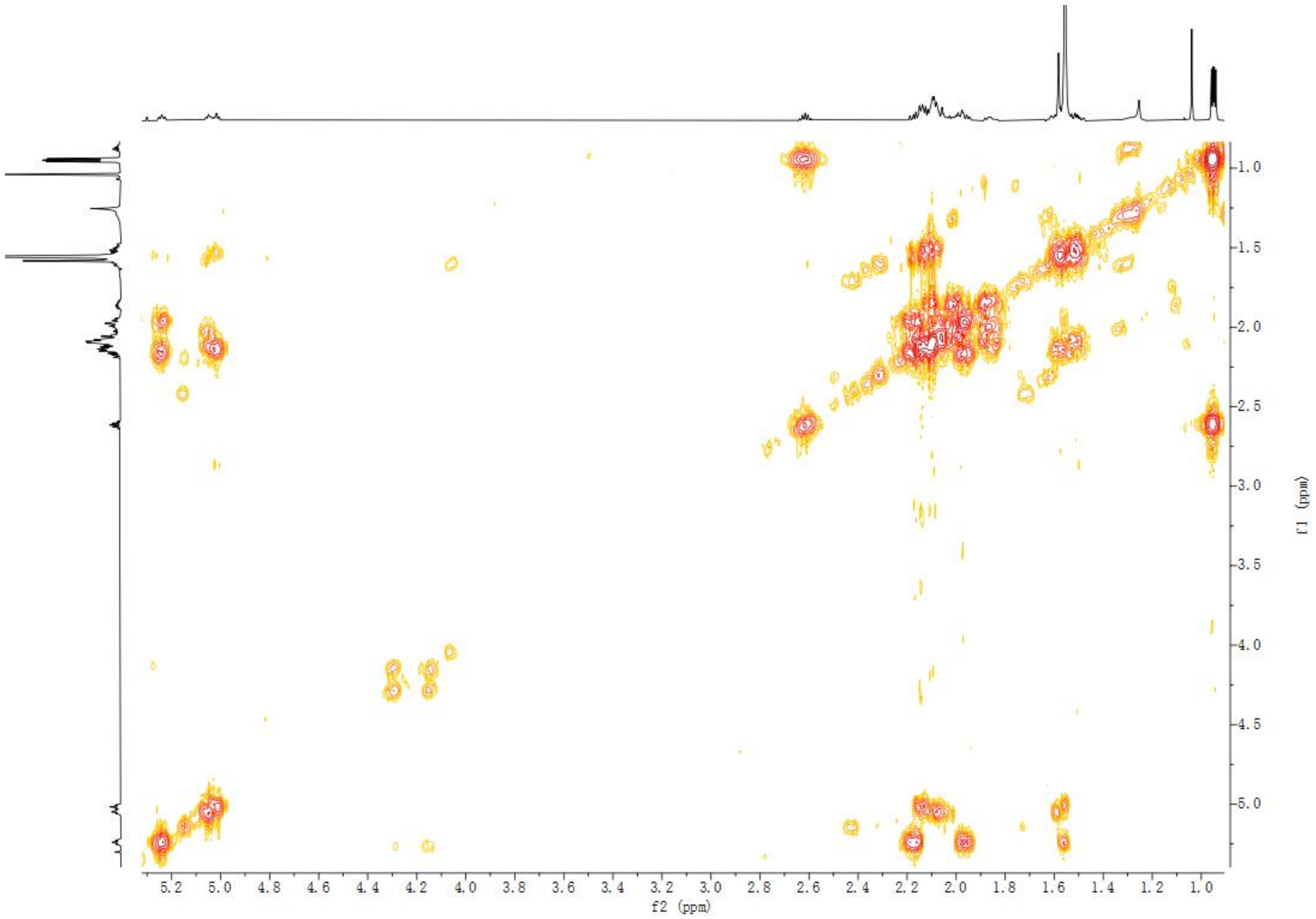

Figure S111. ^{1}H-^{1}H COSY spectrum of compound **16** in $CDCl_3$

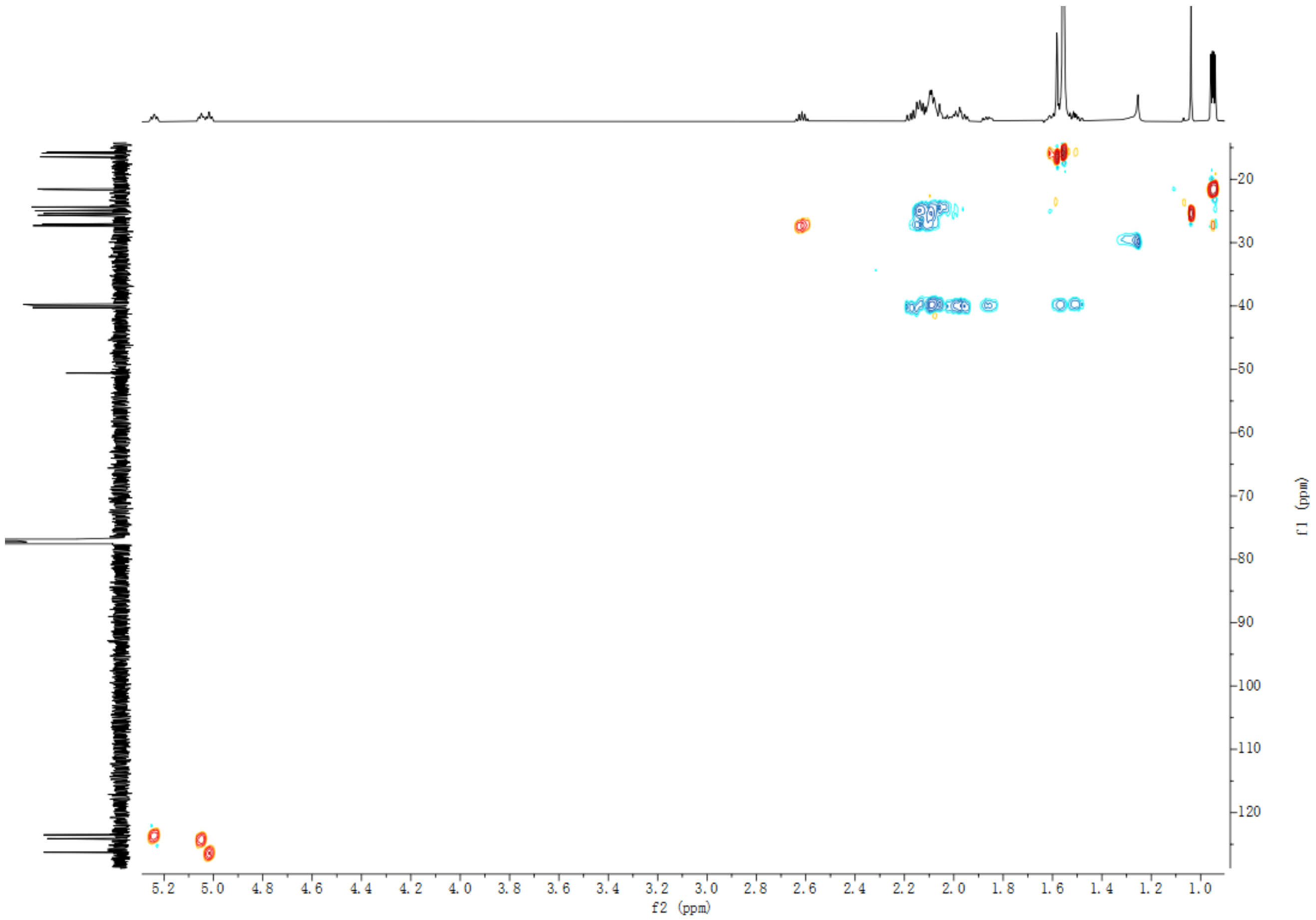

Figure S112. HSQC spectrum of compound **16** in $CDCl_3$

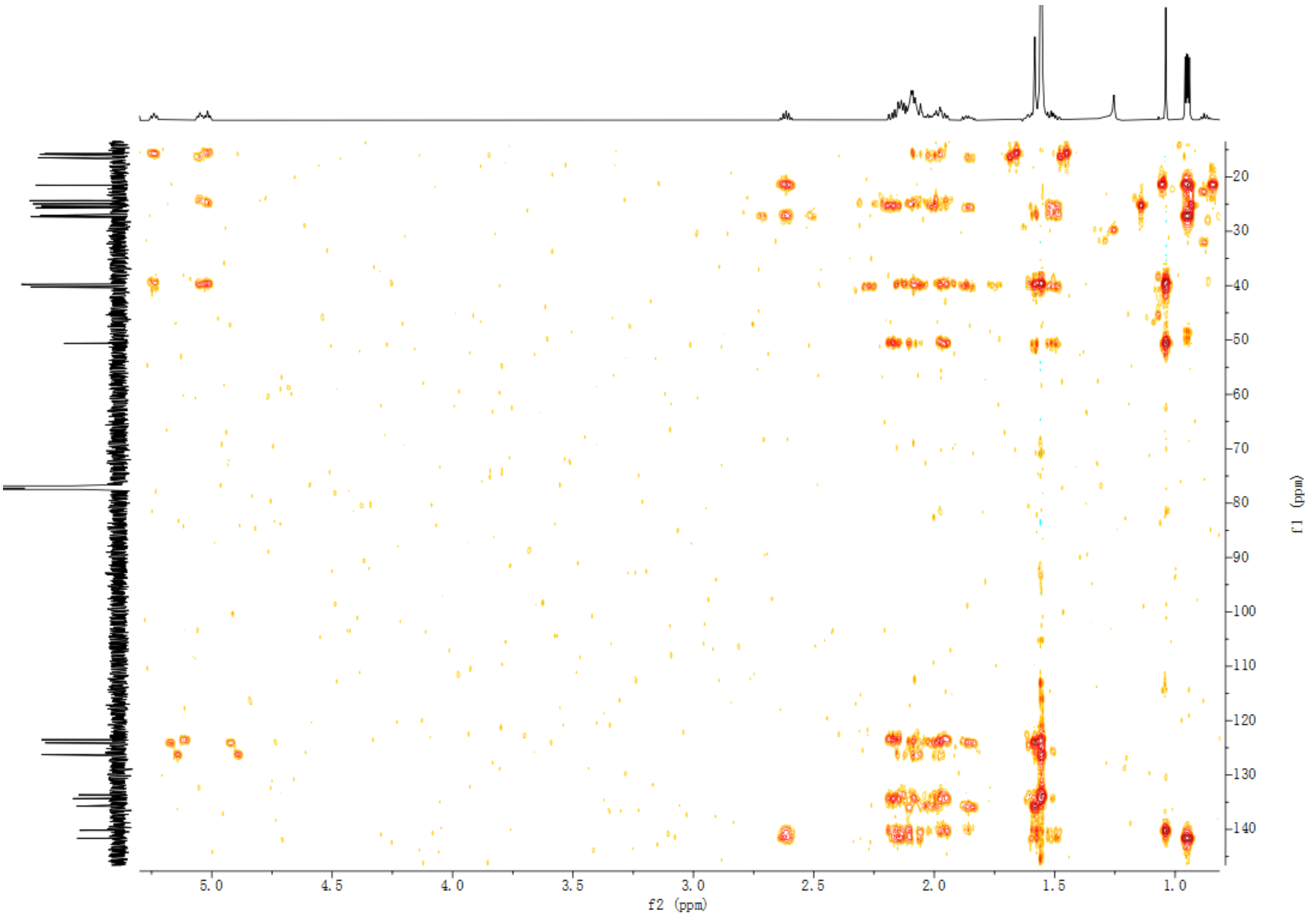

Figure S113. HMBC spectrum of compound **16** in $CDCl_3$

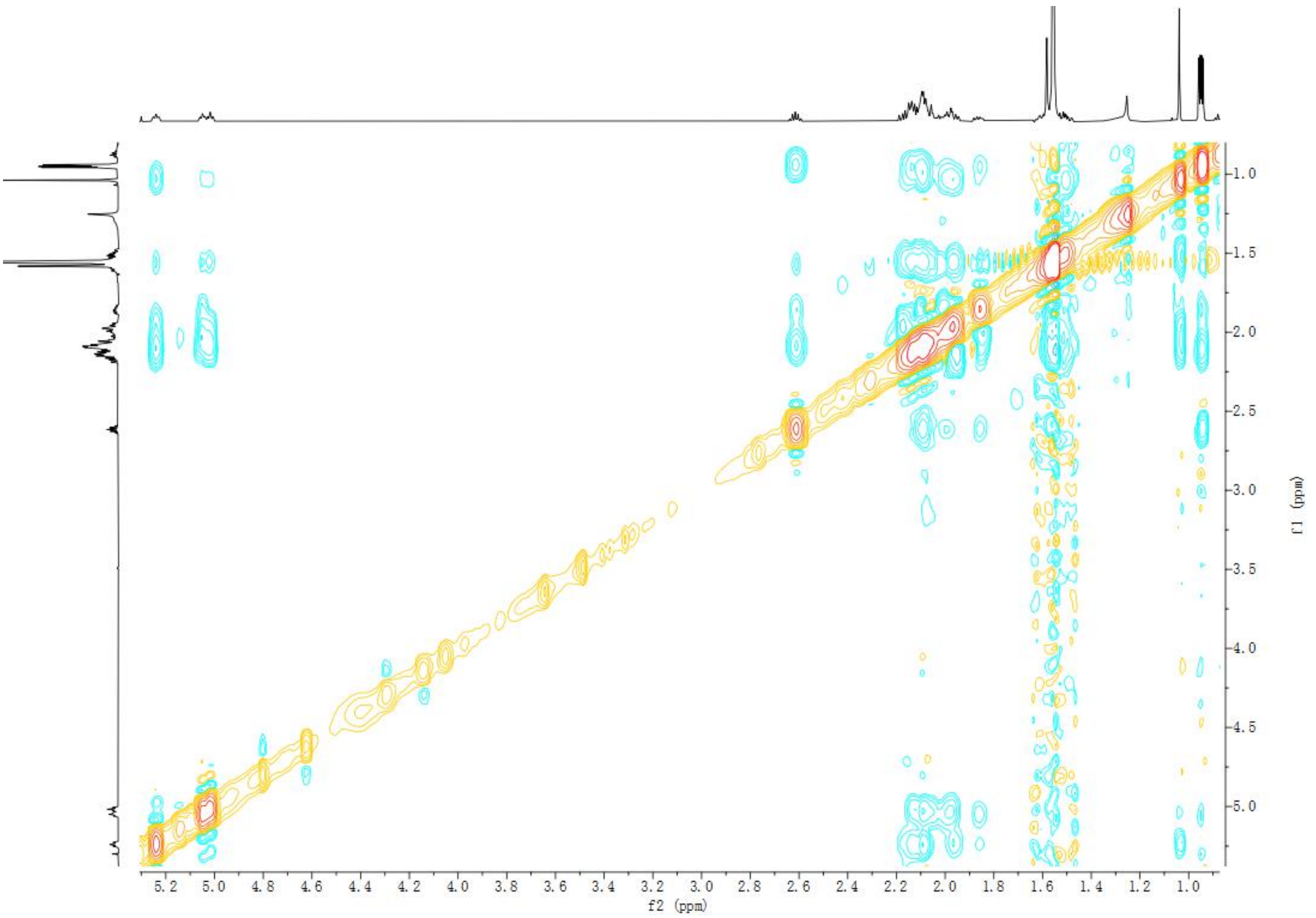

Figure S114. NOESY spectrum of compound **16** in $CDCl_3$

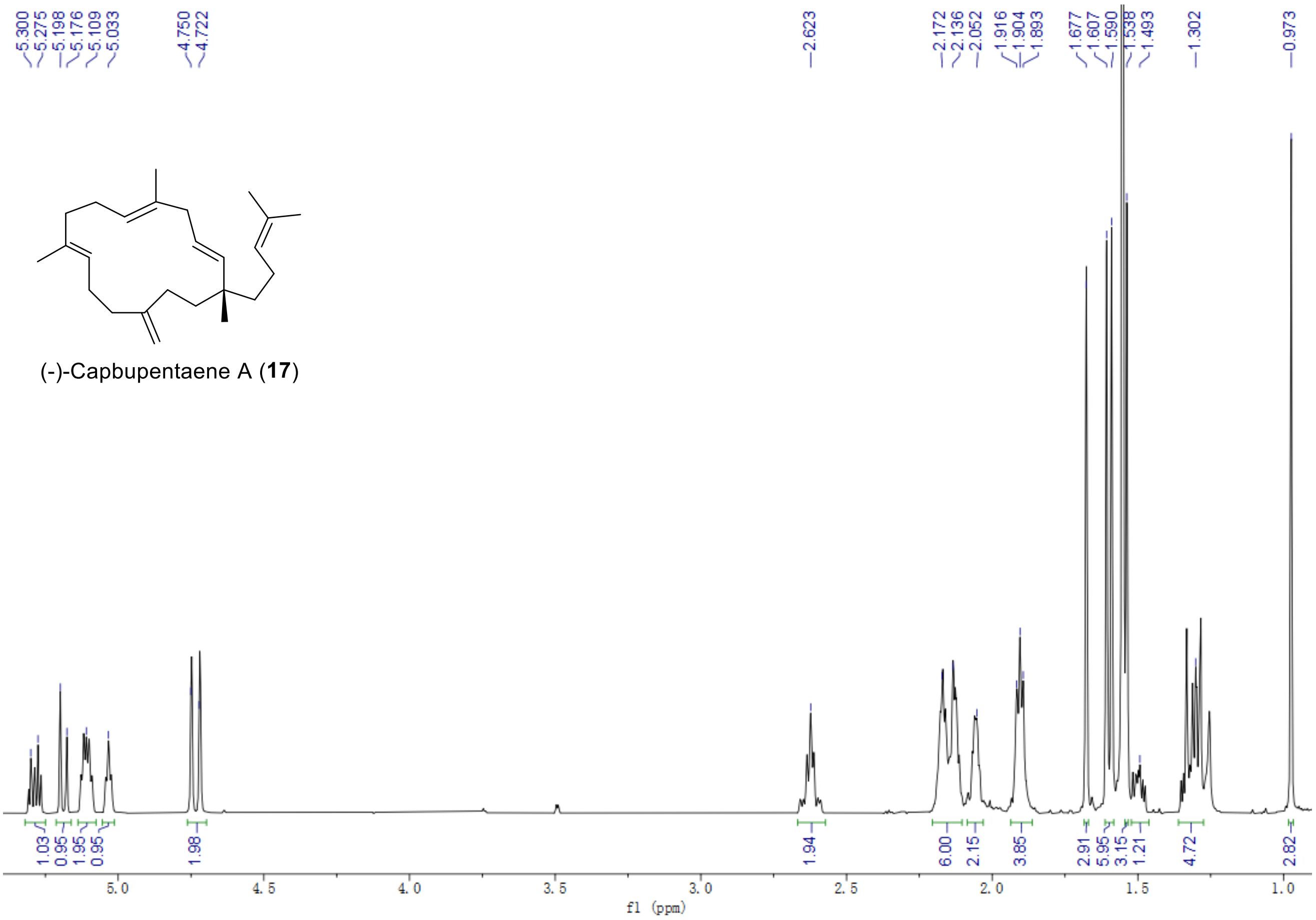

Figure S115. ^{1}H NMR spectrum of compound **17** in $CDCl_3$ (700 MHz)

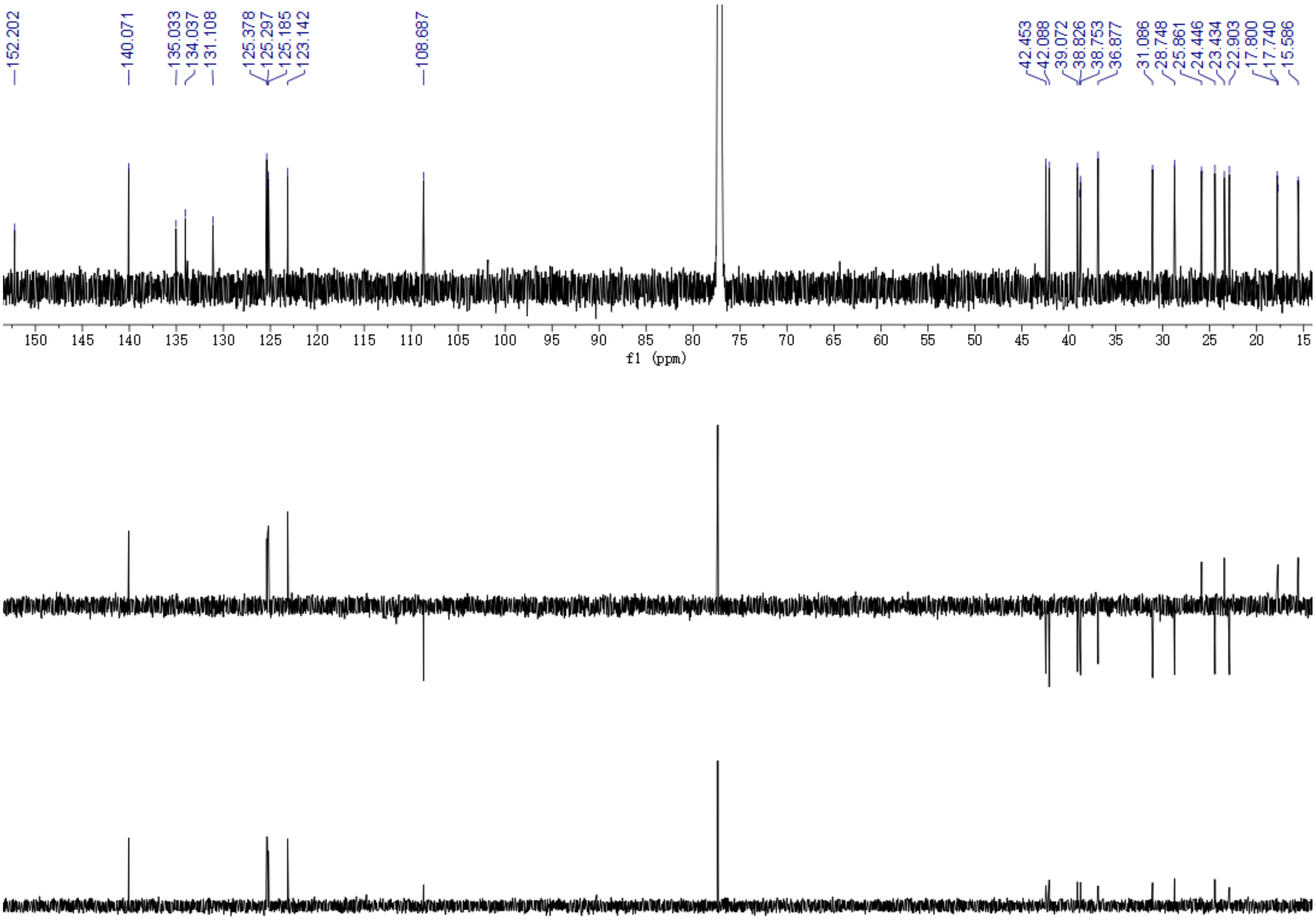

Figure S116. ^{13}C NMR and DEPT spectra of compound **17** in $CDCl_3$ (150 MHz)

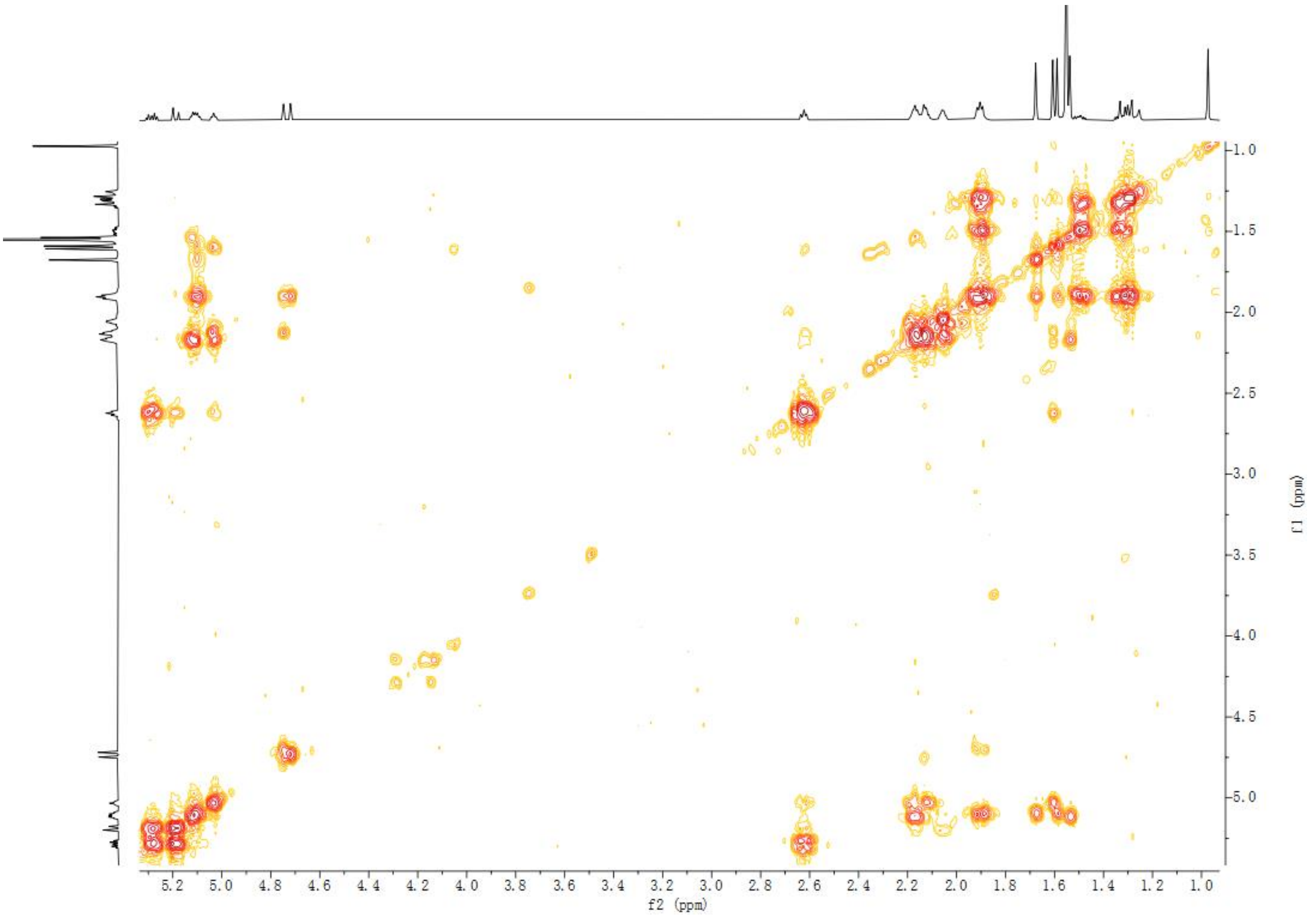

Figure S117. ^1H-^1H COSY spectrum of compound **17** in $CDCl_3$

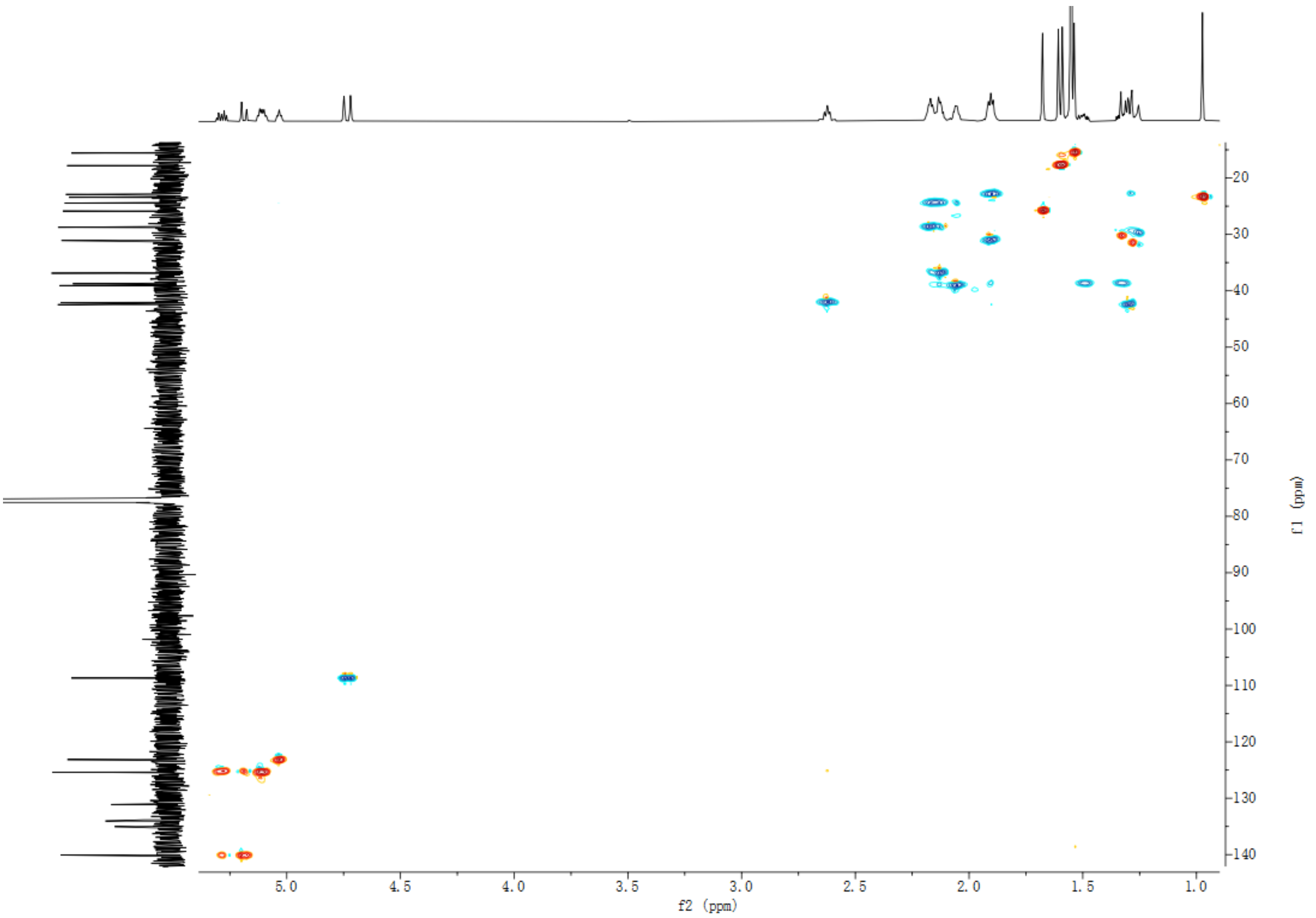

Figure S118. HSQC spectrum of compound **17** in $CDCl_3$

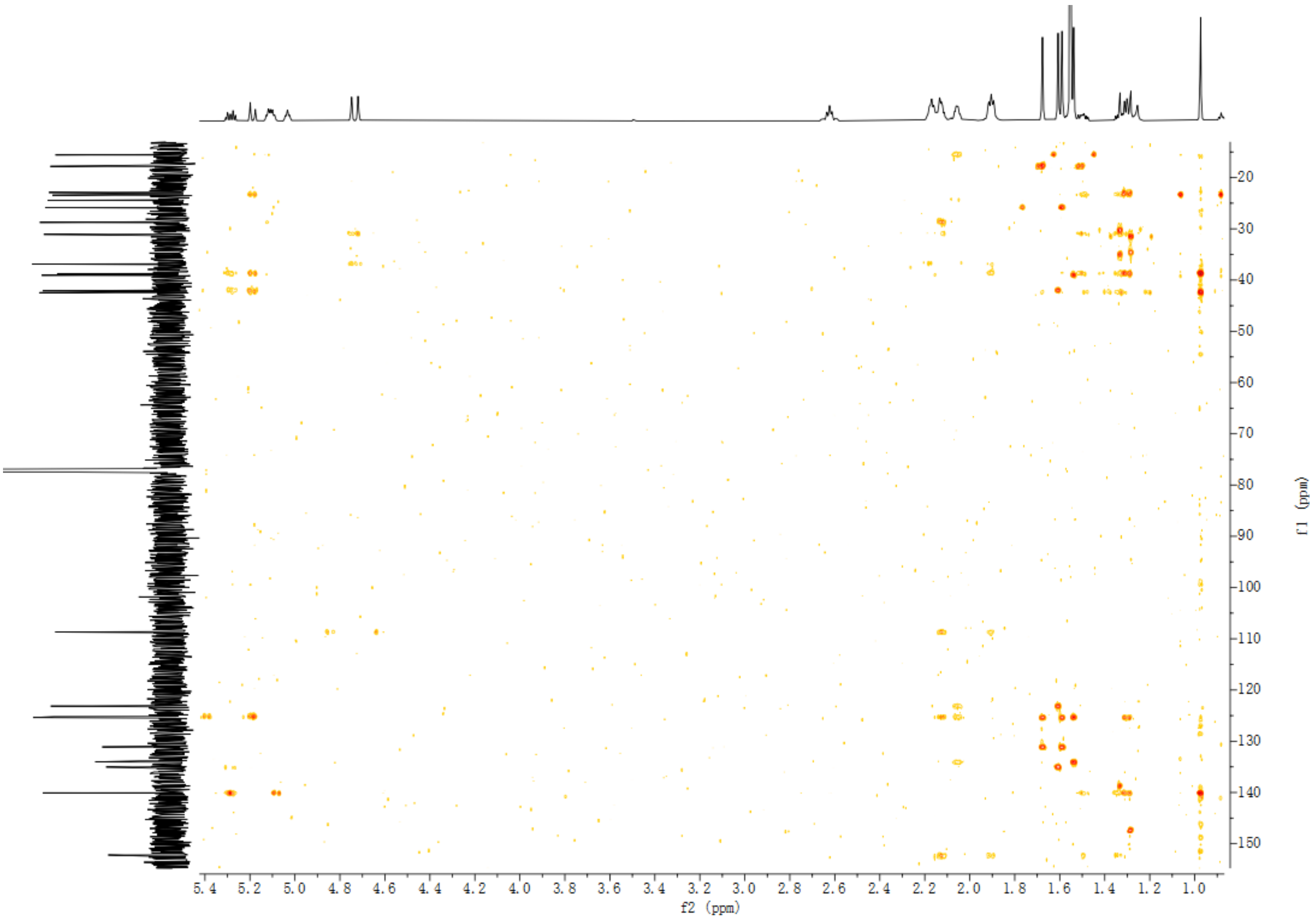

Figure S119. HMBC spectrum of compound **17** in $CDCl_3$

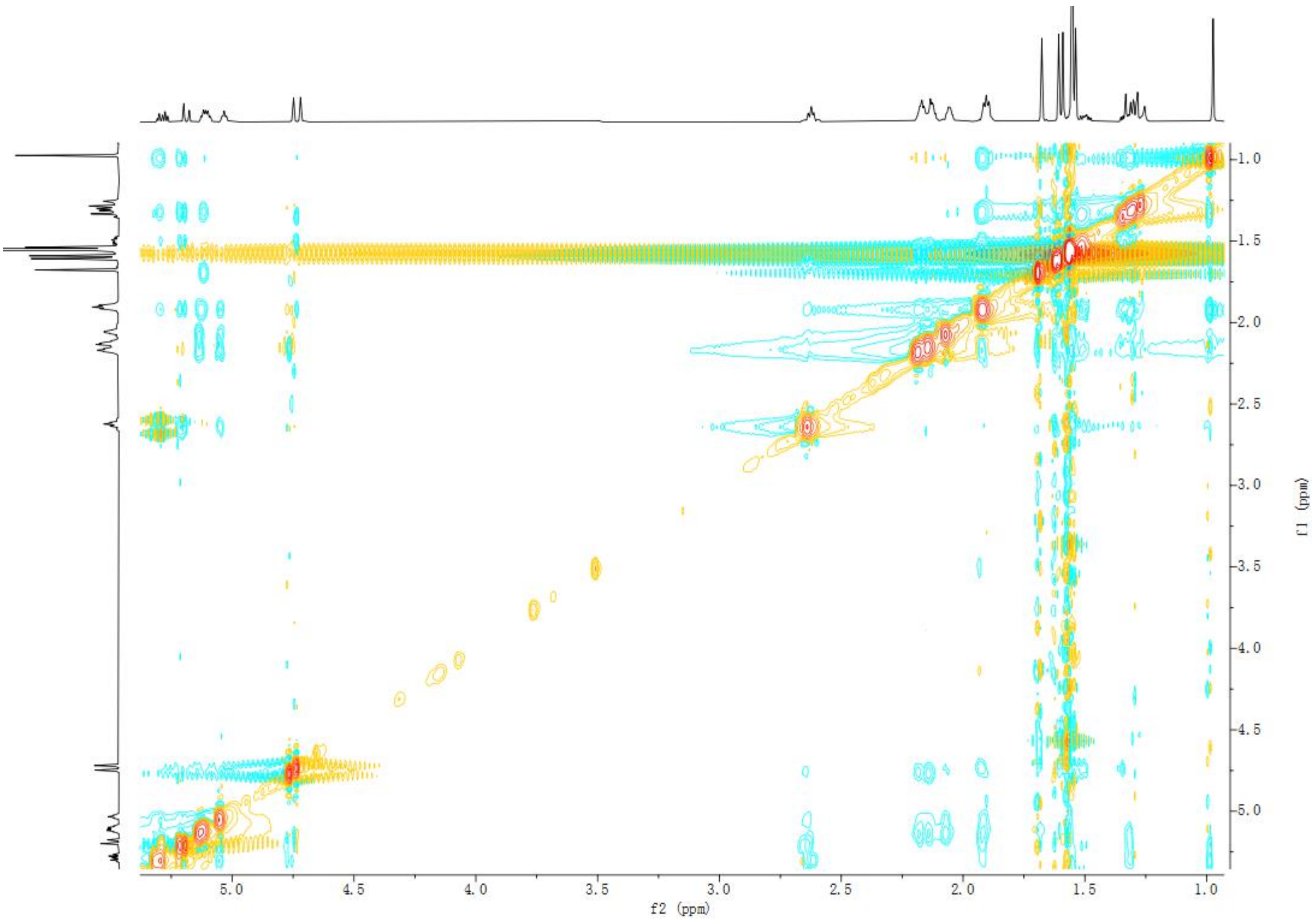

Figure S120. NOESY spectrum of compound **17** in $CDCl_3$

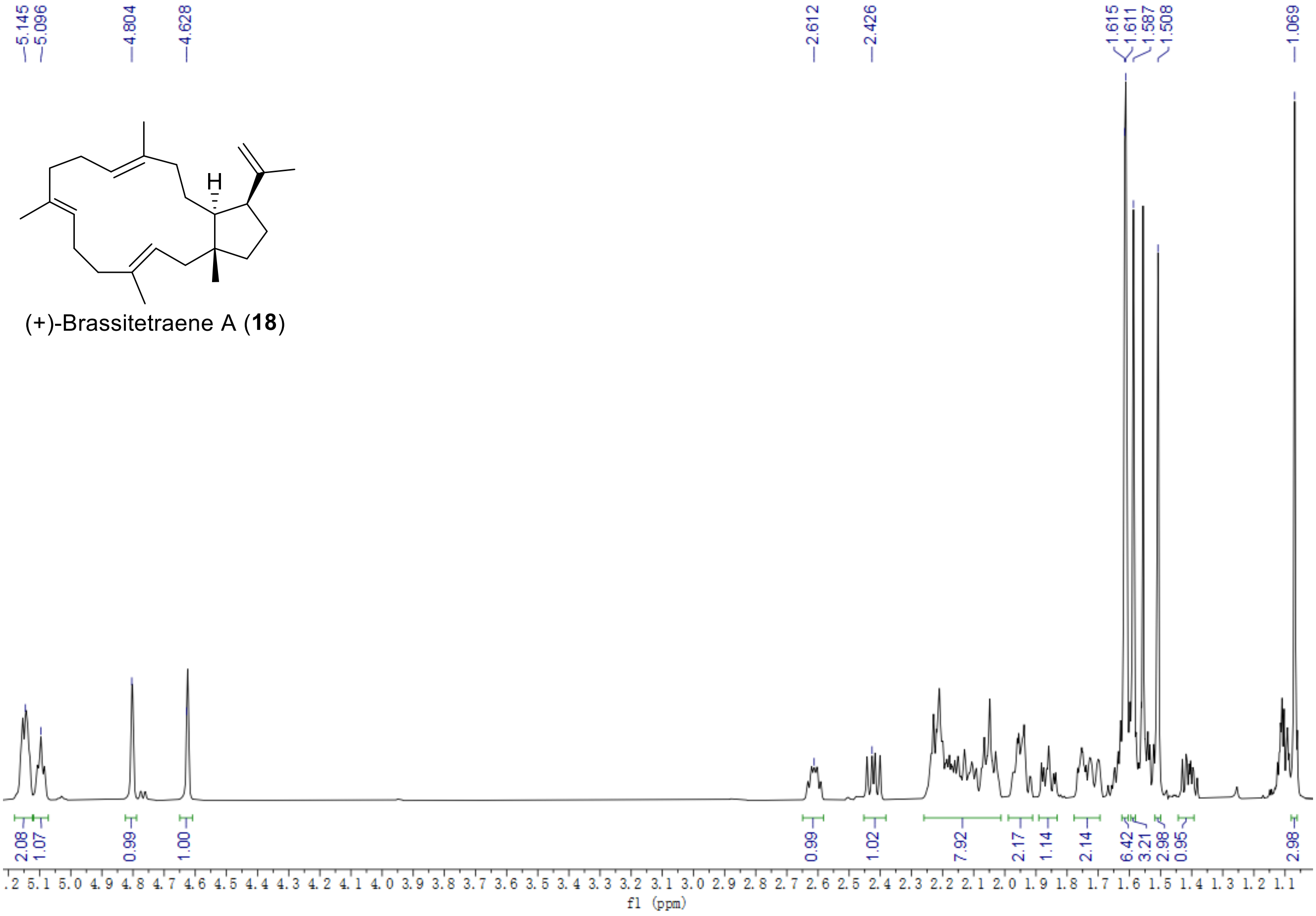

Figure S121. ^{1}H NMR spectrum of compound **18** in $CDCl_3$ (700 MHz)

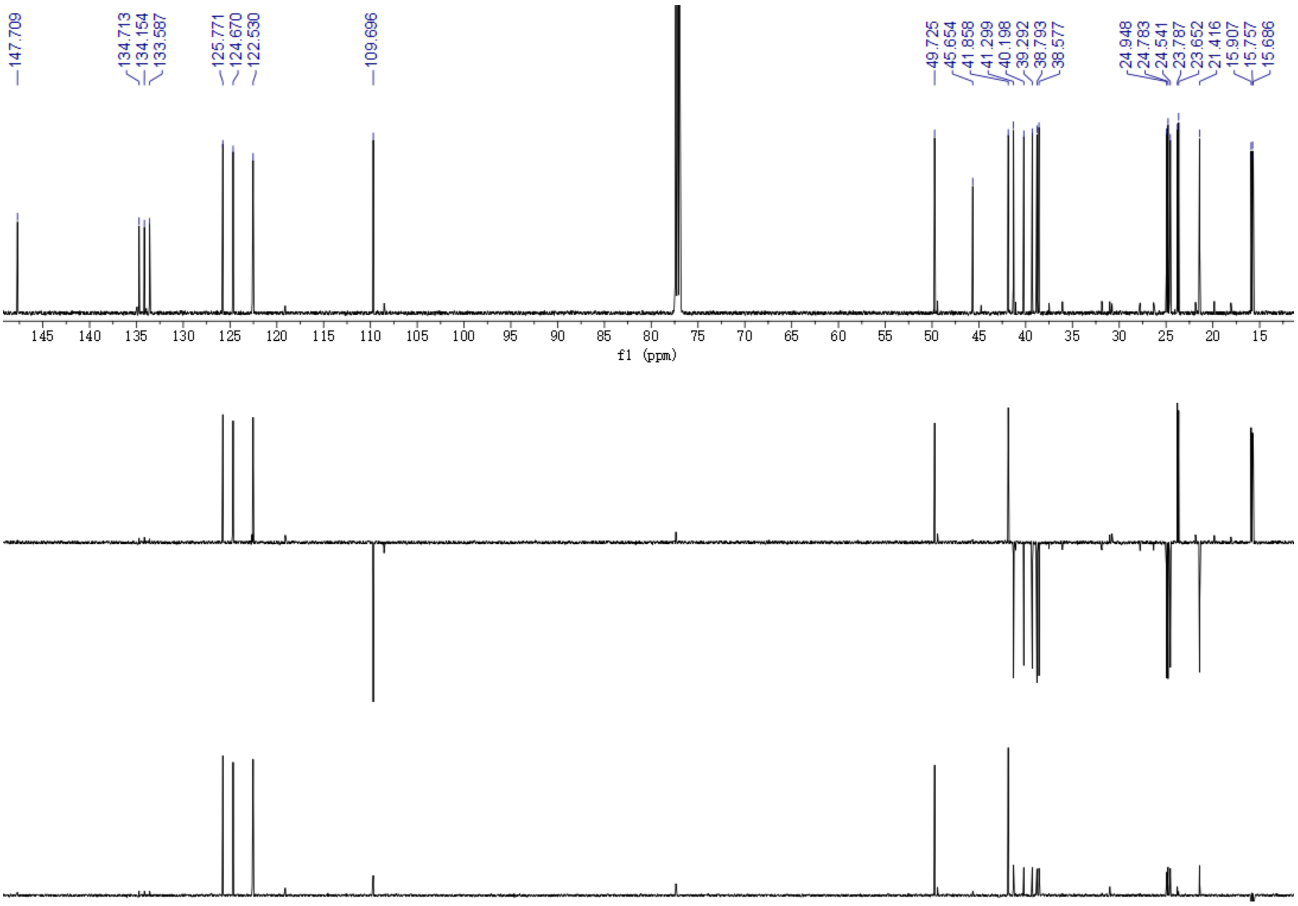

Figure S122. ^{13}C NMR and DEPT spectra of compound **18** in $CDCl_3$ (150 MHz)

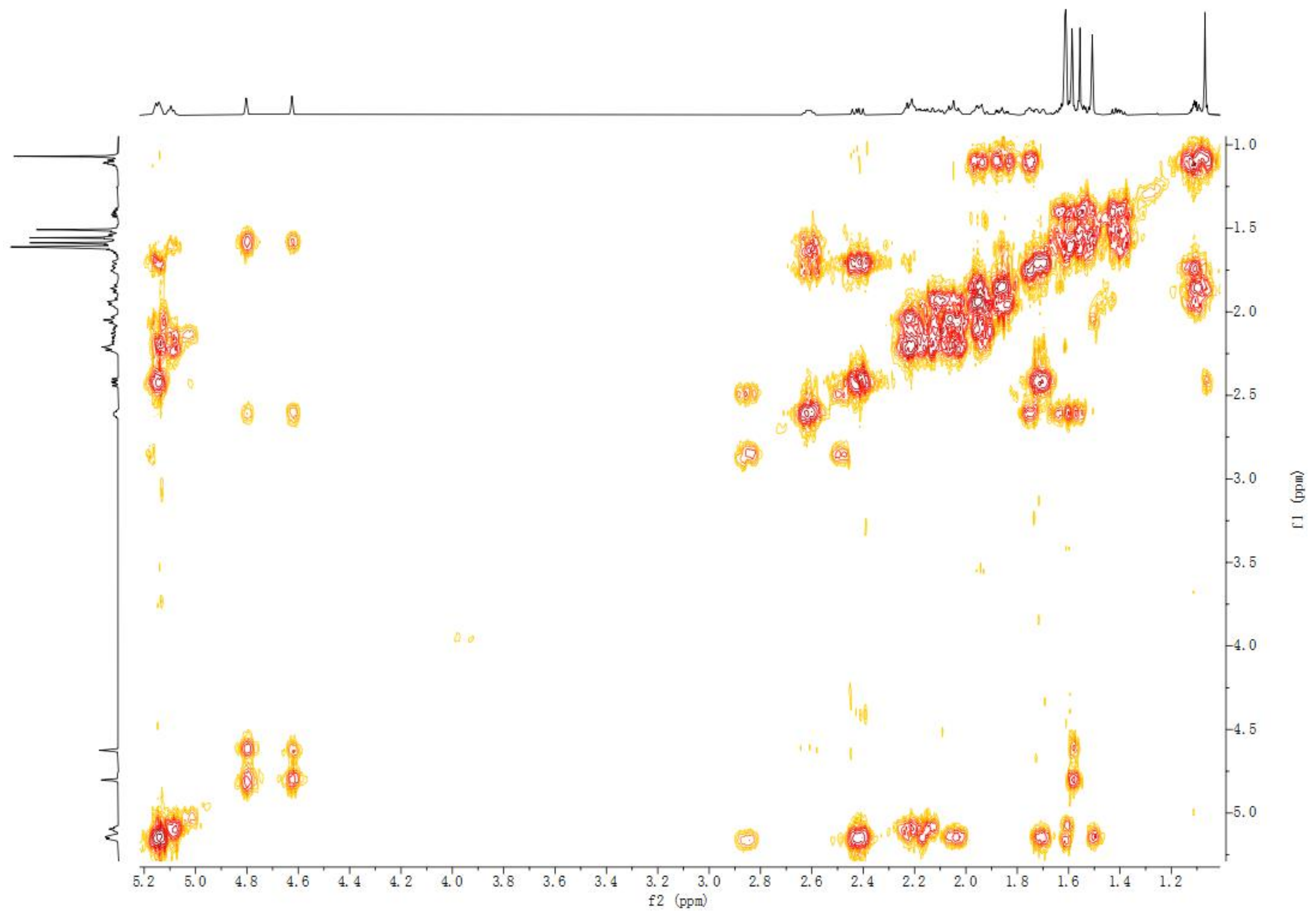

Figure S123. ^{1}H-^{1}H COSY spectrum of compound **18** in $CDCl_3$

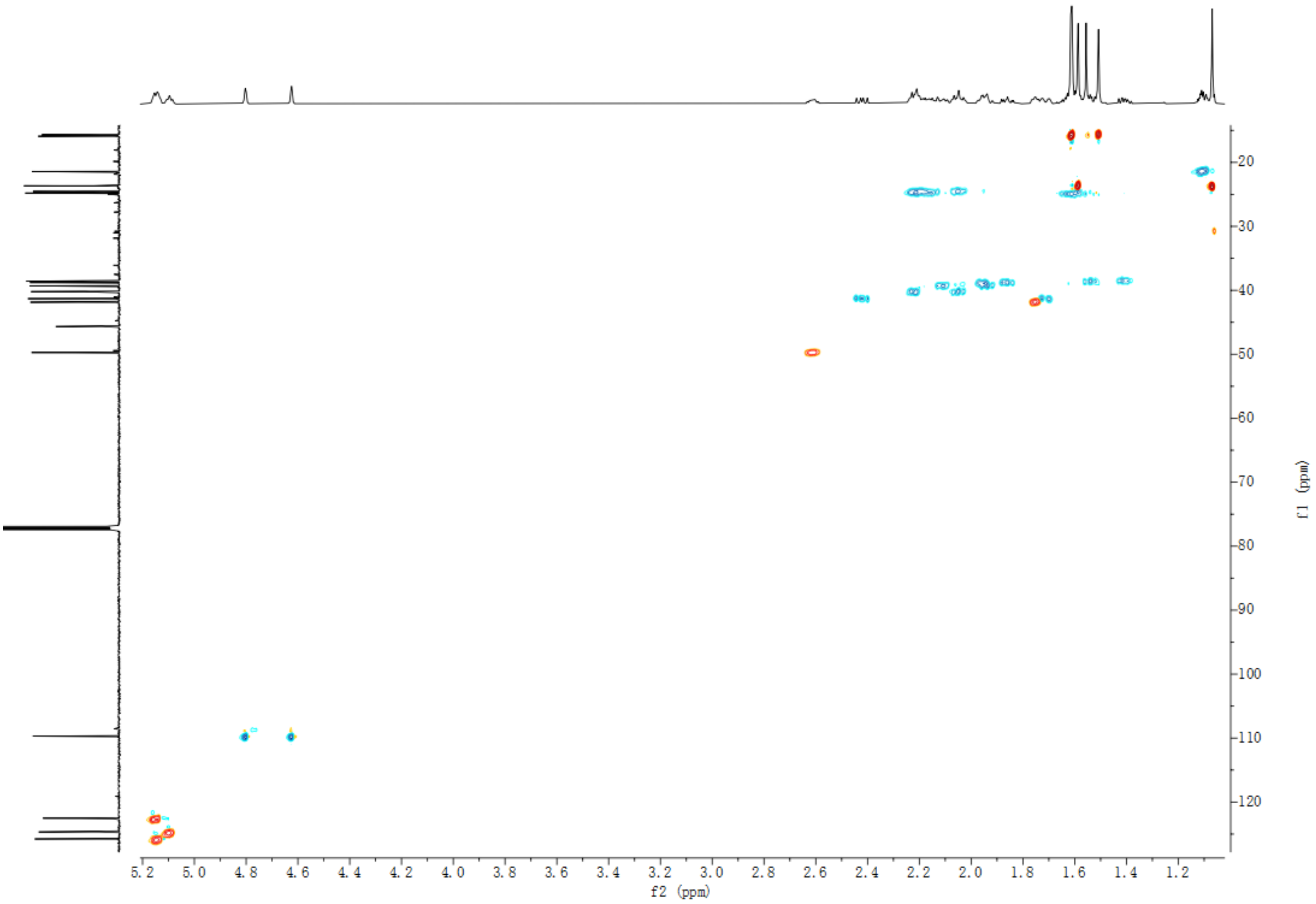

Figure S124. HSQC spectrum of compound **18** in $CDCl_3$

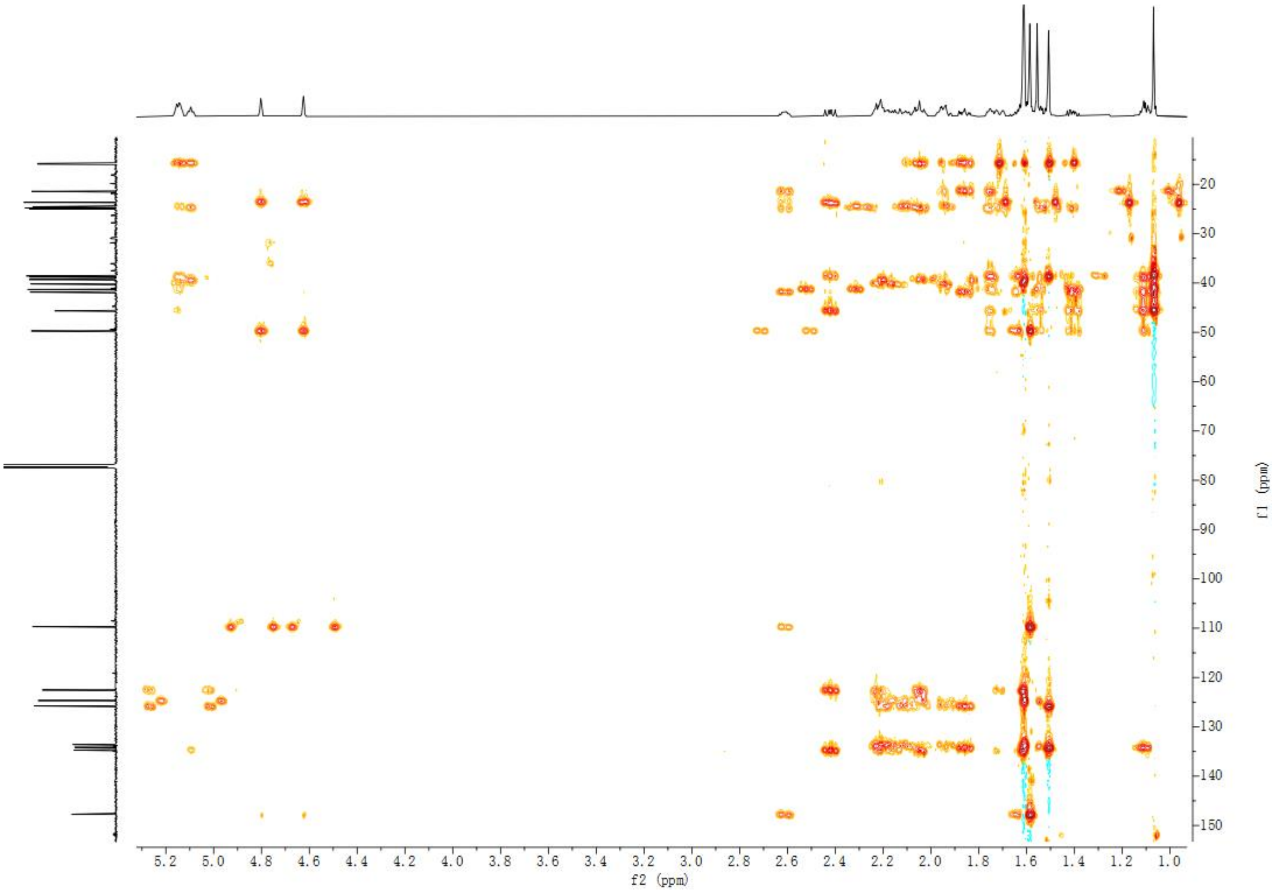

Figure S125. HMBC spectrum of compound **18** in $CDCl_3$

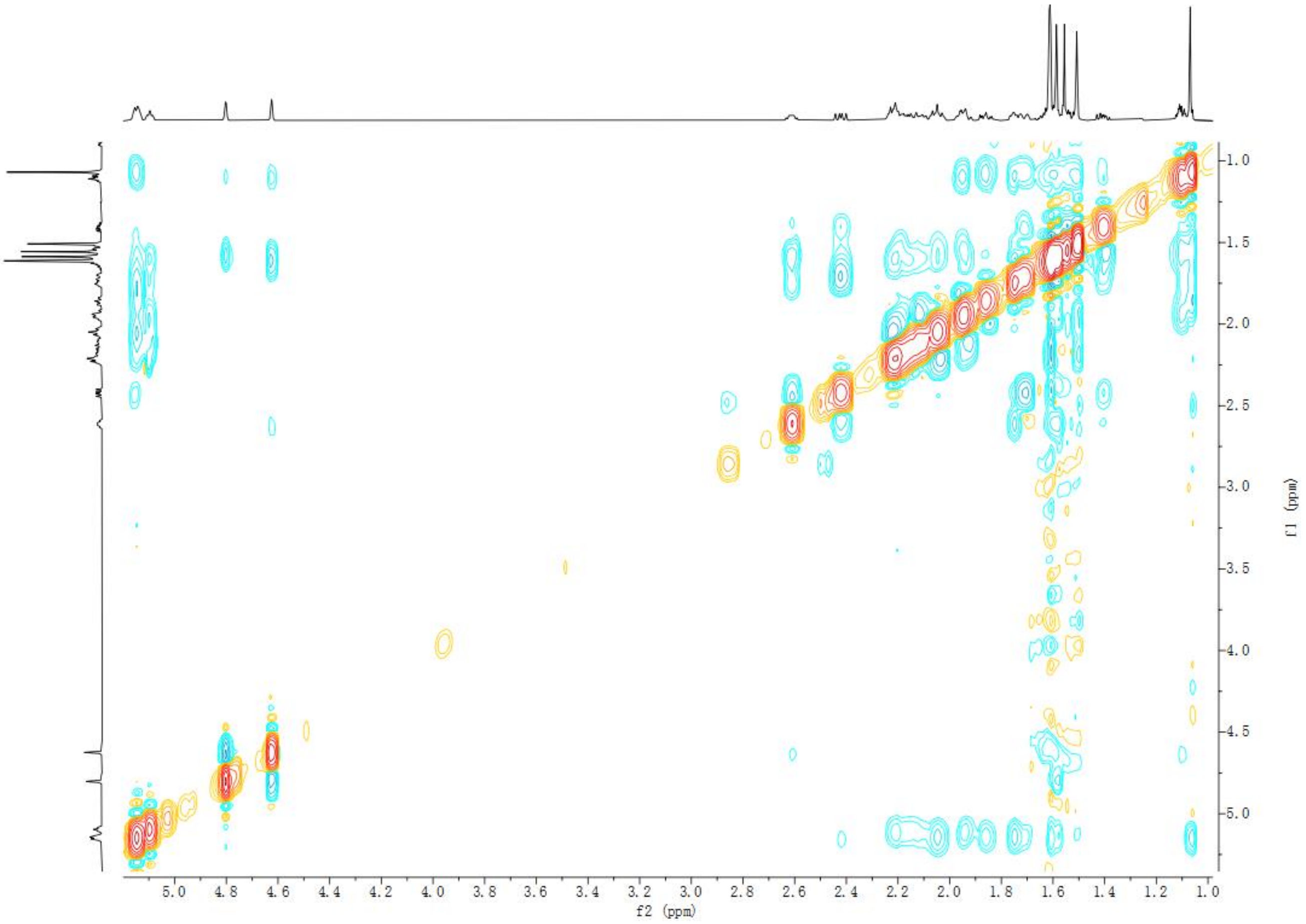

Figure S126. NOESY spectrum of compound **18** in $CDCl_3$

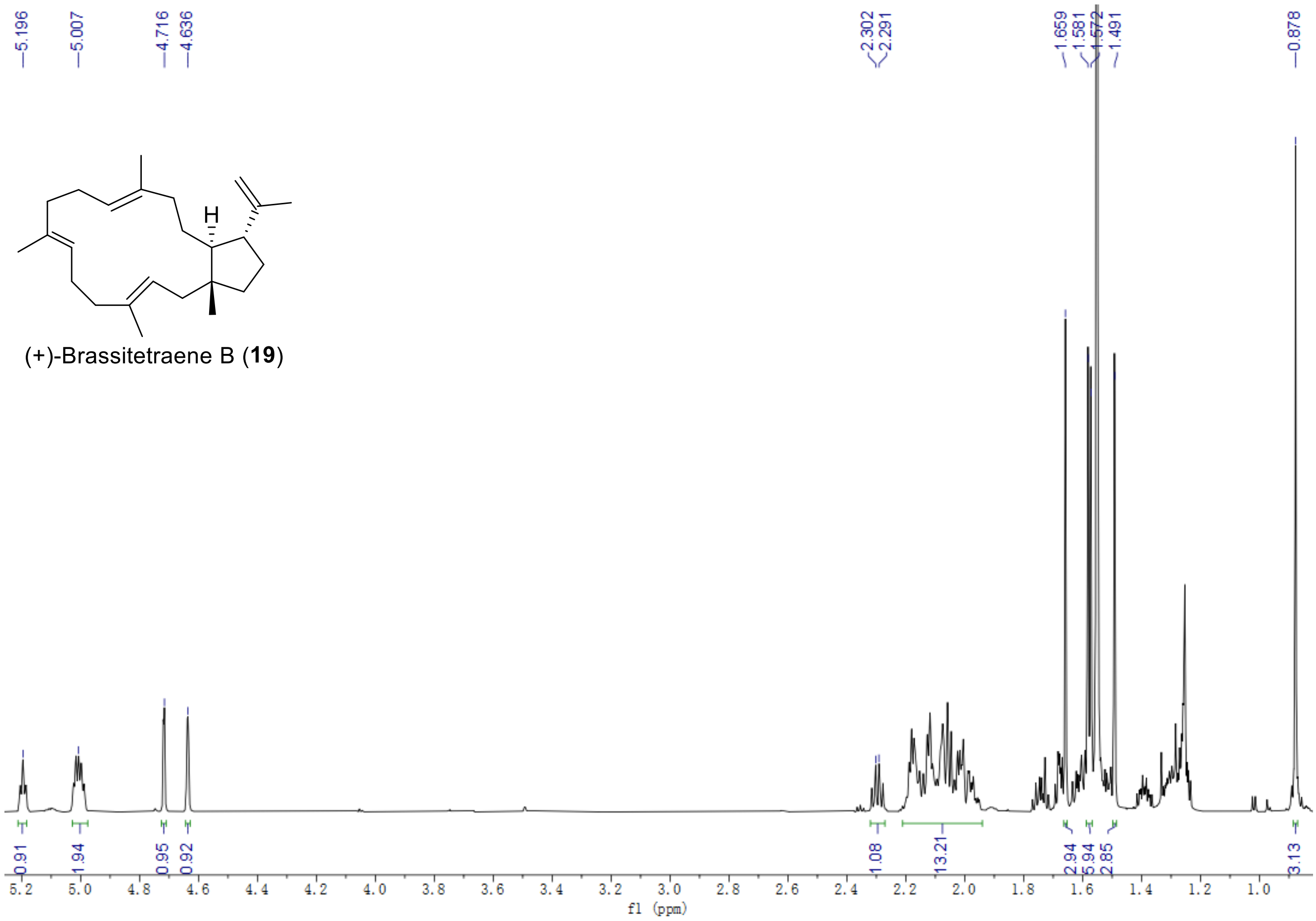

Figure S127. ^{1}H NMR spectrum of compound **19** in $CDCl_3$ (700 MHz)

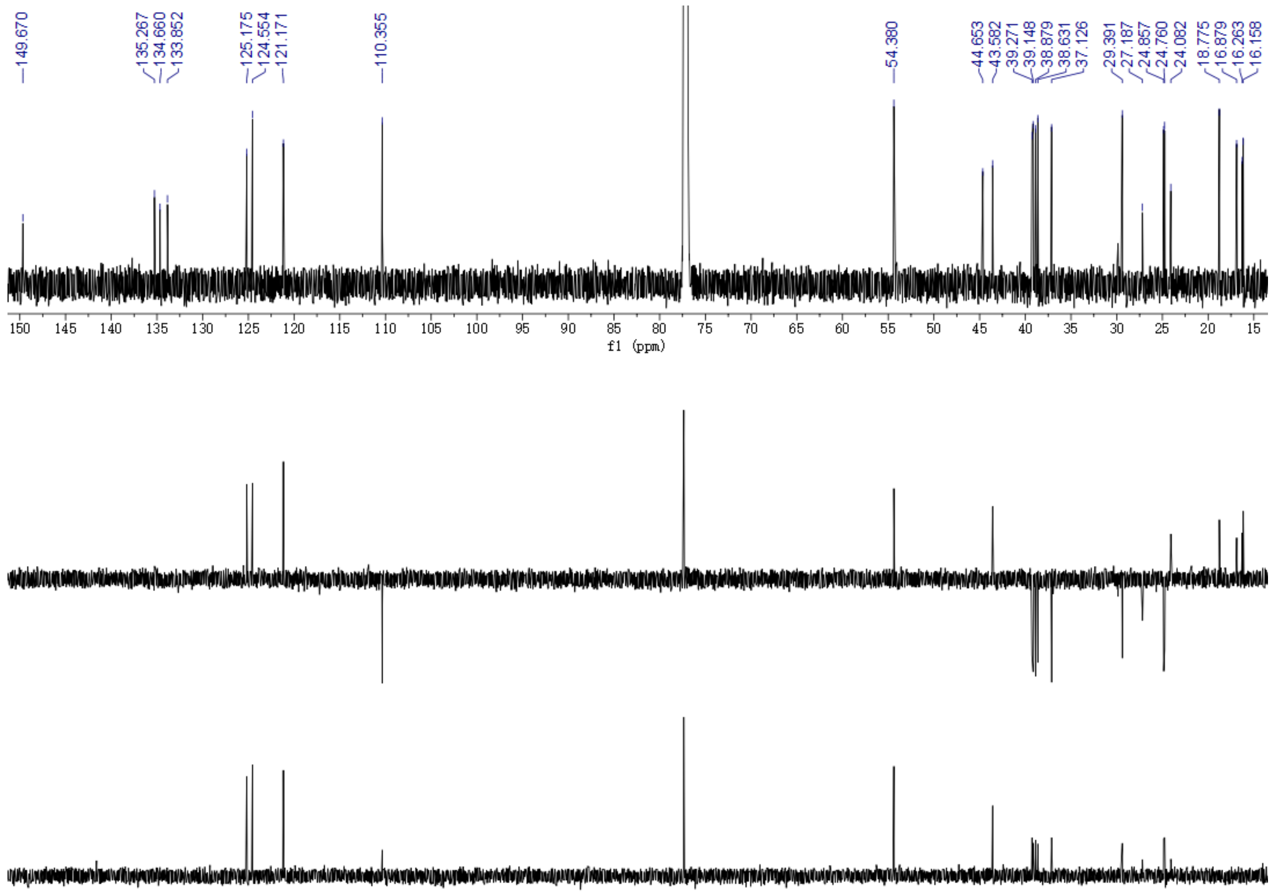

Figure S128. ^{13}C NMR and DEPT spectra of compound **19** in $CDCl_3$ (150 MHz)

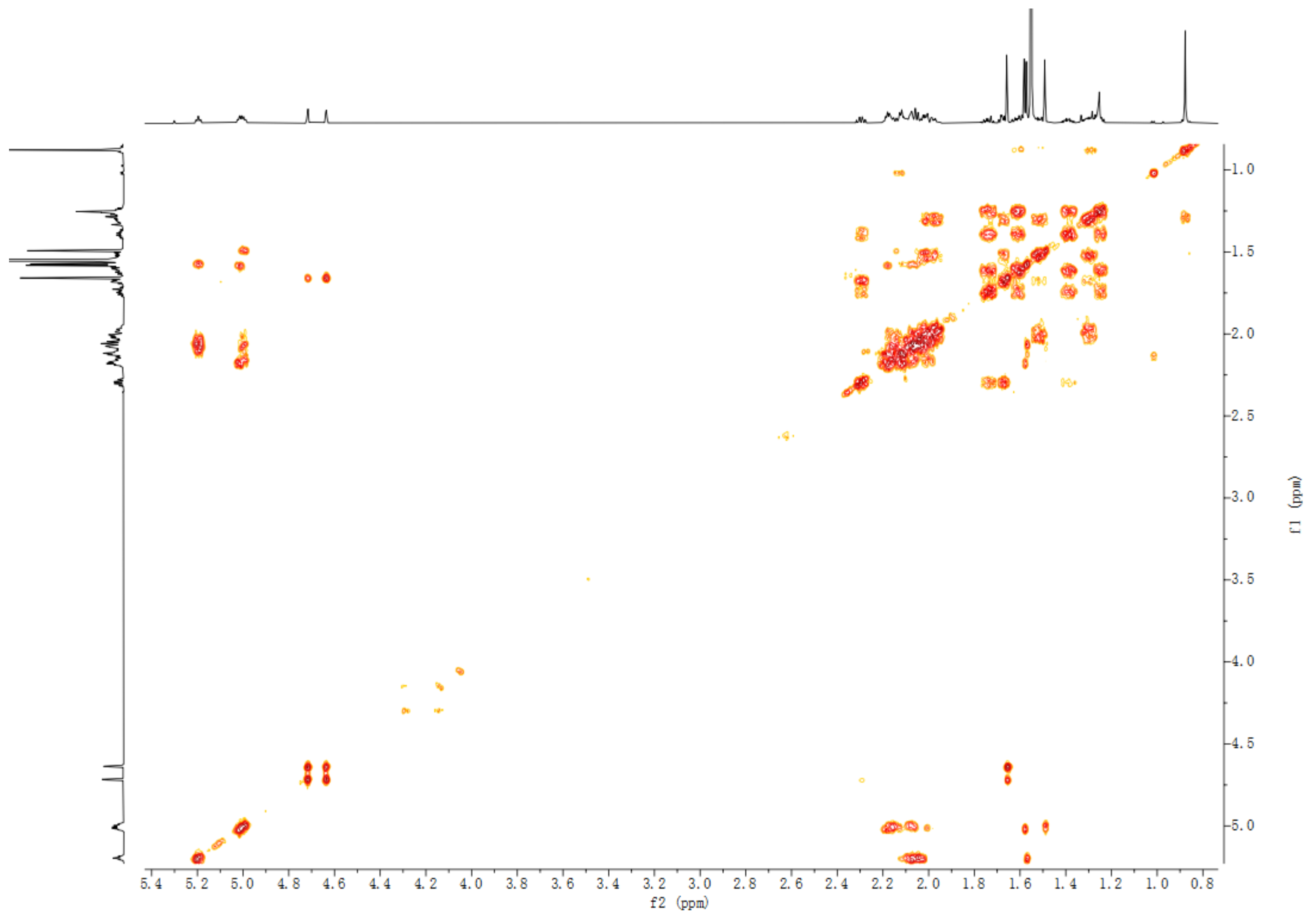

Figure S129. ^{1}H-^{1}H COSY spectrum of compound **19** in $CDCl_3$

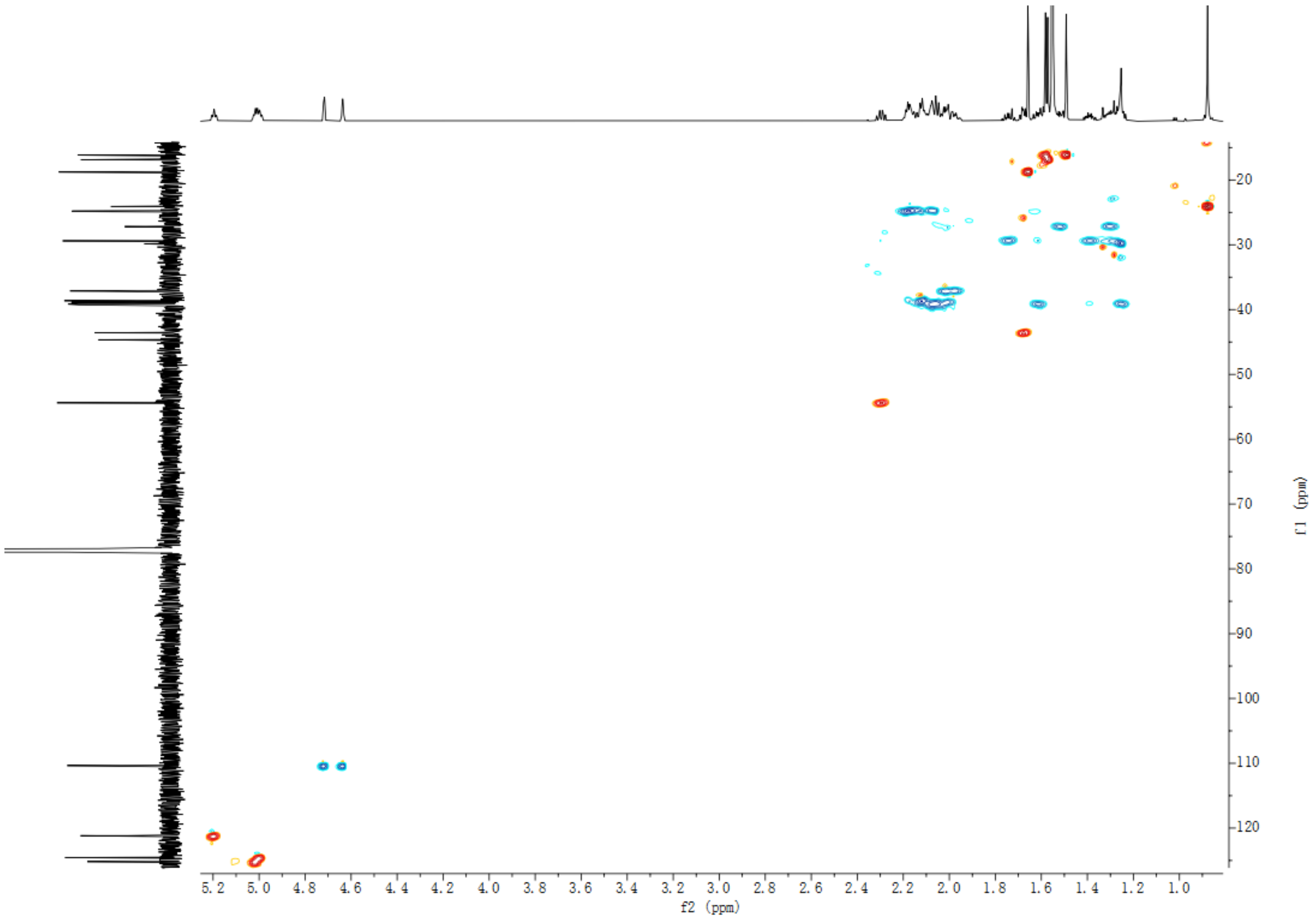

Figure S130. HSQC spectrum of compound **19** in $CDCl_3$

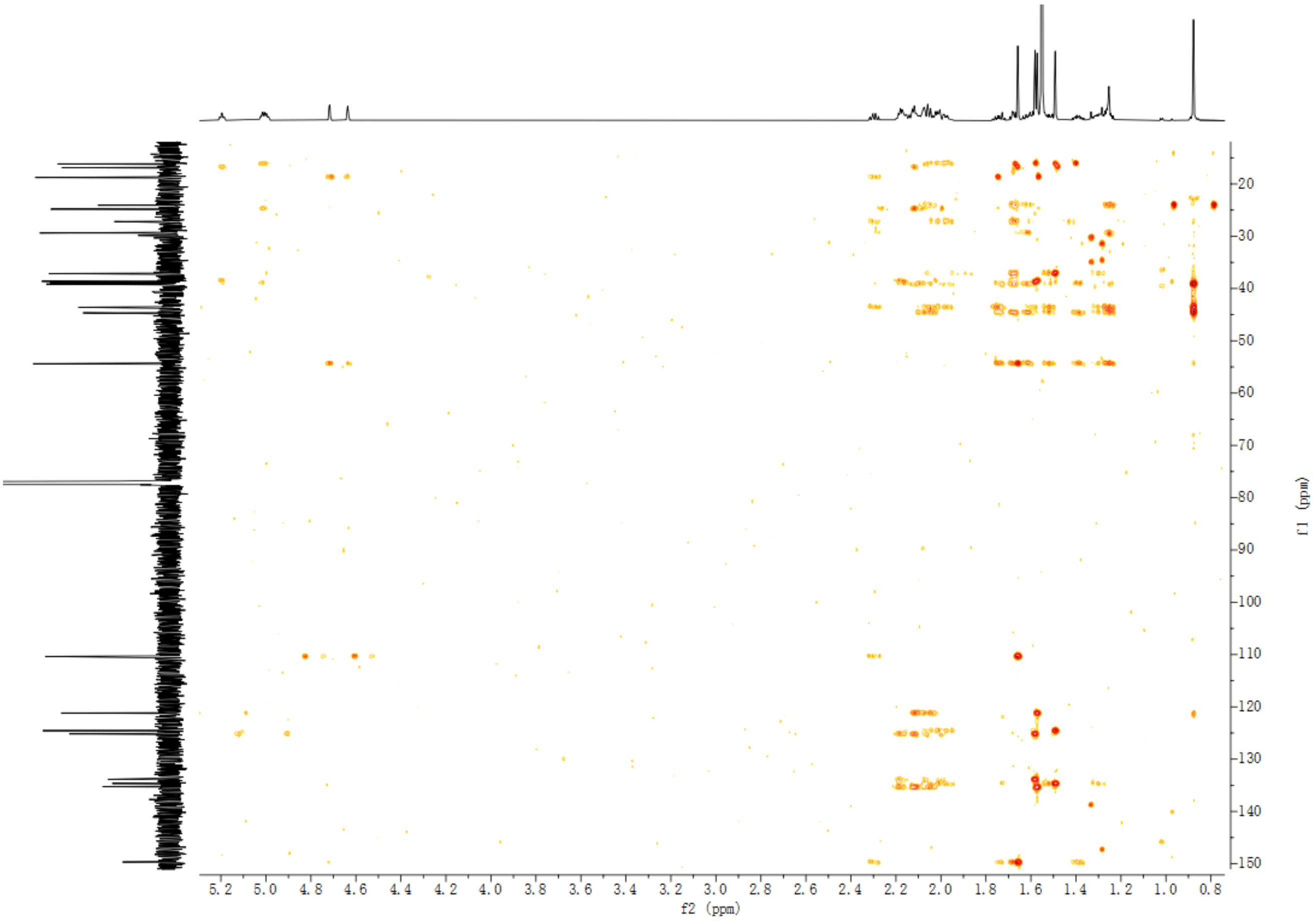

Figure S131. HMBC spectrum of compound **19** in $CDCl_3$

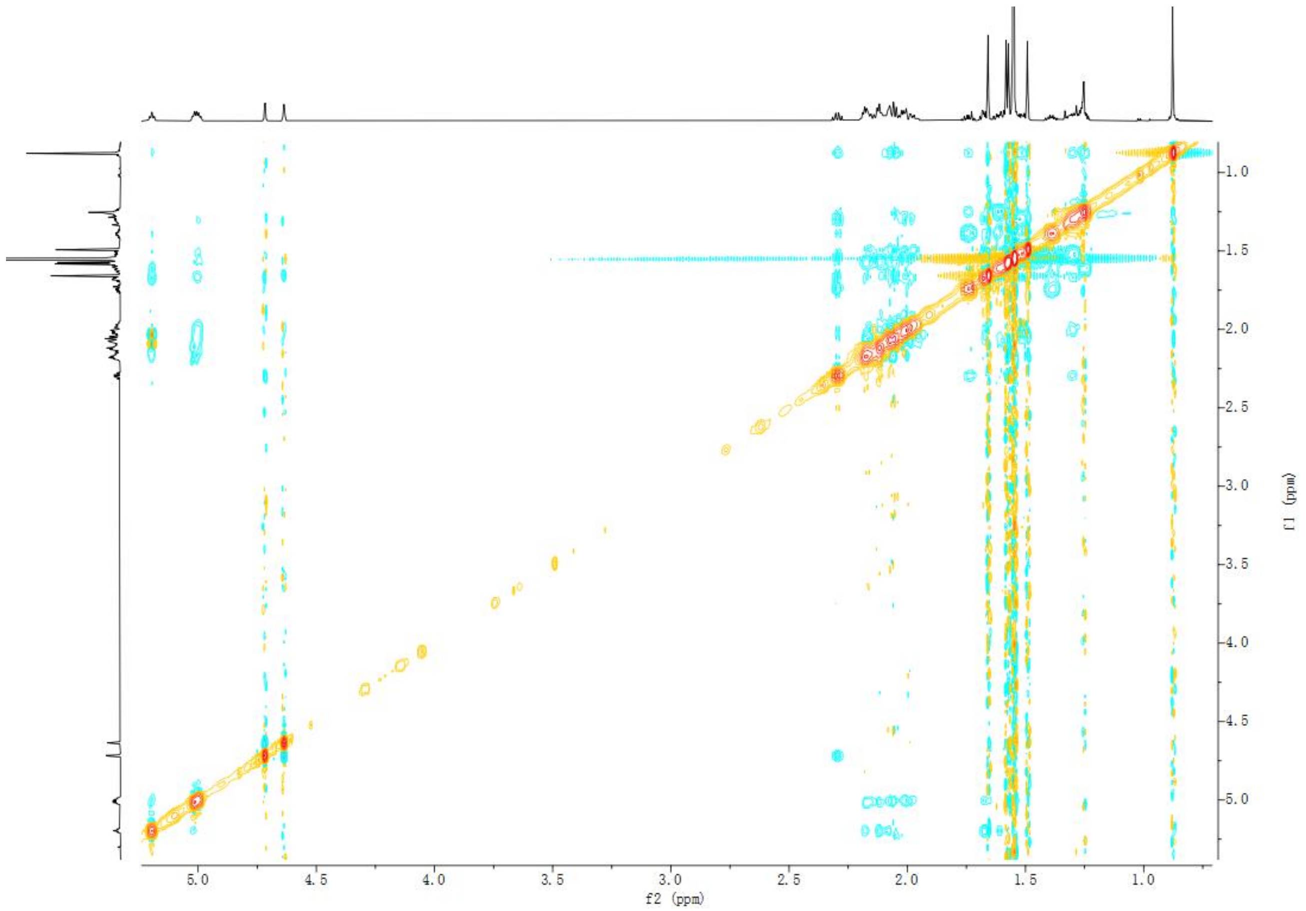

Figure S132. NOESY spectrum of compound **19** in $CDCl_3$

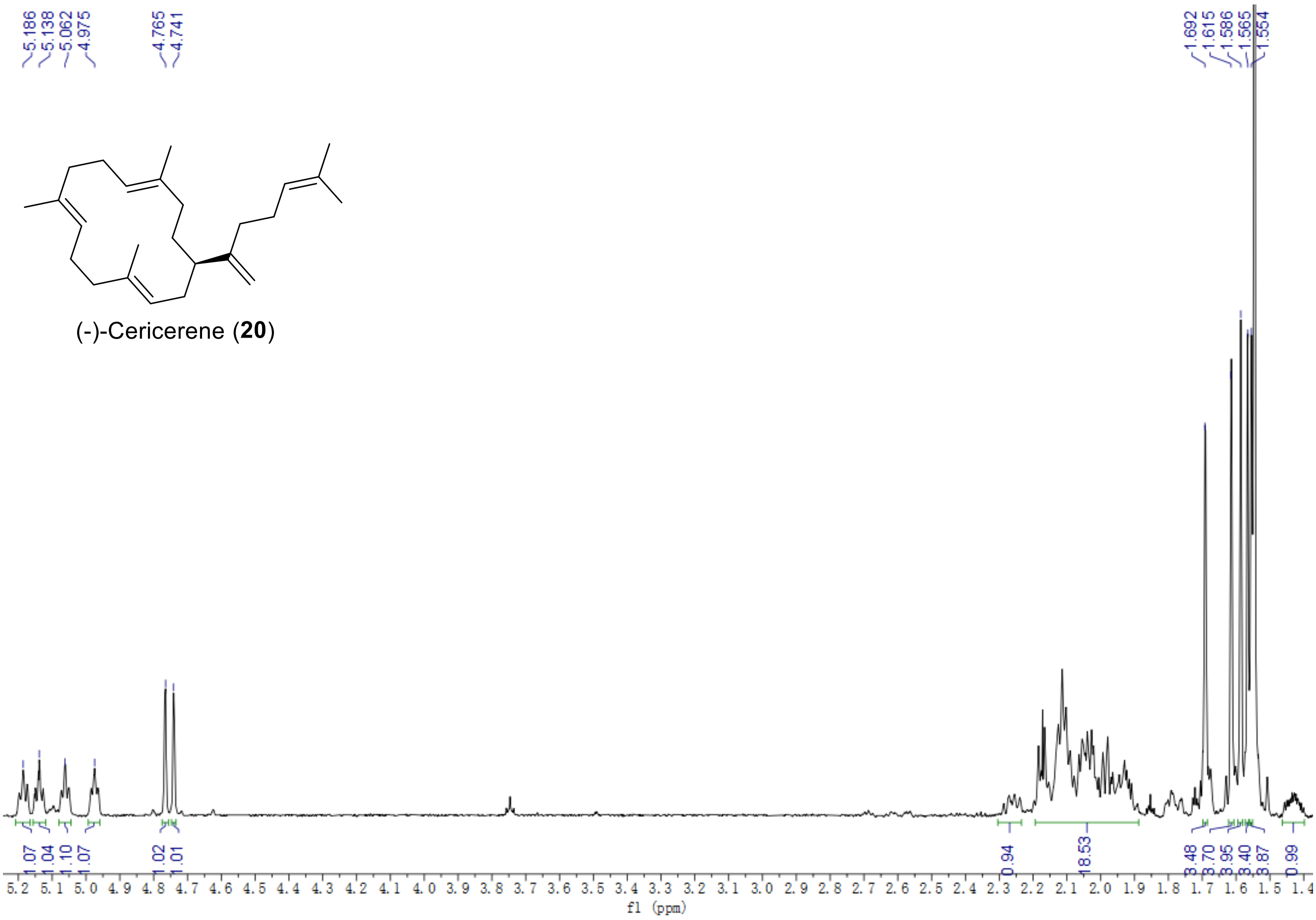

Figure S133. ^{1}H NMR spectrum of compound **20** in $CDCl_3$ (700 MHz)

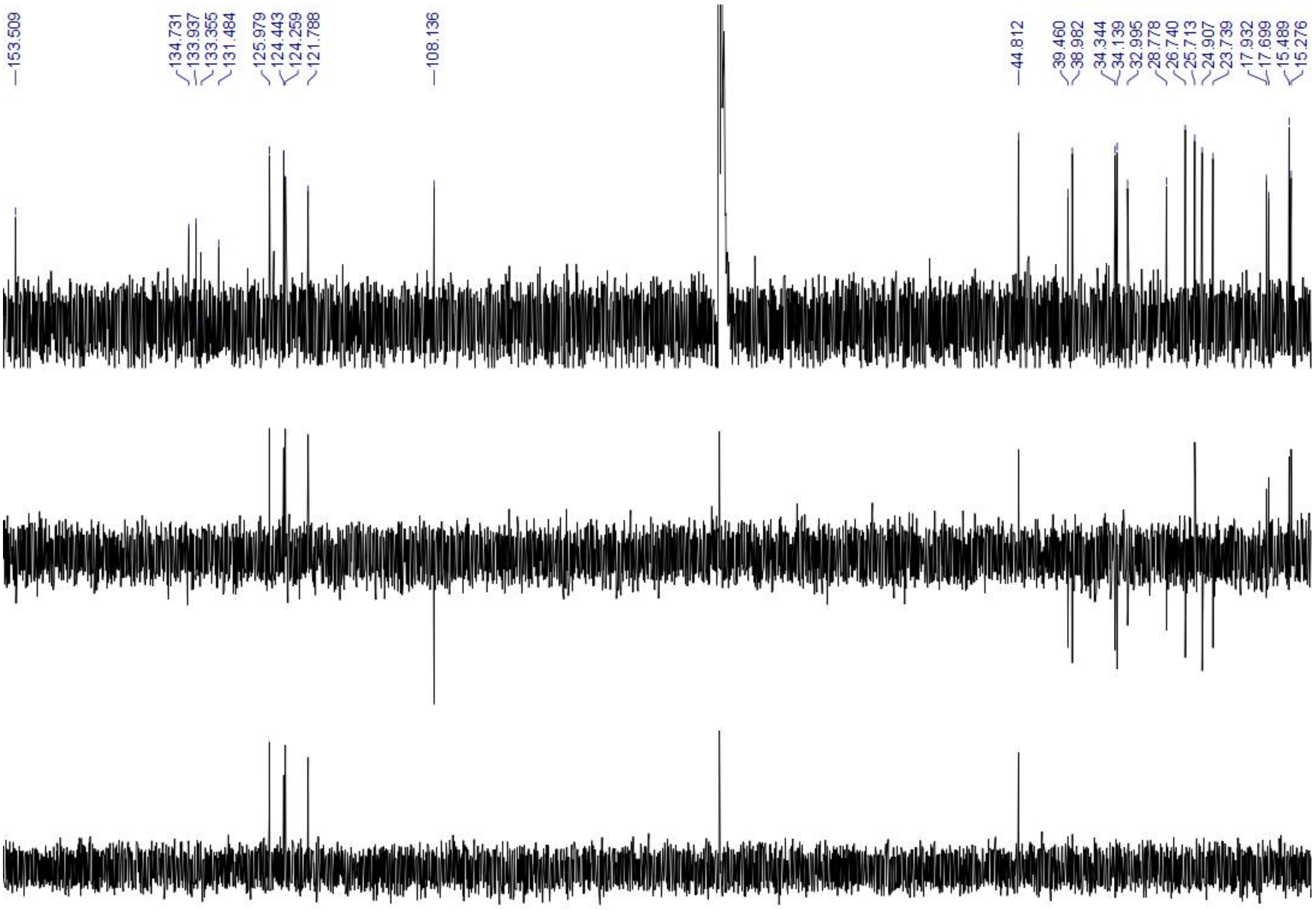

Figure S134. ^{13}C NMR and DEPT spectra of compound **20** in $CDCl_3$ (150 MHz)
